# Prediction of DNase I Hypersensitive Sites by Using Pseudo Nucleotide Compositions

**DOI:** 10.1155/2014/740506

**Published:** 2014-08-19

**Authors:** Pengmian Feng, Ning Jiang, Nan Liu

**Affiliations:** School of Public Health, Hebei United University, Tangshan 063000, China

## Abstract

DNase I hypersensitive sites (DHS) associated with a wide variety of regulatory DNA elements. Knowledge about the locations of DHS is helpful for deciphering the function of noncoding genomic regions. With the acceleration of genome sequences in the postgenomic age, it is highly desired to develop cost-effective computational methods to identify DHS. In the present work, a support vector machine based model was proposed to identify DHS by using the pseudo dinucleotide composition. In the jackknife test, the proposed model obtained an accuracy of 83%, which is competitive with that of the existing method. This result suggests that the proposed model may become a useful tool for DHS identifications.

## 1. Introduction

DNase I hypersensitive sites (DHS) are regions of chromatin which are sensitive to cleavage by the DNase I enzyme. Since the discovery of DHSs in 1980s [1], they have been used as markers of regulatory DNA regions. In general, these specific regions are generally nucleosome-free and associate with a wide variety of genomic regulatory elements, such as promoters, enhancers, insulators, silencers, and suppressors [2–4]. Therefore, mapping of DHS has become an effective approach for discovering functional DNA elements from the noncoding sequences.

Although the traditional Southern blotting technique is a gold-standard approach for identifying DHS, obtaining information from Southern blot approach is a tricky, time-consuming, and inaccurate task [5]. Recently, the DNase-seq technique (combination of DNase I digestion and high-throughput sequencing) has been proposed [6] and this technique allows for an unprecedented increase in resolution. However, methodologies for the analysis of DNase-seq data are relatively immature [7]. Therefore, computational models will be an important complement to experimental techniques for identifying DHS.

Based on nucleotide compositions, a support vector machine model for identifying DHS in K562 cell line was proposed [8]. This method yielded quite encouraging results and did play a role in stimulating the development of this area. However, further work is needed due to the following reasons. First, the sequences in their dataset share high sequence similarities. Second, the DNA structural properties were ignored. To solve these problems, we proposed a new model for identifying DHS, which is trained on a high quality benchmark dataset. In the new model, each DNA sample is encoded by using the pseudo dinucleotide composition, into which the DNA structural properties are incorporated.

## 2. Materials and Methods

### 2.1. Benchmark Dataset

The experimentally confirmed 280 DHS and 731 non-DHS sequences were obtained from http://noble.gs.washington.edu/proj/hs/, which have been used to train DHS prediction models [8]. As elucidated in [9], a predictor, if trained and tested by a dataset containing redundant samples with high similarity, might yield misleading results with an overestimated accuracy. To get rid of the redundancy and avoid bias, the CD-HIT software [10] was utilized to remove those DNA fragments that have ≥60% pairwise sequence identity to each other.

Finally, we obtained 247 positive and 710 negative samples for the benchmark dataset *S*, as can be formulated by

(1) $$\begin{array}{c} S=S^{+}⋃S^{-}, \end{array}$$

where the subset *S* ^+^ contains 247 DHS sequences and *S* ^−^ contains 710 non-DHS sequences, while ⋃ represents the “union” in the set theory. The detailed sequences in the benchmark dataset *S* are given in Supplementary Information S1 available online at http://dx.doi.org/10.1155/2014/740506.

### 2.2. DNA Sequence Representation

In order to integrate the sequence-order effects and DNA physicochemical properties together, the pseudo nucleotide composition was proposed in 2011 [11]. Since then, the concept of pseudo nucleotide composition has penetrated into many branches of computational genomics, such as predicting the recombination spots [12], predicting promoters [13], predicting nucleosome positioning sequences [14], and identifying splice sites [15]. Because of its wide and increasing usage, recently, a flexible web-server, called “pseudo *K*-tuple nucleotide composition (PseKNC),” was developed [16], which can be used to generate various kinds of pseudo *K*-tuple nucleotide compositions.

Encouraged by the success of introducing pseudo nucleotide composition to computational genomics, in the current study, the pseudo dinucleotide composition was used to represent DNA sequences in the benchmark dataset, which can be expressed as [12, 16]

(2) $$\begin{array}{c} D={\left[\begin{array}{ccccccc} d_{1} & d_{2} & \cdots & d_{16} & d_{16+1} & \cdots & d_{16+\lambda } \end{array}\right]}^{T}, \end{array}$$

where

(3) $$\begin{array}{c} d_{u}=\{\begin{array}{cc} \frac{f_{u}}{\sum_{i=1}^{16}f_{i}+w\sum_{j=1}^{\lambda }\theta_{j}}, & \begin{array}{cc} & \left(1\leq u\leq 16\right) \end{array},\\ \frac{w\theta_{u-16}}{\sum_{i=1}^{16}f_{i}+w\sum_{j=1}^{\lambda }\theta_{j}}, & \left(16<u\leq 16+\lambda \right). \end{array} \end{array}$$

In (3), *f*_*u*_  (*u* = 1,2,…, 16) is the normalized occurrence frequency of the dinucleotides in the DNA sequence. *λ* is the number of the total counted ranks (or tiers) of the correlations along a DNA sequence, and *w* is the weight factor. The concrete values for *λ* and *w* as well as *k* will be further discussed in Section 3.1, while the correlation factor *θ*_*j*_ represents the *j-*tier structural correlation factor between all the *j*th most contiguous dinucleotide *R*_*i*_ *R*_*i*+1_ at position *i*.

### 2.3. Support Vector Machine (SVM)

SVM is a supervised learning algorithm and has been widely used in computational genomics and proteomics [17–23]. The basic principle of SVM is to transform the input vector into a high dimension space and then seek a separating hyperplane with the maximal margin in this space by using the decision function

(4) $$\begin{array}{c} f\left(\vec{X}\right)=\mathrm{sgn}\left(\overset{N}{\underset{i=1}{\sum }}y_{i}\alpha_{i}\cdot K\left(\vec{X},{\vec{X}}_{i}\right)+b\right), \end{array}$$

where *α*_*i*_ is the Lagrange multipliers, *b* is the offset, $\vec{X_{i}}$ is the *i*th training vector, and *y*_*i*_ represents the type of the *i*th training vector. $K(\vec{X},\vec{X_{i}})$ is a kernel function which defines an inner product in a high dimensional feature space, and sgn is the sign function. Due to its effectiveness and speed in nonlinear classification process, the radial basis kernel function (RBF) $K(\vec{X_{i}},\vec{X_{j}})=\exp\left(-\gamma {||{\vec{X}}_{i},\vec{X_{j}}||}^{2}\right)$ was used in the current study.

The Libsvm 2.84 package [24] was used to perform the SVM, which can be downloaded from http://www.csie.ntu.edu.tw/~cjlin/libsvm/. The regularization parameter *C* and the kernel width parameter *γ* were optimized via an optimization procedure using a grid search. The search spaces for *C* and *γ* are [2^15^, 2^−5^] and [2^−5^, 2^−15^] with steps of 2^−1^ and 2, respectively.

### 2.4. Performance Evaluation

Three cross-validation methods, that is, independent dataset test, subsampling (or *K*-fold cross-validation) test, and jackknife test, are often used to evaluate the anticipated success rate of a predictor. Among the three methods, the jackknife test is deemed the least arbitrary and most objective one [9, 25] and, hence, has been widely recognized and increasingly adopted by investigators to examine the quality of various predictors [26–30]. Accordingly, the jackknife test was used to examine the performance of the model proposed in the current study. In the jackknife test, each sequence in the training dataset is in turn singled out as an independent test sample and all the rule-parameters are calculated without including the one being identified.

A set of parameters, namely, sensitivity (Sn), specificity (Sp), Matthew's correlation coefficient (MCC), and accuracy (Acc), are used to evaluate the performance of the proposed model and they are defined as follows:

(5) $$\begin{array}{c} \text{Sn}=\frac{\text{TP}}{\text{TP}+\text{FN}}, \end{array}$$

(6) $$\begin{array}{c} \text{Sp}=\frac{\text{TN}}{\text{TN}+\text{FP}}, \end{array}$$

(7) $$\begin{array}{c} \text{MCC}=\frac{\text{TP}\times \text{TN}-\text{FP}\times \text{FN}}{\left(\text{TP}+\text{FN}\right)\times \left(\text{TN}+\text{FN}\right)\times \left(\text{TP}+\text{FP}\right)\times \left(\text{TN}+\text{FP}\right)}, \end{array}$$

(8) $$\begin{array}{c} \text{Acc}=\frac{\text{TP}+\text{TN}}{\text{TP}+\text{FN}+\text{TN}+\text{FP}}, \end{array}$$

where TP, TN, FP, and FN represent the number of the correctly recognized DHS, the number of the correctly recognized non-DHS, the number of non-DHS recognized as DHS, and the number of DHS recognized as non-DHS, respectively.

## 3. Results and Discussions

### 3.1. Parameter Optimization

**Figure 1:**
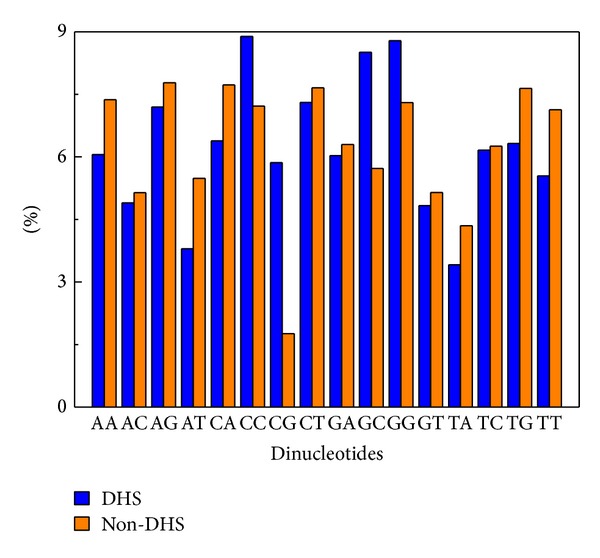
Comparative frequencies of 16 dinucleotides in DHS and non-DHS sequences.

By analyzing the dinucleotide composition of DHS and non-DHS sequences, we found that the frequency of CC, CG, GC, and GG is higher in DHS sequences, while the frequency of the remaining dinucleotides is higher in non-DHS (Figure 1). This is self-evident as to why the pseudo dinucleotide composition was used for the current case.

A series of evidences [12, 14, 31, 32] have demonstrated that DNA local structural properties, that is, angular parameters (twist, tilt, and roll) and translational parameters (shift, slide, and rise), are effective in identifying DNA attributes. Therefore, in the present work, the six structural parameters of dinucleotides were used to calculate the pseudo dinucleotide composition by using the PseKNC web-server, which is available at http://lin.uestc.edu.cn/pseknc/default.aspx.

As we can see from (1) and (2), the present model depends on the two parameters *w* and *λ*. *w* is the weight factor usually within the range from 0 to 1 and *λ* is the global order effect. Generally speaking, the greater the *λ* is, the more global sequence-order information the model contains. However, if *λ* is too large, it would reduce the cluster-tolerant capacity so as to lower down the cross-validation accuracy due to overfitting or “high dimension disaster” problem [33]. Therefore, our searching for the optimal values of the two parameters is in the range of *w* ∈ [0,1] and *λ* ∈ [1,10] with the steps of 0.1 and 1, respectively.

In order to reduce the computational time, the 5-fold cross-validation approach was used to optimize the two parameters together with the parameters *C* and *γ* of the SVM. We found that when *w* = 0.2 and *λ* = 6 with *C* = 512 and *γ* = 0.0078125, a peak was observed for the Acc. Accordingly, the two numerical values were used for the two uncertain parameters in the following analysis.

### 3.2. Prediction Quality

The prediction quality measured by the four metrics defined in (5)–(8) for the present model in identifying DHS in the benchmark dataset *S* via the rigorous jackknife test was listed in Table 1, where, for facilitating comparison, the corresponding results obtained by the previous predictor [8] on the same benchmark data set are also given. As we can see from Table 1, the current method outperformed the existing model in all the four metrics, indicating that our proposed method may become a useful tool in identifying DHS sequences.

## 4. Conclusions

Since DHS associates with a wide variety of functional elements, knowledge about the locations of DHS is helpful for deciphering the genomes. However, strong DNA sequence conservation is not observed among DHS sequences, suggesting that it is difficult to computationally identify DHS from primary DNA sequence.

A series of recent studies have demonstrated that the information coded by DNA structural properties is contributable to the identification of regulatory elements in genomes [12, 14, 31, 32]. Hence, in the present study, we proposed a SVM based model for identifying DHS by using the pseudo dinucleotide composition. In this model, we integrate dinucleotide composition with DNA structural properties. The predictive results of our model are better than existing methods. Therefore, it is anticipated that the proposed method may become a useful tool for identifying DHS sequences or, at the very least, it can play a complementary role to the existing methods in this area.

## Supplementary Material

Listed in Supplementary Information S1 are the 247 DHS and 710 non-DHS sequences of the benchmark dataset.

## Figures and Tables

**Table 1:** Comparison of different methods for identifying DHS by the jackknife test on the same benchmark dataset.

Predictor	Sn (%)	Sp (%)	Acc (%)	MCC
Our method	72.12	86.78	83.00	0.57
Noble et al.^a^	70.43	84.23	80.12	0.52

^a^From Noble et al. [8].

## References

[1] Wu C, M. Bingham P, Livak KJ, Holmgren R, Elgin SCR (1979). The chromatin structure of specific genes: I. Evidence for higher order domains of defined DNA sequence. *Cell*.

[2] Gross DS, Garrard WT (1988). Nuclease hypersensitive sites in chromatin. *Annual Review of Biochemistry*.

[3] Felsenfeld G, Groudine M (2003). Controlling the double helix. *Nature*.

[4] Felsenfeld G (1992). Chromatin as an essential part of the transcriptional mechanism. *Nature*.

[5] Crawford GE, Holt IE, Whittle J (2006). Genome-wide mapping of DNase hypersensitive sites using massively parallel signature sequencing (MPSS). *Genome Research*.

[6] Song L, Crawford GE (2010). DNase-seq: a high-resolution technique for mapping active gene regulatory elements across the genome from mammalian cells. *Cold Spring Harbor Protocols*.

[7] Madrigal P, Krajewski P (2012). Current bioinformatic approaches to identify DNase I hypersensitive sites and genomic footprints from DNase-seq data. *Frontiers in Genetics*.

[8] Noble WS, Kuehn S, Thurman R, Yu M, Stamatoyannopoulos J (2005). Predicting the in vivo signature of human gene regulatory sequences. *Bioinformatics*.

[9] Chou K (2011). Some remarks on protein attribute prediction and pseudo amino acid composition. *Journal of Theoretical Biology*.

[10] Fu L, Niu B, Zhu Z, Wu S, Li W (2012). CD-HIT: accelerated for clustering the next-generation sequencing data. *Bioinformatics*.

[11] Zhou X, Li Z, Dai Z, Zou X (2011). Predicting methylation status of human DNA sequences by pseudo-trinucleotide composition. *Talanta*.

[12] Chen W, Feng P, Lin H, Chou K (2013). IRSpot-PseDNC: identify recombination spots with pseudo dinucleotide composition. *Nucleic Acids Research*.

[13] Zhou X, Li Z, Dai Z, Zou X (2013). Predicting promoters by pseudo-trinucleotide compositions based on discrete wavelets transform. *Journal of Theoretical Biology*.

[14] Guo SH, Deng EZ, Xu LQ (2014). iNuc-PseKNC: a sequence-based predictor for predicting nucleosome positioning in genomes with pseudo k-tuple nucleotide composition. *Bioinformatics*.

[15] Chen W, Feng PM, Lin H, Chou KC (2014). iSS-PseDNC: identifying splicing sites using pseudo dinucleotide composition. *BioMed Research International*.

[16] Chen W, Lei TY, Jin DC, Lin H, Chou KC (2014). PseKNC: a flexible web server for generating pseudo K-tuple nucleotide composition. *Analytical Biochemistry*.

[17] Chen W, Lin H (2010). Prediction of midbody, centrosome and kinetochore proteins based on gene ontology information. *Biochemical and Biophysical Research Communications*.

[18] Lin H, Ding H (2011). Predicting ion channels and their types by the dipeptide mode of pseudo amino acid composition. *Journal of Theoretical Biology*.

[19] Liu B, Wang X, Chen Q, Dong Q, Lan X (2012). Using Amino Acid Physicochemical Distance Transformation for Fast Protein Remote Homology Detection. *PLoS ONE*.

[20] Liu B, Wang X, Lin L, Tang B, Dong Q (2009). Prediction of protein binding sites in protein structures using hidden Markov support vector machine. *BMC Bioinformatics*.

[21] Liu B, Wang X, Lin L, Dong Q, Wang X (2009). Exploiting three kinds of interface propensities to identify protein binding sites. *Computational Biology and Chemistry*.

[22] Chou KC, Cai YD (2002). Using functional domain composition and support vector machines for prediction of protein subcellular location. *The Journal of Biological Chemistry*.

[23] Hayat M, Khan A (2012). MemHyb: predicting membrane protein types by hybridizing SAAC and PSSM. *Journal of Theoretical Biology*.

[24] Chang CC, Lin CJ LIBSVM: a library for support vector machines. http://www.csie.ntu.edu.tw/~cjlin/libsvm/.

[25] Chou K-C, Zhang C-T (1995). Prediction of protein structural classes. *Critical Reviews in Biochemistry and Molecular Biology*.

[26] Esmaeili M, Mohabatkar H, Mohsenzadeh S (2010). Using the concept of Chou's pseudo amino acid composition for risk type prediction of human papillomaviruses. *Journal of Theoretical Biology*.

[27] Ding C, Yuan LF, Guo SH, Lin H, Chen W (2012). Identification of mycobacterial membrane proteins and their types using over-represented tripeptide compositions. *Journal of Proteomics*.

[28] Chen W, Lin H (2012). Identification of voltage-gated potassium channel subfamilies from sequence information using support vector machine. *Computers in Biology and Medicine*.

[29] Chou K, Wu Z, Xiao X (2011). iLoc-Euk: a multi-label classifier for predicting the subcellular localization of singleplex and multiplex eukaryotic proteins. *PLoS ONE*.

[30] Mohabatkar H, Mohammad Beigi M, Esmaeili A (2011). Prediction of GABAA receptor proteins using the concept of Chou's pseudo-amino acid composition and support vector machine. *Journal of Theoretical Biology*.

[31] Zuo Y, Li Q (2011). Identification of TATA and TATA-less promoters in plant genomes by integrating diversity measure, GC-Skew and DNA geometric flexibility. *Genomics*.

[32] Goñi JR, Pérez A, Torrents D, Orozco M (2007). Determining promoter location based on DNA structure first-principles calculations. *Genome Biology*.

[33] Wang T, Yang J, Shen H, Chou K (2008). Predicting membrane protein types by the LLDA algorithm. *Protein and Peptide Letters*.

