# Supplementary material for: Prediction of DNase I Hypersensitive Sites by Using Pseudo Nucleotide Compositions
Source: ScientificWorldJournal. 2014 Aug 19;2014:740506. doi: 10.1155/2014/740506 (PMC4152949; doi:10.1155/2014/740506)
Supplement: Supplementary file 1 — Listed in Supplementary Information S1 are the 247 DHS and 710 non-DHS sequences of the benchmark dataset. [file 740506.f1.pdf]

Supporting Information S1. The benchmark dataset $\mathbb{S}$ contains 247 DHS sequence segments and 710 non-DHS sequence segments.

I. The 247 TIS sequence segments

>DHS1
CTTCGCCAGCCACTCTTAGTCCGCCAGCGCGTGCGGCGGAGGCCGAGCGTCTCTATG
ATCCTGGCTTCTGGCAACGTCATCGTCACGCGCCGGATCCAACCCCCAACCACTTTA
GCCAGCTCTAGAGGCGCGCGTGGCCGGGACGGAAGTGCGCGCGGGTGTCGCCGGGAG
TGCGCGCTCCTCTGGCTGACGGGCGGGCCGGGCATGCGCCGCGGGCGTTTTGGCGGG
AAGCGCGGGGCGGGCCGGACAATGAGAGTGTCCGCCTCC
>DHS2
GTAGTCAGCAGAGCGGAATCTAGGAAGGCTCCTTTCCCGCGGCGCCCTGGAGGCGGG
GGCCCCACCTTCCCACGCAGGCGCTATCAAGCCCCGCCTCCTCACCCGCCCGCGGCG
TGGCGTCGGAAAGAGCCCTCAGCCCCTCCCTCTCTGGCGCTGATACCCAATGGGCAG
CCTCAGGCCTTTAGCGGGGGCGGGGCACCCCCTGGACGCCGTTCTGGTTGGCCCGCG
GCCCGGCGCAGCGCATGACGTTATTAC

>DHS3
CGCCTCCGAAAGAGGATAGGCCCAGCCTTTTTCCTTAGTCTAACCCCCGAGAAAGGC
CTGTGTGGGCAACGCCCCCGCAGTTGACCTATCAGCACTATCGCCACGCCCACCAGC
CAGAGTCCTGTCCTCTCAGAGGCTCCCTATACTTATCGATGGCCCCGCCCCCTCTGG
ATCTCTTACTCTAGCCCCGCCTGCTTCATTACTCCCTCCCCTTCCGCTCCCACACAC
CGTCCCCGCCCCATCTGTCCATCTCTTTCTAGC
>DHS4
GGAGTATGAGGCCGAATCTCATCCTCTAGTCCCAAGCCTCTCCACTACCAGGGCTGG
GTGGCTTCGCCAGGTAGAGCGCCGGCCCCTTTAAGAGCAAACGACCCGGACACGTCT
GCGAAGCTTGCGCGAAGAAGGGGAAGTTTGCGGCTGTCCGCGCCTCCCCGCCTTCTC
GCGGCTGCCCGGCCGAAACCAAACCGAGGGGTGGGGTGGCGAGGACAGGGTACGTCG
CAG
>DHS5
GCTCTAGGCCCTCTGGGATTGGCTGTGCCTGAGCACATCTTCCCTCAGCGTTTCTGA
TTGGTCGCTGGACGCGCGGGATGCGAGTCCCCATTGGCTAGGCCAGGGCGCCTGCGC
GGCGGGGTTCTCGGTCGCCAGCCATTCCTGAGGAGGACTGCCGGTCGTTCGGACGTC
TTGCCTGTCGCTGGAGGAGAGGTCCGGGCTCTCCAGGAAGGTGGCTGCGGCGACAAA
ATGAAGATATTCGTGGGC
>DHS6
GAGCGAGGCCTCCAGAACTACCATCGCTCTGTCTTCAGCGACTTCCCGGTAGCCGCC
GGAAAACTTCCGCCGAGACCGGACGTGACGCTACGACGGCAGCAGGAGCTCCTGGTC
CACAGGCTCACAGGCCCGGCAGCGGCCCCCAGAGGCCGGGCTGGGAAAAGCAGCGGG
CTTCAGCGGATGAACAAGGTTACCACGGGAAGTGTAGGAGCCAAGGCCG
>DHS7
AATTGGAGCTGGAGTTCTGAGCGGCTGGGTACTGTGTATTCCAGCGGTGGGTGTCTG
TACGTAGTTAGAACCTGGCAAGAGAGCACTCTCGAGGAGCCCGCAGGCGGGGCTCGG
CCGGGGACCAGCAGGGCTTGTCGGCGCCCAGGCTCTGCCCCCTACCCGACCCTGCCT

GCCGCTTGCCGCCTGCCTTTGGCTAGGGCCGCCGAGCGCCCTGCCCATTTAAGTAGG
CGGGCCGTACCACCGCAACTTTCTTGA

>DHS8
CATGTATGACATCCCAAAGATGCCTGATTATTTTCCCTTCTTTAGGGAAAGGTGGTC
CCAATCACCAACAAAACAGAGAAAGAGAACGAGAATTATCTCTAGTGGTTTTGCCTG
CTAATCTTCTTTTTCCCTGAAAGAACACCCTCACGGCACGGCGTTTTCCTTAACGGT
CAGGCGTTTTCCCGGGCAGCTTACCTGCTCGGCCTGGGTCTTTCTGGACAGCAGGC
>DHS9
TTACTATCTCCATGAGCAGGTCCAGGCGCGGGACCTGTTCTATGACTCGATTGGGTC
CTGCCCTTGTCCGTCATCGGAAGGCTTACAGCGCCTACTGTTTTGCAGTAAGATTTG
CAGGCTTAGTACCGCCTCTGGAAACAAGGAGAGTGAAGGGGCGTTGACGAGTAACGT
TGTTTGTATTTCTCTCCTCATTGGATGGCTGCTGTGTCAGTCAGACACTTCTCTGCT
TCC
>DHS10
CTCCATCGCTATCCCAGGAAAAAGCTCGGGCCCAGCAGGCGCGTCACAGGGTGGAGG
GGGCGGGACTGCACCATTTGCGCAGGCGCCTCCAATAGTTGGCCAATGGAAAAGCTG
TGGCTCTAGAAAGCCAGCCCGCGCTCCGTTCTGACGTCACTATAAATGCTAGACTTT
ATTGGAAATGTAGTCCTTGTCCTGCCCCGCGCTATTCATGCTTGTCAATGCTGTG
>DHS11
TCTCACGCACTCACTGTCACTATCGGCGCCGCAGCCGCCGCGGCTGTCTAGACCCAC
CCAAGGCCAACCGAGCTCCTGGGCTGAGGAAGCAGGAATGGGAACGAGACGAGTACG
CCTGCGCCGGGTCTGAGCGTCAGACACTGCGCCTGCGCAAGTGGGCCGAGCGCAGAC
ATTGCGCCTGCGCAGCAATGCCATCGGTTAAAGCGCATGCGCAAGATGAGCTATTGC
GGAAGT
>DHS12
CCCCATCCCACCTCTTACCTCCCACCGAGCCCAGCGGGTGGGCAGGAGGGGCCTGAT
TTGGGGACCAGGAAGGAGGAAGGAAGGGTGGGGGCGGGGGACGGGGAAAGTGACTGG
AGCAGTGAGAGCCGGAAATGAGTGGCTAGCGAGGCACCCAGGAGCGCCCTGTCCGGC
GGCGCTCTAGCCCTCGCGCACTGTCTCTATGCTCCCCAGCGTCTAGTCTATTTATTG
TCGCGGGG
>DHS13
CAGGCTGAAAGCCAGGATAGGGGCGTCTGCAGGGGCCAGTGTCTGGATCTGCTCACA
TGACAGTCTTCTGTTTGGCCTGGTTTGGGGCGAGGGGCAACCTCTTTCCCAGGATGC
CAGCCATGTGCAGGGCATACTGAGGATTCAACAGTGGAAACCAAAGTCCATCAGGTG
TTCACCTTCTAGTGGGGCAGACGGGCAAGAAGCAGAATTGGTGGGGGCAGGCAGGGG
TTGGGGTACAGTTTTGACCAA
>DHS14
CCTAAGAAAGCGTGTTTGACTTGATCGCTGGAGCATCTGCCGGCACCGCCCAGGACC
CGCCCAGGACCCGCCCCGCCCCCTGCGGCCCCGCCCCCCGCATGCTGAATGCCATAT
AGGCGAGTGACGTCAGGCCGTTTGTTGTCATTGGCGGCTCCCAAGATGGCGTCCATC
ATGGAAGGGCCGCTGAGCAAATGGACTAACGTGATGAAGGGCTGGCAGTACCGTTGG
TTCGTGCTGGACTACAATGCAGGACTGCTCTCCTAC
>DHS15

AGTTACTGGGACTTGGAGCAAGGGCCTATTTCAGCGTAGTTTCCGCTTCTTGTCACA
TGACGGGAGTCAGTGGGCCGCGCCTTCCCACTGCGACTCCCCGTGGGTCCGCAAACC
GACACGTCACTCCTCGGCCCTCAACCAAACTGCTTGGGGCACGTTCTTTCCGCGCTC
TTGACTCCTACGCGCCGGCCTATGACAGCTCGGCGGGAGCATACAGACCTGCCTCTA
GACTG
>DHS16
AGTCCTCCGCCACTCACTACGCGCCTCTAGATCCCTGCGGCCGAAATATCGCGAGAC
TTCGCTCGGGGCAGCTGCTACTGTGGTAAAGAATTCACTTTTTTACCACAGCGTGCG
TATCAGCAAGTGAACATAAAAACGCCTCTTCTAGTCAGAAACTGGCCAATCCCGACC
CCCGGTCCACCTAGGGCTCCGCCCACCACCGAGAATAAGCCAATCTCTTTGAGGCTT
CTACAGGAGAACGCCGTGCGTTTGAGCTTGAACCTGATAAGCCTG
>DHS17
TGAGGTCATTTCTGACCAACACAATTTGGTCGTTCTATTAGTTGTTCCTGCAGACCT
TACAAAGGAGTATGTTGCTGTTCTTATATGCCCTTGTCTTGTGTTGTTCAAAAATCC
TCTGTGCTATGCTTTGGATGCTGCATGAGCCTGCAAATGAATGCTTAGCATTGTTGA
AGTTGACTGCTGCCCTTTAGCTGTCTGGCTTTGGGTTCAGCCAGAAAGAGTGGCATC
TTTAACCTGAGTC
>DHS18
GTTGCACAACGTGAGCGATGCCGCAGCGGCTGAGCCGCCATCTTCCCGCAGCCGGCG
CCTTCCTTTCGAATACACGCCAGGCCCCGCCCCAAGCACAGCTCCTGCGTCCTCATT
GGACATCATGGGCAATCCAAGCACTGGAGTCCGAGTCCGCGCACTCGTTACCTGAAC
AGGCGTTACAGGCCCTTTGGCGCCTGCGTATTCGTGAAGTGTGAAAAAAGCGCGCCT
CTGTTGGGACGGGAAATC
>DHS19
GTGATCCCTTTTGGGAAATGTAGTCCTTAAGGGTATCGCGCCTGCTTTTTCTATACG
CTCTGACGCAGAGATAGCCACGGGAAGTACGTCACCAAATTTCGCCGGTTCCCACAA
GCTAGGTCGTGTGACGCTGCATGACGGGAATTGTAGTCCTCAAATTGCTAGCTTCCG
ATGCCATGCGCTGCGACTGGAACGCCAATTCCCACCATGCCGCGCGCTCACAGCTCT
CAGGGGCTGTCCTGTG
>DHS20
AGAGAAAACTACACCTCCCAGGAAGCCGTGTGCGTCGCCCCGCCTTCTACTGCCTTT
CACTGCGCGTGCGTCGTGCTGTGGGATTCGCGGACCCCTCTGGCCGCGCTGTGGCTG
TTTCACCGCTCCCCGGCTGGGACTTGGAGTCCCTTATCTTTCCCTGGTTTGGTACAT
GACCCGGAAGCCTCGGCTGGCCGTTGCGTCATCCATGGCGCCGGCGCAGAGCGAGAA
GGAGGTGGGA
>DHS21
GTTTTCCAGCGTACGCACAAGTTCCGCCCTAAGGGACCGCCCCTTTAAGCGCGGGCA
CTGCGGTGGAAGCGCGCGCTGGTCTGGACATGACGTAAACCAATGGGCAGGCCCGGA
AGACAGAGGCGTTTGCTTCCGTGGCCAAAGGCGCTTCATTTCCGGGTGAAACTGGCA
TTGAGGGTACTGGGGCGTGCGTGAGGCGTTTACTGATGCTTCCTGGTCCGGTGGCCT
CGGTCCCGGTAAGCCAGGCATGAAGATCACA
>DHS22
CGCACTGAGGGACTACGTTAGCAGACGCGTCAAAAGTACCAGAAGCTGGCCCCCGGC
GCATGCGCAGAGATGTTCCGTGGTGGGCGCGGCTCTTCTCTTGCCCAACCACCGCCC

TGCCTCCTCCCTCCCCAAGCCGCGCCCTGTGCCTCGAGCGCTTGCCTCCAGGGTGCG
CATGCGCGATGGGGCTCCAGGGGGAGGTGTTTGATACATCAGGGTGTCCCGTTG
>DHS23
TAGCCCTGGTCCATGTCTTCGACGCGGCCCACCAGCAGCCCCGTTTACTAGGCGACC
ACAGCACGCATGCGTTCAGCGCACCTCCGCCATCGACTACTGGAAGACCAGGCGCAC
ACGTCACTACTTCCGCGCGCCCCCATTGGCTGGTCTTCACGAGGGCCCGCCCTCGCC
GTCCCAGCTGGCCTTTCGGGCTGCTACGATCCAGCTCCGTGGCCTAATGGGAGTTGT
GGTCCCAC
>DHS24
TAGGGAAGCTCGGCTACTGATCTATAGCATTAGACCCACAACTCCGGTGGCAAGCAT
CTGATCACACTTACCAAGGATCACACTTACCAAGGAGAAGAAGCGACCACTGCGAAT
CTGTCTCCAAAAAGGAGAGAGAAAACGCGAAGCATCCTTTTTAAACCTTACGAAGTG
CCCGGAAGTCCTGGAATGTCTATTGCGCGTGCGCAGAAGCATGCGCAGTTAGAGAAT
CAGAAACTCACTGAGGCTTC
>DHS25
CTGCTGAACTCAGCGTATCCAGTCCCGCAGCTGACCAATCGGAGCTCGCCCTTCCGG
GGCCCGCCCCATTCCAGGAATCGACCTCCGCACCGGCCTCGAAAATTGACCAATCGG
TTGTGTAGCTCCGCTCTTAGTCCCGCCCCCACGCTGAAACTGACACTTCGGATCGCC
GAGTAACGCTCACCAGACGTCCCGGCCCTGCCCCTCACCTGACCAATCAAGACGGC
>DHS26
CGGCAGTCGGTGTAAACAAGGCCTCGCGCCGCTGCGGGTCCTGCGACCGCTCCTGGC
TGGTGGGTGGTCTCGCGTGGGGCGGTTACCGCCGGCTTCAGTGGGAGGTGCTTCTCG
GCTTCCTCCCCCTCATGGCGTACACACCCCCGGCGCACCACGTGGGCGTGAGGCGAG
GAAGGAGGGGTGTTAGGCCAAATTCTATTTTCATTGGCTGTCACTGCTGCCGGCCTT
TGTAAGGGGGCGCTCTGATTGGTCGATAAGGTG
>DHS27
GTCTTCTGGTCCTCCACCCTTCCCGAAAGGCCAAGCGGCGGAGCTGGGGCGCCAACG
AGAAGGGGGTGAGCGTGATTGGGCTGTCCTACATCACGTGACGGCCAGTACGCGTGG
GATTTGGCTCCGAGGAGGCGGAAGTGCAGCACAGAAAGGGGGTCCGTGGGGGACGGT
AGAAGCCTGGAGGAGGAGCTTGAGTCCAGCCACTGTCTGGGTACTGCCAGCCATCGG
GCCCAGGTCTCTGGGGTTGTCTTACC
>DHS28
CTCCCACCCACAGGTTCAAGCCTCCTCAGTATCTGAGAAAGGCGCGAAGCCTCTACG
CAGTTGCGACCCGAGGCGAGCAACAACTAGCGCATGCGCACGTGAGCCCGCGGCGCA
CGCGCGCTGACCTGTGGAAGGAGATGCCGCGGTTGCGCGTCTCGCGCGTGTCCCGTG
TGCAGCAGCCGGCGGCGGAGCAGTGACGCGGCATCTGGGAAAGAGGCGGGAGTTAAG
>DHS29
GAGCCTAGGAAGATGTCTGTTGCCTTGGTTCCCGGGACCTGCCGCTGCGGAACAGCC
CAAATCTGAGGAAACCTTGGACACAGTTCTGGGTACTTCTGCAAAACCCTTCCCCGG
ATCTAGGCGGTCGTCACCGACAGGAAGCGATGTGTGCATCAATATAAATAAGCCGGT
GGGGCAACTAGATTGCGTGCATCCTTCCAGTTCCGAGTGAGGCTCCTCAAGTCTAAG
CAAGAGACCA
>DHS30
CATCTACAAACGCAGGAAACCACGACTGGAGAAGTTATATGATTGGCTAAACCATGA

ATGACGCAACATAACCCACGCCCACTGAGAATCGTCCTTTCCCAGACTGGCTCAGAT
TCCGCTTTCCGATTGGTCCCTACAGCAAGAGGGCAAGGACAATTGCTTAAGTTGACC
TCTGGGTCCGGAATCGCGGGCAAAGATGGCGGCGGCCAGGTGTTGGAGGCCTTTGCT
AC
>DHS31
CTCGCTAGTGAGGAGAGACTGACCCAATCTCAAAAATACAGAATAGGCATGGGCAGG
GAAAGTTGGTGTGTGAGCTGAGGGTGGAACCAGTCAGGCCAGAGTTCCAAATTGGAA
AAAAACATTAGAGTGTGGGAGGGACTAGAAAGAAGGAATTAAAGAACTCTTTTTAAA
TTTCTTGTTTGTTTCAACTGCCTCCCATCCCAACCCCCTTGCTTTGTTTCATTCATG
GTTAACCCACAG
>DHS32
CTTTTCATGTTTGCTGGGTTTGCCCGTTACACCCCTTCGCCCGCACTTATCTAGACA
GGCAGCTCTCGGCCACCCTCCGGGGTCCTGATTTTGAAAAGAGGAGTGGACCAATCA
GATGTGGAGCGCTGTTTCGCGCTGCCATTTGAGCCTGGGCTGAAACTGCGGGTGTGA
CCCCCCCGTGGTGGCTCTGGGTGTCTGCGGAGGAGCTGGGGGCGGAAGCATGAGGCT
AACGGCTTGGCTTCAGTGAAC
>DHS33
CGGCCATGCCAGTAGAAGACTAGATGCCGGGACTTCACCCCACCCTTGAAGAATGAG
TGGCCGGAGAAGTGATGATTGGCGTGCTTAAAACCCAATCATAAACGGAGGAGGGGG
GATGGTCTAGCGGGGGCGCGTTAATGGGTACCTCTGCCAGTCCCAGACACATCCGGG
TCTTCGACAGCCCATTAAGTGTCGGGGGTCAAAAAATCGCTTCAGGCTGCAACT
>DHS34
AAACCTTCAGGAAGGCATGAATGTCGCCACATTGCCCTTTTGGAGGGAGGCCCTGCC
TCCGAGAAGAGGCGTAGGGCCCAACGTATAGGGCCGCCACTCACTGTAGGTGCGGCC
CAGGGCTTGGAAGAGCAGATCACGCTTCACGCTGACAGTCGGCATGGTGTGTCGAAC
TCACTGCGCCTGCGCAGCGAGCTGACCCGGGCTTCCCTAGCGAAGCGCGTAGGACCC
GCCTCTTCTTGTGCGTGTTGTCTTGAAT
>DHS35
GCCAATAAACAAACAGCAGGAAGAGGCGGGGCCCTGTAGTAACCGCCCCAAACACCT
ACTTCTAACCAGTCGAGTCACAAGACTGGGGGGAAGGGCGGAGCCAATCCGACGTAA
CCCAATCCAAGATTTGAGGGCGGTTACATGCACCCACTCCCTCGGGTTTGGCTCCGC
CCCTCACCTTGACGTAGCCCTCCCAGCCTATCAACGACTGCGCCCGTTGGGGGTGAG
GGGAAGGGGGCGGAGCGTGGGAGGAGGCCGAGAGAGGAAG
>DHS36
GTGGGGTGGGTAACAGGAATAGGCGGGCAAGGCTGCGGGTGATGGTTTCGCCCGCTG
CTAGTGGTGGTGCGCCTGCGCAGAGCCGGAAGCCCTTTGGTAGGCGGGACCCGACCG
AGTGGTGCCGGGATTCCGTCTTAACCCCCGCTAAGGTGTCCAATGACCTTTCCTTAC
CAACTGCGGGGAGTGTGTGGAAACGCGGTTCACTCCCCGGTTTCCTTATTCTGGATC
AGTG
>DHS37
GGGTAACGTTGTCTCGGATCTCCCGCCCCCTCCCTTCTTTCGCTGGCCGCGCACGCG
TGCAACGCCCCGCCCACCTCGCCCCACACTTCCCTGCTCTCACTCCAAGCCTCCGCT
AGGTTAAGTAGACGAGCCGGGCGGGTCTGAAGAAAAGATGCTACGACGGCCGCGCGG
ACGCTCATGGGCGGGGCGAGACCACAGCGCGTGCGCGGGCTCCTGAGCGCCACGCGC

TCGCCTTTCGTTCGTAACCCCTG
>DHS38
TAAATGCTACTACAGTGCCCCACACGGACTGCCACGAGCGGGTCACGTGACTGGACT
CTGGATCGGCTGTGCTGTGATGGCCGCGTAGCGCGCTGCTGTGCGCGCGAGACTATA
AGCCTCTGGGCCGCCGGTGCCTCCGCGCGGTCACCGCCTCCACCCGCCTGGCCGCTT
CCCTCGGCGCATGCGTAGCACACCGACTCCAGGATTGTTTCCCAAATCCCACTTC
>DHS39
CCGGTCGATGTGCTTCAATCGAAGCTGCCAACCTCTTGCACCTTCCTTAAGGAGGCT
CCGCCGCAGCCCCGCACGACGCGCCGGTCGCCCAGCCGCTCAGAGCCGAAATGCTCC
GCACCGCCTCCGCACGGGCTCTAACTCTACCGGATGTGACGCGGCGCTCCTTAGCCA
GGCTCCTCCCCTCCCAGAAACGCGCGCTTTCTTGGCCTCGCCCCTCCTCGCTCTGCC
TTTCCT
>DHS40
ATCGTGCAACCATGGAAACCAGCCCGCCGGCTGCCACAGGGCTGCCGACTCACCAGC
CTCGCGCGCTGCCAGGGCAACCGTGAGACGGGACTTCCGGCTCCCGGGGAAGTCCCG
CCCCTCTCCGCCGGCCGCTTTTGTAGCCGTGGGAGGCGGGGCTTCAGCCCAGGGCGG
GGTCCAGCACGGCGGGGCGGGGCGGGACGGGGCGGAGCCTTCCCGAGCTGCGAGGTC
GCTGTCTCCTTGCGCTCGCCACTGGCTCTCTCTGCGGCAGGTTTC
>DHS41
GGTTGGATTGTGGAATTACGTCTTTTAAAATGTTATTTGTCCAATGAACAAAAACCA
CTCGCACCGCAACTCCTTTTACTATCAAATTCTGCACCGTTGCAAAACTTCCTTCAC
TCAACGCTTCTTTACAAACATCGCGATAGCGGAGCGGTCTCTTGGGCGCACCCCGCC
GCATGTTCAACCCCACCCACTTCCTGTCTGACGATCTCCTTACGTCAACGGTGCGCT
GGAGCGAGTGAGCAGCGATACCTAGG
>DHS42
GACGTAATAACGATCCCACGTACACTTTAAAAAGCAAACTTCTCACATACATCACTT
CCTGGAAGCCCTAGTCAGCCTTTAGCCCAAGCAATGCTGAGCCCAATCCTTCGGCCT
TCCGTAGTCTCGCTCGGTTTCGCGAGATCGAGGCTGAGCTCCGCCGCGCCGCCGTGG
GGGCGACCCCAGAACACTTCCGGGTGTAACTGGTTGTGCTGTCTGTGTACTTCCGGC
AGCCTCCAGACAGTTTCTTCCGCTTCCTGTAC
>DHS43
GTCTCTTCTGCGGATATACCTTGGCTCCTCCCAAGTCCTACGAGTTTAAACTTCGAC
GCACCAAACATGGCTTCTTCGCGGTTCTGCTCCTTGTTTTATTTCGCTCCCACCTTT
CGGCCACACTCAACATGGCCCTAACCGAAGGTTGGCATGCCTGTGGCACTGTCTCGG
GACAGTTCTCGCGAGGTTTGCCTGGGCCCGACTGCTCCCGCCCTGCCTCGCGCCGCC
GCCACAGCCGCTGCCGCTGGCGCCGCTCCTCCTCCGTGTCAGTTG
>DHS44
CCTAAACAACAAAACTCTGTTGCAGGCTCCTTACTACGCCGAAGCGCCACGGCTAGA
GCCACTTCCGGCGCGACCTCCTCACCCGAGACGACCTCAGCCCAGCGAGTACTGCAA
CCTCCTGCAAACACCCGCAGCGCCCACCACAGGCTAAACCCCGCCCCGTGCCGCCCC
CGAGCCTCGCGTGCTCGCGCCGGCCCAGTAACCGCGAGACAAGTCAGGGTCCTGGAC
>DHS45
CGGTCCTTGCTCAGCACTACCCCCTTACCTGCCCTGCGCACCCCCCGCGAGCCCCGG
GGCCCTTAGCTGACGACCGCCCTCACCTCTTCTCTCTTCGCCTGCCGCGGCCTTTTA

CAAACGGGGCCAAACTGTTCGCTGTAACGACAATCGTTATTGGTCAGAGCCGCCCAC
GCGCGGCAAGTGGCCGCCCACCTGAGCTCGCGTTGTCTTATGGGAGTTTTAGTTCTC
TCCTTCAGAGACCGCT
>DHS46
CATCCCATTGAGCTACACTTCACTTTTTAGGACAGAGCCTGGTAGATAGGAGACAAT
CAATACAGATTTGTGTTTTAGGTTATTGCATGCTTATGGGTTAGCTAGGGAGCTTTC
TTTTTATCTTTCAGGAAAGAAACCAAAGAGACAGACTCTCCAGCAGGCAGGGGGCGC
CAGTGAGTGAGCTAGGCATAGAGTCTTGTGGAGAGAATTATGGTGTCATAGAGGCTT
AGAATCTTGGCATTAGCAGGG
>DHS47
ACCTTGGCTCCATCCCTGAGGGCCAGGGGAACCGGCAGCCTCGAGACTGATCCCTCG
CCCTGGAGATTTTTCCAATCAGGCAACTCTGAGGACAACTGCGACCCAGAGCACCAC
AGGGTGTCCTAGAGCCACCTGGGCGTAGAATAATGACGTATAAGGATGTGGAGGCCA
CCAATTGGCTGCGCTGTGCGAAGACGGACAGGTAGGCGGGGCGGGAGGAGGTGTGGT
CGGCAGAGCAAATCCAATAGTCTTTTC

>DHS48
AAACAACCGCGAGCTAAGAGATGGGGCAGCGGGAGAAATGAATTCGAGTTCCGCCTC
CTACCAGGAAGAACCGGCTCGGGCCGAGGGCTGCACGGAGGACCACACGGACGCCTG
CGGGCCCGCCCCTTCCGCTTCACGACGTTCAGCCTGCGTCTGGAACTGGAATGGCCT
AGCCCAAAGCTAGATAACAGGTAGATTGTTTTTCCCGACAAATTATCAAACGACCC
>DHS49
ATATCTGCAGAGCAGCCTTGGCGTTGACCTCTTGTGGCAATTGCCTTTGACAGCCTG
AGAGTCATTCAGAAATTGCCTATGGTTCACTCGGGGGAGGTGGGTGGTTCCTCTCTT
GCAAGCTTATCTTCACTTTGCAGCCCTGGTAAGTAACTGCTGAGCGTTGCACAATCA
GGGAGAAAATATTTACAATGAAAGTTGATCCAGAAAGACTTAGACAAGGGTCAGTGT
ACCTTCAGTTTAGCCTGACCTCTTC
>DHS50
GTCAGAAGCGGCCAATCAGAACCCGTCAGTGGGGGTGGAGCTCCGGCGCCGGAGTCT
GACGAGAGAAGGGAGGGGAACAAGGATAGAGCGCTCGCCAACGGCCGACGCTCCAGG
CCGTCCCGCCCAGCGCAGCTGCACCCAATCACAATGCTCAGATCGGGAGGTGGAGCC
AATCAGGTCCAACCAAGAGGAGGGGACACCGGCACTCCACTAGCAGGAAAACGG
>DHS51
CTCGAGCAGATCTTTCCGCTACTCGGGAGTAAAATCTTCCCGCCAGCCAGCTGAGAG
CATCCACCCTTCCGGGTCGGTCCGTTCAGCCAATCACAGATAACTCCCTTCTCTAAT
TGGTCAAGGGGTGTGGTCACCATGCGCGTCACCGAACAACATTACGAGTTATTGGTT
GAGATTGGAAGCAGGTTTCGCCAATAGGAAGCGCTCTTAAAGCGGCAGTGAGGGT
>DHS52
GTTGGCTGTAGTAACGTGGCAGAGGCAGCAGCGGAACCCCCCACGCGGCAGCGATTC
GACATCCGAATCCACCGCCGCCATAGCGACTCTCCTGTACCGACAAAAACACAAGCA
CCTCTGCCACCACCGGAACCCACTTCGCCAAAGCTCAACGCGCTCGGAAGCAACTTC
CGCCGATCCGATAGGAAGTGACATAAAGTCGTTCAATTGCGCGGACACACTCCTCCG
GAAACCAAGCTCCTTCGACTCTG
>DHS53

AGGTGGACCTCCTCAATCTCCTGCTTCCTCAGTAAGAGTTTTCCTCTGGACAGTACT
TAGGCATTTTCTCAGGCTCCCCGCCAGAGAGCGCCGCCAGGTCCTCGGAACCACCTC
GCGCACTTTAGCTGGGCGCGCGACCCGCGCAACCAGTGAGAACCCCGCTCCCGCCCT
AGGCTTTCTGTGGTGGAAAAAGCGCGCGCGCGGACGAGCAGGTTAGGCGATAGAGGT
CACGT
>DHS54
GCCCCTAATTTGTCCTTTCACCGCCAGCCTAAGCCAATAGTGTGGCTGCATGTAAAT
GAGAAACCGCGAGCATCTCCCCCCGCCCCCGGCTCCGCCCAATTTCCCACCTCTTCC
CGGAGCGCACAGACGCCAGCTGAGTGGGAGGGGCCTAGCTGCGGGCGGAGCACGGAG
GAGGAAGGGCACGAGGAAGAGGCGGAGCGGGCCGAGGCGCGCGGAGCTGGGTTTGCT
GAGTGCTTCCTTCCT
>DHS55
AGAGGACAACGCCAAGACGGCTGGGAAGCCCCAGGCCAGCAGCGGAAGTCCACTCGG
CCCGCCCTGCGACTTTGAAACCGGTGCCCCGGACCACTCCTTGGAGACGGTGGCGCC
TCTTGCGTCATCACGCGGGCGTTGGAGCTTACGCCATCGCGCGTCCTAGAGTCCTAG
CACGCTATTGGCTGGCTGTCAAGCAATGGGAGGAGTCGTGAGAACCTGGATGAC
>DHS56
GATCTTAGGAGAGCAGGAAATGCTCGTAAAGGTTACCTAGGGGGTGCAGGGCCGCCC
TCCCTGGCCATGCAATCCCCCGATTTCTCCCTCACCTTAGCCAAAGCTCGACTGTCA
TTGAGCCTCCTGCGCATGTGCAGGAGGGCGTCCTGGGCTCCCGAGGTTCGGCTTCAC
CCAGCAGTAAAACGCATGCGCAAAGCTTCGGTCAATGCGGGCTGTCCATTTCTCACC
GCCGACTCCCTACATCAACTACGCATG

>DHS57
ATACATGGCCCTTTCTGATACTCCGACTTCTTTCGGCCCACGTTCACAAAGTTTTCC
GTCGAATACCTGAAACCTTTCGACCGCTTCGACCCACTTACCTCATGGCGCCTTAAA
ACACGAACAATGGAGGCCGCACCAACCGCCCCTTCCTCTGAGTAGCAGACAACTGAA
GAGGCGTCTTGCCGGCGTGTCCCAATAAAACCCGGTGCGATTGGCTCCCGCCTTACC
CCATCGCTTATGCCTATTGGCTTAC
>DHS58
GTTCAAAGTCCTAGGTCCAGAAGCTGCAGCCCCACCCGTGACGCTTAAAGGTGGCGC
CGTTGGGATGCCCCGCTCCTTTCGTGAGCGCGCGCTCTGGGCGCTGGGCTCGCGGCC
TCGCGCTGGGCCTTCACTCTCTGCGGCGCAGTCCAGATGTCGTCAGCACCAGCGCCT
GGGCTGGAGGACAGAGAAGCCTTTTCCGTTGCCGGTGCCGGCCTAGCGTCCTGGAAT
TACTTCAATCAAGTAAGTGCGTTTAACGG
>DHS59
GGGGCTTTTGGGAAAATTACATAATCCCCGCCCCTGGGCCGGAACTGCCCTCGCCTA
TCCCAATCCTCCTACCTAGGATTCCGTCAAGTTGACGTCACACGGAACCGGTGCTCC
GGAAGCTAAAGGTGGCCATGCTGTGTGGGACCACGTGGCCAGAGGCAAGAGTGTCGC
TCTAGTAGTATATGCAACAGTTTAAACTATAGGCATGTTCATGTAAATATGGTTTCT
GTCCAACCCG
>DHS60
TAGCATGAAAAGAGGCTACTGGTTTAAAAAAAAAAAAAAGCACTCTCCAAGAGACCA
TGGAACTTATCTTATTCACCACTCCATTCCTAGTGCTGAGGGTGGGGCTTAGATACA

ATATCTCAAGACATACTTGCTAAAATGTTTTAAAAGGCAGCTTATAGAAAAATGTGA
TTCCCCAAAGAAGGTTCCAGTGATCACGTAAGTTTAGGGTATCCATTTTTTTTAAAC
ACATCTGTACGTTTCTGTTGCTCCTTCTAGCC
>DHS61
CAGAAAGCTCAGCGTGGAGTCGGGGGACGCTCCTCCTCGCCAGGGTTCCCGTCCCCT
TTCCCTGCTGGGTAAATCGCATTCTGTCTCTTTAAGGAGTGTTTGGCCGCGACGAGT
TGGAAAGCCCGGATGCGTCCTTCGGTTGGGCGGGGTGTCTCAGTGACGTCACTGGGG
GTATAAAAGGGCCTGGGTGGCGGGCGCCTGGGCAGAGCGTCCTAGCAGTGTCACTGC
GTGGGTTGGTTTGTGTAGAGAGGCGTG

>DHS62
GCGGTAACGGTGAGGCTTTTTCACGCCGCCGGTAGCCGGCGCGCTCTTGCGAGCAGC
CTTGGTAGCCAGCTGCTTGCGTGGCGCTTTACCGCCGGTGGATTTCCGAGCTGTCTG
TTTAGTACGAGCCATGGCAAAACCACAGAAAAGCTTGCCTGCAGAGACGTCTGTGGA
GGAAAGGAAAGAGCTACTCTTCTTTTATAGAGTCAGACCACCAACTATTGGACC
>DHS63
GCTCCATCGCACTAATCAGCCGGGCGTGGTGCGGGCGCCGGGAAGACTGGGCGGGGC
GAAGGCATCACCTTTCGATGGGGCCGGCGCTGTGCGTCCTCCAGTCGAGGTGGGGCG
CTGTGGAAGAGCGGCAGCTTCCGGTCGGCTTTTCTCTTCCTTCGGCTGCCGGCGCCA
GCACGTCCTGTTTTCGTTGGCCGCGCTGGGATGGCCGCCACAGCTGTAGGTGCTGCT
AGTGTTTAGCG
>DHS64
TGGCTTAGAAGTCCCAGTCAATTCAGGGTGACCTTAGCAGACAAAGGCTGGAGCGCC
CCAAAACCCGCAACATTCGCAAAGATAAAACATACCTCACAGGAGTCGACTCCTACC
TCGAAAAGACAGTATGGTGCCTGGGCTCCTGTATATAAGAGTTCTTTTGATGACGTC
ACGACGCACGCTCCCGGCAGCGTCCGCTTCGGCTCCTCCCCCTAACCTCCCTCAATC
TTCCTCTCATCCAGTATCACCTTCTTCCC
>DHS65
CAATGAGAGGAGTCGGTTGGTCGCCCTGTGGCCAGTCACGTAAAGGGGGGGGCGGGG
CGGTCGTGGGGGTGGAACATGCGTATTACTAAGCGGTCGCCGGGCTGATGACATAAA
AACCGGGTGCCGGCAGGCGCCAGTCGCAGGTGTGCTGCTGAGGCGTGAGAATGGCGT
CCCGCGGCCGGCGTCCGGAGCATGGCGGACCCCCAGAGCTGGTAAGTCCCGGGGCCC
GCGGACACCCCTCTCCCCACTTCT
>DHS66
ACCTGGGAAGGGATTCATTTGGGGGGGTCACACCAGCGGGCATCCTGGCTCAGGCGG
AACTCTGGCTCCAGACAGTGCGCCCAGGACCAAACGGTCAACCCCGGCCTACTCCCG
CCCCTCCCAGATCCTCCCCTCCTCGGACGCTCCCGCGTCTTCCTCTCGCTCAGGCAC
TGAGGGGCGGGGAGATGGGTGTTAAAGAGGGGATATCACCAATCAACGTGCGCCTCC
GGGAAACAGCGCTTCGCACTGGCTGGTTGGGTCTGAC
>DHS67
AGGTTTCTCCTTCCCCTCTTTGTCCGGCTCCTCCTGCGCCGCCACCGCCCGTACTAC
GCGCACCGCTCCGACCTGCGCAGCCATTTCACCCACAAACACACATTTAAATGGCCC
GACCGCCCCCCCGTCCGCGCATCGCGCAAGCGCCGCCCGCGCACTGCCCGCTGGGCA
TTGGAGTCTCTCCTCCGCCCTCCACTTGTCGGCCGTTCTTCGTCCAGAGATGAACAT

GC
>DHS68
ACTTCATGGGTACTGGATCAGGGAGAAACCACAGGAAAAGGAAGTTACAGCATTCTA
TCACTGCAGACCTGAGGACATATACACAGAAGAAGAACCAAATATCCACCCAAAATG
CTCTAGGATTCCCATGTTTTGGTTCCCTTCCAAAAATATATCACAATTCAATCAGTC
GTATGACCAACAGCAAGGGTTTCAACAAACTCTTTGAAATCTAGAAAATGCTCTTGA
TACTCCAGG
>DHS69
CTATGGATGGTGTAGCGGTCAGTCATCTCGCCGCTTTCCCCTTCGCTCTCAGGTCAG
AGGACGCAGGTAACAACTCGCCGCTCTAGCGTTTTACAGGAGAGTGAAGCCACCGCG
CGGAAGCTCCAGGTCCGCGCCGTCGCAGAAGAATGGCGTCACAACGACTAAGTAGCG
TAACTCTCGCTCATAGGGCTCAGAGGCGGAAGCGGAAGCACATTCTGTCACGCAATT
GGAGT
>DHS70
TTCTTAAGGAGCAGAGTCTTTCGTCCCTTCGCCCCAAATCGCTCAAGTCATTCCTAG
GAAATCCCGAGAAACACTTCACCTCACCTGTCAAATTGCACAAAATCCACTTTCGAA
TTTCTCCCGCGAAACAACGTGACGTCATTAACGCGGGACCTCACCAGATGAGGACCC
AAAACTTGGCCCGCCCACCTCCGCCTAAGCCAAAAACCGCCGCTTTCAAATCGCCAA
TCAAATTCTTCCTTGGCTTCGAGTCT
>DHS71
GGTTCTGCGACTCGTCTCCTCCGGCTCGATGTTATCCTCCTCGTCCGAGGAGCCTGA
GCTGCTGCTCCCGTATGCCGCAAAATAGCTCAGAGGGTCCTTCTCCTCCGCTGCCAT
GACGGCTGCGAGCGACAACCCAGCACTCCGCCGGAAGCCGGAAACCGGAAACCGGAA
GCCACCCCAGGGCCCGCCCCTCGCGTGGCCCCGCCCCAACTCTTACAAATAAGCCTG
CA
>DHS72
ACGCTGGTCAAACTCACAAGACCGCAGCAGCCGCCTCCAACGGCAAAGCTCTAGGGA
GAGACCATAGAGCCCCGGCGGCGGCGACGGCAGCTAGGGCGGCCCCCGACAGCGCTT
TGCACATGCGCAGAGAGTACTACGTGGTTGCCCTACGACACTTCCGGCCGATGCCTC
CGGGCTGGCGTCCCTGAAGCCCTCGCTGGGAGCCCGAACCCGCCCGGACGCGGTGCA
TGCTGGAACTTGTAGTCTT
>DHS73
GCCAAACCCAAGTCTTACACTCTTAGTGACCCCCTCCTTTCCTCCTTCCCCCTTAGG
TCAGCAGGGTTTACTCATTCCAATTGCAATGCTCCATGTTGCCGGAGTAGCTCCTCG
CTAGAAGAGAGATTAGTTATGTCACCGTCGGCTCCTCCCATTGGTCCGGGCGTGTGG
AGCGCGGCGCCATGACGTCAGACGCCCAAATGAGTGGGGCGGTGAGGGGAAGGAGGA
GGGAAGTAGGACTTCAACA
>DHS74
TCCTTCCTCGGACTCCAACTCCCGCCGTGCCTTGCGCTGAGATCCCTGCGGCAAAGA
ACCGGGCTGTGTCCAAAGTGTTCTCTGGAAGTTGTAGTTCCTGTATTGGTGAGGCAA
GGAGGAGGCGGAGTGACTCGGCGGCCATTAGCTGTGTGTAGTTGCCCGGGACTAGGA
GCTTAAGTGAAGAGGTACGCCTTGTTCGGTGGAAATCAGCCGTAGCCATGAGTTT
>DHS75
CTGCCTTTAGCAAGTGCTGACTGCACTGAGTCCACTGGGACCTTTTCCTTTTGGGTT

TCGAGGCTCCTCCCACTCACCCCCACCCTAGTGCTGGAAGAAGTGAGTCTGCAGCTG
AAGGGTTGCTGGGGAGTCAGTCTTTCCTCTTACTTAGGAGGGGACCAGGCATACACA
CACTGTCACAGGTCAGCCAGGCCAGTCAGGGACCAGTGACTCACCTTCCTCCTCAG
>DHS76
AGGTACAGCCAAGATTGGAAACAAAACTAATAGAGATCCTGATAAATTCAGAATGTT
CTTATTCATTTAATTTGTTAATGCTTAATTCTAACTTGAGGGATTCTGATAAATTTA
CCACTTAACTGAGAGGAATGTTTTTGAAATCAAGTATTTTGATCTAAGGAAAACACT
GGGTAAATTTTCTGTCAAATTGTGAGCTGAGATGGCTAAGACTAAGTATATGCAAGA
AGATAATGCTTGGGTA
>DHS77
GGATAGAAATACGTCTCCGAGTCCTCGTCATATTGGAAGTCCTCGATTTCCACCTCG
TCATGAAACACTGCCATGGTCAGCGGGGGTGGCCGAAGGGGTAACGCCCCAGCAGTC
CGAGGCCAGCTCCGAGGGTTTAACTTCGCCGGAACCGGCCTGGGCTCGGCATCATCA
GGTCGCGCCGGGCAAGGACTAGCCCTTCCGGCGCATAGGCAATGACGCAACTCCGCC
CTGCGCGGCCAAATGGATAACCGGTAGCGGT
>DHS78
GAAGTCCGACCCTTCGATTAAGCCCCGCCTGATTGGGAAAGGCTCTGAGAAACTATT
TGCATGATCTTGAAAGAGCTGTGCTGAATGGTTGAGGTATGTAAGCCGAATGTGAAA
CAGGCTCTCTCATTGGCCTAAATTCCCCCCCTCGATCTGCGTCGCCATCCCGGCCCT
GCTGGACCCCGGGGTCAGTAGGAAGCCGCGGGGTGGTGGCGAGAGAGGACCCAGGTG
TC
>DHS79
GCTCTGCTTCACTTCCATTTCTAAGATTTAGAAGCAAAGACGGAAAGAGAAGCTTGG
CGGGCCCAGCTGGGCTGCGGGAGGAGAGGGGAAGGGGCCCCAGGCTGGAGCAGAAGT
TGCTGGTGCGCCGAGCTGCCTTGCGGTTTAGGCCCAGAGGTTCACGCGAGCGAGCGC
GCTGTGGTGAGCGCGATAACCGGCGAGCCGCGAGGCGATTCGGAGAATTTCTGAAGG
CTCTGT
>DHS80
GGGAAACCCTGGACTCCATCCCGGGATTTGTCCCCGTGTCGAAGATGGTATCCAGGT
CGCCGAAAACTTCCCGGAAGTTTAGACCAAGACCTACTTATTATCTACGGTGTCCGG
GAAGGTCCATGATTGTTCCACATAAAGTCAGAAGGGAGGAAAGGCGCACGCGCATTG
GGGACTTGACTCTGGAAAGCGTCCAGACCAAGGTCAGCGGAGAGTAGGCATGTAGCT
TCTGCA
>DHS81
AGTCCACCCAGTTTCCAGACTTCTGCCCGGTCCTACACTCACCGGCCAAGGCCGTCT
CAGCTGCCGATTTTCTCCAACTGTGTTCTCCCTCTCCCGGCCTAGGTATCTCCATAG
ACAGCTGCTCATGCGCACTTCCAGCCCGCGGCCATGTTGGTGAGGGGCGGAAGTGTC
GCTCTCGCCCCGCCCATAAACTCAAACTCAGAACTGCCCCACTTCCGCTCACTCCAT
CTT
>DHS82
TAAAGGGCACAGTGAAAAGGAAGTTGGTATCAACGTGATCTCGACTAAGGGACCGAG
CGGAAGTCCTCAATCGTACGCCACGCGGAGAGCATGCTGGGGGCCGGAAGGAAGAGG
AAATTCCAGTAGCCGATCAGGAGTCTGCAAACTCCGGTGGTAGGGGAGCGCGCTGCT
GTTTAGAGCCACGAGTTACCGGAGCGCCTGATTCCTGCGCCGAAGTCAGTGGTGGCC

GAAAGTCCGGAGTCGCTGTAAAACCTGAGATT
>DHS83
GGCGACATTGTCCTTGTTCCGGACGTGCCAGCTCTTCTTGGGCAAGATATTCATGGC
GTCGTAGCTGTCCAGGGACTGGCACGCCCGCCTCTTTGCACTTCCGATTGGCGAGAG
GATGCCCCCCTTTTTCTTGTCCCTACTTCGACCGCGGATTGGTTCCGAATTAGTTGG
TACGGCCCCCTGGCCTGTAGCGACAGGTGATTGGCTGAGACGCCCTTTATCACAGCG
AATGCTAGG
>DHS84
CCATCTGCTCCTTCTGATAAGAGACTCGTCTGTCTGCTAAGAAGGTAGGGAAAAGGC
TCACTAAGAAAGAATAATGCAACTCTGCAACCTCTACAGCCTCGCTGGGTGGCTCAT
GGACCTGCACCAACTAATTAGGGTTCAAACCAACATTTTTAGGATGTATTTGGATGG
GATTTGAATTCAGTGTGACTGCGCTCTAAACAGCACTACATTCAGCTACTGGTGTAA
TTTGGACAATGAGC
>DHS85
GGAAGGAGGAAAGTCTAGCTGGTTTCGGCTTCAGGAGCCTCAGAGCGAGCGGGCGAA
CGTCGCGACGACCGGCTGAGACCTAGAAAGACAACGACCACTCTGCTACGCCTGCAA
CCGTTTAATGCCGCCGCGTCCGCAAGCGCGCGCTCTCACCCTGCGCCGCGTTGCGAC
GTCACCACGCTGTCCAGCCCACGGCCTTGCGGGGAAGACTTTAGGGCCAATCGTGT
>DHS86
CCGCCCTACTCTATTCGCATCCGCCCGCCCGTCCCCAAGACCCCAAGTCCCGCTTGC
AGCGCCCTTAAACCGGCCACAACAGCTCACCCATCCTGTCGGCGCCACGGGCCTGAG
CGAAAGAGAGAAGCACCGCAGGAAGGAGCCACAAGCCACCGCGAGCAAGCCCCGCCC
AGCCAAGGACCCCGCGCCGCTGTCTGCGCCGCGCCTGACCCGAGGCTGGGGTCTCTC
CTTTGAAAACGACG
>DHS87
CTCAAGAAGATGGGGCTTCGAGGCTGAGGGAGCTCGGCCCCGAAAAGACGCCTGCGG
GAGATGGCACACCTAGCTAGGACCCCATACTGGGCAGGCGATCCTTCCGAGCATGAG
CACCTGGCGCTAGCGAACCACAATTCCCAGCACGCCAGGCGCCCGTTTCCGGGGCGC
GCCGCGGAACCCGGAAGTGGTCACGGAACTCGGCTGCGGCTCCATGGTCTGAGTTGT
CAGCCGTTGTTT
>DHS88
CTTCAGAAAATCCCTGCCACCTCGATAGGACGTCCTCTTCTTGAGCTCCCCTCTCCG
GCCCTCCCTGATACAGCAAGGTTGAGCATTCGGGCACTGGCTTCCCGAACTCACTTC
GACTCAGGCACAGCAGGCAAAGATTCAATGGGTAGGGCCGGACCGACTGCCCGACCT
TCGCGGTGCTTCACTTGCCTCACTGCGCATGTGCCAAAGACAATCACCCAAAGTACT
CGAAA
>DHS89
CTTGTTGCTAGGGGAAGCTTTGAGGGGGCGCGGGTGGACGGACAGCAGCGGCGATCT
GCGACTGCGCGCAGGGTGGAAGTTGGGAGGGGCAAAGGAAGCTGCTGCTGGGCGCGG
AAAAGGCGGGAAAATAGACTGCTCCGAGGTTAGCAACCATCTAACCTGGAAAAGGGC
CTCCAAGTGCGAGGAATCTTTGCGCGTCGACGCTGGGAAAACTCAATCGGTCAAATC
TC
>DHS90
AAACAAACCTCAACGCTTAGCCTCCCGGCACCTCCTAGGGCCGGAAGCTTCTCAGCC

CAAAGCCGCTGCTGGCTGCAACCTCCGTCCCGCAGTCCAATTAGCAGCCGCGACCCG
GCGCCCGCCCACGCCGCGTCACGAGTCAGCCAAAGATGGCTGCGCCCAGGTAATTTG
AGCAAAGGCCACAGTGAACTCCGGCGTGGCTGAGGAAGGAGGAGGCACCCACAGGCT
GCTGGGAGGAGAGCATAAG
>DHS91
GAGATATGGGGACAAACAATCCTCGCAAAAACGGTACCCACCGTGGTCGCTGAACTG
CAAGCGAGGACCCACCGCGACTCACCTAGACACGCGACTTCTGCGTGGCTAAGACGA
AATGGCCAGCGGGCGCCTGCGCAACCTAAATAAGGTCCCTTGTGCGAGTTCTGCGCA
CGCGCAGCCAGCTGCCCGCACCGCCCCGTTGCGTCACAAAGAGCTCTGGCGCATG
>DHS92
CTGCCCCTAGAGAACCAGGTGGGAGGTCAGGAAGGTCTGACGGGAGAAGGGGAACTG
AAGGCTGTTAGGAAGGCAGGCGCAAGACAAGGGGCAGGGGAGAGATTAGAAGGAATA
AGACGCGGGAGAGGCAGGCCTAGAAGGAAGCCGGGAGCTTTAAAAATAACCCCGGCA
CTGCCCAGAGCGCGGCTGTTCCTGGCGAGTCACGCTGACTCAGCACATTGCCTCTGC
>DHS93
ATTGCAGGAACAAACGCTTCTTGCTGGGGGGAAGACGGAGACTCTTATACCGCGGGA
GACTAACCTGTGAGCAACAGAAGCACCACGCTACAAAGAGCATGACGAGTTCTTCCA
GGCTTGGGAAAGCACGGGTAAATGCCCGCGGTCCTGCGCGCCAGCGGGGAGCAGTAG
AGACTTCCGGCGTCGGCTGCTCGCAGGGCTAGAGCGTCGAACCGGGACAACCGGAGG
CTTCAGCTCACCTCGGATTGTAAAT
>DHS94
ACTTATCATGGCGGATAAATGGAGGAGTACGAGGCAGCAAACAAGCGCTCCGACGGC
AAAGAGCTGCGCGATGCGGTGGCGGCAGCGCTTCTGGAAACCTCCAACCACGTGGGG
TGAGGGGCGGGGGTTGGTCACTGCGGCCCAGAGGCCCGCTCTTCCGCTGAGGCGCCG
CCCGCGCCGCCTGACCAGAGGATACTCGACTGCCGCTCCCGGAGCCAGGGCGTACAG
GTAATATGCTCCGCAG
>DHS95
GCTACCTCCCAAGAGACCAACACGACAAGCGCGCGCCCACCGCATTGCCATGGCAAT
TCAACCGCCGCTGACACCGGAACCGGAAACTTCATCAGGAGGTGACCCCTTCCCGGC
GTTCCGCGGAACGGGCGAGTCCCGCGCCTCTCCCAGTTGTCTCGGCGTAGTCCAGCT
GGCCTCATTGGCTCACAGGAGGAAAGTCGTCTTCCCTCTTGTTTTGCCTCTCGTTG
>DHS96
AGAGCCACCCAAGTCTGCTCGCTCCTCCTCCTCACTGCCGGCCGCCGCCCAGCTTCC
TGCACTTCGCTGCAGTCACCGCCGCCCCGCCTCTCCTGGGACCATGTAAAGGGCGGA
GAGCCTGGCAGGAGGAGGGGCCTATGGGCCGGGCGGCCCGGGAGGGTGCCCCGCTGC
CCTGCCCCACCAGTGCCATTGGAAGGGCCAGCCTGAGCCTCCGGGACGAGGGTCGGG
GTGAGGAATTCCAGACTTCCCCAGGGT

>DHS97
GAAAGGATAGGTACCATGTCAAAACCGCAAGTTTAAGCAGTTAAAGAAGCTTTAATA
GAATACTATTGCAACAGTTTTATTTTTGTTGAAGTTAGTGACTACTGAGTCGGCCAT
GGCAAGTATGGGCAGCCCTGGAAGTAATTCTCCTTTAATTTCCGAAAGATGTACCCA
CGAGTTTAAAGGCGAGTGACACCATAGCAACCAAGCCCCAATAGGAAACAGCCACAA
GGC

>DHS98
AGAAACATACCTTCCTCCCCATCCCCACCCCTACCGAAAAGAGAGCATCCACGCCTC
AGAGCAAGAGACCAGCAAGGAGACCTGCCATGCCACTCCCAGCCAACAGGGGGCAGA
AGCGCATCAGTTGCATCAGCTCCCTAACGGGTAAAGAGAGCAGCACAGCACGCCTAC
CTTTGACAGCCTTTCGTGGTAATCTGGACTCTCCTGCTTCCCTCGCAGGCTGGGGGG
TACGAACCAGAAACACACATTGACAAACTCAGGCT
>DHS99
TAAACCAAGCCCCACAAAGAAGTCACTTCGGCGATGTAAACGCGACTTTGCGGCTTC
CTGGGCTGCTAAGGTGAAGGACGCCCGCCCTCCCCAGAACTTGAGAGCTTATTGGTT
GGCGATGTCGTCACTCAGAGTGACGTCAGGGGCGAGGAAAGAGGCGGAACCGTAGAG
ACTTGGCTTCGGGCCCTTCTAGCTTGGGGGTCCCGGGAAGGAGCTGGGAGGACCTAG
G
>DHS100
CTCCTCAGTCCCCACACTCCGGGCTTCCCCAGTCGGTCAGCACCCATCACCTGCACC
CTCGGGCCGCGGGCAATTCACGGATTCCGCGCCAAGCTAGGTCCAATCGTCCGAGGG
ACCGGCCCCGCCCAGAAGCCGTTCGCGGCATCCCGCTGTTGCCATGGTGACCAGAGC
GGTGTTTGTACGGCGGCTGCCGAGGCCCAAACTCCCTCTTCCCGCCTCGCGCCTCCC
TCTTCCCGCCTAAGCTTCAACT
>DHS101
CAGGGTTCACAGCTGTTCTGAGCCCCGCCCCCAGGTGGCAGGGCAGCCCAGTCAGTC
AGTCACGTGCTGGCGGCTGGCCAATCATCGGGGGCGGCGCGGGGAGGGGTGGTGTGG
ACGGAGAAAGTGAAAGGTGAGGCACGGCCCTGCAGATTTTCCAGCGGATCCCCCGGT
GGCCTCATGTCGCGCAGTGGAACCGATCCTCAGCAACGCCAGCAGGCGTCAGAGGCG
GACGCCGCAGCAGCAACCTTCCGGGCAAACGGTAACT
>DHS102
GAGCCAGAGAGCCAGACGACTAGTGTGCGCGAGGGCCGCTTCCGCTTCCGTCAGGGG
AGGCCAGAGGAAAAGGGGAGGGGGGTCGGGTGCCGCGAACTTGCTCTGAGGGGGTTA
GCTGCGCCTCGGATTGATCTGCGTCGTGCCTCGATAGGCAGTAACCTTCATCGCTGC
GGTTGCGTAGCGTTTACCCATGCGCCATTTGAACTTGTGCTTAAGGGATTTATCTTT
GCC
>DHS103
CGCAGTTAAGGCATTGCTACTAAGAAACTCGTCTCTGAGAAATATCGGTGGTTGGAA
GGTTGTAGGGTGCTTCCACCGAGAAAGCGCCTACCCAGTGAAATACGCCTGTGACAG
AAAAACCGAGGCCAAGCCCTGCCAGCAGTCTTGCCCAATCACGGTGCTGAGACAGTG
AGACAGCCAGCCCGGGGAAAGTAGCCGTGCTAGGACACAGGAAGCGATACCACAAAG
AACATGTGAGTCTTGTGAACAAGGAATG
>DHS104
GAAGGAATTCAGGCTACGGAGAGGGAAAAAAAAAATAACCGAAAGCTGGAGGCCAGG
TGCCGAGAGTCGGAGGTTTCCGGAGAAGCGATGAAGCCCAGCCAGCCCGGAGGTGAC
GTCACTCGACAACCCAGTCGATGATGTCATCATTGGATGCGAGAGGGGCGGGGCTTG
GTAGTCCCTCAGGGGAGTTTAAGGACCTGGGGTGTAGGACCCATGGAAGGCTCTCAA
ATAGCAGAGCTAGAAACACTGGCC
>DHS105
CTAGGTGAGGCCAGTGGTAGAGACTTGGTGGGTGCAGACTGGCCTACAGTAGGCGGG

GCCTCAGGCGAGGAAGCGTGGTCATGCTACAGAAGGGGCGCAGTTTGAATGGAGGCG
AACCAGTTTGGATAAGGGGCGGGGCCATGCTTGTGTGGGTGTGGTCAGTCGCAGAGG
GACGGTCATTGGGGGGCGGAGACTCAGGTGGGCGGAGACTCAGGTGGGACGGACAGA
AAGGAGGAGTCGTACCTG
>DHS106
GTGCACAGGAGGAAATTTTATTGTCTCTGATTTCAACTGTGGAGGTGGGTGGGGAAG
GGCTGGAGGCCTGGGTGCGGGGGTGGGGGGGGTCAGTCAGGGGCAGGACCACTGTGT
TTTGGCCGGGACATAAACACTTAAAAAAAAAAAAAAAAAACGAGATCCAGGGAATTG
TGAAAAATTGGAGCTGGGCCGGGTGCGGTAGTGAGCACCTGCAATCCCAGCACTTTA
AAAGGTCGAGGTG
>DHS107
GCTTTCGTTCCAGAAAACGTGCCAGAGGGCAGGCCGTCTTAATACAGTGAAAGAATT
GAGGAGACTGGAATGAATGGTATAGGTGGTGGGGTGGTTTGAAAGACGGGAACGTAG
CGGGCGGAAACTCTCGAAAAGCGACTGCGCAGGCGCGCGCCGGGCGCGGACGCTCAC
GGGTCACCCAGGGTATCCGCCCCAAGCGGAGAATATCGGCGCAAGCGCGGAGTAGCC
GTGTCACGCATG
>DHS108
CTCTCACCCGCCTGTTCTTCAGCGTCCTCGCCTTCTCTCTAGGCTCTGCCACAAGGA
GCTAGGACCACGCTCACCTCACGATTTCCCTACCTGCCTGTCTCGCTTTCTCACCTG
CGTTCCCTCCTTTCATCCCGGAGCCCGGAACCTCCGCTTCCGGCTCCACGTCCGCCC
GGAAGAAGATCTGCTGCACACTTCCGTTTCCGGTCCGTGCCCTTGGGGCTCCGTGTC
CTGCTGTCTTTCCGTC
>DHS109
AGACCTTCAATAGCGAGGTTGGTATGGCAGCCGGGTTCCCCTGCTCCTGCCCGCACC
GCCCCCACCCCTGGTCGAGGCCGGGGCCCGGAGGGTGGGCGCGGGCCTGCGGGTTTT
TTAAGGGTGGGGTGAGCGGTGGCCTAGCAGTGCCCGTGGGGGAGGGGTGTTTCTCCT
CTGGGTGCCCCTCCCCCGGGTCCGGGAGGAGGGTTGGGGGAGGGCCGCCCTGGGGCG
GAGGGAGGTTCTGGCGTCTCCGGCGTCCTATGGAATGGCTGCGA
>DHS110
CCAGTACAACCTAGGGACGTCAGGGGTCCTGGGACCCACGAGCCGCTCTCTGCCGGC
CGGCTCCTGGCCCCTCCTAGCAGACCCAACCGACATCCTTCACCCTCAGTTAGTGCG
CAGGAGGGACCGGGACGCCAGCCGACTGCGCTCCAGAACTATTCCCATTGGGCCAGA
GAATGAGTGACGCACTAGGCCCCGCCCCGGCCCGGTGGGTTACTCCTTTCATGCCCG
G
>DHS111
AATCTATGCAGCAAACAAGCTGGAACCCGCTGGGTGGCACCTGCAAGCAGCCGCCCG
GACGCACCCGTAAGGACACACGCCGGAAGTGCGTTCGCCGCCATCTTACTTAGGGAC
CTGCTGGGGTGCGGGGAAAAGGCGCAGTCTCGGTGGGATTGCGTGCAGGAGGGTCGT
GGTCTGGCTGTGGCGGAGGAGCATAAGAAGGTAACGCGACCTGGCGCGGCAGACAGG
GCTCGAAGACCTCATCTTGATTAAGAAGGCCACAAATTCG
>DHS112
GAGTCCACATAGTGCGTTTGAGACGGCTGTGGCGGCAGCAGCTGGTGCCTGTCAGTA
ATCACGCATAATGCTGCCGCCGCCGCCGCCGCCGCCGTCACCGCTACTCTTGCCACC
GCCGTCGCTGCTGCTTGAGATCCTGTTGCTAGTTTGATTAACTCAGGGTTGGGCTGC

TGCGTGCGACTCTTCCAGCTCCCGGCGCGTCCCCAGAGGTCCCTACCGGGTGGCACG
ATCTATTATCTAGGAAGCCAATCGATG

>DHS113
GGCTACCGGTTTCTAATGACCTATATGTAAGTTAGCTTTATAAGAAAGTTCTGAGTT
TGAAACTCCTTTCAGCAAGTATTTGTAAGTCAAAAAAGCGAAGAAGAGGCACCATTT
ACTGTCATTTCTCCTTGTGACTGAGATTACTTACTACAAAGAGGAACTGACCACTTT
CTCTGACATCTGAAATAGAAAGCTACAAAGGCTGATAGGCGTGGCTTCAAAATTGT
>DHS114
AACCTGAGTCCCTGCAACTACATTTCCCAGGGGCCCCTGGGGTAACCCTAATGTGAC
TCTGGGGGCGGGGCCAGTTGGACATCCAATGAGAGCACTTCCCCTGCAGACTTTGGA
AGTGAGAGCACTTCCTCATAGACCTTGGATGTGGGAGGATTGCATTCAGTCTAGTTC
CTGGTTGCCGGCTGAAATAACCTGGTAAGTGGAACTCTGTAAGGGGCTTTGAAATTG
>DHS115
TGTTCCATAGATGGGGACACTAAGACCCCAGGTCACAGGCTGCAACTGGAGATCCCC
AGACAGCTCTGGCCCCAGATGGTTTCTGTGTGACCTGAGCTGGATTTGGGGCTTCCT
GTGAAAGCTGGATGCACTGCCACATGGGGCCCACATGAGTTCCTGCCTGCCCACCTG
GGACTGGTGCTGAGTCACTGTGGTGCCCTGTTAGCAGGTTAAGCACTGTTCTGCCAA
AATC
>DHS116
GTCTCCACATCCCCTAGAAAAGAAAAGACGGGTGTGGGCCTTAGGTTACAGCGCGCC
GCCAGCGTTTGGTTGCATGGCGCCGGGGGAGGGCGCCCTAACCGAGAAGCTGCTTAA
TACAAAGAGCTCCAGGCTCCTGGCGGTTCACCAGGTCTAAACAGCCGGGCTTTATTT
GTGGGGGCGATTGAAAAAATTGAGGGTCAAGATTGGGGTGCTGTGCAAATAAATGCG
TT
>DHS117
CGGACCGTACACAACCACTCCCGGCATGCCCCGCGCACGCACTACCTCTCCCACCCC
GCCCCTCTCCCGCCCAAACACGTGACCTCCTTTCGTCTCCGTCCACGCCCACTTCCG
TTCCTCCACTTTCCCTTAGGAAGGAGGAGGGAGCTGGGGGTGTTAAAAGCGTAGCGA
CTTCCTCCTCCTCCCCGCCCCCGCTCCTGTACCTCCCGCTACAATGTCTTCCGGGTC
GCTAGCGCCTCGACGCCTTCTGGGAAA

>DHS118
CCTTTGCTGGATTATCTCCTTTCAACAGTTTTATTGTAATTACCCTGAAAATGCCGC
TATATATAGAGTGAGCCAAACAGCAGCTGTGCCTGGGGGGAGGGGGAATAAGAGGAT
GGGCGGGCAGGCAGCAGGGAGGAGGTGGGAGGAGGAGACAGGAAAGGAAAGGGAATC
AGACAGGAAGAAGGGTAACAGCAGGGGGAGGGCAGGAGCTTCGGCAGAGATCTCAGA
ACTCTGGTCTTACGGAGAACC
>DHS119
CCTGACTCCACAACGTTCAAGAGAATCACTCACACACACACACACACACACACACAC
ACACACACACACACACACACACAAGCTATTTACTACCTAAGCTAAACCAAAGAGAAT
GCTGCAGGCCCAAATATTCATATTGTGCTGGAATTCATAAATAACTAAAAATCTTTG
ATTCTCTTCTATTTTCTCCAAAAGGGAAACAGTTTTAACACATGGGATTAGGTTCAC
AAAAGATTTTCTTTATTTCCTCCTGCTCATGC

>DHS120
CAGCTGATAACGTTGATACTGTGCAGGCACACATCTCCCCACATTCCCCAGGCTGCT
GAACCAAGTGTACCAAACTAATCGCAAGGACACAGCTTTGAGCCTCCCCGGCGGCAG
TCCCTGGAGAGAGTCCGCTCAGACAAAGCAAAGCGCGCTCCCAGCGGGGAGGGGTGG
CCACTTTTTCTTTGTTCTCCCTTGGAAAATCGGCTGTCTCTCAAGTCTCGTAAACCA
GCTG
>DHS121
GGAGGGTCTGATCAGGGTTTGGGGCTGTAGATGGGGCATAGGGCAGAGGTGGAACCT
AGGACAAATGCAGGGGCGGGGCTTAGGACTGAGCGACAGCGGGGTCTGTGGAGAGGG
GCCAGGACCACACGCCCCGTATGGAGGGCGGGGCCTATGGCGAAGGGGCGTAGCCTA
GGGAAAGGGGCTGGGACCAAGGCTGGTGGGGGCGTGGCCTAGGAACAAGTCTCGTGT
GGAGG
>DHS122
CACAGAGAAGGGCGAAGACCCGCCCCTGCAGGTGATTGACGGGCACTCTCCTCCAAT
GGTCAACGCGCTCGGCCCCGGCGGGCTGGGCAAGGGCCGCGGCTCGCGGAGGAATCT
CTCTTGCCCTTTAGTCCCGGGCAAGGTGTTGCGGCCGGCGCCATTTTCTCGAGCCGC
CTGTTTCGGGTGCCGCCATGTTGGTGAGGAGAAATCACCGTTACGGCCACTGTCCAA
ATC
>DHS123
CGGACTGCTCATTGTAAGGAAAACTATTGCTGGACATTGCCGCGGTGCTGGCGGGTG
CGGGGTTGGTCCTGCCGGGGGTTCTCTCCAAAAGGTTCCAGTCCAGAGGGCACGGAA
CAAAACTAGTTCTCGTACGGAGGAGCGCGCGCAGGCGCTTCCGGACTCGTCACGGCT
CCCTGAGCCGACCTGGCAGTCCACCCCCTGCGCATGCGCAGTGTGCAGGCCTGCTAG
ATTACCACCCGGG
>DHS124
CAGGGAAGCGAGAGGAGAAGAGGGCCCTTTTATTTTCCACTCAGATTAATATGAAGC
AAAAGTCAAATATTACCTGCAGGGATTCTCCCTAGCGCTACTCAAGCCGTCAGACCC
ACGGCATGCACAATCTTCCTGATAGAGGAGCCGCCGGACTCCCTTATTTATAGCCCT
GGCGATTGACAGCCGCAACATCCAATGACGACTCCGCCACCGAGAGGCGGGCTTCCT
AGACT
>DHS125
AAATTGGGGTCTCTAAGGTCAGGGCCATGCCTTCCCCTCAGACTGAGGCGGCTGAGG
GCAGGGTCCTCAGGCTGATGGCTGTGGGAGTCGTGCCACGCCCCTCGTGCCCCCGGG
AGGCCCGGCCGGGGCTGGGTGGGGGGATCCGGTGGTTCCACATGGTGCAGTTCTGCG
CTTTGGCCACCAGATGGCAGCCGAAGCTAAGGGATGGGGCTGCCGTCTCCCGTCTTG
CTGTCCTT
>DHS126
AACTCTCGCGAGTCGAGGTATCTTCTCCCCAACCACTGCTCTTATTTTAATTATTGC
AGACGGAAGTTGAAGACTATTGACATAGTAAATAGCTCTGGGTGGCTTGAAACGAAA
GTTTAACTTTGCGGACAAACAGGACTTATTGTAGGGGGTGGTCAAAATAGTCCCGGC
GGGGCGGGGCCATGACCCCTGACGTCGCCGGTCCGGCGCGCAGTTCAGTTTGGCGGT
TCC
>DHS127
CCGTGATCCTACTGCTTTTGTCATTTCTTCAAAATACAGAAACTGCCATTTGTAGGG

TCAGTGTATCTTCCAACGCCTAATTCTGTTGTCTTCAATATCACCCGTCATTCACTC
ACCTCCCCTCCACCCAAGAAATATAAGTTCTGCTGCAATTTATGTGTGAAATAGGAT
CCAATTTTCCCCAGCAAAAGATGGGAAAGAAAAGGCGAGGAATAGGTCAAATGAGGA
AGATACTCCCATG
>DHS128
AAAGAATGTTACGGGGCATATCCTTTCTCTCAAATAATGGAACGAGGTTAAAAAAA
AAGGTTAAACATAAAATGGCGGCGGGCTGGATCGGATCACGTGACCGCCATTTCTTT
CCTTCCCACCCAAAGGAAAGCCCTTCCTTCAGAGCAAGCGCAAGACATATAGCCCCG
CTCATTGTTAAGCGAGGCCAAGGCAACCCTTAGGCGCGCTCTATTTCCCCTTTCGTA
GCGA
>DHS129
GAGAGCAGGCAGAAGGTCAAGGAAGTTGTACAGGGAATGCCACCTCCCTGCACACCA
TCATCTATCCTGGGCTTTCCAGGGCAACCTAGATTTCAAACCCTCTGCTCCCTGGTG
CCCCTGTGAACAGCAGAACTCACCACCCTGTGCCCCTAAAGTCCACTCACTGCAAAT
GCAGGGCCCCATTCTGAGACTGCATTCCTAGCAAATGACTCTCCCTGGTCAAGGAG
>DHS130
CAGAGCAGGACAACTCAAAGAAATTCCTCCTCCCCCAAGCAGACCACCTCCCTGTGT
GCTGGGAAAGAAAACATGAAATCATCTCTTTACAAGATGGACCTTACTGAGTCACAG
AAAGACACCTTCAAACTATCAGCTCTGTGGTCTTCCTGGGGTAACAGAGATAGGGAT
GCTTTGTAAAATAACCTATCTGTGGCTATCTTCTGTCTTGCCTAACCTTATCTCCAT
CACTTCCCAAGAACTC
>DHS131
CCTGCGCCAGTCTTTTAAACCGGCTCGGAGGCGGGGCTGGCGACGGCGGGAGGCCCC
GCCCCCTGCCGGCCCCGCCCCCAGCTCCACTTTTGGTTTCTCTTTCCGCGGTGGCGT
CCGGCGAGGACCGCTTCGGCCCTGTTTCCCTCTCTTCTGGACCCTCCCGCGGGGCCC
TCTGCCCGCCTGTTCGCACCTGCCCCAGCACCCGCCTCTCGAGGGGCTCTGGCCCCG
ACCCTGCGCCTTCCGGCCACTTCTCGG

>DHS132
AAAGAGGAAAGGGCGATACCACCCGGAGACCTCCGGCAACTTTCTGCCCGCGGCAGC
TGCTCATTCACGCGTTTCTTATAAGCCCAGGAGCCAATCAGAGGCTGCGTCGGGAGG
AAGTTGGAGCGCGGACATGCCCCCTCTACGGTGTCCTTGCAAAGTGTGGCAGTCACT
ACAGGTTTGAGGAAGGCTCTGTCGCTGCTCTGGCCGTAGGCACAGCGGACTCTGGAG
GACACTGGTTTGTA
>DHS133
ACGTGAGCGTTTGGGATATAATCTTTGCAAATTGCCTATAGTTCATACAGCACAGCA
AGAGCCTCTCTCTCTGCAGCCAGTCCTTTATGATCGATCATACCCACCCCCTCTCCC
AATGGGCTGTATCCTAAGGGTAAGTAGACAGCATGGGTTCTCTTGCTTAAAAAAAAT
CGTTAACTCAACGGGGGCGCCTGCTGCTTGATTGGGAGTATGTCGGCATTTAGAGCC
TG
>DHS134
AGAAGACAACAGACAACCGTTCAGCAGTGTGTCCATTTGTCCCTTCCCCTGTGGAAC
CCAGAAAGTAACCCACTCCAAAAATCCCACACTTACTTTCCGGCAGCCGGCCGCGCC
GCGTTTCCGAGAAAGGAAGCTGTCACTGTGCGTCTTCTGCGCAGGCGCCCGAGCCCT

CATGGGAGGTGTAGTCCAAGTTTGTGGGAAATGTAGTTCTGAGAACCGAGCTTGGTG
CTAGAATCTACCTGAGGTTT
>DHS135
TGTTGTGGGTTTGTTGTATTGCTGCTGTTGTTGTTAACAGCAGCTTCTCATCTTGTA
ACTACAAAGTAGCTGCTTTAACGCCATAGTACTTATTAACATTCTCTCATTATTGTT
AATAAAATCTTCATTTCTGAACACAGCAGGGCAGAGAAAGAGAACAGCATAAGGCAA
TGGCCACATCCATGCCATAGAATACACGTCAAGCATATACAATCATTTAAGCATTCA
GAATGCCCAAGGAGAAATT
>DHS136
AGGGCGGTCCTTACAAGACGGGAGGCAGCAGAGAACTCCCATAAAGGTATTGCGGCA
CTCCCCTCCCCCTGCCCAGAAGGGTGCGGCCTTCTCTCCACCTCCTCCACCGCAGCT
CCCTCAGGATTGCAGCTCGCGCCGGTTTTTGGAGAACAAGCGCCTCCCACCCACAAA
CCAGCCGGACCGACCCCCGCTCCTCCCCCACCCCCACGAGTGCCTGTAGCAGGTCGG
GCTTGTCT
>DHS137
CTACATTTGGAGATGGCTTGAGTTGTTTTTTTCTTAAGCAAGCAGCAAAAATGAGCA
GGTTTTTTGTTGTTGCCTTTTTTAACCCTTCTTGCTCTCAAACAATCACACATGGAT
TATGTCTTCTCGAAGCTGCCTTCCACAGAATTTTTCTTTTTTTTTTCCTGTCAAAAA
GCTTGAAATGTAAAAAGTTTTAATTAGAAATGTGAAGGGTGAATTAAACTCATTTCC
TTCAGATCTCCCAAC
>DHS138
CCTTCACTTAAGGCACCCACAGCTCCCTGACCTGCGCGCAAGGCCTCAGGCCACAGG
GGTCGATGTAGAAGACTCAGCCATACCTTTGTCCCGCCCCGCCGCCCGCCATGTCCT
GGCCACCTCATCTGAACTCTGCAGTTCTCTTCGCTCAGGAACACAGCTGATAAATCC
TAGCCTAGCCCGTATCTGCTCTAATCTCTAAGCCCCTCCCAGGGGCCAGGGGTGGGT
TGTGGCTTATACAGGAGCTCC
>DHS139
GAATGTTGCACATCTTGGAAAGAAAATCTATAAAAGTAGTCTTTCTGCATTATATCT
ATATTTCAGCTTTTTCTTCCTCTGAAATAATAATTGGTGCAGCCAGTGGGCTATTTG
CTAAGCAGGAGGCACATTTTCCTTGCCTGTACAGACAGAGGGTGAAAATAAAATACC
ATTACTGGTTTGTAGCACTGTGGCAGTGGGAGACGAGAACCAGGCCTCATATCCCAG
CTGGCATTCAGCTCTGTCTTCCTCCT
>DHS140
ACGGGCCACCATCTTACATTAGGGTAAGACTCCTCCGGCCTCCCGTGCGCAGGCGCA
CGCTGATCCGGCCAGGAGGGGCGGGGGAGGGGCGGGAGGAGCGGCGGGCGGCTGAGT
GGGAGGTTGCGGTGGCCGCTCCCGCAGCACCCTGGGAGTTGAAGTCCTCGGAGGGTA
ACCTCCCAGGGGCACGCCGGGAGTTGAAGTCCTCTCTCAGAGGGCGGAGGGGATCGC
CCACCTCACGTGGCTGCTGGACTGATG

>DHS141
CTATACCACGCCCAGAATTCTCTCAAAAGGGAATAAAACACAGGTCAAATTCCTCAC
CCACACACTCCACAGTTCAACCCCTGCCAGGGAAACCAAAAGCAGGAAAGGATCTCC
AGCGGCGCCATTCTCATTTCCGGTCCCAGCACCCCGCCTCCATGACGTCAACGCGCC
GCGCCACCGGGCTGCGTCATCTCGGCGCGCCGCTGCCAGGGCTGTACACCTGCTGGC

TGCCATGGCTGAGGTGGGCCGTACCGGGATCAGCTACCC
>DHS142
CAACAACAACAAAACGTGCAAGAGCGCGCCTGCGCACACGGTCCGCTACGACCCCCA
TCCCTCTTCGGGCGTGGGGTGGCGCTGGGCGCATGCTCCCCGGCAGCGCGGTCATCC
TCGCCTCCTTCGGGCCGCTCTAGCCGCCCCACTCGCTGCTCTGCGGGGAGGCGGGCG
CTCCCGCAGGGGTTCCTCCAAGATGGCGGCGCAGAGGAGGAGCTTGCTGCAGAGT
>DHS143
ACATGTCCTGCGTTGCCATATGGCCGCAACTACTTGCATCTCCACCCAGTTAATCTG
CTCTGGGGCCTACAAACATCTGACTTTACAGATAATGCTCTAAGGGTTCCTTGGGGC
CTGCAGTACTGTCAGGACAAACTGGTTCCTTAGGCAGCAATGAGATTAGTGAACTAA
ACGTCCACATATCAACCTGAATGATCTTCAAAACACTGCTGAGTGAAAAAAAGTAAG
AAACAAGCTGAGACCTATAGCA
>DHS144
TTTTCCTACAGGCTGATCGAAGATGATGAAATGCCACATTCGCTTCAGTAAACAGAC
ATCTCAAAGTCCAAAGGAAACAAACTCAAAGGTTAGTTCATTTGTGTCTCAAATAAT
ATTCATCAGCTGGTTTGCTAACTAGGGCAAGTCTCTCTGAAGCAAATGCACGAAGGA
ACGCGGGTATCATTATCCACTAGGTGGCAATATAGAACAGGAGTAGCCTTAGCCTTA
GCCTGTTTACTGACACGTGG
>DHS145
ATTAAAGAGGCGGTGCTAAGCTGCAGACGGACTTGCGACTCAGCCACTGGTGTAAGT
CAGGCGGGAGGTGGCGCCCAATAAGCTCAAGAGAGGAGGCGGGTTCTGGAAAAAGGC
CAATAGCCTGTGAAGGCGAGTCTAGCAGCAACCAATAGCTATGAGCGAGAGGCGGGA
CTCTGAGGGAAGTCAATCGCTGCCGCAGGTACCGCCAATGGCTTTTGGCGGGGGCGT
TCCCCAACCCTGCCCTCTCTCATG
>DHS146
AACTGTGGACTGGGAGAGACATTTGAACCCTCTTTTCTTTTCGCTCCCCTTTTGCCC
CCTTGGGGTGTGTGAAGCGAGGAACGTAAAGGAAGGCGAACATTTGGCTCTCTTTTT
CCTTCCCCTTTCTCCGTGGCTGTGTAGCGGAAGAAAGGGAAGAGAGACTTTTTGTTG
TTGTTTCCTTGACTGGGGTCTCCACCCTCCTGCTGCTTTCTCTGCGCTTCGATTCTC
GTTATTTGCC
>DHS147
CCTGTGTGAACTCGGGAAACTCGCAGGCAACACAAACGCTGCGCGCGAGTGCCCGCC
CGCTAGGCGAGCGCGCGCCGCCAGGGCCGAAGGCGGGGTCCCGCCGGCCCGCACCTG
GGAGAACACGGCCTGGAGCTGGGCACGCGGGGTTGCGCCGGTGACGCTCCACCGAGT
CTCGGACGCGCTCCTCGCGCCCGCCGCGCCCTCGGAAGCTTGCCCCCGAGCACCACA
GCTAGCAGGGT
>DHS148
AGCAAATGCCCAGAGCAAACCCACCCCCCCCGCCCAAAATAAGTTTCTTTAAGAAAA
AAAGGAAAAGGGAAAAAATGCGAAATAGAAAAAAATGATCTTCTGTGCTTTGCTTCA
ATGAAAATGAAATTTGAGCTCTGAGACTGGCCCCCTCTGCTCTGCAGTTTTTTCCAC
TTCACCTTCTCACCCCGTCCTCCCAACACAGAGTGTGCAGGAGCGAGAAGGTGCTGT
AAG
>DHS149
ATGTAGTCAGATGAGGCTCCCAGTCCTGGCTCTGCTGCTGAGCACCATTGGGCCGGT

GGTTGGGTGAATCTCTCAGCCTCTCTGAGCCTCCAGCACTTCGTCCTTAAGAGTGGA
ACTGGTCTGGACCTCCCAAGGGGGGGTCACTGGGTGCAGAGTTCCTGCAAGGCTGGG
CCCGGTGCCAGTAGCGGTCGCTCTGTGGGGCCTTTCGCACGGTTGATTTTTATCTCC
CTGTGAGATTAGCGCCTGC
>DHS150
AGAATGTGGGAGAATTCAACTGAGTTGCTGATTGGATTTTCAAACTCATCGCAGAAA
TCACACCTTTGAAAGCCAAAGCTTTCATTTGTAAAACCTAGGCTTTTTAAGAGAATA
AAAGTATTCTTGAAAATCAAAAGGAAAAATTCTAAAAAGCAGTACATCAGACTTGTG
TCCCAAGCAGGAAATTAGAAACACAGTGGAAGATGATGTAAGAGCTAATTTTTCCTC
CATATGTGGAAAACATACATGTACACACACACAC
>DHS151
GGGGTTTGACTGTGCTTTTCCCTCTTGCTTCCCTCGCTCTTTCTGCAGCTGCCACGA
AAACCCGGAACGGCGGAGCGGCGCCGCCCCTCGCGGCACCTCCCTGGCAGCCCTTGG
AGGCCGCGCTGGGCATGCTCAGTCAGCTGGGCCGCCTCAGCTCTCGGAGTAGGAAGC
TCGGGCGCTCCGGCTGTAAGGAGCCGCGGCGGGGGGAAAATGGAGCCCTTCACCA
>DHS152
ACTGAGGTCTCGCCCGTTCTCGCGGACCTGTCCGTGCGCCCAGCGGCATCACGTTCC
GGGATGCCCTTTGCAGCCACTGGACTCCCAAGACCCCGGCCCTGGCCACCGCCATCT
TCACCTTCTTCACGTTTCCCTTCTGCACATGCGCAAAGGCCTGTCACGGCGGCTGAG
CCGGGCCAAACGTGACGGAGGAAGCCGGGAGTGAAGAAAAGCCGAAGGGTTCAACAG
GGGCGGGGTTTAAATACGCAGTTGGGAAG
>DHS153
CCTCTCGGGAGGGACTTAGGGGGCGTCGAAAATGATGTAAAGACCGAGTTGGCGGCG
TGGGGAGTTGTGGCTCGGAGGGCGAGGGTAACTTTCTGGGGGCAGGGGAAGGCAAGG
CTGTTTCCCCGGAACGGGACCGGAACAGGTTTATTCGGGGTCACAGAAGGAGTCACC
TGCGCAGATACATGAGGCTTAAAACCTGCCCTTTTATTAATTCAAGTACTGTTGAGG
GGCTG
>DHS154
CGAGGTCAGGGATGAGAGACTTTTTTTCCTAGAATTGGTAAGACAGGAAATCAATCA
GAGGCAGAGCGACGCCTCTGGCTCTGGTCTAGTGGTGCAGCGTCTCTAGCCCTCGCC
CCGCCCACCGTCCCCGCGAGGCGTCCACTCGCCGAGCCCCGCCCTCACCCAGCACCC
ACCCCGCTCCTCGCGCCCTCCCCGCCTGTTCCCGCCCTGTGCTGAGGCTGCGCAGTC
GGTGCCATCTTCTAC
>DHS155
GCCACATTTAGCCAGTTGTACATTCATTCAGGCTGCAATTCTTTCTTATCCCTGGGC
TCAGGGCCGCACCCAGACACACGAGGCCGGGCAGGCATGACTCAGCAGCCCTTAGGA
TCTGCGTCCTGTGATTTTCTCAAGGAGCACAAAGGTGAGCGTAGCCATGGGGGAGAA
TAGGGAAGGAAGAAGGGCCAACAGTGGCCGAGTCAGCTCTGCTTAGTCTGAACTGCC
CCT
>DHS156
TTTCAGCAGTAGGTCCAGGAAAACAGCCGCCCCGCCCCCTCCACCTCCCGAGACTAG
GAGCCCGCCAGGGTTAAGTAGGAGGGATACCCTAGGGGCCCCGCAGGGTTTTTAGGG
TTGGGGTGGATTGGAAATTGTTCATTGCCTTTCAGTTCCGGGACATAAAGCGCGCTG
GAAGGACACTGAGTCACCAACCACCGCCATGGGCCGGAAGCCACCGCCCTCGCGTCA

ACTGCAATCTAGAGCG
>DHS157
TTCTCATATCCCATGACATTCCATATAAATTGCATTATTGCTTTATAGGCCACACAA
CATTCAAAAGTATGTTTATATGCATTGCCTGTTTTTCCTTGATCTGATAAGACTTTG
ATACTGATTTTACTACAGAAATTTAAGTTTGAAAATACCACAGTTAGTATTTCTTCT
CAGAACGTTAATTGTTCACATCACTAATCACTACATGCATATTTAGCTCTGATTATA
TTGCTCAAAAACTGCACCCATTATCAGAACAGT
>DHS158
CAGGCAGGAGGCAGATAATTCATTTTTAATGGGGTAATATGAATTCATCAACATGGA
CAGATTCATACACAGAATTACAAACACAACTTGCACACATCCTGTAGATATAAACAG
TCCTTTGCATACTATGTCCGGGGTTGGTGCCACGTTGCCCCTAGGTGTCCATCAACC
TGCTCAGGGGCCGGCAACGCCCCCTGGTGGCTGCCACAAGAACAGTTTAATTCTAAT
TTCACTTCATTACCTTCACACTCGC
>DHS159
CAGCTCCTTCTCGTTGTACCGCATGGCTCCGCGGGGAACGGGAACCCGGGCCGCGCC
CTCCCGGCCGCCGTCCCCGCTCAGGCTGGGGCCGGCGCCAAAAATGTCTCAGGGCGC
AGACACGGAAGCACCCCCAGGGCTAGTGCGCCTGCGCACCTTGGGCCAGCCATGCCG
CCCGCCTCCGCTGCGTGGCGCTGGCGACCCGTGGCTGCTGGCGTGGGCGGGGTACCG
GTTGGTAGGAGAGACGGACC
>DHS160
AAGCAGGAATTAGCCTTGTAATCCGATCCTTTTAGTCTCTGCATTTAATTTTAAGCA
AGAAACCAGGAGACTAAAGATGTAAGACTGACGTAGAACAGCAGAGGGCAGTATTTA
CTCATGACTGAACCCTCTCTGGGCGCAGAAAGTTTTACGTTTGTGAGGGATCCTTGT
GGCAGAAATGTATGACATATGCCCTGTATTTGCATTGTTTGGCTTGAATTCTTATTT
TCTAGGTTGGTTCCCTTAAAGG
>DHS161
TCCCAAAGTCTTGTCTACGTTCTGTTAAGATCAATAGTGAGCTGCTTGAGGCTGAGT
ACTTTGTGCATTGTGAAGCACATTCTAGAGATGGGGAGAGACACATGGGGCCTTATC
CACATTGGAGTCTATATTGTGATCTTCAGAGGATAAGTCCGCAGTAAAGGCTGTGAC
TAAATTTATGTAGCCCATGCTTAAATGAACAAATACTGTGGCCTAGGGAAGTAGC
>DHS162
GCGAGTGTATGATTTGGTCACAGTACTGTTGCTTTGACATGCATAATGCTAATAACT
AAGAAAACATTACAATATTTTTGAAATTTATCTGTAACATGTTGAGGTAGGCTTTGG
TAATCCATTGCTCACAAACCTAAAGAGGAACAGTTTTTTTAATCCAGAAGAGGGCG
CAAGAGAAGGCCAAATGTGTTGAGTAATTTGTTTCTAATTGAAAAGCTAGATGTATC
AGGACTCAAGTTTTGCTGAGTC
>DHS163
CACTGGAAGAGGCAGTAGACTCACCTCCTCACACCCCACTCCTTAACCCTCACCAAC
TTGTCTGCTAGATCATGGTGCCCAGATACTTGGAAGGGTTTAGCCCTGGGGCTCTCA
CATCTCTCTTGTTTCTCAGAAGGCAAAATCCCATCAGACCTGGAAAGCAGGAGACAG
GGTAGCAAATCCAGAGCAGGGGCTGTGGAACCACTTCCCATGTGAAGAGTCTGAGGG
AGAG
>DHS164
AGGACAGATGGGACGCTCTCTCCCCGCGGCCCGCCGGGCTCTCAACCTCGCCTCTGG

TTGGCCGGGTCTGCGAAGCTCTGGGCTGCGGTTGGTCCTCTTGAAGAGTTAGTTCCC
GCGGGAAACTCGGGGAGGAAACGAGTCAGCAACCCCAAACAGGCTTAGGCGGGCTAG
AAGCCGGGGTCGTGTCTGACTGGGGCAGTCGCACGGCCGGCGGTGCGGCCCGCTCGG
CTCTCGCGGAGGCCACAGCCTCGAGAAAGGGT
>DHS165
GTGAAAAGAGGGACCCGACAGCACCCACCCTCCAAGTAGAGGCAAAAGTTGAAAGTG
GCGGGCTGGCTGCGCTTCCGAAGAAACACGACTGGCGAAGGCTCACCTTAGTCTCTA
CGGTGGGTCCCTCCCTGTAGCCGACCCGTTCCTTGAAGCGGGCCAGAAACGCCGGCT
CGGCTGGCCGCACGTACGATACCTGGTTCCGCTTGCTCATGGTAGCTCTGGGTAAAG
ACAGAAGACAGGTTCCG
>DHS166
AGGTCACTCACCTCGGCTTACTCAGCACTTCTACTTCCTCCTACTTCCGCAAACAAA
CGGGCCTCGGGCCACGTGATGGCCTGACCACCGCTCTCCGCCAATCACTGGTGGCCC
TGTCCCAGGCCTCCTCTAGGCTCCGCCCCCGCGCCCGAGGACGGCGGGAGGCGGGGT
CCGCCATTTTTTTTAACTGCCCAATGGAAGGGGCTCCCTTAGCCAGGAGGACGAGCT
T
>DHS167
GTCCTGAGTCTGGTCCTGTTGAGTCCTGAGTCCTCCAGACTGGCCCTGCCAAGTTGC
CAGGCATCTGATGCAGCAGAGCAGCGCTATGCTGCCCTCTGCTGGCCACATGGAGGC
AGTGAGTCCCCACACTGCTGTCCACTCCTGGCAATGGGCTCAGGGGAAGTGGACTGT
GTTTCTGGTCCCTTGCTTCTGACAACCTGATAGAGCAGATATGGAAGGGCAGAG
>DHS168
TTTTCCGTTGATCCCTTAGTCTTGTAGTCGCTACTTTATAACCGGTCCCTAGAAGGG
GGCGGTTCTTTCCGGAAATTATGACTGCAGCTGTTTTCACTCCGCTGTGACTCAGAG
CGCTCCGGGCTGCAGGAGAGGAAGGTAGGGGACCAAGCTACCGGTGCAGGGCCTGGA
TCCAACCCCTGCTCTGGAAGACTGAGAGCTCCACATTCTAGCTCCCTTTCCCCAGTC
CCCAAATTTGTAGGGG
>DHS169
TCTGAGGACGCAGAAAGTTAATCCGCATGATCACCTCGTGATTATTTTCTGAGGTTC
TTTGGCCAGGGCAGCACGCCCTCTAGATTTTGTCATTTTACATATTGGAATGGTGCC
TTAGTGGTCTGGGCGTTACGTTACCGAAATAACCACCTATTAAATCTGGCTATTTTT
CAGTTGCTACCGTACTGCTTACCACCTGTTTTGTTTTTTTTTTAATTGAGCATTCAG
TTCAGAAAACTTCAG
>DHS170
CGGAGCATAGTAGGAGGAGGTGGGGTCAGAATGAGAAATAGGGATGCTTGGGGCAGA
AGTGAGGTAGAGTCTTAAGAAGAGGAGGTGGGGGGAAACTACATGGCATGGAGGCCG
TGAAGGCAGAGCATGATTAAAGGAGAATGGGCCCTACCTAGAAAAAGCAGGTGGATT
GGACCTGAGGGGGCGGGGCCTACAGCAGGGGCGGGGCTTAAGACCAAGCTGAAGGCT
GG
>DHS171
CTCACTCCGGACTCGACTGACGGGCAAACATCGCTTCCCCCCCACCGACTCTAGGTT
CCCCCCCTTTCTCCCCTCCCCTAGATTTTTTTTCCCCCCCTCCCCTACCTCTTTCCC
GGATGGCCTCTTAGACGACCTTGGATTGGTTAAAGTTCTTTAGAACCCGCCTATACA
CTGTTCCTATTGGTCCCTGGATACAAACAACGACGCCATTTTCCCACCAGTTCTATG

GAAACAGAAAGTTACGCCTCAAG
>DHS172
GCCAGACTCTAGCCGCTCTCGAGGGCCTTCGAACTACAATTCCCAGCCTGCCTCTCG
CGCTCTTAAGCCAGGCCCTGGGGCCTTGCCCTCCAAGGGCCTCGCACGCCGGAATCG
CCGGGCACTCTGGGAAGGGTCAGGCTCCGGCGGGAAGGCGGAGCCGTTGGGGAGCTT
GAGGGGAAGCGTGGTCTCTCCCGGAAAAACCACTTCCACCGAGAACGCTGAGCGAGA
GCCAGTAACAGAG
>DHS173
CTCTCCCCAGGAGATGTCAGATTCCCACAGCAGGCAAATCAGCTTGGCAGAGAAGTA
GCAAGAACCTGAGAAGGCATGTGTGCTGTGAGTCAGCCTTGGGCAAAACAGCCAGGT
CTCAATGCAGGGGAACCCTCTGGTGCTGTCAGACCCATGCCAACACTGCCCTCTACT
GGCCAATTAAGCTGGTTGCAATTTTCTGCAAAAGAAATACCAGTGTATAAGGAGAAA
TTCTGCA
>DHS174
GCAACGGAAACTATACAGGCAATATCCTATTAGTGTATCAAAATTTTAACAAACTAA
ACACAGTATTAAATTGCAATTTTTTTTACTTTTCAATAAAATACTTGCTAAACAGTC
TTGTCTGAAATTATAAATTGCCCAAAGTTAACCATAAATGAGTCATTCTATTGAATT
TAACAAACATTTAACTTTGCAAGAAATAAATTTTTATTTTTCTTCATTATCATACAG
CATTTAAGAATAATAAATCTGTCTTGA

>DHS175
CTGTCCAGTCCCCAATAGAAGGCGCGGGAGGAGCATGACATCATCAGCATAGAGTGC
CATTGGCTGGGCAGACCCTGCGGGCAGGACGCTGTCTCCAGTGGCCAGAAAGTTAAC
TCTTCCCTAGGCTGGAGCCATGTGGTCTTTAGGGGGTGAAGTTGGGGGAACTTCCAG
ACTTTTCCTTCCCTGGGTGACCAGTGTCGTTCGATGATAGGGGCGCCTATCCCAACC
AAACACTGGAAAA
>DHS176
GTGGTCAGGCTTGCTGTTTCGCGGAGTCCGGGCCAATCCTGGTGTTAGCTTCCAAGT
AGGGAGGGGACAGTATCGAAGGTGTGACCAATCTTGTTTCCAGAGCCTGAGTTGCGC
GAGCCGCCAAAGGATGTGGCCAGCCTGGGGGCGTGGCTGTCAAGATCTTGTCCAATC
CTAGAGTGAGCCCAAACTCAGGGGCGGAGTTTCATGATCCCGGAACCGGTGGTAGTG
TTTGGG
>DHS177
GGAAGAGTGCTTATCAGGACAAAGAGGGGCCCTCAGTTCCAGCAGGGCCAGCTGGTC
TGGCCCAAGACAGGAGGATTAAACAGAATTCTTGGATGTTCAATGCGGCTCCATGGG
GACCTCTAGCGGCTGTTTTGTCGAACTGCGGTCTCCAGCCTTCCCAGCCTCAGGACA
TTCCACACCTGGATGGATACTAAGTAATACCAATAACCAACATCTATCCTAGGCAGC
GTC
>DHS178
GCCACCCAGTACACGTACCCGCCCGCCCCCATCAGCCCCAACCCAGAAAGCACGCGA
CAGCTCCAGCAGGTCTTCAACAGGCGGTGTTCTGCTGGGGAGGTCGGCGCTCCGGGT
GTAGCTGGGGGCGCAGGTTTGGCGGGCGCGGCGGCGGTACCGGGAGGCGCAGTGATA
TAGGACTCAAAAGGCTGGGACAACCGAGACCCCATGCTCTGGAACTCGGCCTCAACC
TTGGGCGTCTTTGGTTCAAACTGGCGTAAAC

>DHS179
GACACCTCAACAGCTACATTACATTGAATTCACAGCATTCATAAAGTGTGGATTTCA
AAGTGAATCATCAGCCTAATGGAAAGTTCAGAGCAGAATGCTCCCCTCTCATTGGTC
ACTGTGCAGATCCTCGGCTGCCAGGAAAAGGACCAGAGCTGTCAACACAACACTGTA
GCCCAGGCAACCAGCCACACTGCTAGGAAGTTTTCAACACTTGAAAAAAAAAAAATAC
AAGTTATTATAGAAACTTTCAGGTGCTCCATAGGTTGTT
>DHS180
TAACGTAGCCTGTGCAACTCTGCCACCTGCTGGCCTCAGGTCCTCAAAATCCTGAAA
TTGGGGCTGGGCCTTAGATTGTGAACTAAGTGTCCTCTTGGCTGAAACAGCTCGCTA
TTCACTCCTATTTTCTTCTTTAGATTTCAATTAAAAGGCAGCTCTGCTAGTTACTGT
GTTTTGGCTGGAATGGAAAGCCTTTGGAATAGCAGTGTTCCAGTGTGTGAACGTAAG
TAATAGGAGTAACATTTCAGGCCAATC

>DHS181
GCGTTACGACTGGAAAGCACGCGTGCGCACAGGCGACAGCAGCGGAGGGGTGGGGCC
AGTGTACGAGTGCGCGTGCTCGGTGGGAGCCCGCGGAGTACCTGGAAGGAGGTGGGG
GCGAGGTAGAAAGGAGTGGGGTTGAAAAGCGCTTGCGCAGGACGGCTACGGTACGGG
GGTGGGAGGGCTTCGGAGCACGCGCGCGGAGGCGGGACTTGGGAAGCGCTCGCGAGA
TCTTCAGGGTCTATATATAAGCGCGG
>DHS182
GAGTAATGCAGGATGATGTGGAGATGCTTTAAGATTAAATAAGAGCTATATAAAGGC
TTGTCAGAATGAATTCTAATGTGCCATGAGACACTTTGCATACTCTTAAATATGCAA
AATGTCTCTTGGCACCCTAGAATTCATTCTGACAAGCCTTTATATAGCACTTATTTA
AACTCCGACTTTATTTTTAGGGAACAGGTACTCAGAGATACTCTTTGAATGGAAGCA
ACAAAGGGCAGTGTTTATC
>DHS183
AGAGGCGGATTCTCTCCTACTACAGAAAGACGTCTGCTGTTTTGATTGGCTAGGAAA
GACGTCCGTTAGGACGTGTTGCCCTTTCTGTGTAAGCTGTGAGCGTAGGCGGCCCTG
AGGGGGTGTGTTGCAGGGGTTTCCAAGCCCAGCACCAGCACCCTTGCCCTTTTCCAT
CAGGGGTTCAGCCTAGGGTCCCCGCTGGTGGGCGGCTCCCGAGTCTTGGAGAAGAGC
ACGAGAAC
>DHS184
GTTTAGAAGGCTCCCTGAGCAGACCCGTGCTTTCAATGCGCCAGGCCCCACCCCTGC
CAGGCGCGGTCCACCACCAGCTGTTTGACCATTGACTCTACCTTCTGTTCCAGGCCT
TCCTTCGCCTCTACAGTGATATCTTCCTTCAGCCCCTTCCTTTCCCATGTGATCCCA
CTTGGCAGCCAACCCAGCCTAAGGCCGTCTTATCCAAGGCCGGCCCCTCAGCGGCTC
CGCCCATCTTAGTCCCAGCAGGCCT
>DHS185
CTAACAGAAAGACTGGCGCAGTTAGGCTTTTTCGGAAATTCCAACAGCAACAACCAA
AATGGAGGCATTAAAATCGATCCTGATTCGACTGGAAACCTGATTAACAAGGTTAAG
GTCTCCTGGCATAAAGTTCAGTTACAATGCAGATATCGGTGAGACAAAAAAAAAAAA
TGACACGAAGAAAGGCTTGTTTACCCACCCTGACCCTGGGCACTATATGGCGCTTTC
CACCCT
>DHS186

TGAATATAGGCCAGTTCCAACCTCATTACACGATATTGCATCCCGCCAAAGCCAGGG
GAAGCAATGGCCTCCCCATGCACCAAACTGCTAATAACGTCAGCCACTTTAAAGCAT
TTAGTGGATATTACACAACTGCGACTGACCAAGTTACATAATGTCTTACTGCACACA
TGGCATTGCATGAAATGAAAAATATAATATACCAGTCCAAATTAGGGGAGGGGGGCC
AAATTATAGTCTGGAACTCAGTTTTCCTGTAA
>DHS187
GTGGCCTTGACACAGACAGCTAGTGTGAATCCCCGCCCACAGAGGGGCGGCACGTTG
GTTGCCGTACAACGTGGTGGTTCCCTGCATCTCTGCCCACGTCGGAGAGGTGCGTCG
GCTTCCGTACAACACGGATACTCTCTCTCTGACGCAACTTCCTGTCCTGCGCAATTC
TATTTGACCTTTGAACTGGCAAAGGCTTTTTTCTTCCTCTTCCGGGGACGTTGTCTG
CAGGTATGGATGTTGTTCT
>DHS188
GATAAGACAAAGCCAGTCACTCCAGTTATGGACAGCTCTTGACTAAAATGAGACCAG
AAATCATTGGCGTGTCACCTTTACTTAGAAGTTGCATATGGAAACAGTATTAGGAAA
GGCGCATCTCTGGTTAAGATAAGCTGAGCCCAGGTTTTCTTTATTCAAAATGAGGCA
GGAGGAGCAGATTTAGCAGACTGACAGATAAAGTTTTTATCTATCCCAGGCAATAGG
G
>DHS189
ACCTCTCTCACGTGACCCTGACCGGGCGCAGTCCCGTCAACGCCGGCGCGCGCAATC
GCTGGAAGTCGGCTCGGGGAGGCGTGGGGGGAGGGGCGGAGCGCGCGCCTCGGTCCC
CGCGGAAAGCTACTCCGCGCGTCTGGATTGGTCGCGAACGCGGCTTCCCCGCCCATC
CCGCCATTAGGAGCGCTCCCATTGGCTGATGTTGGCGCGAAGGTGCGCGAGTCAGCC
CTCGCGCTGGGGGCGCAGGAAACAATA

>DHS190
AAAATGGCGCCTTTATCAGCTCGGCGCACGGATATGGGGGGGAGACAGAGGAAGAGA
AGCGGAAGGAAACAGCGATTCGATCCCAACACGCACGCGCGGGAGCGCAACGGGTGC
TGCGAGGGCTACGTTCAGCGCACGCGCACGCACGCGCGACGTCACCCTCCCCACGAA
GGGGGTAAAAAAGTCCCCCGCGTTCCCAGCTTGGGGTGGTTTGCGAACAGAGTGAC
>DHS191
TATGCAGCATGTGTGAAAAGTCTGTTTCTGTTACAAAGCAGAATAATTTAAAAGTGC
ATGCTCTTTTCTGTGAAGTCCAGCCTATATATAAATCAATAGAGCAGAAATGTAGGA
AGAGACGTAAATTATATTTTGTCAAAAGGTCAACCTCTATTGGTCTGACCAGGAATA
TTATATCAGCATCTATGAACGCCATTGTTTTGGGGCTTGGTTTTGCTCAGATATTG
>DHS192
CTCCAGCATCCTGTTGATAGTGACAGTAACCTCTTTTCCCATTTGGCAGAGGAGGAG
ACTGATAGAGAGAGGGTGAGTCACCCCCAGGCATGATGGAGAGTGAGTCAGAGAAGG
AAGGTGGGGAGTGCTCTGGGGCTGCCTGTCCGGCCCGGGCCCTCTTGGCTCATGACT
CTTGCGTATGAAGGCTTTACAAGCACAGCAGCCACTTGTTCTTTAGCTCCTTCCTGG
GA
>DHS193
CTTTTCTGGGTAAAAGCGAGTCAGAGTTGAAGGGGGCAACAGTGTTTGCGTTCGGCC
TCAGAGGTGACGCTTCTTTGGGGGCGGGCTGGAGTTTTCTGGCCGTGAATGGCAGAG
CGCTAATGGCCGCCTCCAATGTGGCCCAATCAGAAAGAAAGGAAGGCTGGGAACTAA

GAAGCTATTGGTTGGTGATCCCTGGACCAATCGGAGGAGCCGTGATTTGGCGGGAGT
CTTGAC
>DHS194
CCCCTTTCCTAAACGTAGTTCCTCTGATTACCCTGTCCAACGGACGTCGCTATGATC
CAATTAAGTTTTGAGGCTCAGAGTGACGCCCTCTAACCTCACACTGTCCAATAAAAA
AAGGAGTTTCAGATTAGCACCAGTTTTGACCAATGGATGAGAAGATCAGCGTGTATT
AACCTGTAGAAGCAGCACAGAGAATGATGAGCAGCCAGTTCACCCAATGGACCTGCC
TACTGCAGCGTAG
>DHS195
TCAGTATTAGCCAAGCAGCAAAGTTTTGCTGCTGTTTATTTTTGTAGCTCTTACTAT
ATTCTACTTTTACCATTGAAAATATTGAGGAAGTTATTTATATTTCTATTTTTTATA
TATTATATATTTTATGTATTTTAATATTACTATTACACATAATTATTTTTTATATAT
ATGAAGTACCAATGACTTCCTTTTCCAGAGCAATAATGAAATTTCACAGTATGAAAA
TGGAAGAAATCAATAAAATTATACGTGACCTGTGGCGAAGTACCTAT
>DHS196
AGTGCTGGCTGACTGTCTTCACTCAGCGCTGTAATCGGCACATCTGCAGACAGGAAA
TCCTCCTCACTGAAGTTCCCAGTCTATTATCTGAGACACCTGGTGTGGGCGGGTGAT
TCAGAGACAAACAATGAGCGGGGGCCCCCTGCACAGCAATTAACTGCTTTAACTGCT
AATATGTTTAATCCACCACGTGGAGATCAAGGCTGCGTCGAGGAAGGGGCTCTGGTC
CTCCTCTGCTTCCAGACTG
>DHS197
TGGGACCGGAAAGAGTTTATAGATTTCCCGTCTACCCTACCTCTGAGGTGAAGGTGG
GACTGCCCTGTGGAGCCCACCCTTTCCGTTATGCGCCCGCGCGGCGCAATGACGTAA
CACAGGCCCGCCCACTGCCCCTGTTGGGTTCCTGAGTCGTGCTGCGTCGACAACGGT
AGTGACGCGTATTGCCTGGAGGATGGCGGACGCCGGCATTCGCCGCGTGGTTCCCAG
CGACCTGTATCCCCTCGTGCTC
>DHS198
GATTCTGAGAAGAGAGGTCCCTGAGAGCAAGGATGTGTATCCTGGACACTAGAAAAA
TGTATCCCCCTCCATAAATTACTAGCTTGATTTTTCTAGGATTTGTCACTCTGGCAG
AAAATATCCATTCTCCCCGTCTTCTTTAGTTAGGCAGCCTGACTTTAATTATGGCAG
CCATGTGCTCAGCGAAAAGACTACATTTCCCAGTAGCCCTTGCGGCAGGGACTGGCC
AATGGAATGTG
>DHS199
TTGGTCATCATGGGATTGAAGTAGAGGCTCTCGTGGACGCTGGCCATGGCGGCCCGA
GACTAAGTCTCGGGCCCGCGGCGCGCTGCCGCCAGTTGTCACCACAAGGCACAACAC
TACCGGGTCTACATCTCCGCCCGCCCTCACAATATCCCAGGAAACAGACATCCGCGT
GCTCGCGGCATTTGTTAACCAATCAGAGCAGTCTGTCAGAGCACATGGGAGAGCGAC
AGCCAGTCAGAAT
>DHS200
CCCGTTGGTAGTAAAAGAACCAATTAGACTGGGAAAAAAAGAAGCAACGCCCCAGGT
TTCACCCCGGCTTGACAGACGCAATTGAGGCCATCCTTGAGTCCCTCCGCCCCCAAG
TCCAGTAATGCCAGCGATGGACAGACCTTCTACCCAATAGCACGCCCCTTTTCTGCC
GTTGGGTCCCGCCCCCTTGAGGGCTAAGCCAATGAGCGCTCCGGGTCTCAGCGGGGT
GGAGGGGTTGCACTGCGGTAATATGGC

>DHS201
TTGGCTCATTTGGCATATCTCCATGCTAAATTTAAAAGTAAATGTCAAACACGATTA
TTATATATGTGGAAATTCACCTCCCAGGGACTTATGTTCCTCATCTGGAAAATGAGG
GGGTGGACCAGATGCTCCCTAAAGGGCTTTCTAGCATTTCTGTTCTCTGGTTTATGT
ATTTTGGGCATTGCATTTCACTTTTTGTTTACTACAAACTGAAAAGAGTGAAAAAGG
ATAATATCCAGCCTCCAATTCCCAATCATGTTACAG
>DHS202
TACTGTAGTTTCTCCACGCAGAGCTGCTGTGAGGATTAAAAGAGGCCGCGTGAGTTG
AGCATCTCGTCGCCATTACCGTCATCACACGCCCCCCACCCAACCCGGCGGGCCCAC
CCTCTGCACCCACGGCGAAAGCACTCTTTCTAAACCACGCCCCGGATGTCCCGCCTC
CAGCGGCTCGGAGGTCTTCACTTATTGGCCTCCCCAAACCAACATGGCGGCCCCCTA
CAAACTTCAAGCGCC
>DHS203
GTAGTGAAGGCCGTAAAGGTTGAAGAACCAAAGCATGCTTCTCCATCTCGGCCTTTA
AGGTCCGGGTGCATTATGTAGCGTGGGGTTCAAGAGTTCATCTTGCATGACCAAGGG
AATCTAAAATTTTGACTCCTACTTGATATTTAAGCTCGCGGGAAATGTGTAGTAGTG
CCACACTGTGAAACGGTCCCATTTGAATGATAACTGGAAGGCGTGGTCTGGTTTGGA
GAATTGGTGA
>DHS204
AAATTCTCATCTCCTGGCTCTCCACACCTGCCACCCTGATGGCCCCTGTGCTGCGTT
TGATGCCGCCTCTCCTGGAGAATGACCATGGTGATCATTCCTGTCATCCTCTGAAAT
CAAAAGAGAGAACGTGGGCACTTTCTTAAAAGCCTGAAGGGAATGTAGACATTCCGA
CATCTGGTAAGGGAGACGCTCTCCATCGCTTCCCCCGGGGGCGTCACCGCCCCCTGA
TGCCCCGCCCTCCTCACGACTCAAGTTCCTCC
>DHS205
GCCACCTTATCAATGGATTCTGAAATTATGGATAAGGGATTATAATCATGTAAAATA
GTTTTAATTAACTTTAGGAAGTTTTATCATCTTTCTTCCTCAGCTATTTCTAGGAAT
GAAATCAAGCAGAACCTCAAAGGTCATAAATTGAGAAACTTAATATAGAAACTAAAA
ATGTCTAATTGGCTGCTAGCTTGTGCTTCAGTTAGTTAACAATATTTAACTTCCGCG
TGTCATACATGCTCTACAGAAGGGG
>DHS206
CTGTCTGAGACGGACTTAAACCTGCACCAGCTCCAGAGCCCTGCGGAAACAGAGATC
GGTCCCCTCCGGCGTCTAGGTAAGGCCCACCCTCCCAGACCGCAAAGAGCCAGGATC
CGACGGCAGCCTCTCCGGACCAATCACAACCTTCCTTCATCAAGGAGGCGGGGTCGG
GGGGGGCTGACTTTTCCTTGTCTCCGCCCCTTTAGTCGAAGTAACCAATCTCAGTGC
ACCTAGGGGAAGTAATCGCAATGGGAG

>DHS207
CTGAGCGAGCATGAGGGAAGGGCTAGCCACAGAGATCATGGACCTAATGCGGGCGCT
GAAAATAGGGATTAGGGCAGGATTCCGGTTAGAAAGCGCCAGTGTTAAAGGCTAAGG
AGACACAGCGCCCCCTAAAGAGGGTGCGGTTTAAAAATCGCTAAATCAGTCCGACAA
TATCACGTGTCCTCGGCCCTACCAATTGAAAATTAACAATCGCTTCTTCTGTTCCCC
ATGGAAAC

>DHS208
GGTCTGGTGACCTGGTTACATTTTATTATACACATTATTGGACCATAAGGACATTTG
TTTCACCTAGATTTTAAGATTATGGAGACTGCTGTCATTTTTATCTTATTTAAATCT
CTAGGTTTATTGGAAGAGTAAGATTGATGAACTATAGCATGCACAGTTTGGTACAGT
AGAGATCATTAATACTTTTAAAAGTTCTGCATTAATTGACTTGGAATCCTTAGAAAT
GGAGTGGTGACATG
>DHS209
GTTTCTTTATCTCCCTGGGCATGTGGCTCAAGGTTCCAGTGACCGTCCTTCAACCTG
CCGCAAACTTATCTGTGTTGAGCAGCTTCATGTGCAATTGAATTAAAACATCATTTC
CTCTCTTTTCCCAAGGGGTGTTTATCATCCTTTCTGGGAGAGTGACCATTTTGTTCT
TATCTCTCAGAAGAAAATTGTCTTTCCTCACTAACACAGGTTCTAAGAACCTGACCT
TGGTGATGACAGTCAGTGCTACAT
>DHS210
AAACCCTTCAAATTAGCCTGGACTCGCCTGCATCTGGCTCGGAACAGAGTTAATCTC
AGGAGTGAGAATATAGCCCTCCGGTGGACGCAACTCGCGCCTGCGGCTAACAGAATG
TCTCCAGGGGAGGGGCTCTGCGTCCGTTCCGCCCAGGCCCTTCCGGCTCACGCGCCG
TACGGAAGCGTGCTCAGCGCTGGGCTTGTGCTCCGTACGGAAGTGTGCTTTGGCGCA
CCGGAAGCCGACTCAACAGAGCTAT
>DHS211
ATGAAACGCGAGTGCCAGATTCGCGGGGCGGCTTCAGAATTAGATTGACGGTTCTGA
AGAGTCACGTGGACTGTAAGGCCGGGCCTAGTGGAAGACTCCTATGGACCGGCTGGG
TGGGCGTTAGGTTTGATAGTCGTGGACACAAGCCAATGAATGCACGTGAGAGGTGGT
GCTTGTAAAGATGGGCAGCCAATTAATCCAATTCCTGACGAGTTCTGGGTGATGAGG
TTGGCTTTGGACTTTAATTG
>DHS212
GTTCGGAGACTGGACGAGTAGGGGCGGGGCTGATACCCGAGATGGGCCAGGTCCCCC
ATAGAAGGCAGATTAATAATACCCATCTCCAGGAGCCGGGCCCAGAGACGACGGGGC
GGGGCCAGGAAGGGGCGGGGCCTTGGGAGGGTGCTGTTTCCCGCCAAAGGAACTGGG
TTGTGGGTTGGAGCCCTTAAACCACAGGGGTTCGTTCCCTGGTGCGAGGGTCGCTAG
AAAGCTCCGGTGGTC
>DHS213
ACTGCAGGTACAGAGCCTCCTCTTCCCGCAAAATGGCGGCAGCTCTGCCGTCTTCCA
GAACCTTCCCCAATCTGCGCATGCGCCAAGACTCCCCGGCTTTGGAAATAACCTCAC
TGGTTTCGCGGCAACCGTCCAAAGTACAGGTTCTCATTGGCTAAAGTCCTAAGTATG
GGTGGAGCAAACTCAAATGCGTCTTAGCAACCACCGAGTTCCCATTGATTGGCTAAC
CTCAGAGACTCCAGAAAATGG
>DHS214
CGTCAGGTGGAAAACCCTATAAATGAAAGGCCCTATGCATAGGTATTAAATCAGGGC
TTAAGGACGTTGAGAAACCTCGAGGCCAACCCCCACACTATTTGGATGGGATTGAAG
GGGAAGAGCGGAGTAAGATCAGACGGCAGTAGGACTTCAGCTCCCCGCCATCGCTGT
GTCCTGTGGTTGAAACGATGGAGGTCGCCCCATCCCTGTCCTAAGGGAGGCAGTAGG
>DHS215
ACCTCGCCTCTGAAGAACTACAATCCCCAGCAAGCTTTGCGCCTGGGTTAGCGATCT
AGCTTGCTTTGTTGCCCGCCTAGTTTAAGATTGGGAGTCGCATTTCCAATGCGCAGT

ACCCTGGGATTTGTGGTCTCCCTAGATTAAACAGCCAGAGACACAGGTTGATGGTCA
TGTTGCATGCCTGGATCTGTAGTCCCTTTGAAGGAGTTCGCAGTGCATGCTGAG
>DHS216
GGCACAAGATTAGCATGTTACCTAAGAGAGCTTTTCTCAATGCTAGATGGCTGCAAA
TTGTTTATAGATAAAGTAGCAGGAGAAAAATTCAACAAAATCCTGTTTCTCAGTGGG
TCATTATTTTTTTAGTTATGCTTTTAGGCATGAGCTAATCTCTGATTACCTCAACAC
TGACAATAAAACTGATTTATAAATAATCACAATGGAACCATTCAGTATTGGAGCT
>DHS217
CACTGTCCCTGAGATCTGCTCTTCAAGAGGAAGCCTAAAGTGGAGAGTTTGTAATCC
TGACAACGCCCAGCTACCTGCGTACCCCGGACTCCAACTCCCAGTTTGCAACGCCAA
AGGGTCTAAAGGGCGAACGGGGGCGCATTGCATCCTGGGAGCAAGAATCGCGATAGG
TCTGATGGATTGTGGGAACTGTAGTTCCATGTGCGTGCGATGCACTCTATGGTAGGA
TAGACCCGG
>DHS218
TGCTCTTATAGCAGGCGGAAGCGTCACCCGGGCCAGCAGCCGCGGGCGGGGCGGCGG
CCCTTCCCGGTGACTGCGTAGGAGTCCGAGCTCCCGAGGTCGCCTAAGTGCAAGCAG
AAGTGAGCGTGGCTTCCGGGCAGCGCCCTCCTCAGGGGCGCGGCTTCTTTCTCAGTT
CAAAGCCTTTCCCCGAATCTCTGGCAAGGACCTGAAAAGCTTTTTCTTACTAAGGTG
TGAATGACTGAGACGACAT
>DHS219
ACTTTGCAGTCTCGGGTGTTCCTGCCCGGTCTTCTGTTCCTAAACTGCAGCAAAGGG
AAAAGGAACTGAAAAAGGCAGGCTCGTCGTTGCAATATCACCAAAAGAGAAAATTAA
CGGCATGCCATCAGGACCACAGCAGTTGGAGAAACAACTCTTTATCCCGGCTTGCAG
CCACGAGGTCTTGATTGGGGGAGGGGTGGTGAAGAATAGTCTGTGGGCTGCTTT
>DHS220
GGGAACTGTGTAGGGGTAGATTTTCGCTGCAGTGTTCCCCGAGCCTGTTAGACGCAG
CGCGCCGGGAGACTGAGAGAGGAAAGGATAGAGGAAGTGCTGCCCTAGGCTGCATGA
GTCGAAGCAAGCGTGTTTCCTTCCCGCCAGGCAAGTGCCCTTAGAAACCGGGCCCCG
CCCCCTTCCTGGCCTGCATTCCCATCCCCTCTCCCGGGGCGGAGGTGAGGACCTCCT
TGGTTC
>DHS221
GGAAGGTCAAGGTCTCCAAAATGTTGAGTCTTCTACTGAGGTTAGAGCTAGCAGCTC
CAGCAGTGTGGGCATATCTGCATGAAAAGGCAGAGAACAACCTAATGAGATTGACAA
GCAGTGAATCTGTTTGAGACCACATTGAGCCTGGCCATCCAGCACCAAGATGGCTTG
AAATAGCTCTCAGAGAGGCCAGAAATGCTGTCTTTCAAACATGTTCCCTGGAGAAAG
GGAATTGTTCCTGCTAC
>DHS222
ATATGCAAATTAGCTGCTCCAGTGCCGAAAGCTCCGCCCCATTTGTAGTTGCGTCGG
CTGTCCAGCACCCAGTGCAAACGAGGCGTGGGTCCCGGAAGCTCTGTTCTGCGGGTG
GCCGCTCGCGCCTGCGCAGTGGAGGCGGCCCAGGCCCGCCTTCCGCAGGGTGTCGCC
GCTGTGCCGCTAGCGGTGCCCCGCCTGCTGCGGTGGCACCAGCCAGGAGGCGGAGTG
GAAGTGGCCGTGGGGCGGGTATGGGAC

>DHS223

TAATACATTGGTGCTCCCTCCAGTGGCTCAGGGAGAAGGAGGAGCACTATCCTTGAT
ATGGAATTGGAATATTTAAGAAGTGTATGTTGATCCTAGCCACGACTCAGATAGAAG
GAAAGCTCAGAATAAGTTAATCTTTTGGATAAAAGAAGAAGATTACCTCACTTTTGA
CTACAGACAGCTCAATTTGGAGATTTTACAAAGAACAGGAAATAACATATTTTGCCT
CATGTTCATGCC
>DHS224
ATTGACTCCTCGCTATGTACCACACTCTTTCTCGGCAGCGACCTGTTGGCAGGGTCA
CAACTCCTTGGGGTCTTGTCTTCAGTCTGGCAAGTCATAACTTCTTTCAGTTTTTTA
AGAATAATCAAGTTCTGAGAGAAGATATGTTAACAAAATACAGCAGGGTGGTTTATG
AGTAAAAGAGGTGATTTACTAATTACACTGAGAAATGTACCTGTCAGTAGACCA
>DHS225
CTTAGGGTGGAGTTAGTGCGTTCTTAGGGCCAGTAGGAGGCGAGTCTGAGTAGAAAC
TGATCCAGGTAGCCTCGGAAGGGATCACCTGCTGGATGGGTGTGAGCTCCTCAGGGT
GGGGCGCCGAAACCCTCATGGCTCCTGAGGTAGAGCCGGCCTCCTTGGTAACACATG
GGACTGGGACTTTTAATACCACCCGACAGGCCCAGCTTGTCTCGTCAAGCGCTAAGT
AAAT
>DHS226
GGGGAAGTTAGAAAATCGGTATCACACGAAAACAGTCATTTCCCCTTTAAGGACCTC
AGTGGGCCGGGAGCCAGCCTTCCCGCACCTTTTCCAAAATTTCCAATCCCCGCTTGG
CATTTTCTGCCCGAAGGTCTCCGATTGGACGCTCTGTACCAATCGGAGGCTGGAGCG
GGCCCTGAGAGGCGCCCGACTCCGCCCCCGCACTGGCCCTGCCCACTGCCCGGTCAG
CCTCGTTGCTATGGGGACTGGT
>DHS227
ACTACCGATCCCAGTGTGCAAAGCCTCCAGGCTTACCTTCCCGGAAACTGGCGCGGT
GCCCTCTGGGACTTGTTCTGGGACTTGTAGTCTTGTACTGGCGCTGGCCCGCCTTTC
TTCCTTAGTGGGGAAAACCGGGTGTAAGCCCCGGAAGTACTTGTTGCCTGAGCAGTG
GGCTGCTTAGGAAGAGAAGGTCAGAGTTCGCGGGGGCAGAGGCATTCTTGCCGCTGG
CCCAGTCACTATGTAGTGGAGGGGCAGACAC
>DHS228
CCAGTTTCATATTTCCTTGTGGGTAAGAGGCAACCTCCTCATGCTGTCATACCAAAG
TCAATCTCCACTACACTTTAGTTCTGCCTGTTCTTGATCTTTATATAAATGGAAACT
TGCAGTATATATTATATGTTTTTGTGTCTAGAAGTTTGAAATCAAGGTGTCAGCAGA
GCCATTCTCTTTCTCACAGCTCTAGGGGAGAATCCTTCCTTGCCTTCTAATCCTTCC
TTGACCAAA
>DHS229
AAGCAAATATCATTGCTGAGTGCTGTAAAAACACAAACTCAGCTGACAAGCCCACAC
ACCTTTATTTCTGCAGGGGTATCATGACTTCCCTGAAGTCATCCTTACTCTATCTAG
GTACAGAGTGTCAGCCTGGTGATAAGTGTGTACAGCAGGAATTCAAGACAGTTCAGA
AAGTTATCTTGTTTTCAACAGTCTAGTTGAACAAAAGGTCACAGAAAGGTGGCC
>DHS230
GCCAAGGCCAGAATAAGATCCCGGATGCCAGGAGAGCCTACAGTTCACGACCGTGGG
TGATGCTTTACGTTTAGATTCTCCGCCGGAGTCCCAGAGCTCGGTGTTCAGGATGAT
GAGAAGGCGTGGAACGAGGACTTCAAATCCCAGAGTGCAGTTCTGCCTGAGGCTTCT
GCATGCTGGGAACTGAAGTCCCTGAGAAGAGGAGGTAAGGCGCTCCCGGCATGCAAC

GCGTAAGACTGCGACGGTAC
>DHS231
CTGTGCAAGCTAATCATCAAGCCTTAAAAACATCACACATATACACACACAAATACG
CTAACCAGTATTTTTGTCCCTTGAGATATATATATTTTCTCACAAATGTAAAGAAGC
CTACACAGCTGTTTCCTTTAAAGATAAAAAGGGAAAAAAATTATTCTGCTGCAAAAT
ACAACTAAAACAATTAGAATTAAAAAATGTTCACAACAAATATTTTTCAAAGGAATG
TCCCAAAGACCT
>DHS232
ACCGCACCCCTACCTACTTGACCCCGCTTCTCCCGCTCGCTGTCGTCCAAACACTTC
CTTTTTTCCTTCAAAGGTCGCCGCTCCTGCCTCACCTCACATCCGTTTGTGGCTGCC
GAGTGCTTCCTGGGAATTGTAGTTTCTAAGTGGAACCGCCCCTCCCCGCCCTTTCCT
GTCCACTCTGGTCCCCTCGTTCTAGGAGCTTTATTTTACTCTTTCCACTTACCCATC
C
>DHS233
TGTATGGCTTTTCAGAGAAGGTGTAGGAAGATTCAGATTCAGGTGGCCACCAATAGC
CTATGAGAACCTACACATTCTTAAAAATATGATACAACTTGAATTCTAAGTTCTAAA
ACTGAATTGAGTGGGGAATGTTTTAAGAATTATGAATTTCTTTACAAATGTTTTCAA
GTAAGAAACATTAAACCAAAGTATTTATTTCAAAACTACTGTAAGTTCTATGTTGAC
TGTGGTGGTGGCTACAAATCATTA
>DHS234
GTGGGCTGCTGTGACTGAGCCCGAGTACTTGAGCAGAGCACCGTCCACAGCCTGCAA
TTTCAGAGTTACCTTCTATGGAATTGGAATTAAATGCCCAGTTAATACAAAGGAAAA
TTTCCCTTGTGCAAAATCTAATCAAATGTAATATGTCTATATTATAACTACCATTTC
TATGACTATTATAATTCAAGTTCCTAATTATGAGTTCAGTTCATAAAAAAGAAAATT
GGCTTTCATTTGAGCAGCATTTTCAGTC
>DHS235
AAAAGCAGCTATGAGCGGAGACAGCCAAATCTATGCCAGCCTCGGCAAACGCCGATT
CGTCCGCAGTCACTTCCGGCAGTCGCTCTAAAAAGTACAGGAAGTCCTGCTTCTCTC
TGACAGGTGGGGAATTTAAAAGGAGCGCGTGCGCAGAAGAGGCAAAATTCGGGCCTT
AACGAGAGGGTGCCCCCTTCAGCGCATGCGCAAGGAGGCCCGCTGAACGCTCACCTG
GACTGTGGTATTTAC
>DHS236
CCCAGCTTTACCCTACAGACGCCTCTAGGGTCGCTGCCTGATCGCCCAGTTTAAGGG
AGACCCGTCCTAAGGCCCACCCTCTGCCACCGCCTCCACTCCGGGAACCAATCACAG
CGGACGCCGGGAATACTTGCCTGCTCTTCCATTGGTCGTGGGTCACACGACGTGAAC
TGGGGCGGTGGCAGCACGCCAGGCCTCTGACGTCCCAGGGCTCAAGCGCGGAGGACA
TGTGGAACTCCACTCC
>DHS237
GCCCGGTACGTAGGAATAAATCTCGCTTCTCTCGCGAGAGTTGCGGGCTCCGGGCGC
GTGCACGAGAAGGGGCGGAGTGAAGCGTACTCTCAACACGCTTGCGCAGCAGGGCTA
AGTGACGCCAAGCAGTTTGCTGCACTTAACCACAACCAACATGGTGGCTGCATTACA
TTACATTCCTTCTTGGGTGTGCTGCGGGAAGCAATCTCGGTGTTTACTGACTCC
>DHS238
GTTTGGTTTTCAGGCCCTCTTCCTGGAGCTGGCTGAGCAGGGTTTCCAGGCATCCAG

CAGGTGGCACTGTCATCTGGGCTGGAGCATGCAGCCAGCAGTTCCCTTTTGCAACTC
AGTTGCCTTGGCTAAGGCCCAACCAGTCCCAAGTGCTCAGGACAGAGGCTGGACGGG
CTCCTTCCTGCACCCCTGCTCTTGGGACACAGTGCCCTCCGGCTCTAATAGGCCAGG
AGTTGGG
>DHS239
CTGCTAGAGGGCAGAAAAGGAGGCCTGGCCCTTTAAGAGCCAAGCTAATGGCAGCTG
GCTGAGGGGCTAAGAAAACAGCTTAAGGTTTTGTCCCGTTTCCTTCAGAGTCCCCCA
ACCCTGAAGGAGTTTGCGCAAAAATACCCTAAAGTTTTCCAGGAGGTGGGGAGGTCA
CCCCAGACCCCCCAAAGAGCTGAGCCTTCCAGCAAGGGATAGTGGCTGGGAGGGTC
>DHS240
CGTTCATGTCCCGACTGAAAATGGGCTTCACGAAGGTGCTGGCGGTAGAAGTAGCTT
GGCGGACGCCGCTCCCCGCCATCTTGCCAAAGCATCCACTCCACAACCCCACCCCTT
TGCAAGCAGCGCGTGCGGACCGCGGGCGAATGTCTTTTCCCATTGGCTAAGGAGGAA
CGCCCCGCCTGTATGCTGGGTGGGCGGGAGGATGACTCTGAGGCTCTCGGATTGGCT
GGGAAGCCTCTCT
>DHS241
CCACTGGCCAACCTATTCAGTTGCCAGGCGACATCGTTGCCAGGCGACTCCAGCAGC
GTCCGCGGCGCCGCGGTCGACATAATCAGCACCCACTGCGCAGGCGCAAACAGCCCA
AGGCTGCGGCCGGCTGCAAAGCGGGAACAATCATTTACAGGGTTGCTGTGGAGACCA
AAGGCAGCTCAGCTTTGTTCAAAGTACAGAAGGGAAAGGAAGTGACTTATTTACGCC
CTTCTCTGTACTGGGTAC
>DHS242
ACCTGCAGCCTCTTTCTCTCCAGCTTGGGCAGAGTCTTGTCATCTGTTACCTCCTCC
ACCAGGCGCCGCACTTGTGCCCCAAAGTGTAGCTCCACCTCATCCAGGGTGGTGTCT
GTGTCCTCCACCGTGTCATGGAGCAGGGCCGCCTGGGGACAAGTTCCCACTCAGCCC
TGAGATGTCAGCCAAGGGTTGCCCATTGCTGAATGGGGCCCCTAATCCCCATCCCAG
GCTC
>DHS243
GAACTAAGCTCCTCTCAACCCTTGCAATTTGTATGTTGGACTCCTAAGATATAAATT
ACAGTTCTCCCACTGAGAATAGAGAATTTATCCTCTCTCTCTCTGTGTGTGTGTGTG
TGTGTGTGTGTGTGTGTGTGTGTGTGTGTGTGTCCTGTTCTTCAATGTCCAATAATT
GTGTCCAGGGAAAATAGAGAAGTACAAATGTCAGACTCTCAAAAAATAACAGAGTAA
GGGTTCTAGGTTGTAAATGCCTCAG
>DHS244
ACTCTGCAGATCAGGTGGGTTTGGCTGTGAGCTTCTCTGGGTCCCCAGTGGTCCAGT
GGCTGCAAGTCCCCGTGGGCTCTGGCCACCAGAGGGCAGCATGCTCCCCACCCACCC
TCATCACACTCACTTCTCCCCTGGCTCTGGAGGAGCTAAGACTCCATGCAGCGCGTC
CCCCGCCCTTTGCCCAAACTGCACGATCAAGTCTGAGAGAATACAGGAGATAACTCA
TCATCCTCGATTAGGCA
>DHS245
CTTTAACCTGACGTCACTCCCTCTCCCAAGGGGGTGTGTCTTGGAAGGCCGACAGCA
GAGACCCAGCCCTTCGCCGCGCCTGCGCAGAACGGCCGAAGTGTGCACGCCCCTCTT
TGGCGGCCTCAGGGCCTAGAACAATGAATGAGTCAACGCGCCTGCGCGCCACGAGAG
CCTACCCACGCATGCGCAAAAGGCTTAGCTAGCTAAGCCGCGGGAGGCTGAACGTCA

TCAAACGCGCCATAGG
>DHS246
TATCCCAATCATCTCAGAACAAGCTGTTACTAATGTACTGGAGTTTCTGTGCAAACT
GTCTACCATAAACCATGAAAGGATTCAAAGTTCATAGTTCCTTCTTTGTTCCTTTGT
TAATCACTGACTTCTGACTAGTGGGAGGTGCCTCCCAAGTTTGCTAATGCATTCTTT
TTGGATAAGGATGACGCACAGATTGTCCTAATAAGGACTTAGATTGAGAAAGACCGC
CCCCTCTGAGAAGAGGGGACAAGTCAGAGAG
>DHS247
TAAGCCATAGAACCCCAGAATCAGGAAATGTTTGCTAAACGAGGGGCCTGGCAGTTG
GCTTCTTCTTGGAAAGTGGGGTTCCCTCCTTCCCCCATGGCTTGTCATTGGATGCAA
GTGGCTGGCCTTTTGTGGGCCTGTCTGCGGTAAGTGCTTTGGTCTAGAAGAGTCAAC
TTGTGGTTTCACATCTGGCCGGGCTGACCAGATGTTGCAAGGATACAGAAAGTGGAC
ACTCAATCCCCTGGAGGACACAATCTGAGGCACATTA

II. 710 non-TIS sequence segments
>non-DHS1
GGCTCAGCACACACTTCACTGCTGCCGTCCCACACAGGAGCTCCAGGCTCGGATCAA
CCCAGCAGCAGGAAAGTCTTCTTCAACAGGGGCCAAGGCAGGATCCCCAAGGAGGCC
ACCTTCCAGAAACTGCTAAGGAACGAGGTCCGGGGGCCCCAAGCCCTGACTGGCACA
GTCAGAGGAAAGTCAGGGCGGAGCTCAGCCCCCGTCCCCTCCCCTCGAGAGCGGGCC
TGAGCCCCTGTCTCTCCAT
>non-DHS2
CCCGTCTGACATCTCCTGTTGCCTGGGCCGCCTCGGCCTGGACCCTGAGGCCAACCT
AGTGATGGAAAGAGAGCGAGCCCTAAAGGGGGCTGGGCTTGGTCAAGTTCAGCACTT
CCTCCGCCATCTTCTCCCCATCCCCCATCACGACCCCGGGCAGCGTCTGCCTCTCGT
CCCCGTGGTGTTTGCCATCACCAATCCGTTTGATTGATCATCTCGGTGGCCACAGGT
TGGTAATAAAGCTGGT
>non-DHS3
TTGGTAATAAAGCTGGTTTCAGAGCCTCTTGCATTTCCCCCAGGACCCCGTTGGCAT
GACAGGACTTGACACCAGCCATTCGTCACCTGCTGCCTGTGGTCTCTGCACAATGAC
CAGCAGACAGACAAGAGCCAGGCCAGGAGGAGGCTTCGGGAGAGGCTGGGCCTGTGC
TCAGGGCTGAGTGAGACTAGTGGACGTGGCCGGCCCCATGCCTCTCTGTGCACGGCT
GGATCTTGCAAACAGTCGAGTC
>non-DHS4
ATCTTGCAAACAGTCGAGTCCTGGCTGCTGCGCCACCCGGGGATGGCATCGTCTGTC
ACACGCTTCACTCTCCCTGTGGCGCACTGGCATCCACCATGCCCACCTCAGAGGCCA
GGAGGCACGTCAGCCGGGAAGGCTGCAGGGAACCCCCAAGCCCAGGCCACCAGGGCC
CTACCCTGCCATGGGGCGCACTTGCTCAGGCCTCTCCTCACCACAGGGTCTCTGGGT
GCCTCTGTGAAGACAGGATCCCAGTA
>non-DHS5
AGACAGGATCCCAGTACAGGAAAGCCCCAGGCTGGCCCCGACCCTCTCCTTTTCTGG
GAGGAAAAATGGGTTTATGTTGTGAATCCGCTGAAGCCCCTGGGACGTTGTCCAGGG
ACAGGGTCAGTGAAAATGTCTCAAAGGTCGGGGACTGGGCACAACGAGGCATTGACC
CCAGATGAATCCACAGAGCTCCCGGAGCAGGGGAAGTAGCCCTTGTTCACCCACATG

CACAC
>non-DHS6
GTGACAACAAAGCAAAGCCAAGTTGGTTCGCGTGTGACTTTGCGGGCGTGGTGAGAA
CAGCTAGGCGTCTTCATGCTGCTGGGTGTCAAACTGAAACTCTCCAGCTTTAAGGAA
ACCAGATGCTTTTCTTGCTCTCAGAGTGATGTGCAGGCTGCCCTGAGCATGCACACA
CACATGCACACCCAGCAACACCTTCACCTTCAGACACGCTTTTCTCTAGCGCAGCGT
CCCAGCTCCTTAGCAGAGGGCT
>non-DHS7
CTTCTCACACGTGGGTCAGCTTTGCTGCGGGGGTGGGGGTTGCAACTCTGGAGGCTC
ATTTTATCGCCGGGCCAGGGCAGTGATCTGCTCGGTGAGAAAAGAAAAGGCCATTCC
CTGCCTTTCTGCTGGGCTCCTGGAGACTCCTGGAGACTCCCGGGCATGGGCAGGCTG
GGTGGGCTGGGAGCCCCGCTGATGCCCGGAGGGTGGGCTGAGGTCTGGACGCCGACT
TGCCTTCCTACTTGAGCC
>non-DHS8
CCGACTTGCCTTCCTACTTGAGCCCAAACAGCCAGGTTTCTGGGTGGGCAGAGGCTG
TGTGCAGACTCCAGCACACAGGGCACCTCTGACTCCCGCTTCGCTTTCCTCCCAGCA
GATCTCCGAAGACCTGGGCAGTGAGAAGTTCTGCGTGGATGCAAATCAGGCGGGGGC
TGGCAGCTGGCTCAAGTACATCCGTGTGGCGTGCTCCTGCGATGACCAGAACCTCAC
CATGTGTCAGATCAGTGAGCAGGTAGG

>non-DHS9
CAAATGTTGGTTTCTCACTCCAGCTAGTTTTATGGAGCCGATCCGGCCCCCAAGGAG
GACTGGAAGAGAAATACTGCTTTTGATTTCCTGTCTCTCCTGGCGTCTTGCTGCCCA
GGGCCGCTTCAAGGCCGCATAGCTGCTTCCGTGACAGGAGATCCCCCTTTTGTGGAG
TGCGCCCCTGTGCTGGTGTTGGGGATACAGGACCCCACCGACGTCCTGGGCCCTGAC
TGCTATGCAGAGCTGATG
>non-DHS10
GTCTTACCAGGAGCAAGATGGAAGCATCCAGAATCCCACTTGGTCCCCCACCCCTCT
TTCCAAGTGGCCTGGAGGGGCAGATAACCCTGAGGCTCTGACGCTGCCAGGCGGCCG
GCACTCCTAGCGCACTCACACTTCACCCACGGTGCAAAGAATTCCCAGCCTGGTAAC
AATGCCGCTTCGCACATCAGGAGAAGATTCCGAGAGCCTCAGGATGGCTCTAAAGTG
CCACTTTGTATGTCACTGAGCACCTTG

>non-DHS11
CTTTGTATGTCACTGAGCACCTTGGAGATTTCCTGTGAGGCCAGTTACAGGGGTAAA
GGACATGCCACCCCACACGCTCTGTATTTCCTGATGATGGAGTCACTGGCCGGGCTG
GAATGGCGCCTCTCCCTACCCCTCATTGACAATGTCTGTCCCACTTGCAAGAAGAAA
GAATACGGGAAGGTCAAGCTCTCAACACCCTGGCAGTCTGCTGGACCCTCCACCCCC
GCCTTTTCCAGAGGACGTGTGATGT
>non-DHS12
GAGCCCAGGAGTTTAAGGCTAAAGTGTGCTATGATTGTGCCTGTGAATAGCCACTCC
ATTCCAGCCTGGGCAACAGAGCAAGACTGTCTCAAGCAAACCAACAGACAAACAAAG
GCAAATGGTGCACCAGGTTTGGCCATGGGAGTTTGGCGGCCCCCACTCTAGTGCTGT
GGTAGGACAATGAGCTCACAGGTGTTAATTTTCTCACTGTACTTCATACCTATCATA

TGCAGTTCATTTATTGGCTTTGG
>non-DHS13
AGAGCCCGGAATTTTCATTACAACTTTCAAAGAGAGCAAGAGGAGGAGGGAAAAAGA
TTTCACAAAAGCATTATCAAGGCCCCAACCCAGGATGCCCTGCTGTACAACCAAAAT
TTGTAAGAGGTCAGCCTTTCAGGGAGTCAAAAACCCAGCATGTGGAAACTGGCAGGC
CTTTTATATAGTGGGAGGGGTTCTTAAAACGACTACTGTTTCGCACTGAAGTCTCTG
A
>non-DHS14
TTTATAGTCCAAAGGTGGGGTAGGGTGGGGTGAGGAGAAAAGGGCTAAGAGACATCT
TACATTTAACTGCCTGTGTGGTGTCATGTTGAGCACAAATTACATCTATTATCTCGT
TTAGTGTTCACTACAACCTTTATGAAGTGCAGGCTACTAACCTCCATTTCACAGAAA
TGAATACTAAGGGTTTGTGACATGCCCCAAATCGTGTGCTCTGTTGAGCCTGTATTG
AACCTGTGTGACTCC
>non-DHS15
GTGGAGCATCTGTTCAAGATTGTTGAAGGACACATAAACCAAGTGTCCAAAAAGGAA
GTGACAGCCCCGGCAATAAACTCAATCAACAAGCATTTGTTGAACAGCTAAAGAGCT
GAGTCACTGCTTTAGACACAGATAAGGGGATGAGATAAAAATCCTGACATGACTTGG
GGCATCCATGTGACCAGTGCTGGGGAATCCCACAAGGACGGAGACCAGAGAATCAAG
ATAAGACTACCAAGGCAGCCA
>non-DHS16
TGGGCTGGTGTGTGAATATCTATAAATAAGTATATATGGTGTATATTATATGTGTAT
AAATAAAGTCTGTACATATTGGAGCTCTGGGAGATGCTGGAATAAAAGACAAGAGTT
ACATCTGGACTTGGATTGAGGTTTTGTTGTGGTTTTTTAAAGCATGATGGGAAAGAG
ATAGAGAGAGCAGCTATTGGTAGAGAGGGTTTATTCGGGACCCAGGAGCCACTCTTA
GGCTTGGT
>non-DHS17
CAGCAAGAGAACACACACACCACACATCCTCTGGGAAAATGATCTCTCAGACACACA
CTGAAATGTTTTTAATCAAAGACCGATACAGTAGGAATGCACAAGGAGTGCAACGTA
AAAAGAGGTCATCAATGTAGCTAGTGGCTTCAGTCACAGAAATAGCACGTGCCCCCA
CTGATTACAGCACCGCGGGGAGACCGTTATTAAATTATACCGTACGGCTCCGCT
>non-DHS18
CCCTGAAGATTTCCAGTGTATTCCACAGTGTTTAAAAAATAGTTTCCCCAAAAATAT
ATCTTGAAAGCATTTGACACAGTGGTATTTGTGGCGTATCTGGCGCTTGTCAGTGTG
GGGCCCAGGCCTGATGAGCCACCAGCCCAGCACCCGAATGCCTGCTGAAGGTGGCGC
ACACGTGTGCCTGGGATGTCCGGCTCTTCACAGGGGATCAGACTGCGGGTCACGTTG
AGAGCCAACACC
>non-DHS19
CTTCCTTTCGGAGGTCGAGTTCCTAGCATTTCCATCCGCTCCTCCAGCCCGCTCCCT
GCCGGTTTAGCTGAAGCTTCCCTTTCGGGGAGTTACCTAACTACCTCCCGAAGCCGT
CGTTCTGCTGCTTGGTTAATTCCTCTTATCGGGGGGGAATTTTCTTTGTTCGTTTGT
TTTTCCTCCCCTTTAAGTCCAAGATAAATGTAGTGTGGAAGATTACGTCATCGC
>non-DHS20
GCATCATTTCAACCCCTGCTTCAAAGGGTTATGATAAGGACTCGAGGAGGCTATGGA
TGTAAGGCACTTGTGTGGTGCTGGTGCAGAGCAAGCACTCAATACATGTGGGTTGTT

AGTCCTATTCGCTCCTGACATCAGCAGCTGGGACTCCGCTGACTTCATGGTGAGCCC
CAGAGACAAGAGAGATGGGATCACCCTTTCCTTGGACTGTTCCTCCCCTTCCTCC
>non-DHS21
ACCTCTCTGGCTTCCATGCTCCGCCTGTAAAATGGGGGCGCTATTGCTTCAGCTGCT
GAAGGGAGGGGGCTGGCTCTGAGAGCCCCAGGACTGGCTGCCCCGTGACACATGCTC
TAAGAAGCTCGTTTCTTAGACCTCTTCCTGGAATAAACATCTGTGTCTGTGTCTGCT
GAACATGAGCTTCAGTTGCTACTCGGAGCATTGAGAGGGAGGCCTAAGAATAATAAC
AATCCAGTGCTTAAGAGTCAGG
>non-DHS22
CTAGAGCACAGGGAGGAACTTGTCCATTAGTTTGGAATTGTGTTCTTCGTAAAGACT
GAGGCAAGCAAGTGCTGTGAAATAACATCATCTTAGTCCCTTGGTGTGTGGGGTTTT
TGTTTTTTTTTTATATTTTGAGAATAAAACTTCATATAAAATTGGCCCTCTCTTTTG
TTCCTTTGAGTTGGAGTTACATACAAGATGGGGGCATTACTCCTTTGGAAATGCCAA
GTTTATCCTATGGC
>non-DHS23
TCTGGATTTAGGGTTGCTTCTGAGGAGCTTTTAAAACTAGACACAAAAAGAAGTTCC
AGCCGATTCAGAGAAGCAGTCCAGTCAGCACAGCAGCTCGCGGTTTTCCCAGGTTCA
TCTTGACGCATCCTGAGCTACTTAACTTCGGTTCCTATCCCACTGATCGTTTTAGAG
CCTGAACAGACAAAACATCCTGGTTACCAAGACTCGAAGAATGCATAAGCTGGGACC
AGGCAAAACAAACAGATCACT
>non-DHS24
TGGACAATTTGGAGTCTGTCCATGGCAGATTGGATGTGAGAGACCCACTAGACCTAT
CCCTCTGTCTTACCAAAGTCCCACAGTGTCCCCAGGCCATACTCTTTGCCTGGACAA
TAGCAGGCACCCAGTACTGGTTTCTGGCCAAACAATGAAGTAATGAATGAATGGCGG
CAACGCTGGCTGCATGGACTTCCCCAAGGTCACACAGTGCACTGGGAGTAGTGAGTT
GGGTCTC
>non-DHS25
ACTCAGAGCACCTGTTGAGTGCCACCCCAAGCCAGCTTTCTCTTGCTTTTGTACTTT
CTCACAGATGAGTGAGTGTGAGGAGGTCTGGCCTTCCGTGCCTGTTACCACTTCCCC
TCCAGGTCCCTGGTGCCACAATGCTCTTGCCCCTGCGGTTGAGTTATCTCTTACAAC
GAACTCTGGCCAGGGTGGGGCATGGGCCTAGACCCTGGAGTCAAGGGACTTGGGTTC
TAATACAGCCTCGTT
>non-DHS26
CTGTCCAGAGCACAGATTCAGAGTCTGAGCAAATTCAACCCAAATGCTTCCGGTGCC
TCCTCCGTGCCTGGCAGTGGGCTCAAGTCCCACCAGTCACTTCCTAAATATGAACTA
ACCATATCATGATCTGTGTCGTGATTGCTGCAGCTGCGGCGGGAGCTCTCGGGAGAG
ACCTGGCAAGCCCTATCTGCCAGCCTCCACCGCCTCCTTCACGGAATTGCCTCCTTC
TTGCTGGAGCTGTTGTGAGAACCAGATGAG
>non-DHS27
CTCCTTCTTGCTGGAGCTGTTGTGAGAACCAGATGAGAACAACAGCCAGCATGCCTG
GAGAGCTTTTCTGCACCCAGCACGGTTTGGGCTCTTTTCAAGTGACGCTGCGTGTGG
CCCGGCCAGCTCACCACACTCACACGTGCCTCTCATGCCTCACACCCGCAGGGCGCC
CACACCCACTTCCACACTCACACCCGCCCACCCCCCTGCTCACAAGTGCGTCCTC
>non-DHS28

CCTCCTTTGGTCAGTGGTTTTAGGGGCACTAAAGTTCACTTTTGTGGGTAACCTGCA
CTGTTAGCTGTTGATTTAATTAACTCAGCCTTTCTCTTCCTTGACCAGAACACGGCC
AGCCGGGCTGCTCTGCAGACACCCATTTGGACAGCCGACGGCTGGAGGCGTCTGGCA
GGAGGAGCATGTGGTCCCTGGCAGCCACTCGCCAGCCCCGCTGTCGTTGACAGCTGA
GTGTTTTCT
>non-DHS29
CAAGGAAGATGACCTAAGGACCAGCTCCCAGCAGAGAAGCACAAGGCTGGAGTTAGT
ACAGCATGCCCGCATGTAAGCCCTGATCGCAGACTCTACCCTGGAGAGGTATAAGGC
ACCACTGATGATCAAGTCCCAAGGTCAGCAGGCAGCCTGTGGGGAATCCCCATGCAG
TCACCATGGGCCATCAGTGCGGAGATGGGAGCGGGTGAATGGAGGAGCTGGAGTCTG
TGCTGAGGTCTG
>non-DHS30
CTGGAGTCTGTGCTGAGGTCTGGACGAGACAGGGCAACCGTAAGTCCCACTTGGCCT
GAGACAGTCCCAGTTTCTGCCTGTAGCCTCAGTGTAGTTAACAGCAGTGTCGCCTTG
CACTCTCAGATGTCCTGGTTTGGACAGTAATTATAGGGTCATCCTAAGGTGAGGATA
TTCTCTAAAGAAGAGGGGAACTAACACTGACTGCATTCCTACCAATGCCCAGGGGCC
TTGACACAATTTCCCTCATTTAAGCC
>non-DHS31
TTAAGCCTCACAACAACCCTAAATCCTAGATGCCTCTTCCTGCACATCCAAGGTCAG
AGGGCAGGGAATAGACATTCAAAGAAAGATCTGTGGCTTTGTCCAAGTCCACCTTTC
AGGTGCTAACATGTGCAGCGGGATGAAAGAGAACTCTCCACAGTGCCTGGGGCCAGC
TCCAGCAGGAGGAGCCGGCAGGAGAGAAATCTTTAGAGTGTTCTCTTTAGCTTACCA
GCCAGATAGGGAAAGAA
>non-DHS32
CTTTAGCTTACCAGCCAGATAGGGAAAGAATTTTATCATGTATGGGGAAAAAATAAC
AAGTCATAATCATAATCATCCACTTTCAACAGTGTTTTATAATACTAAAGGGTAGAA
ACTCAAAATAGATGGCCCTTTGTTCAGCCAGACAGAGGAAAGGCCTGGATCTGCAAG
CTCAGGCCCTGAGCTTAGTGTGGAGAGACAGCAGCCCAAGGAAACAGGGGGATAAAA
TTGAGCAGCATCAAGAAACCACAG
>non-DHS33
CTGTAACCCAGACAGCTCATCTCAGGGGCCCAAGGAGAATATTCTGATGTGCAGAGT
TGGAAATCTGGATTTGTATGTGCAGTATCTTGATTCTGAATGCACTGGGCAGGCTGA
ACCAAACATCTTTGGTTTAAATTGGGCTCAAAGAGCCATTAGTTTGGCACCTCTCCT
CCCACCCTCACAAAACAACAAAACAAGCAGAAAGAAGCATGAGCAGGAAAGGGCTGT
CCTGGGGAACTACACTGG
>non-DHS34
CATCCTAGGCTCTACAGAGGGAGAGGAGGGAGCTTGGGCCAGCCCTCAGCCCACTCC
CTTCAGGGTGCCTGGAGGCTTGCACCCAGGTCTTTTCGGGTCTCAGCCTCAAACTTC
CTGAAGGGAGGAAGGAAAGGAGGGAGTGGGAAAAGAGAGAAGGAAGGAAACATTAGG
GATTGTGCCAGGCGCTTTGCGTATAAGTTCCCTCTTAATTCTCACAGTCATGAAATT
GGTGGAGGAAGACAGA
>non-DHS35
AGTGAAAGGCTTGCGCTAGATAAGCTGCATCCCTTTCCCGTCTTCCCTCTGCCGGGA
TAGGGCCACCACATGGTTGGGCAGTGCAGGGGAGGGAGTTTCCAAAGTCTAAGGCTA

GGCCATTTCCCAGGGTCTGGTCCCTCAAGGTTTTCTCCTTCCTTTCTTCCCTCCTTC
ACAGAAGCCACAGATCAAAGACTGCCCCACAGCCAACCTCAGCTTCAAGAGCTTCAG
TACCAC
>non-DHS36
GAACATCTGTTAATGCCTCTCAGGCCACTAGGTTCTAGGAACTCAGTTTGGGAAACC
CCACCCTCTAAAATATAATCCTAACTCCTCAGTAGGCATGTGAGCCCTTCCATTGTC
TGCTGTCTGCCTCCCTCCCCAGGATCATTTTTCACCTCTCTCTTATACCTCTGACAT
TCGCCCTAGAACTGTTTGTGGTTCCTTAAACATGCTGTCTTATTTCAAACTTCAGTG
CCTTTGCATATGCTAT
>non-DHS37
TTTCAAACTTCAGTGCCTTTGCATATGCTATTCCACCAGCCTAGAATGCTGTTCCCT
GCTGTCCTCTGAGTTCAACACTGCCAACTCTTTGAAATGTCCCCAGCTCCCTCAGGC
TGACAAAGTCACACCAGCCCCAATGTTCCTGCAGTGCTAGCTTTATGCTTATCAGCA
CTTCCCCAGACTATAAATGTGTTTCTCGCCTGTAAATCGTCTTGCTTCTTGCTCTCT
CCTGGCCTAATTCTTCAG
>non-DHS38
CTCTCTCCTGGCCTAATTCTTCAGCTCCTATCAATCCCATTAGATTCCCATCTCCCT
TTAATACATTTCCTCTTTTTTTAAAAAAAGTTACCAAGAATGAGTTTCTTAGTTGCA
TGAGATCAAAAAAATCCTAAATTCTACACTCAGAAAGAAGAGTTTTAAAACACTTTT
AGTCCAAAAAATGCATGTTAAAAATGGCACCCCCCCTTCCCCCCAACCCCAAGCATA
CAGGTCTCTTTTCAT
>non-DHS39
CTCCTTGATGATAATGGTAATGACTCCCCTTCTGATGCTAGCTCCTTTCATTGACAA
TGTCTTCCTTTAATGAAACCATTTTATTCACTGACCACATTTCCCAGTGATGACAGC
AACTCGCCTCCAGAAGAGAACCCATTTCTCTGATGCTATCTTCCCTGCAGCTGCTGC
GTGGATGTGCTGGGTGCTGAGCAAGCTGGAGCTCAGGCTCTCATACATTTTGGC
>non-DHS40
CTTCGGTCAACCCACATGTAACTAGGTCCTGTGTTGCTGCTGGGAATATAGTGGTGA
ATAAAACAGTTTCCACCACCAGGGGAGGGCCACCTAACCCAGCTTGACTTCCCAAAG
GCTTTGACATTTGAGCTCAAACATGAAGAATAGATGATAGGTAGTAGTTTGCTGGGT
GTGGAAGAGTGGAGGTTGGCATAAGAAAGGCTGTCCAGGCAACGTTTAGCACATGCA
GTATA
>non-DHS41
GTTTGAGGCCTGAGAGCAGTGTGGTAAGCCCAGCAGGGAAGAAGCCCCCTATACTTC
TCTAAAGCCTGGGTGCCTGCTTGAGGAGGGAAGGCAGGAACCCAGGGGCAGGGGAGG
GGATCTCTTCAGCAATTATGATGACCACTGTAACCTCCTGGCCCAGGGTTCCCAGGA
TACTGAGTAAGGGGCCCAAAGACTATAAACCCAGCTGTGAGAATGCCTGTTTGCTG
>non-DHS42
TGGCTTCTAGGGAACAGTTAGTTGTTTTGCACATAGGGAAGGAGGTAGCGGGCAGGC
ATGAGGAGGCTGGGCTGCCATGAGCAGACCAAATCAACAGCCTCCCAATCTTCCTAT
CTCCCCACCTCACCCCACCCAGCTCCAGTATGATAACCAGGCAACTGCGGCCCAGCC
CAAGAAAGCCCACACTTGTCCTCCCTCTGCCCACAGCAACAAAAGCAGTGCAAG
>non-DHS43
TCAAGCCACCTCACATTTCTATTGGTCCTCAAAATTAACTTCCTCCTCCTGGAATGG

GATGGAGGGAGGAAAGCCCTGCCTCAAAAATTACCAGTAGAGTCATCAGCCCCAGTG
GTAGCTGAAGCAGTATACACACCCAGGCATGCAAAACTCACACACGGCATGTGTGCA
TACCCACTCCCACATGCACACTCTCACACACTGACACACAACACTCACACTCAC
>non-DHS44
CTCACACACTGACACACAACACTCACACTCACATACAAAGGCACCCACACATGTTCA
TACATGCCCGCACTCATACATGCTTGCAAACTCATGCACACAATCATACTCCTATTC
TCACACTCACACCTTCTCTCACGCACTCACACTCTCACTCTCATACACACTGTCACA
ATCACATACACTCACATGCAGTCTCACACTCATGCTCCTTTACATACTTACACGTGC
ACA
>non-DHS45
CAGTCTCACACTCATGCTCCTTTACATACTTACACGTGCACACTCAGACTCACACAT
TCATGCGCACTCACACCACCAGCCATCTTCACGCCTCTCCCCACCCTTCTCCTTCTC
ACCACCTTCCCCACTCTCTAGTGGTCACAGACTCATTAAAGAGTAGCAGAAACACAG
GAGGCCTCCAGGGCTCTCTGGAAGGTTTAGGTATCTGGGACAGGAAGAAGTAGGAGC
AGGGAGCTCT
>non-DHS46
ATCTGGGACAGGAAGAAGTAGGAGCAGGGAGCTCTTGTCTGTCCAGCATGACAGAAT
CCCCAGGGATGGCGGGGAGCTGGGCCTTGCAGCTCAGGAACAGTGGCGGGGTCACTG
GTGCTACAGCCACCTTCTCATCAGGAAAAAGGAAAAACCCTTCAAAATGGTTCTTTT
CAGCTAGTGCCCCCCAAATCACAGCCCTGGAAATGAAGGAAAGGAGGAGAAAGAAGC
AGGACAGAGCAGGTTCCTCTGACCACTTCAGGAGA
>non-DHS47
GAATGACTGAGGGTGCTGTGGGAGCCTGAGAAGCCTGCTGGGGCTTCAGATCTGGGG
GACCCAGAGTAGAGGTGTAGGTGGGAGGGCCTGGCCAGCCCCAGGCCTGCTACTCCT
CCCCAACCCCACCGCAGCCTGAGAAGATACATACTGACCCTCAGGTGCCGGGGGCAT
GGCACTCAGATCCACGGGCTGCCCCTTCTCCAGGGCCTCCAGGACAGCACCGAATCT
CTGCAGAAGTTGGAAAAGCAGGTGTG
>non-DHS48
CTCCCGTGTGGATGTAACTTTTCTCAGTGACACGGTCCTCTCTTGCAAGTGGTGGCC
ACGGGGTGTTTCTCCCTTGCCCAATAAGAGGGAAGGCTCATGTGAGAGCCGGATGGC
CTGCCTGGCCGGCAGATGACCACTTCCCTCTCAGACCCCTGGGACATCCGAGATGCA
GGTGAGGGGTGGGTCACCTCCCCCACACCCTTCCAGAACTTTCCAACCTGTTC
>non-DHS49
CCCTGTGCTTATGTTGCATTAAGTCAAAAGCAGAGTGGTCTATTGATTTAGGAAACA
CTAGCCCAAGGGGAAGAAATGACTAGAAACAAAGGACTAAATGTTTCCATGTCAACA
GAAAATAATGCTAGCAACAACAGCCACTGCAGTAAGCGCACTGGTCTCTCTTAAAAC
CCCAAATCGCCAGCTGTTCGGCTCAGAACAGGTTGGAAAGTTCTGGAAGGGTGT
>non-DHS50
CAAGTCAGGCCATGCTACTCTTTCGGCGGACTGCCTTGTTTAACCTCCGGAGTGCTC
TAGACAGAAGGTGCCACCGTAATCGCCTTTCCTAGATGAGGAGACAGAGGCTCCCAG
AGGGCCTGAGCCCAGGGCCCCTGAGGAGTGAGAGCAGCAGTGGGAATGGAGCCAGGG
TTGAGGAATGAGCCCAGGGCTGTCTGATAGCAAACCCCTGGCCCCTGTGCTTATGTT
GCATTAAGTCA
>non-DHS51

CGAGTACCTGGATGCACCTTCCCCCTGTTCCCCGTAATTGTTACTGCTCAGTCCTGC
TGGCTTCTGTTCATTCGCTCTGCTTCAAAGGACTTTCTCTCCTGGAGCCAGATTGCC
TGAGTTTAATCCAGGTTTCCCTACTCGCTACTACTACTTACAGGCCTCTCCATCTCC
CTTCCAGCCTTAAACACCTACTGAGCTCCCCTGGAATTCCCTGTCCACGTCCTACAC
ACTCTCCTCATGGAGTCC
>non-DHS52
TCAGTCTTGTTTCCAAAGGCTGTAGTTGGAACTGTGCTTTGTAGTGTTCCCCCGCCT
CCCCCCCTCCCTCCGCGGGGGAGGGAGCGGGAGCTGGCGGAGATAGGAGAGAAAGCA
GGAGCGGATGTGAAGGGCTTTGGTGCTGAACTTGGATCCAGCCCTTGCCTAGAGGGC
GCAAACAATCGTCCCATATCCAAAAGTCTTACGAAGGGCGCCAAAAGTTACTTAAG
>non-DHS53
GTCACTGAGTGCAGAGAAAGGAATTTCTGTGATGATTCCAAACTTAGAGTCAGTGCT
AAGGAAGAATCCATAGAGCTCCAAGGACTTCAACCTCAGGGGAGAAAAGCAGTAATT
TATGGATCTTTCTAGAAATTACGGTTGGGTTCTTTCCCCTCTCAACCCAGGCTTCCT
TAGTCACGTGACTGGAATTTAATTATCAGTGCCATAAATAATCTTGTGAATGGAAGC
AGTGTATTTG
>non-DHS54
AGTCTTTGCAAGAAAACTCCAAGGTATTTCATGTAATCAGATCCAAGAATATGAACC
CTACCAGATCTTTAGACATGCCCAGAGAAGACCGAAACCAAGATGTTCAACATGTCC
AGACAAGTAAATCTAAGACTAGAGACATTTATCAGTGCAGGATACCTCCCCTATCTT
CACGATTATCTGCTAACAGCTATACCCTTCTTCTTGGAGAAATTGCTCTTCCCCCTT
TCCAAGTAGTTGTGGTTGGACTGCTGGTTATA
>non-DHS55
CATATGCAAGGTTGGCATGAACAATGGGTCAGGCTGTCTCTTCTTTTACTGTGCCAA
TCTTAGATCTCAATAGGGAGATCAATCCTAGTGAACCTGATTCTGGGCAGTCTTCAG
ATACCTACAGCATCTTCTTTCCCAGATTTGAGCACTTATAGTCTACCAGGAGCCACA
CTTAGTGATTTAAAGTCATCTGTTTTAATTCTCACAGCAGCCGTATGATTTCAGTAT
T
>non-DHS56
AAAAGTGTGATATCTGGGCCAAAGATTATATGTGCTTCAAAATGTATTTTGTAATAC
CATATTATTTTCTAGCAATATTGTAGCAAATCACATTCTCAGAAAAAACAAAGAAAA
AGCCCAATTCTGCATGTTATTGTCAGAACTAGATAACTTCGATTTTCAACAATTTTA
AATTTGATCGGTAAGACCTTTTTTTTAACTTTCGTGTCCAAGTGCCCCATAATTAAG
>non-DHS57
ATGTTTGCTCATGGAGCAGATTACTCCTTGGCTGGCCAACAGACTTAATTGTTTAAA
ATTATCCTAGCCAAAAAGCCAAGGCTAATCTGCTGCCTATAAAGCTCACATAACATC
TTTGGTGTTTTGGCCTGGGATAGGTGACAGATTTTCTATTGTCCTGGTTACCTTCCT
CCCAGGAATGAGATCACAAGCAACGTCATGGAGTCATTACTACTATATACACTCTAC
AGGTCAGAGATTGGTCATGG
>non-DHS58
GTATTGGCCTTGTCTTGGGGTACTTTGGGAGAAAAAGCCTTTTGTCTGTGTTGATAG
AATAACTTGACATTCAAGTGCTGTTCTAAGTATTTCCTGATTTTTACAGTCCTAAAT
GGATTGGCTTCTTTCTGGTACAATAGGTCAGGCTACATGTGAAACACTTCTGCAGTT
AAAAAAAGAAACAAGAATGTAAATCATGTGCCCTGTTAAAAGCAGCCAGTTCCTAAG

ATGATTT
>non-DHS59
GACTGAACTGCACTTTGGTTTCATATTATTTGCTCTATAATATCTGAAACCATGATA
TGATTAACTTATGTGATGCTTAAGACAAGTTTAAAGACACAAGATGGGAAATAAAAT
TTGGGAATATTTTTTCATGGGTAATGTGGCTAATGTCTCTACCTTATTCTCAGCAAC
ATATTAGAGTATCATATCTAGAAATGTGTAAGAACACCAATCACAAAAACATGAGTC
TGCTTAGTTAACACAGAACACATGGAT

>non-DHS60
GTCTTTTCCGTGGTAGATCAAACGCGAGGGGAGTGAGGAGGCGAGCAAAAGATTGTA
ATGACTGAAGCTGTTTAATTGTGATTTGTGGACGTTTTCCTTTAAAGACATTGGCAG
AGTTGGGCTAGAACCTGGAACTGCCGGATCTTGGAGTCATATCGGGCATCTATCATG
AAGCCGAATAAAACCATAGAACCATCTGCCCATAAATCTCTGCCTGTATTAACTGGA
GTGGTCGCTG
>non-DHS61
AACCATACAAAAGGTGGAGCTCTGGGAGGCTTGATGACTGAATGTAAGAATAAGGAA
GCAGATTATTTATTGCTTAGAATCTTAGATTAAGGCCAGCAGCAAAGAGACACGTTT
CTATCCAGAATGCCTGTCTATCAGCAAATAAGAAAAGCAAGGTGATTATGTAATCCT
GGGGCAGGAAGCCCATTTTGTCTGTAACTGGAGCTCTCTGATTACTTAGATTCAAAT
CCAGAAACTAATGGGCCTT
>non-DHS62
AATCCAGAAACTAATGGGCCTTGCCAAACTGTGGTCTAGTCCTAATTTTCTGTCTCA
TTGAGTTTCTAGTGCTAAAGCTGTGATTGACAAAGATGGATATTTAGGCCATTGGAA
AAGGATTATCTAGAAGGAGTTGCTGGGGAACATGACACTCCCACAAGCTTCTCCAAG
GCATTTTTTACTCTGCCTTCCCTACAAAACAAGAAGATCCCTTCCCACTGGGAGGCA
AGTCTAACAGCCAGGATTGAAG
>non-DHS63
CAAGTCTAACAGCCAGGATTGAAGTATGGATAAGGAAAGACAGATTAGGAAAGAAAT
AGCTAGCACTTTTTGCCTACGTCCCTTTTGTACTAAGTGGCATCGGTCATTAAGAGA
AGGATAGTCAGATTGAAGAGCCTAATCCTGGGTTCTGAGCAACAGAAAATTGATGGG
CCTGGTCCTCAGGACTTATTTCAACTATTAAGAATATGCAGGCTTCCAAGGACATTG
GCCTTACAGTGTCAGTCAG
>non-DHS64
CATTGGCCTTACAGTGTCAGTCAGGGTTCCCTGTAGCTTCAGGATTAAATATTTGCT
TAGAAAAGTAGCCACTGTATGGCTCTTATCCTAGAGATCTCTTTCTTTCCTCTTCTA
AGTAAATGATTGTTTTCTGCTATCTTCCCATTTTTGTTCCATTGTTGAATATTGGGC
TATGGCAGAGGTTAAGTTTACCATTTAGTTCATAGATTGTAGGAAGATGAGATGTCA
CCTTTGGACAGGAGAGGAATGGATA
>non-DHS65
GTTTTCCATTGAAGCACACACTTCTCCCCATAATAATATAATGCACAGCACCTGTCA
GGGATATTAACCATACATACCCACTCAAATGCTCCCTTTCCCACCAAGCTTATTAAA
ATAATAGTTTGTGGCCACAATTTCTTCCCTTCTCATTTATCCAATACACAGCAATCT
TGTTTCAAAACTCCTATGCTGCTACAGCTGTCCACAGTCTCATCATACGCATTCTTT
GC

>non-DHS66
GGCCTTCGAAATCAAGGTATAAACTACAACAAAACACTTTCGAGATCTAGTTTGATT
GAAGAGTGCATTCTTACTTATTTATATATTCTCTGCAATTTCTGATTCAAGATAGTC
CACAATTCTAAAGGAAAACCAAAATAGAATAATAATTACAAAACAAAATAAAAAGTA
ATTGAGGAAGATAATTGAGCAACTTATTCTTGACAACTAGCTTTTTAACAAACAGAA
AAGTGCACTAAAAATACTGCCTAGTTGTTTAAAGTAGGCTG
>non-DHS67
CCTGAAGTTCCAGAGTCTCTTAAATGCTAAAATATTAACAATACCATTCCATTTTTT
CCCCTAACACTCAGAAGCATACTGATACCCATCCTGTACACAGCCTTTTCTTGCCTG
ATAACAAGAGCACTAATGACAGAGCTATTGCATCAGGTTGATTAATAAACCCCGTCT
TAGGCAGCCTCATCATGTGGATAAAGAAAAGGAGTTAAGATGATGTTCCTGCTGCCT
TCCCCAACATAGAAACACACCCACAACA
>non-DHS68
AGAGTTTGGGTCTACAGGTGATCTTTTTTCCGGACTTTTAGCAATTGCTGTGGAAAA
AAACATTTTTTGTCATTGACATTTATTTATATATATAAATGGATGAATGGATGGATG
GATAGATGGATGTGTATTTTTGAAGTCTAGTGGAAATGTTTGATCTCATCCTGGAGC
TAGAATGATAAATACTGTTTTCCTGGAGCAGAATGAAGCAGGGAGATGTGTAAA
>non-DHS69
AAAACATGGATCATCACTCGAGACAACGATTTCACATCGTCTTCGTTTTTGATGTCC
AGTTTCCGAAGCATGCCTGAGAAAGAAAAGCATGCAGGTCCTCACGGCCTCCTTTGT
CTAAACCGGCGCAAGATTCGCCTGCCCACCCGCGGCGGGAAAATCGCTACTGGGATT
TACAGAACTCAGGTTGACCCCACTTGAAATTGACATCCCACCCTTTCCGGTCTTTGA
ACAAGAGCTGCCATTT
>non-DHS70
AAAAGGAGTGGCTGGCAGAGTGTCCTGGCACCAGGAGGGGACATGAGGAGAGACCAG
TGGACCAGTGCATGGAGCCTTGAGGAGAAACAGGACAGGAGAAAGGAAGGGAACTAG
CATTTATTAATCACTTACTGTGTGCTGGGCCCTTTGCAGGACACTTTATGTAAATCC
ATGCTTTTACTTTGTGTAAATATTCACAGCACCCACCACAGGGAAACATTATTATTC
C
>non-DHS71
GCCTCCGTTTGTTCCTTTACTTACTTGTGAAACCATCTCACCAGCCTGGCCTAGAGC
CCAAGAAAATATTATGGGCATGTCCTGGAGGTCCAAGGAGCAAGAACTAGCATCCTG
AAGAACAAAACCTCTGCTGAGCTGGAGTGTAGTTGACTGACTCATCTTGGTTTTAAA
ATGAAAGTCCTGCATTCCAGGAACCCCTTCAGTCCTTATTAAAAAAAAAAAAAGATT
ATTGGTCACTCAAAGTGCAAGAATCCT

>non-DHS72
ACAAGGAGGAACATTGCTGTGATCTGGCAGGAGCTTTCCACTGACTTCCACCTGGAA
CCTGCCATGGTGGCTGCATTTAGGGAGCGGGGCAGAAGCAGACACCAGGACCTGTGT
GAGAGGCAGCTCAGCTCTGCTGGAGGCCATAGGGGGAACGTTGCTGGAGGGACATTA
CTCCTGGGTGACCCACTGGATGGGGGTGGGGGAAATTCTCCAACAAAGTTAACCTGG
AATTGGTAGTTTTGACAACG
>non-DHS73
CATCTGTTTCTTGGAGGCTTGCCCCACATCAGGTGGGTGGGACACACCCAGGAGGCT

GTGCTCAGGATTTCTGGCCAGTGTCCCAGGTCACCAGGTCCCACCCCACCCCAGAGA
CCTCACCTGGAGTCTTCTCTGGCTCAACCTTGATTTGAGGGTAAGGCAGCGGTACAT
GTGTAGTAGCAGGTCAGAAAATGGGAGAAAAGAGCACTGGGCCTCTGGACCCAAAGA
GGATGGAGTTTCAGACT
>non-DHS74
GAAAATCGTTTGACCTCATCTATGCCCTGAAAAAATATTTGGGACTCCCAGGATTTG
CGGACCACACCTTGAGAACTGTTATGTTACTCAATGGCTGGCATACAGTAGGAATCA
GATAATTAGTATGGACTGAATGGAGAGGCCTGGGAGCTGGGGAGGAAGTGGGGGTCG
GGTGGGAACATATGGATGCCTTTCAACCCAGGTGAAATTTGGGGATTGTCCACGTGC
TCCTCC
>non-DHS75
CTTTCAACCCAGGTGAAATTTGGGGATTGTCCACGTGCTCCTCCATTCCCCATGTCA
GTGTAGACAAATGTTGAGTCATAGTTTGCAAAATGCCTCTCAGCTGGGCCAGGAGCC
CTGGTTTCCCTGCTGGACCCACCCCTCACCCCTCCTTCCCAGGCAGGCAGGCAGGCA
GGAATGCGGGGGAGAGGTGGCTGCAGCTCCACCGCCCTCATGGGGGAGGAAAGTGCC
AGACATTTCTTCAGGACCATC
>non-DHS76
GGAAAGTGCCAGACATTTCTTCAGGACCATCCATCAAGTGCAGGCAGGCCTCTCCCC
TGACCTCCCAGGCCTCTTCCTGGAGTCCACCCTGTGGGACCTGGACGTAGGGTGGCC
ACTGGGAGCTTGGAGGTCTGGCGTGGCACTGCTCCCTGGTACACGGCTGGCAGTTTG
GGATAAAGAAAGGATAAATTGCTCCCCTCCTGTCCAGTGACTCTTGTTTTGTGCT
>non-DHS77
CAGTGACTCTTGTTTTGTGCTTCCCGCCCACCCTTTTTGGCACCAGTCAGGCTCAAT
TTATCGGGAGAGGGAGCAGACATGGTCCAGTCCTCTCACCAGCCAAGCCGGGGATGA
ACAGCACTGGGAAGGCCTAACCAAGAGTGGTTGTCCTGTCAACCACAGCTACTGAAC
TTCCTGATGGGCCCCGGTGGCCCTGTAGCCTCCCATGCCCCAGTTCCTCCCCACAAG
GGGAGCAGACAGCCCCTGAAGAGACTG

>non-DHS78
CGGCCAGAACATTTCTTGATTACCATGATAGACTTTGGAGATACAGAGATGTCAAGA
GACTCGACCCCTTTCAGGTGGAGGGGGAAAGCAGGAGTCAGTGAAGCAGTTGCATTG
ACAACTAACCATGAAGGCTTGAGATTATTGTTAGAACAGAGGTAGTCATATTTTTAC
AAGGACAACAACCAATATAACTTGGAGCCAGAGGAAGAAGGGCTCCAGGAGTCTG
>non-DHS79
CATTAAAGACTGTGGGACCAGGACCCTAAGTCTCCTTTCCTTCTGGGTGGGGAGTTC
TGGGGTTCTTGGTGTGTGGGAGAAGTTTTATAATTGCTTCCAAACAGCTGGGTTTAA
ATATAAAATAGACACACTCATTTTTGGCTCTTGGTTTGTGTGTGGGAACAACATGAG
TGGGAAGGAATTTCTGGGTGCAGAAGGAAGCAGCCGGGGATTTGACCAAGCAGGGTA
TCAG
>non-DHS80
CCAGTTCCAGTCCATCCAACTGGATGGTAGCTCCCCCACCGTTTTCTCTCTGCCCTG
GTCCTAGGGGCAGGGGTGCGTGTGCGTGTGTGCGTGCGTGTGTGTGTGTGTGATC
ATCATGTCTACACCACCCACCACCATCACCCCTATCCTCCAGCTTCTGCTATAGCCT
GGGAGTTCCCAGCCTTTTAGTCCCTGTCAGGATAGTTTCATTCTTGTTCAGCTTTGG

GGCCCACACAATTTCAGTACAAAAGGG

>non-DHS81
AGGTGAGATCATGGGAAAGACTTCTCACATATGTATGTTAGGGGTACAAGATCTGTG
GTCAAAATGGGCTGTGTGATCCCAGACAAATCACTTACCTTTTCTGAGCCCCAGTGG
TAACTCAGGATACCCTACCTGCAGGCTTGTGGAGAGGATGGAGGGGGTGGTGGGAGA
GTACTTGTGAACTGTGGGGTGACATGAATGTTGTCAGTTGTCAGGGAAGGCAAGTCA
TGTCTAGTGGGACTGAGATAGAGCTACCCTGCA
>non-DHS82
CAGAGGGACAAAATGACCTTTCTTTCAATGCCATACACCACTTTGTCTAAGGCAGTT
GAGGAAGGGAAGTAGATATAATGGAGCTTAGTGGCAGAGCAGCTGGAATCCTGAGAG
AAGAGAATAACTTTAGTTCAGAGCAGGTGGGGAAATGAGAGATTGAGTAGGAGGACC
AAGGTTGAGGGAAGCAGATCTTCTTAGTTGCTGTCAGCGGCTGATGGGGAAGATTGT
TGGTAGAAG
>non-DHS83
CTTTTGCTGCCTGTCGTCACCGCCACCCCAACAGCCGGGCAGGCATCGCTGCCGCCA
TCTTGGAAGCGGGCGCTGCGGGGGCGGGGTCTCGCGTCATGGGGCGGGGCCTCGGGG
CGGAGCCACCCGGGCTGACTAGGCACCCGGTCCCAGACACCGCGTGGCCGGGGGCTT
GTGAGGGAGCAGCGCCTGAGCCTGCCCACCTGGAACGCGTTGTAGGCGACCCCCTGG
GGAGCGGGTGCTAGGGTTCAACTCCACTACGCAGACT
>non-DHS84
GAGGCTCATGGAGAAAGGAAAGGCAGGCAGAAGGGAGGGAAGCAGAACGGCCAGAAG
TGTAAGTGGGTGGAGAGAGGGTGAATAGGGGTCAGTAGGACAGACATGGCGGGAGGA
CACCGCAGGACACAGACTCCAGAGATGCCTACCTTGGCTCTTCGGGGTCTGGGGCTG
GGGCTTCCAAGAGGAGCCTTGACGTTGCACATGGGACCTTGACCGCTGGCAGGACTC
TTCCGGCGTAGGATGT
>non-DHS85
CTTTTGTTTCAACAGAGGGCAGTAGAGAACAGCAAACAGTTTACATTTCTAAGCCCA
GAAGAAATAAAACAAATCCATTTACAACATTCTCCTCAGGTCAAGGTACCCAAAATA
TGGCACACTGTGCTTCGTGTGTTCATAAATGGGGCATGTCCTTGTGAAAGTAGGGCA
GATCCCGGAGAGTCAGCGACTCGTAGACGACCCTTCGTCTTGTGAACAACAGTTT
>non-DHS86
GGTTATCTGGTTCCTTGCTGATCTTCCTACTACAATGTACTCCCTTAAATCAGAGGC
AAAGCAGCAGCATAAATAAGGTCTTGGGAAAGACTACATTCCTTGGAAAATAATTTA
ATATAAATCTGTTCCTCTCAAGTGTCTTTTTTAATGTGATTTTTTAAAATTAAAAAA
TAATAGCATCATGACTCTTGAAACCTATATAAATCAATAGGAATGCCTTTTAATACC
ATAATGAACATTGAAAGCTGAGTGTTGTTATTACCACC
>non-DHS87
ATGTGCATTCCTGGAAAGGAGTCGGGTTTCAGAGCCTGATCCGCAGCGTGGCTCCTC
ACAGCACTGCGAGCCAGAGCTTGGCGCGCGCGGTTCCGTGGGAAATCTGAACACTGG
CGGCTCAGCAGCCCGGTAATCTTATCAGGGCCGCAAGAGAAAGCATTTTGGTTCCAG
AGAAATCTGAAGGCAGCTCCGCTTTTCTCCAGTAATAATATTTAAGTCTGTGGAGCC
AGCCT
>non-DHS88

CCTCTAAGTCACAGTTTCTTTCCTTGTTAAATGAGAATCCTGGTACCTTCTTTAGAA
GAGTGTATGAAATGATGTATGTGAAAATACTTTCTAACTATGTTTACATGCGCTAAT
CTTGCTTTTTCCTTAAAGTTGATATGTGAAGTTTGAAATGTTTAAAATGTGTATTTC
AGTTTTAAGAAAACTCTAAACATGATTGTGGTTAAGATTGAAAAATTAGTGGAGCAG
TTGTCGGTATG
>non-DHS89
GCTTCAGTTTGGAGATAGGGTAGGGTGCATGGAGGCTAGGGCTTGGCATCAAATGCA
GGTTCTGGGGTCACCCTTAGGCCTCTCTTCCCTGGCTGAACCTAGGGCTTGGGCCAG
CAGGAGGCCTGCTGGAGCTCTGCCCTCCACCTCTGGCAGGCAGCCCAGGCACCTGGT
TCTTGTCCAGCTCACAGTTCTTGTACTCAGTCTCTCCCTGCTCCTGGAAGGTGACAA
TGACGAAGCCCACAAAGATGTTCATCAT
>non-DHS90
CTCAACAATCAAGTTACGGGACAGGAGTTAGGTCGGCGCCAGGAAAGACCAGACCCA
ATCCTGGAATGGCCCTGCCTTTCAAGAGGTTGCCCCATCTATCCCTAATCAATCAGT
ATTAACAATCTGCCCTGTGGGCAGAGAGGCATACACCTATGGGGGACTGCGTGCATT
TTGTCAGAGACCCCTGGTCAGGCCTACTTTGGAGTGACCAGTCAACATACCATCAAA
CCAT
>non-DHS91
GGAGTGACCAGTCAACATACCATCAAACCATCAGAGAGTTGGTGGGTTTGTTGGACG
CCTGCCACAGGTAGCAGTAGCACCGTGGGGGCTGCAGAAGGGCAGGCTGGCTGCCTG
GTCCTACCTGACCATGTCCATGAGGGACCTAGGCCCCAGCCATCACTCACTGAGGCC
ATCCCTCGAAGGTGGAGACCGTGAAGAGGGACATCATGGCTGAGAGCACATTGTCGA
AGTGGAAGTCGCTGTGTAC
>non-DHS92
GAATCTGGTCTCCACCTAGACTGAGGTCCAGACATGCTCGATTTCCTTTATCATGGT
GAAAAGAGCAGGGGTCCCCAGTGCCTAGTGACCACCAGCAAGATGAGGCCCTTAGCG
CTGTGGAGCCCCACCCACTAGGGTGTGTCTGGACTGCTCAGTGGGAAGGGGCAGGGC
TGTCAGTCATTTCCCTCCCTCTGCATCATCACCGACACTCTTCTGGCAACCAGTCTT
TATTCA
>non-DHS93
ACAAAGTTTGGCTTTCCAGTAAGGAAAGGGGCTGGGGCCAGAATGGTATGGGGAAGG
GAAGGGGCAGAGCTGGGGAGTTCTGGAAAGAGAGGGCAATCCTTCTGCCCAGCACCG
TGGGGCTTACATAATGGGCTGGAGGTGGGGGGCAGCCCCAGCCAGTGAGTGTCCAGA
GCTGAATCCTGTCACCTTCTCCAAGAGGGTCTCTCAGGTCTGGGAAGCAGCGGGGCA
TGGAGAAGGCAGTGCCTATGGAAGGGGAG
>non-DHS94
ACGCTCCACTGTGTGCTACAGTATACATCAGGCATTTCTAGTTCTAGTCCTAGTCCT
GCTCTTAACTTTCAAAGGCAAATCAGGTTCAACCTCAATGTGAAAGAATAAACAATA
AATCAGAGAGCATTTATTATAAATTGCCAAAGAAGGTTTAGTCCAGTCAGCCAAGGT
GCCCCTCTATTTCTCAAATGCTAAACCCACTCAGAATGAGAGGAGCTGCTCCATCAA
CCGAGTAGATCCAGA
>non-DHS95
GCTCAAAGGAGCATTTTACAACAGGAACCATTATCCCTGGTAGCTTGACGTCCAGCC
CTGACACGATTTTCTGATTGACTAAAATTAGGGTAATGAGTTATGAGGGCAGAGAAT

GGAGCTGCGATCCCTGTAGGCGGGGGCAGTACCGCCTCTTATTTCCTCTGCCCAGGT
GGTTAAGTTGGTAGATTGGGGTGTAGGTGTCTCTGGAGTTGGGCAGCAATGGGTGGG
GTAGTAGAAGGACACCTGGTCT
>non-DHS96
CACAGCGCCCTCTAATGAAAGCGCCGCCGCCGCTGCCTGGCCCGCTTGCCCCGCCTG
GCCCGCTCCCCCTACCCCTGCGCCAGCGAGTCGGGGCCCTCGGGGCTACCTGGACGC
TCGGCCAGGACCTCAGTGAGTCCAGAACATACTGAGGGAAAGGGCCGCTCGGAAAGA
TCTAGGAATGAACACCCATCCTCACACCCCAGGAGTGCCTTGGTTTCAGTTGAG
>non-DHS97
ATAGGTCAAGAGAGGCAGGTCTTCACCTATTACCCCCCCAAACAATATGCTTCCCTT
CCCTGTTTCCTCTTCAGGATTTTCAGCAATCACTGCCAGATGCAATCAGACTCCTTG
AATAGGAGCAACTGAGGCTTGTTTGTGGCTCGGATTCATTAGAATCAGGAGGCTGAA
GAAAGGAATAAATTACTTAATTGCACAAAAAAGTTGCAGCCAAAGTGATGGTGAGAG
AAACTGATTGTG
>non-DHS98
CCTAATAGTGTGAGCTGGGGACTTGGAGCCCTGAGGTGCTCCCTGGGGGTGAGGGGC
AGTGAAGTAGCACCAGTGGGACTTAGGGGGACACAGGGGAGCTGCTGCCTTCCCTGG
CCCAAGTAACACTGGGAATCAAACAGTCCCTAGGGTAGGGTGGGTAAAACAGGAGGC
AGGGGCTTCTCTGGCCCCAGTCTCCCAGGGCACCAGGCAGTGTGGTGCTGCCTGACA
CAGAC
>non-DHS99
TTCAGAGGACCATCTTTCTCTTCCTGCAGGTGTCAGCGGAAGCAGTGGCGGGTGGAT
CTGCCGGCCACCAGCGTGGTGATCACGTTTCACAATGAAGCCAGGTCGGCCCTACTC
AGGACCGTGGTCAGGTGAGGCCAGGAGATGCATTACCTGTCAGGGGTGTTAAGACAT
TAGCTGTGTCCCAGGCACAGTCAGACCAGGGCTGTCAGTGGGGGCTGCAGACAGGGT
CTCCTGACTGCTCAGCACAG
>non-DHS100
AGACAGGGTCTCCTGACTGCTCAGCACAGGGTGCAGTGTGTAGCTCCTCCAACCAAG
GGTTAGGACCAACATATGAGAATTTTCTACCCCAGGCTAAAAGTTTATACCCCTAAA
AGACCTATAAACTTTATCTTCTTGTCTTTTCTTAGCGTGCTTAAGAAAAGCCCGCCC
CATCTCATAAAAGAAATCATCTTGGTGGATGACTACAGCAATGATCGTGAGTACTGA
CACTGATTTTATCCCTGTGTCTGG
>non-DHS101
CACTGATTTTATCCCTGTGTCTGGCTCTTCTCTGGGCACCAGTCTTCCTGTAGTGGG
GGTGCTAATGAGGCGTGAACAAGCCAGAAATCAGATCCACAGAAAGAAGAGGTGCCT
CTGTGATGCCCCAAGGAGATAATCGGCCCTTAGATGATTGAGAATACTATGGTTGAA
TGCAAAGCCCTTTATGAACTCCAGGTTCAAAATGCTCTGCTTCTTTTCTTTTCCTAG
CTGAGGACG
>non-DHS102
TCTGCTTCTTTTCTTTTCCTAGCTGAGGACGGGGCTCTCTTGGGGAAAATTGAGAAA
GTGCGAGTTCTTAGAAATGATCGACGAGAAGGTAAGATTCTTCTTAATTCAGCGCCA
AGACAGTTGAATTCTGACTTTTCCTGTAATAACATAAAGAAGTGCTTTAGCTCAAAG
AGCAATTAATTGTCTAGCTTTTTTCTGTCTCCCTCTACCAGAGCCGCTTGGGAGACT
TCTCCCAGCAAACACAGG

>non-DHS103
GAGAGGATTCTCCCAGAGATGAAGTTACTATATTGTTTTATTCCTTTCTACTGTCTT
CTTTGCTAAAATGTTCCCACCTCCCAACTTAATAAAGAAATCCACAACCCTGTCTCA
TGCATAAGTAGCTATGACCCATCTCTCTCCTTTCATTCCCAATCAGTAGTTGTGAAT
GGTTTCCCACGATCATCACCTTCACAGCTATTCAGCTCTTCCACTTGGCTCCACTCC
ACATTGCTTACTAAGGTCA
>non-DHS104
CTCTTCCACTTGGCTCCACTCCACATTGCTTACTAAGGTCATTAGTATCTTTACAGT
TTCAGAATCAGATGGACATTTTTTATTCTCTTCTCACTTGAGAGGCAGTGTCATGTC
ATGAAATGAACCTCAGCTTGCTCCTTTTTCGAGATTTGACTTAAATGTCACCTGTTT
CAGCAAGGCACATGCTGACATGTCTTAGAAGTGTTGGGTACAGTTTTCTCTTTGTC
>non-DHS105
CCGACACATACATTCCATTTAGAACTTTTGTCATTGTCTTATCATGACTTACAGATC
TGTCCCCTGCAGTGGCATACGAGGTTCTTGAGGGCAAAGACTATATCTACATTAATC
CTGTCCCAGTGTCCGGCCAATAGAAGGTATTCAGTAATAGCTATTGAGTGAATAAGT
TAATGAATGACTGAATGAATTAGTAAATGAATACTCATACATAAGTCTGGAAAACAG
AGACACGAAAG
>non-DHS106
TCTCAGCTAAATACCAGGCAGACCAAAGATGGCTGTTTATTTTTCTTGTGCTTCAAA
TAGGCTTACAGAGAGCTCTGGTCCAAGGCGTAATTATACGGATGTGGTTGTGCTTCA
GTCACACCAGGAGGGACCTGGAAAAATACTTCTGGGAATGGCACGTTGACTCCCAAG
CCACCATAGCAAGACTCCAGAGCATGTTTATAAACCACAGCTTCACAACAGGAG
>non-DHS107
TAAATGTGAGGCCACATGAAAGACAGTACATCTGTTAAATTCTATAAATTGTTAAAA
GCAAAAAAGGGAAAGCTTCAACACCCCATCCCCTAATATTTTGAGTATTAAAAGCAC
AGCTGAAATATTAGGATTTGAAATCTGATACTGCCCATGTACAGTAAGTAGCTTTGT
GACATTTTTATCCAGCTGTGGAGAATCTCTGCACTGTCATCAGGTACAACAAAAGAT
CAAACCC
>non-DHS108
AGTTGGTACCACACATGCATCCTGCATGTGTCCAGATCTGGGAGAACTACAGGATCC
CAACTTGTGAGGTGGACTCCACAGTTTCCTCGGGCTTCCTGCAGACGCCTGCTTGGT
ACCACCTCTCCAGTGTGAGCAGAGCAACCTCAGGTCCCCTCAGAGGATAGGCAGCCA
CTCAGCATGCAGGGGCTGAGGTCATGGACCCCAAACATCCCATAGGGTCAGAAAGAG
>non-DHS109
GAGAGGTTCTTTACGGAGTGTGTGATCCGCATGAGATGGCATGCCGAGTGCCATCTT
TGCATGGCTTTATTTTTACTTTTAAAGATGAGAAAGCTAAAATGGAATGTCTAAAAG
AGTATACAGGTCCTAAAGCCACACAGCCAGGGTGATGGAAATATGATATGATTCAAA
TCCAGACTCAGCGGCCCTAAATACTGTGCTTCAATCTTTAAAACCCAAAATACATGT
GGACCAATGTTTGGAGCAACAAGAGGCTGTGCCTTTCTACAT
>non-DHS110
ATTCTTAGGGCCTGAAAGGAAGAGCGGATGGAGGAGCATGAAAGAACACAGGAGCCT
GCAGGTGAGCTGGCGTCCAAGAACCTGTTCGAACTGAAATGTGCTTGTGCCGCCATT
CCCATTTTTAAAGAGAGAATCCAACAGAAAAATGTGTTGATTCCTTTCAGATTTGCC
ATGCCTGAGAAACACCGTTCATTCAGGCCATGGGACTGCACCCAAGTTCTTGCC

>non-DHS111
TTGCTATGTGGCCAAGAACAATCAAGAAGATATGCTCTCCATGGAATATAAATCTCC
CTCCACTACATATAGTGTAATTTATCTCTTTATATGTAGCTAAGTTTCTATTTGTAA
TGCATACAAAGCATCAGAACAGATGAGAAAGAAATAAAAAGATAAAGTGTCTCTCCC
ATTTCTCCCCACTCTCTTTTCATTGCACACTCCAGAGGCAAACGCTTTTAAACG
>non-DHS112
CATGTTTCCTTCGTGGTGGAGCCATTGATGCCTGATTGAGAATCAATTCTTGAAAAA
ATTTTCTTCTGTCTTCTTCAATAGGCCTTTGAATATACAAGACCTCTTCATACTGTA
TTCTAAAGATACATTTAACCTACACATATACACACACACAAAGACATTAAATATATA
AAGAATTAAAAGCTTACATATCATACCAAATTGCAATGTCTTCATCTTCCAGTATCA
>non-DHS113
GGCACTGCTTCTGTAGGAACTTTCCATCCCAGGGGGTGGGGTGGGGAGGGGCTGAAG
CTTTGGGGGAGTGCAGGTGCTGTGGGGGTCAGCACTCCTCATGCTGCTCCCGTGCTG
CCTGATTACCGCACAGGCATTTCTGGGGCAGCCCTGCTATGCAGGATCTCCGAACAT
CAAGGGAGATGTCAGCGCCTCTGCTTAGCACCAACGTTCCCACAACCATCCCAAGAA
GCACCTATGAATGAGGCAC
>non-DHS114
AACAGTATTTACTTAGCCCTGTGTGGCTCCACACAATGCTTTGTGTTAAAGGATTCT
TAAGTGTGAGACATGGGCCCTGGACCACAGAGGGGACAAGACATGCACCCATGAGAA
GATCAAGAAATCATGCTAGGTAGCGCATAATTCAGGACCAAGTTGTGTGTGATTGTT
GGTGAACACATTAACAACAAATCAGCAAATGGAGAGAGTCTGTGGCCTTCCTCCAGA
GATCAGGGAAGCTTTAAAGG
>non-DHS115
TAGGTGGTGGTGATATGTGCTATGAACGAAAAATGAGGTGGCTAGGAGAATAGAGTG
CTAAAGGTGAGGGGATGGCCAGTTTAGGGAAGCTGGCGTTGGCCTCTCTGATAAGAA
AACATTTGCACAGAGAGCCTGAGCAATGCAGGTGTCCTGAGGAATGTCCAGGAAGGG
TAGATAGCAGGTGCAGAGGCCTGGGATGGGAACAAGCTTTGCATGTGGCAGGGTCAG
CAAAAGGTAGAATGAAAG
>non-DHS116
ATAGCGTGGGTAACCACAGAAACTTGTGGAATCTCTTTTCCTAGAAAACATGAAGAA
CAGGAGAGAAGCCAGTCTGTCTGGAAATTGCTTCAAAATCTCTCTATAAGCACGATG
CTGGACTGGATTAGATGCCCCTTGAAGGATCCTTCCAGACCTACACCACTAAAATGC
TAAGAAAAATAACTAAAATAATTCTCTATCACTTATACCCTGGAAAATAAAATAAAT
ACAATTTAACATCTCCCAACATAGTCACTAGAAACA
>non-DHS117
GTGTTAGAACAGCCCCATTCAGAGTGAGTTCACTCCCTGCGCAGTTGGCTCACTCGG
CCTCTGTCCATAGAGTTCTCTTGTTCACGCTGCAGGAGGAAATGGAGATTTCCAAGT
GGGAAGGAGCCTTCCCGATGCTTTAACTCTTCACTGAAGGGAAGAGGAGCTGCAGAG
AGCGAAATAAAATCTAAGCCGCCCCAGCTGCAGCGCCTGGGAGAGGAGCAAGGCTGG
GCTGGGGATGGAGGCCAAGGCAGCTGGGATCACTT
>non-DHS118
CTTGAGAGGGAAAGGAAAAGAGCAAAGGGCATTTTTGATTGTCTTTCACAATCAGCA
AATATTTACCCAGTTTGTCTGCCCTAGCCCAGGTTCCACTGAAGGATACACGAAAGT
GGAAGATGTTGTGTCTGTGCCCTTCCCCTGGTACTTACAGCACAGTTATAGGAGTAG

AACGTTTGCATAGAAAAGCCACCAAGACAGGGAGGCCTTTAATAAGGGCCTATGAGT
AGTGTGGGCAGGAAGT
>non-DHS119
TTCCTAGCTGTGATTAGGAAGTCAGAGTTTTGGGTGGATGGAATAAAACTTAGTGGG
TTTAAACTCAAGAATTTTTTTCTAGCAAATAATACTTGACAGGCCTTGTAAGAACAA
TTAAGAATTACGTTCTAATCATAAATTCATAGCATATATAACATAATTAACTTTTCA
GGTATAGTAACATCAATTTTAAAATTTAACACTACCAAAATTGTTGACCTACACAG
>non-DHS120
GGCTTGTCAGGTCAGACAGAGTAGGAGCTTTATATCAGGGTCTGAGAGAGAACTACA
TGATATAAACAATGGGTGGAGCAAGGAGTAAGGATCAGAAGGCCTACAGAGGGCACT
GACAGTAAGAGATTCTGGCATCAGGAATAGCTTATTCCTGGCACACATTAGGAGTGG
TCACAAATGACATCAACTTACCACAAACAGGTTGGCAAACTGTCCAAGTTGATGTTG
AGCTTT
>non-DHS121
ACCATCTGACCTTGGTTGGAGCCCAGCAGTCCCCCACCCCCACCCAGTGGAAACAGA
ACAGAAATGCGTGTGTGCCCAGGACCCGGGGCATGGGACCTCACATGTGGCTACAAA
TTAGATGGGCTACAGATTAGTTGTGGGTTAGAACCCCAGATGGGTTCACCATCTTCC
AGGGGCTGGGGAACCCCCTCCACCTTGGCTGAGAGATGAGCCCACTTCAAACACACT
>non-DHS122
AGAGCATCCTGCATTTGTTGCCTCGGGGTGCAGCCCCAAAGATAAAGCCAGCAGTGT
GCAAATGACAAATGCTACCCCACCTCCGCCAGGCAGCCAGAGCCAGGGCCGAAGGAC
GCGGAAAGGAACTGGTGTGGAAACCTGCCCAGGAACCGCACTCTCAACTGAGAAGAG
TCCGGGGCGCGTCCCCGCCCGGCCGCCCGGCTGTCACTCAAGCTCTCCTGAACTT
>non-DHS123
TGCCTTTGTTAACCCTTATCCATTTGTTGACATGTAGACTGTTTACAAAAAAAAAAC
ACATGGTTAAATGTTAAATTTTAATTAAGGCCCCCAAAAATTAAATATAACTTTTTA
AAATGAAAGGAGTCACCTTTTACATGACTCAGGTGAAAAAACAGTATAAACATTAAT
TTACTTTGTGTTCAAAAGAAAATTCCAACTGCTGTTGGGGAAGGACACAGAAAA
>non-DHS124
GACAGGCTTTGGGTATAGAGTGCTGAGGAAACTGAAAGACCAATGTGTCTTTCTTAC
CCCAGAGGCTGGCGCCTGGCCTCTTCTCTGAGAGTTCTTTTCTTCCTTCAGCCTCAC
TCTCCCTGGATAACATGAGAGCAAATCTCTCTGCAAAAAAGATATGGGGCAGCACTG
TCCACAACAGCCTCTACTGGAAACAACCCAAGCACCCATCACAGAATGAATTAG
>non-DHS125
CAGCATTTCCCAGGGTTTTCGGTAATTACACTCCATTCAATATCTGAAGAATTACAA
TAATTCTACATGGAATATACTTGTGCCCTTCAGATGCAAAGCCTTTAGAGAAGTGAA
AAAACAACTTAAAAGTAAATTAGCAGGGAAGTTATCATAGTTACTATTTATTTCACG
GCTCCATATATCAAGGGCTAATTATACATTGTACGTTACATTTTCTGGGAGGGATTT
G
>non-DHS126
TCTTGATTCCCATAAGAGGAGCTTAGCTAGTGCGGCTCGCTGGCGCCACCTAGAGCT
CCGAGGCGGAAAGGCCGTCGCCTGCCCGGCTCGCGGCTGTCAGCGCGTGGAGACCGG
CTTTTGCCGCGAGCAGCAGCAACCAAGAGGATCCTGCGAAGAAACGCGACAGTTTGC
GTTCCAGCGGGGAACGAGCCCTGCTATTGGGGGTGGGGAGCACTACATGGCAGTGGC

>non-DHS127
GGTGATAATCTCGGCTCCAGATACTGTTACCTCCCTTTCCCCTTCTTGTGTGGTGTG
GTAATAGCTGTTAATTAGTTCTGGAGCATTACAGTATCACTTGTAGTTTCCCCACAC
CTTACCCACAGTAAACACCCTCTTTATTAAAATAGTATCTAATTACTCAATTAGTAT
GCATTCTCATTACACTCAAAACATTCAGCTGAAGTTTTCTTCCAGGACTTTGACTGA
>non-DHS128
GTTGGTTGGCTCATGTATTTTCAGGTTGACAGGCCTTTTCTGGAAGTAATGCCTTAA
TTCAAAGAATTATAGTAGATTTCTGAGAAAGTTACCCCAAATTTTATGAAAACATAT
TTACTTGGGCAAGATGGTCAAATAGAAGCATCTAGCAATTCTCCCCACTTCAGGAAC
ACTAAATTGAAAAACTACACAAAAAAGAACCTTCATAACAACCAAAAATCAGGTGAG
CAAACGCAGTACTTGGTT
>non-DHS129
ATATTAAGGAAAGAGGCGTTGAGGAGGGTAGAAAAGACAGTCTTGAATAATGTACAC
CACTCTTCCCTCATCCCCTGGTAGCAGCCACCTGGCACAAAGAGAGAATCTGTGTAC
TAGAGAGAGGGAGAGTGCAGTGACTGTGGGACTCAGTGCTGCCTTGTAGCAGTGGAA
AGCAATATGGGGCAGAACTCAGCTTGCACCCACAGAAGGAGCATTTAGACCAGCCCT
AGCCAGAGGCAAATCATCCAGCCCAGTAGTCA
>non-DHS130
GATTCTCAAGAACCAACCATCATCATAATTATGAGTTGTAGGCTCAAAATTGGAAAA
TAGGTAGTATTTTTGGGTGGTTATTTACTAGGTCCTCTGTGCAGGGAGAAATCAAAC
ATCACACATACTGGCATATCTGACAAACAGTGTCAATATTTTTTAAAGTACAATGGA
GAGTCAGAGTGGGAGAAAATGAGAAAACGAAGTTTAGAGGAAGAAGAAAACCAGAAA
TCCTTC
>non-DHS131
CAGTTTAGCGGGCAGAGAGTAGTTCACAGTGGGAGGGTTCACTCTGTTCATTCATTC
ATCCATCTCTCACTCCTTCCCTCCCCCTCTCTTCCTTATTGAGCAAAGTCACTGTGC
TAGACACTGCGGATTTGGTACTAAACAAGCGGAAATGGTCCCTGAGTGGGGAGTAAC
CCTCTGTGCTCCCCCAAGCCCCTGAGCCTGCGACCTCCTGGCACTGTTGGGAGTTCT
CTAGGGG
>non-DHS132
CTGTTGTCTTGACTGCCAGGTCTCCTGTGGGGCCCCTGGGGCCCTGTGATACAACCT
GTCACCCTGAGATGGACTATACTGACCTTCTACCCAGCCAGACTTTCCCCAGGGCCT
CGCGCCCACTTCTGTTCCCAGAGCCACCTGGCCTTGATGTCTGTATGCAGTGTATGG
GGCAGGGAGGGCTGGACAGGGCTTTTGTTGGGGAACCACAAATACCTCTCGTTAGGC
C
>non-DHS133
AGATCGGAAATCAACCAGCAGACCCCGCCCACCCCGACGCCGCGGCCGCCGCTGCCA
GTCAAGAGCCCGGCGGTGCTGCACCTCCAGCACTCCAAGGGCCGCGACTACCGCGAC
AACACCGAGCTCCCGCATCACGGCAAGCACCGGCCGGAGGAGGGGCCACCAGGGCCT
CTAGGCAGGACTGCCATGCAGGTGCGCTGCGCCACACGTGGGTTCGTGTGCATTTGT
GTGTGT
>non-DHS134
CTCTCTTGAGGCTGACAGGAAAAGCCCAGATCAGCAAACCTTTCCAGTAAAGAGCCA
GATAGTAAATATTTTCCACTTTGCATCCCAGAATGTCCCTGTTGCAGCTACTCGACT

CTGCACGTGTGGTGTGAAAACAGCCACAGACAATATGCAAATGAACAGGGCGGCTGT
CTTCCACTATTTCCTCTTTTAAACGTGTAAACTCTGTTTCCAAAATTAGCTGGTG
>non-DHS135
GATTTCTGTCTGGGGAATCAAGTTTTTGATTTGTGCTACCTAATGGAGGGGAAAATG
CTGAATTTCTTGCTGCTCTGTTTGAGAAATAGATGGAAGCATGGGAGGAGCCAGAGA
CCTCTGCAGCAGGATTTGGTCTAAGTAGAAAAGGAAGATTTTTGTTTCAAATTGCCA
GCTGCTTATGTCAGACTGACTCCCTTATTATGCCTCCAGTAGGCCTGTCAATATGG
>non-DHS136
TTTCTTTGCTCAACCACTCTTCTCTTAAATTCTTGGGATAGAAAAGAGGACTTAAAA
GAAGTTTCAGTGTGGAACAGACATTTCTTACCATTTTCCCCATTTCATCTTTTCCAG
CACAAGCCCTGTAGGTATAAAGTTTGACTTGTGATTTCAGAAAGGCAGACAGTGGAA
GAGGATGGCTGCAAAGCAGTTCAAGAGGTACACACCAAGTAAGCACAGGTTCTCTCA
TCAGATTCT
>non-DHS137
TAATGCCAGGCTGACAAAGGACACCCGGAAGAGCTAGCTGTGGAGGACCCGCCTCCT
GCTGCCTGTCCCCTCTGCAGCAGCTTGGTGAGCCTCGAGTCCGCCATGGGGTCAGGA
GCCGGGTGAGACCAGCCCGCATCCTCTTCCCCATCTGCACCCTGAGCACTTGCCTCC
TGGCATCCCACAAGGGGCCACTGCTGCCTGTTCCATAGATGGGGACACTAAGACCCC
AGGTCACAG
>non-DHS138
CTCTGGTGCTTCTCTTCAGCAGGTGCCCGGGGAATGGACCTGCCGATTCTGTGGGTT
GCAAGTGTCCACAGCCTGAATCTTGAGGTCCCTGGTCCATGAAGGGGTCTGAATGAC
TCCTGATGTTGCTGTCAAAGCATTGGACAGACAGGGATGCCAGGCCCCTTGCCACAG
GCCCGGGCAGGTGGGAGCCGGCTCGTGGATGAGGAAGCAGAGATTGTGTCTGAGA
>non-DHS139
TCTTACCCATTTGAGGAGTTTCTTCATTTACAAACCTCTCCTTCTGATAGACTTTAT
CACTTTTGTCTTATCATTAATCCCTTTCCTCTTCTCATCTAGGTATAAACCCCTGTA
TACTTTACACCTTCTTTTAAATGCTTAATATAAGTAACCACAGGAGAATCTCTTCTC
CCCTTGAAAAACTGCAAATAAAGATGTCAGCTATTGAGTATATTTGTGGCGTCTCCA
G
>non-DHS140
CCTCCGCTCCTAAACCACTTGTGGCGCGTGTCGGGAATACAGGGCGCTCTGGCTCTT
CTTGGGGCCTCCGCTGGTCCGACCATCTGGCAGCTCTCTTAACCACCACGGCCACCC
CACGAATGGAAGTTTGGGACGTGCCTGGGTTGCCCTTAAGGAGGACACCCCCGCCCC
CACCGCCATTATGCATATCGTGGGAGAGGCGCGTACAGGGCTGAGACACTCCTCAGG
TCCCAGAAC
>non-DHS141
AGTCCTACCAGGCCCAGACAGGAGGCACCAAAAGGACGCCAGAGCCTTCACCACCCA
GCAGGTTCTGTACCAACTGATAAAGTTGATAAAAGTGACCCCAACCCTTAGTAACCC
ACTGGTGTAATATAGAAACATCTATAAGGGCGTATGTGTGCGTGTATCACCCTAGCC
ACGTACATAGTCCAGCAGCCAGCTCTATTGAGAGCAATCGGCCACTTCACTTCTCTC
CCTAGAACT
>non-DHS142
ACATATCTTCAGGGCTCACAGAACCCTCTGGACCACTTCCCCAGGCTTCAGTTGCTC

ACACACTCTTTATCCTTCCCCAGTCTCCTGCTTCCAATCAGGATAGAGGTCAGTACT
AACATCCACCCCCCTATAGCTTTAGGCAACTCCCTTCCCTCTCTCTGCTTTTGTCCC
CCCATCAGCACTGGGCTAAGAACAGTGTAGGGGAGGCATCCCTGGGCCTCCTCAGGG
GTGACAGTCTGATGTGACCTTCCTCCACCTTTC
>non-DHS143
TCCAGTCCATGGAGGGAGATACTCATGTATTTGACATTTGAAAACCATTTTTTTTAG
CAACTATGATAGTGGTTTCTAGACTTTGTCCTTAAGCACAAGCCATTCTTCCTATAT
GACATGATCATAACAAAGATTTATATATAGCTCCAGAGAATAGTGAGATTTGCAGTC
TTCTGATGGATATCAAAGTTGAAGGCTAAAAGACACTTTGGTGGTATAGCTGGG
>non-DHS144
TGAAGGCTAAAAGACACTTTGGTGGTATAGCTGGGAACAGATTCTGTAACAGAAAGA
AAAAAGTATGCTCAATCATTCACCATTTTATTATGAAATCTAGCTACGTGCTATGAC
ATACATAATGAATGTATGTCAATACACCCATTATTGTCACTTCATGTTTTAAGTATA
GAGATACTGTATAGGTGATAAATAATGCATTTGTCACCATTAAGTAAGTCATCACAA
ATGTGCAGTCA
>non-DHS145
GTCATCACAAATGTGCAGTCAAACTTTAGAAATGTTTAGATCTAAGAATCGTTAACT
TTCATTCTTTTGCCATCTTTCCAGCCAACTCACAGCTACATCAAGATCTTGGAAGCT
ATATGACCCTACAGTATTAATAGTTGACAAAATAGAAATAGATGGTCACGGACTTGG
GACTGTGGCTAGTAGGTGGCAGAGGGTACCGTTTATAAGAGAAGTTTGGGCTGTTCC
TAAAC
>non-DHS146
CTGTGGTCTATGCAAGTCAATTCCTTCTGAACCTCTAGGGCACTGCTCAAGTTGCTT
AGCCTAACCCTAGTGGCTGGGGAGTGGGAGGTGTCTGAATTTCCCCCACCCTCCTTC
CAGGCCAACTCCTTCCAAGAATCACTCCAGGCCCTTTCAGAAGATAATTTTGATTGC
ATTGTATTTCAAGGGTTCTAATTTTATTTCTTCAGCTAAAAAATAAGCTCCTTCCTG
GGACAGTTCTATGTCT
>non-DHS147
TTCCTGGGACAGTTCTATGTCTTGTTTTTCTTCCCGCCCCCCCCCCGCCCCCACAGT
CCCTAGCCTAGCTGGGCACAAAATAGATTATCAATAAATTCTTGCTATATAAATGTC
TCCACCAGGGCCAGAGAGCTTCACTTGAGGCAGTTGCTGACAAACTGGATAGTCCAG
GGAGGCCTCCACAGCTCCTGAACTGCAGTCCTTCGATTTTTGGCCCAAACTAGGTCA
TGTCCTGAAGG
>non-DHS148
AAACTAGGTCATGTCCTGAAGGGAACAATTAGTATGAGTGTCTGGGACTCTTTCCTA
TTTCAACTTTACCTGCTTTAGTGTACAAAAGAACCCGAGAGATCATTTAATCCGGGT
TTTAAAAACCCCTAGATCTTGTCAGAATTGAAAACATATGAAATAAAGTGCAGCATT
GCTCACCAATCAATTCACCCAACTCTATGCCCAAGAGGTTTCTACCAAACCCTT
>non-DHS149
AGGGCCTGAGCACATTTCAGTCTGTTATGCAGGCTGAGAGGCCAGGCTCCTCCAAGG
CCCCATGAGCTACTGGTTGTTGCAGCCCTGTGAGGCAGAGGAGCTGCCTGCCTGCTT
TTTTCTCCTCTTGTTGAGGAGCCGGTTGTTAGAGGTCTTCAGGTCCTTGATCTTCAC
CTGGTCGTAGTCTACCCGCATAGTGGCCAAGGCACTGGTCATCTCCTCCTGGGGACA
GAC

>non-DHS150
CTCCCAGTGGCCTAGAAAGCTAAGGCCATGCACCAGGGCAGGACAGCACAGAGGCCT
CACTCGGGCCAACCTAGCTAAAGGTGCTGCTATGGCAGCAGGCAGCTCCCAGCCCCT
AGGCCCTGCCCATCTCTAGGGCTTGAGCCTGGGCCAGAGATCTGAAGGAGGTTTAGG
GTCAGGGTCCTCCATCCTGAAGTTCCGAGTGACAAATTAAAACACAACAAAATAATC
CTTTTGTTAAAAGAATGACCCTGGGCC

>non-DHS151
CAAGGAGGAGTGAGAATGAGAGGGCCAGCCAGTGTGTGAGAAATCAGCCTCCCCAAG
GCAGAGGTGAACCCTCAATCGCCCCTCCTCCCTTACTCGCTCACTCAGCATTCACAG
GCAGCAACGTGGTCCCTGGTCACCATGGCAACCAATGGCCAATGCCTTTCTCAAGGC
CAGGCCCATCTGCCCATCCCACAGGGAGATGTCTCTGTGGAAGATGGGAAGCCCAGC
CACAACTCCCAGTGGCCTAGAAAG
>non-DHS152
AAAGTATGCTGGCTTTTGTCACAGTGACATTTTCCAAAGTGGAAAAAAGTTAAAAAT
ATCTCCCACCTTAAGTTCAGATGAAAAGTCACCCTTTGGGTCGGGAATAGTGAGGGG
ATGGCTAGGCCCAGGGACTCCCTCAGGGGAGAGCGCTGAGTGCCATCTGGGCTGGGG
CTGCAGCAAGGCAGGGCTGGACTGCACAGGGAGGCAGGCAGACCGGATCCAGCAGCC
TCAGGGCCCAAGGAGGAGTGAGAATGAGAG
>non-DHS153
CCACAAGTGAACCTCCTCCAGGCGAGGGGATGCCCTGCAGCATCCAGCCGTCAAAAC
CTCCTGTAGAAACGGGAGCGAGCATCAAGCCTGTGTGTGTGCATGCTTGGGCGGAGT
CTACCCGGAGGAGAAAGAGGCTTCCGGGCTGGCCCTCCAAAGCCGGTTGTACAGGCG
GCAGCTCAGGCTGCTCACTGCGGGCCCTATGACCCTTTCTGGGGCCCTGCTTGCTCT
CTCCAGTCTGCTTTT
>non-DHS154
CTTGACTTCACATGACCAAGAAGCTAGGTAATCCTGGGGCATTGAAAGCTGCCCAAG
AAAACAATTATGCTGTTTCTTCACTCTCATCCTGGCCTCAAATGAATATGGGAAGTA
GGAGACAATGAGGTGATTTTTCAGGATTTACTAACATTCCTAATTTCGCTATTCTCC
TTCCTTTAGCACAGTTATCAGAAACCATTCAGTCATACCTTGCCTCCCTGTTTTCTT
CTGCCATTGAAATAACAGGGGCCTAACT
>non-DHS155
ATCTGAGAGTGCCATCTGCTTTACTCAGTCTACTCAGGTCACATATGAATCTCATGC
AAAAAATGCCCTCACGGAACCATCCAGGATAATGTTAATATCTGGGCACTGCATGAC
ATAGTCCAAGTGGACATCTAAAGTTAATCATCACAGCTTCCTTTACAAATCAAAGTA
ACTGTTGTATATGAATGCAGCAGAAAACTTGACTTCACATGACCAAGAAGCTAGGTA
ATCCTGG
>non-DHS156
GTCAAAGGCCTGAGAATCAGAAGAACCAATGGTTTTCTTTCAGTCTGAAGGCCAGAA
GCCTCTGGGCCCGGGAAGAACCAATGTTTCAGTTTGAGTCCAATTTCACAAAAAACT
AATGTCCATTTCTGAAAGGCTATCAGACAGGAGGAATTCTGTCCTACTCTGAGAAGC
CTTTTCGTTCTATTCAGGCCTTCAATTGATTAAATGAGAGCCTCTCACATCTGAGAG
TGCCATCTGCTTTACTCAG
>non-DHS157

GGGCATCTGGGTCAGCTAGTAGCTGTTAAAAAGATTAAAGGGAGGTGAAGACGTGTA
GCACTGAAGGCTGCACTGGCTCACTGATACCTTTATTTTCTCAGAATTGAAAAGTTT
TTCATAGTTGTAAAGATAGTCAATACCATAGGTTTCACTTTTACTGCAGGAACTTTC
CAACGAGAGGTTTCTCTCGTTCTTTCTTTAATTTTATCTTTCTCTCACTAGGCAATG
CTGCACTTAACAG
>non-DHS158
GAGGAGAGGCTTCACCAACACGGGGCTTTCATCCACAGCGAGCGCAGAGCGGGCTTT
GCCAGTTTATGGTCCTTTCCACTTTGCCTCTCTCTTTCCCTCGCTCAGTCTCTTTTC
CCTCTAAGACTTAACAAAGCCCTCATAAAACCCAAGCAGAAGGGTGAAAAGTAAAAG
CAACACAATTTCAAAAGAGAGAGTGCAAATAGCAAGCCCTCTGGAAGAGCTGAAGAG
GAAGC
>non-DHS159
CATTAATTCACATGAAGGCTGGTTGTTTTTTATGCCACCTTCTCCCCTCTCTCTCTT
GCTCCCTCTCTTACCATGTGACATACCTACTCCCTCTCTGCCTTCCACCATGAGTAG
AAATTTCCAGAGGCCTCACCAGAGCTGAGCAGATACTGGTGCCATGTTTGTACAGCC
TGAAGAACTGTGAGCCAAACAAATCTCTTTTCTTTACAAATTACCCAGTCTCAGGCA
TTTCTTTATAGCAATGTAAAACAGACAAACGCACC
>non-DHS160
TAGTTGATGCAAATCTCAGACAGGTTTTTTTTTTTTTCTTTTTTAATGGTCTACTAT
TCTTTGTTAACTGAGACTCCCTGTTCTCCCACTACCCAACCCATAGGTGGCCTGCAG
CACCTGGTAAACAGAAAGGATAGATTTAATTGAAAGACTCCTTTTATTGCAAGGCAA
TTGACAAATGTTACCAGAAAATAAACTCCCACCATTCCCAAGGGAACGGATTTCAAT
CTGGTGGAACAGGCTAGACTACACGGAAC
>non-DHS161
AGGACAGAAGGGACAGGATATCAGCTAGATGTCATTGAATATATTTTGTTTTGTAGA
TTTGGCTTTTCAATCATCTACTTGTTTCCATGACTGTAACACTAATTTAAATTTAAA
AGATTCAATGCTGAAAAGCCAAACTCAAAATACAATAAGTCTAACTAAATATTATTG
GTTGCATAACCACACAGAAAATAGAAAAGTTATTCTATTCCTGATGACTTAGACTCT
CCTTGAA
>non-DHS162
ACACATTTCCAGTTGAGAATCAGGCACTGACTCTAGCAACCCATGAACTCCCAGAAT
CTGAGATCAAGTTTTCCAAGTGATAGGGTTGAAGCTCTCTTGTTGTTTTGAGGTGAC
TAGGAAGCACATTTCCCCCCTTTCTATAGAGCTGGAAAATAGCAGGGGTCTCTGACA
ATTCTCTCCAAAAACCTGTTCTCTCAAAGTCATCCAAGAATCACTCTCCCCTCTGCC
TCTTATACAGCCTTGT
>non-DHS163
CACTCTCCCCTCTGCCTCTTATACAGCCTTGTGGTAGGTGGTGGGCTAAGGTGTCTG
ACTATTTTAGGGTCATCATTTAGCAAGTCCAACACAGCTTCTCTGATCTCTCTTTTA
AACATGGGAAGAAGTATCCTCTTTTGTTCTCAGCTTTGGAGTTTCAACTGATAGTAA
AGGTGCAGGAATTGCAGGCCTGATGGTTGAACAGCTCTGGACAAATTTCGACAGCCA
TCACAGCAGACTAGACCACAGA
>non-DHS164
CACGAGCTACAGAAAAGTGGAACAGGATATAGTCACATTCAGCCTTCAACAAAGAGC
AGCTTATAGTTATCTTTAACTTTTCTTGAAATCTATATAAATAAGGTGAAATTTTGT

CTCTTTATCTGCTAGTACATAACAACAAAAAAAGTTGCATGATAAATTCTTAGCAAG
CACAAAGGTTAAGTTTTGGATTGCCTAACTTAAAACATGAGTGAGATGACTTATGCT
CATGTGGAGCCCT
>non-DHS165
AAAGACCACATTGAGTGTATTGGAGAAAAAATTACTTATTTTTCAAGAATGAAGAAA
AGGAGACATATTCCAAGCCTGCTTGGTGCCTTACATGGCCTTTAACAGCTTCTCAGA
AAAGAGCACTCCAGGAATGGGATCAGTTACACTGATCACCATTTTCTCTACAGTGCA
AAAAAAGAGGATACTATGCCCTGTTTCTAATGCTGCACTGAAAGGATCAGGTAAGA
>non-DHS166
TGCACTGAAAGGATCAGGTAAGAGAGAGTTGCCATCTTAGTCTTTTCTACTCATATG
TGGGAAATAGAATATGATAATGAGGTGGCATGCAGAAAAAGAAGAGATTACCTAGTG
ATTTCATATGAAACCATCCATCCCCTTCACAATTGTTAAGTACCTAATTGAACAAAG
TTCTGTAACAGGTATTTTACCAGAATAACTGTGTAGTACCACCTAGAGACTTATGGT
TCAGGCCCTTAT
>non-DHS167
GGTAAGTGGGATTTGGGTTTGTTTTTGTAAACTGAACTTTTCAGTAAAACAATGTGC
ATAACTCTTAATAACATACTTCTTATTGAATTGTTAACACTTTAAAAGCAGCTCATG
CTTTACTACATATAGAAACTGTTTTAATGCTAATGAATGTTTGAATTTTGGCTAATA
TTTTTATATGATGACTAGAGCATTATTTTTCCCAAGTTTTTATATAATATAAATAGA
AAGTTTGGGCATTCGATACTAG
>non-DHS168
GCAGATTGGAGCTGTCCTCAGGCATCCCTGGAGGCCCAACGAGCAGCCCGGCCAGGC
CCGTTGTGGTTGACAGAGTTGGTGTCTGCCCACCCGAGGGCAGGACAAGTGGATGAC
CCCGAGCAGAGCCCCAGTAGGGAGCTGTAGGGGGCCTGGAGCTCACCTCTCATTCTC
TTACTTAATGGAGAGAACTGCCCACCGCTCAGAGGAAGCGAGGCACTCTCCTGTCCC
GCTCTCCCTGTGGGCTACTCAGAG
>non-DHS169
CCAAGCACTGGGGATATAAAAGTGATGAAGGCACAGTTCCTGCTCTCTAGAAAGGAG
CCCATGGATTTGGCCTTCTACCTTTGGTGTTCTCAATTCTGCGACAGTCTCGGCAAA
AAACCCAACCGGGCATAAAGACTTGACATTCTGCACAGGAAGGAAGCACGCAGGGAC
AGAATGTCCATTTGTGAGAAAAAGGAGATACAAAAAATAGACATCCCTGGAGGGCTC
CTCCTCCTTCTTTCAATTTGATCC
>non-DHS170
CCCTCCCTAGAGAAGACAAGACTGTTCAACAGCATTGCCACCCCATCTGCCAGGTGG
CCGGAGGGAGCAAAGGCACAGCCTCCCAGGGGCCCTCTGCTCTGACCTGGCTGCCTG
GTGGAAAAACACTACAAATCTGGTAGGAGGTGATTTGATTTTCTTGCCTGGTTGCGA
TGCAGTTATGGTGTTATAAAACAGAGGGTCCGAAAGACTCTCACAGGGAAGCAGAAG
GAAATGGTCTTTTC
>non-DHS171
TGCTTAAAAGAGAGGCCTTAGGAAGGGAGACACAGCAAAACTGGAATCTAAGTTTGG
GGAGTACCTCACACGGCGGATTTGACTCTCAAGCATCATCCGTAGATTTGGTTTTGT
GTTACTCCTGGGTGTCTTTGTCAGTTCCTCCAGGGGTGCCATGGACTTGCTAAAAAG
GTGCTGGAATCAAAATTTTAATTCACGTATTTGTATTGATTTAGTTATTCCTCAGGC
CCCATCTACTTGCAAAGATTTCGAG

>non-DHS172
CATCTACCTCATAATCTCGTTTTGGTCTAGTTGTATCTTTAACAGAAAAAAACTTGG
GAAAGTGTTTAATATTAAAAAAAAAACCACAATAAATCCATTTTCTACTTACATCGT
TTTGTAAAGTTCAACAGAACAGATTAATTTAAATCTGAGAAAGGACAATAATATGGA
GGATGGAAGAGTAACTGATAAAATAAGTACAAACCATTAAAAAATGGTTATATCTAT
TTTTCCCACACACAGTGACAGG
>non-DHS173
GTTGTGCCATTTCATCAGATCTTCTTATAGTCAATGTCACATTAAATTAGAATCTGA
GTAACTAATGTTTAAAAATAGCTGATATATTTGAAATTTAGGCAAAAAACTCATTTT
TATTTGTAAAATGTGCTCAGTGTTAACTTTATTGATAATAACCAAAAACAAACCTAA
TATTTTATGATTTTAAAATTATTTTTAAGCACAAAATAGACCCATGTTGGGGATGAA
TAACATGTCTGAG
>non-DHS174
AACCTCCTCAGGGGATTAGTTCTCAGGGCCTGGTCCGCTAGAAGCCAAATGCCTGTG
CCCAGCCACCCGTGACGCCTGCAACAGGCAGGAGATCCCCCAACAGTTACTCCCAGC
CTTCATTCCACAGGGTCTGGTTTTCCTGGAAGGTGGGAAGTCCCAGGGTCTGAGGAG
AGGGAGCGCAGGCCCCCATTTGTAGGAGTGAGTCAGCTGACCCGCCCCCGGGGTTCC
TAATCTCACTAAGAAAGACTTTGCTGA

>non-DHS175
GTGCTGGGTTCACCTTTTCACTCTGCTGCTAGCCAAGCTCACAGCAGCCCTCAGGAG
CCCTGTCTCTCCCAGCTGCTCATGAGCTTGTTTCTTTGGCCTGACACCACCTCCTTG
CTCCAGCCTCAATGTGTGTGTTTACTTATTTTTAGAAGTGGGGGTAGAAAGTGGAGT
CCTCCCTGCATCTTATGAAAGGGGCAGTGTCTCAGAGCTGTATCCATCCCGTTTCGC
CATTCTGTGGGTGGAGGGTG
>non-DHS176
GAGGGTGTCCCCTGAGAGTGCCAAGGTAGGCGTCTGGATTGTGCCATCCACACACCC
TCTTCTACAGCTCCAATGGAGTATGGTGGGAGTGGAGTTAGGTGTCAGTGCTCAGTT
CCTCAGCCTACCATCTTTGCCAGGAACTCCCTGCCCATCGCTTCTCCATGTTCTACC
CTTTGTCTTCCTCCCCCAGTGCCACAGACCCCTCAGCCCCAGTCCCCATGGCCTGTG
TGTTGTTGCTGAGCACAGT
>non-DHS177
CATTCCATGATTATCCTCCTATGGCTCGGACACTGTGGACTGCCGACCACTTCTCAG
CACAGTGGGCTGGACCAACCCTCACCCTGTAGGCCCAGCAGCACCCTGGAGCCCAGC
GTATCCACATCCCACTAATATGAGGGGCATGCAGTCTCAGTGTAGTTGTGGAGCCCC
TCCTTCTGGCCCAGGTGCCCCCCGTCCACCTGTGGCAGCACAGTGTGCAGGGGGCTA
CAGACCACGAATGGGTC
>non-DHS178
AAAGAGTTGGGTGGCACTGAAGCCTGGTGCAGGGAGGGGCCTGCCAGGGACTGGAAC
TCGATGTTTGGGGACAGTTTCTTGTCCCCAGGGTCCCTGGGCCACAAAAAGCTGCAG
GGTCAATCCAGAGACCCCAAAGCCCTGGCCTCCTGCCCAGCCCACTTCACACTGCCA
CCCCTGCATCCCTGGGGTCTGCTGGGTGCTTCTACCTCCTCCTCCATCCTAGCAAGC
TCCTATTC
>non-DHS179

CTCCATGGCACATGAAAATGACATGGAATTCAGTTTCAGTGCCCGTGAATGCCTCTT
GCTGGCACCCAGCCTCATACACTTGCTTACACATTGTCAGGGGCTGCACTGGTGCTG
TTAGGACAGAGGCCATAGGCCTGCAAAGCTGGTCCTCTCCAGACAGGCTTGCCGAGC
CCTGGCTCAGGCTCTGTGGATCCATGCCGAGAGGCCAGGGAGGCCAGTGAGGTGCTG
AGGAAGGAT
>non-DHS180
GAGGTGCTGAGGAAGGATCGGCCCCATGCCCAGCTCTGGGCTCTGCCGTGGGTTCCG
GTTTCATCTGATGTGATGGGGAATGTGGTGTGGGACAGAAGAGGACAGCGGAAGGTG
ACACATGTAGCTTTGCAGGGGCTGTGGGAGGAGTCATGGGGCCTGGGTGGAGGTGCT
GGAGGCTGACGGGCCGAGTTTCCGGAGCTCAGAAGGCTGGGGAGGTAAAACTGAGCA
TCATCAGCCAG
>non-DHS181
CTCAGACAGGACACAACAGGAATCCAAAATGGCTTTTGGGAGGTCACAGGGGTGAAA
GGACGGTGGCTTGGGTCTGGGGGGAAGGAGAAGGGCATGGAGACAGGGTGGGACAAC
TCTCCCAGTAGAGCTAGGACACACGCCTCCGCCACATCCCAGGCACCCTGCCGTGCC
CTCCGGCTGCCTGCTCCCTCGGTCCTTCTGGCCACGATGGGGGTCGGGGGTCTGGGT
TCATCTCCATCT
>non-DHS182
AAGGCTCACCAGAGGACTTGTATCCTCCATGGAATATAGACAGCCTGGCTTTGAATT
TTGGTCTTGCCAATTACAAGCTGTATGGCTGTGGGCAGGTTATAGAGCCTCTCTGTG
CCTCAGTGTCTTCATTTGTAAAATAGGATAATAATAAAATATCTTGGAGTTGCTGGA
AGAGTTAAATGGAATTAATACATTGTAAAGTGCTTAGAATAGTTCCTGGCACATG
>non-DHS183
GGTTCGATTCCTAAATCACAAGATGCCAGGCAAAGAAATGCTACACAAGGGCTCTTT
GTCCATGGGAAAGGCACACGCTACAGAAAAGTATTTTAAGAAGCCAGCAATGACTGC
AGTGCTGCACGTAAAATGTCATCATGTTCTTAATGAGGCAATTGCTTGGTCAGTAAA
AATACTCTCGCCCTGGGTGGTTAGTATGTTGAAGTTAACTTGGACACTGCATGTGGC
AGCCACAGCCATCTTCCCACAAG
>non-DHS184
AGCCATCTTCCCACAAGAAGTTAGGCTGCATGTAAATAAAGCCATCACCTACCCCCA
AGTGAATCCTATCTCCTTCATTGGACATGGAATTTTCCTGGGAACCTGTGTCCCTGT
GCTTCTGGCGAGGTGTCTTCTTTCCTTAAGTCGAGTGCACCATTATTGTGTCTGTTT
GGAGTACTGGATATCAAATTAAACTGTGTGAGTGTGTCTGCATATCTCTGTCTGTGT
AAAGGGATTTGGAA
>non-DHS185
TAGTGCTTCAGTGTACCAGGTTGGTTGGTCCACTCATTCACTCATTCATTCCTTCAT
TCACTCATTTTGTGAGGCTGTGTTAGAATAGGAACAACACAGACTTCAGAATCAGAG
AAACCTGGAGTTCAAATCCCAGCTCTGGCTTTCCTTGCATATTCCCTGATGTCTACT
TATCTTTTAGCTGTACTGAAAGTCATATGTCAAGCATCCAGCACAGGACTCAAAGCA
TTAA
>non-DHS186
AAACTGAGAGAGGGTGAAGAGAGGCAGGTTTCCAAGTGTGCACTGTTAATGCCTACA
TAATCAAGACTCAGCTGTTCAGCACTGAGTGGGAGGGTATTTCTGGGAGACGGGTGG
AGCTTTTCTCCTCCTTGTTGGTGTCTTCACAAATTCCAGCAATCAAAATAGTCATTT

TCCTTCCTTGCCAATGTCATCTTAAAAGAACCTCTCTCTAAACCCAGGTGCTTAAAT
TGTCCAGTGAG
>non-DHS187
TGCCTGGTCAAACCATTTAACATATGACCATGTAAAAATGTAACAACCATTCTTAGC
TCACTCATGGCTGGCCAGATTTGACCCAGGGGCTACAGTTTGCCAATGTTTATTTGC
TAAATTCTCTCCCTTTGTTGCATCATTTGAGAGAATGATATACCATTTTCTCTAAAA
AGTGAGGTGTCATTTTTTTCCCATCTAATGTCAGGCTGTCATGACTCAGTTACTT
>non-DHS188
GCTGGGAACCAAGCAATAAATACTATTTATTTATTTTAATAGTATGCTTAGTGGAAG
TATTACCAATATTTCCTATGCTTAAAAACATCCCCAAATGCATTTTGTGTTAGGCTC
AGCATTTTCATCGTAATTTACAACTTAAAAGGGTAAATTCTATTACTCTATGTCAAA
CCAACAACTGGGTAAGTGCAGAATGTTACCCTGTGAACTATGACCAAATTTATTGCT
GCAGTCATTAGTACATATTGATTCTGCTTATAACAACCCAAAGGG
>non-DHS189
TTCCAGCGAAACACAAAGGTAATTTTTTTTTCTGGTTAATATTTAGCAAGAATTCTG
CAGAGTGATCAAAAAAATCAAATACTCAGTATTTCAGAAATAGATTAAATAGGTTAC
TTTTTTACTGATAATGTGAAAGAATGATATAAAAACTTGATTTTCCTCAACAACATT
ACTTTCTTTTGTAAATGTGGTTTCTACAAAGATGAAACTACTAAAACTTACAGGTTT
TATTTTTTTCTTGTTTTTTTTGACTGA

>non-DHS190
AAGTCTGAATAGCAGCTGGGTAGGCACAGTGCTCTAAGATGTCCTTGAAACTACCAG
AAAGAAAACTGAGAATTTTGGGCATGAAACTTTACTGCTACAACTACTGCTACTACT
AGTAGATGACATTGAATGTTTTCAATGTACCAAACATTGTTCTAGCTAGTTTTCATG
CATTATTTCATTTAATCTAATAACTCTATGGGTAGGTATTATTTTCATTTACATTTT
ACAAACAGGAAATCTAAGGCTTGG
>non-DHS191
AGTAAACAATGTGGCTTTGCTGTCTTTGTTTTGAAAAGAATTAAATTAGAAAGGTGA
CAGTCTTCCTAGAGTTAGAAATTAACCTCGAGGTCAGAATAACTTAGCACTTATTAT
TTTCCCCACATGTAAGGTTGCATCTGAACAATGCACATGTCTGTTCTGGGTGTTTCA
TTGAATATTCTAATTTGAGACAGCCAGAGCCTGTGAATAACCTGGTTACTACCCACT
AGTGTGTGTGTTGTGTGTGTGTTT
>non-DHS192
GAGGGGAGATTTAGAGATGGGAAGAAAATATGACTCCCAGTGACTGTGGTGGGAGAG
ATGACATGTCTGGAGGAGTATGGAAATGCACAGATTTAATCTTGAGCAAATCACTGA
AATTCTAAGGTGTTTTTGTTTCCTCGTCTGTAGAATGAGAGCATTAGACCACTAGAT
AAAGCCTAATGGCTCTTGTAGCCCGTTAGGAATTTATTGATAATCACTTCCTGACCA
TCATGCTATCCACTCACAT
>non-DHS193
GTTTGGGGACTGCTCTTCTAAAGGGATAAAACAGTTGCCCTGAGTGGACTTTTTGAA
GATGTAAAGCAAGGGCTGGGGAGATGTCCTGAGCACCGTGGGATAATGGCCTTTGGA
TGGAAACTGAGTTCTGTCGTTCATTCAGCTGATGTTTAAGGGCGCCCTCCCTGGGCA
GGCAGGGTGCTGGTTCTTGAGGGTGTGGACACCCAGAGCCAGTCCCTGCGCTCTCAC
AGTTTTCCCTCTGGC

>non-DHS194
GCTCTCACAGTTTTCCCTCTGGCTGCAAGCGCTGGGGTGTGGCCCTAGCTGGTCCGT
TGGGCTCTGCAGGAGTCTAAGCTGGGCTCGGGATAGCCGGACAGACCCCAGGCCAGG
GGAAAGCTCACTCGTGGCTGTCGCATCTCTCCCTCCAAGCAGGTCGAAGAAGGGGGC
AAAGCAGACACCCTGAGCTCCAAACTGCAGGCTGGGGATGAGGTTGTGCACATCAAT
GAGGTGACTCTGAGCAGCT
>non-DHS195
ACATCAATGAGGTGACTCTGAGCAGCTCCAGAAAGGAGGCAGTTTCCCTGGTGAAAG
GATCCTACAAGACCCTCAGGCTGGTAGTGCGCAGGTAGGTGGCAGACCCCCACCCTG
TCCCTCCTACCACCTCACCCCACCTCTCTCCCTACCCTACCCCACCTCTGGGAAAAG
AGCTCGGGGTTGGTAGCTTGGCTTTAAACCTCCATGTATGGATCCTGCCTTTTTTTC
TTTTTTCTTTCCTATCTTGTGGGATGTGG
>non-DHS196
TTCTTTCCTATCTTGTGGGATGTGGGGAGGAAGTGGGGTGAAGACATTTCAGACACT
GCTGTCTTTCAGAACACTCATTTCCCAGCAGGTACTGGTTTGTCAGGGATAGACCTC
ACTGGACTACCTAAATTTTGGGACCTGATGAAAAGTGACACTCATCCCAGATGCGGT
CAGAGCTCTAATAAGCTTTTTGTCTGGTGGGACTAGAGAAAGAAATTGGCTTGGAGC
TT
>non-DHS197
ACTTCAGGCTCCCCAGTAGTTGGGACTATAGGCACACACCACCACACCCAGCTAATT
GTTGTATTCTTTTGTAGAGACGGAGTTTCACCATGTTGTCCAGGCTGTGAGCAATTC
ATTCTTAATATAGCATCTACTCAAATGTAGACATTCTCTGATTCTTAAAATAGGATT
TTATGAGGTTAATTTTTCTTTGTATCTCATTTGCCTTTTACAGATGATCTAAAATCA
TTTGGCAAAGGATGAGTGAAGTTATTC

>non-DHS198
GACAAATAAGCAGCTTTGGTTCTGCAATATAACCTGGAAATAGTGAGGACACCGACG
TGGAGAATAGAGATGGGAGTTGACAGGGATCAATTGTTCTAATGCGGAATAATGCAA
GACATAAGATCAAGCCAGCAGGAGTGAGAAACAGAGATCATATCAGGGTGATATCTG
TGGGGCAAAAGTAACAAGAAGATAAGGTGTAGGCGGTGGAGGGTAAACCGCGAGGCT
TCCATGAAGAAGAGTGGCCTGGGAAGT

>non-DHS199
TGTAGCCTGGTGTGTAAAGCTCATTTAGTGAAACCTCAGAGCTATTATGTGTCAACT
TTTCTACAGTAAGTATTTTGCCACATGACAAATGTTCGGCAAATGTCGATGGAGTAA
CTCTGACATGATTTTCAGGCAGTTAGAGGGTCCATGCAATGCAATCAGTTGCAAATG
ATGAGGTTAAAGTAATGGTACTGTACACACAGTACTATCCTTCCAACTAGTAGGTTA
TTAACACCTATGTACATCTCCAGCTGCT
>non-DHS200
TATTGGAGAATTCATAGCCACACCAAATCTATGAAGAGTTTCAAGAGTGTTTGTGTT
TGGCATTTTAAGAAGAAGGAAAATATAATCTGCTAAGAGATGCCAGAACTAAGGGTC
TGATCAGCCCTTGAAAGGACAAAGCAAGGGCTTTTGAGAAGTCTACATCTCTGTTCT
ACATATTTCTTCAGAGAAAGAGTGACTTTTGTAAGCATTTTAACCATCCTACAGTTC
AC

>non-DHS201
TCCACCACAAAACAGGTAAGTAGAATTAAAATATAAAAACAGCAAAAACACACACAA
CAAAAACTGTTCCACCTTTGATATAAATCCTTTTTAACATCTACTGAGCAAGTGAAC
TGGAATCATCAATAGTCTGTATTAAAGTAAAAGAAGTTTATACTTTAAGAATTAGCA
TTTTTAAAAAAGACTACTATTAGAATAACAGAGTGCCTTCAAACATACATAAAATAA
AAATGGGTATCTACTTATGTGATCTGAAGCACACACC
>non-DHS202
GAGGAAACCTGCCCAGTTCAGGCTCCTCCGTGGGCTTTGTCAAGTCCTCCGAGACCC
AACATGGCTGGAGACAAAGAATTCCCTCCAAGCTAGAGCCTGTACAAAGAGGGTAAC
TTAAAGCAGTATTGTGAGAAAAGTGGAGGTGTGTAAGATCTTATAAAGAGGAAAAGG
AAAGGACTTAATAATTCAGTATTATCTTCTGATATAATCAGGTATTTTTATGGGGCA
GTCTGTGCTGTTAACCATAAGG
>non-DHS203
GCAGTCTGTGCTGTTAACCATAAGGAACATCAACTGATTTTATAGATATTTGAAGAG
TCGATGTAAAACAACCATTAACCATCACTTTCTTTTATTAACAAAAATAAAATAAAG
TCTTAAGTATGTACAGCCTATTATATTCAGCTTTCCTAGGTATACTTGCCTCTTTTG
ACTCCAGCCCACCCCACCTAGCTACACAGAGCTTACAGACAGGTCTTGGAGGATCTA
CCCTATCCCTATTCTGGCTTG
>non-DHS204
CTACCCTATCCCTATTCTGGCTTGGGCCAGAAAAAGACACTAACAAAGAGTGAAGAG
GAGGAAGAGGAAGATGGTTATAGAGACAGTGATGAATGGTAGTGGTAAGGGGGTGAT
GGTGGTGATGGTGTAATGGAAGTTAGTAAGATAAATCAATTACAATAATTGGATACT
TTAATCTTTTAAAACAATATCCTATAGATAGTTTTAAGCATTCAATAAAAGGTTAAA
TTCCACCAGTTTAGCCTCAGTCTTTCCACA
>non-DHS205
GCCTGTCTTTACTGCAATCTCTACACATAAATTGTGAAGATTTCGTGGACACTTATC
ACTTCCCCAATCAATACCCTTGTGATTTCCTATGCCTGTCTTTACTTTAATCTCTTA
ATCCTGTCATCTCATAAGATGAGGAGGATGTATGTCACCTCAGGACCCTGTGATGAT
TGCGTTAACTGCACAAATTGTAGAGCATGTGTGTTTGAACAATGTGAAATTTGGGCA
C
>non-DHS206
TTAATACGTTGTTCAAGGCCACAGGTAAGAATTTTATGAAATTGTCATGGAGAAAAT
TGGGCTCAAACTAGCTCTTTGATGCATTTGCTATGGGAACTTGGCCACATTATTAGC
TTCTGTGAGACTCAGCTGTTTGTTTTCATTTTTAATGTAAAAAGTATAGAAAACAAT
CTATAACTTGCAGCATTCATTTAAAACCCGTATTATCTGCTGAGCACTATGCTA
>non-DHS207
GCTGAGCACTATGCTATGTTAGGGAGATAAAAAGTTAACAAAGACGAATCTTCATTA
AATAATATTATATCCCAGTGAGAGAGATGGACAAATATGTCAGTGGCTGCAAGAAAG
TATGATGTGTGCAGGTGTTAAAATTTCACACCATTCTATGTGTTTAGGAAGGGAGAG
CGAAGATCTGCCATCAGACTAGTCCATGGGGTCTCATTGTAGGAAATGAGGCTTACA
TTGTGCTTTGAAAGATGGACTGGAGTTAGTTGGATGA
>non-DHS208
GCTCTAGCACCTACAAGAGACACCCTCCCTCTCCACCGTGGATCACCTGTCTCACCC
GTGTGAGTCTCCACACAGAAACCAGTCATGGCCCTGCCTTTGCCCCCCTTGAAATCA

CCCAAGAGGATGCTTCAAACTCAGGCAAGCACAACTGATACTCACTCTGCATGCGAC
AGCAGGGGAGTGACCTTACGAGAAACTAATGGGCCTCGCTTCCTGAGAATCACTGTC
AGAGC
>non-DHS209
CTTCCTGAGAATCACTGTCAGAGCCCGTAACATAAGATAAATCACAGCCAAGGATTT
TTGAAAAGTTTCCTGATGAGAGAACCATGACCTTTTCAAGGGTAGTGATGGATTGAT
CAGGGCAAGCAATAGGTAGATTTTTTAAAACAAGCCTGGCGAAAAAAACAATGATAT
ATATCTACCAAAGTTGGCTGGAATACCCGGCTGGTGTCCCAGTCAGGAAACACAGGA
AAAGGTTACA
>non-DHS210
TCCGTAAGTCAACACGGTTTTATCGCAGGCCAACGGGAGGGATTCCGCAGTTCCCGG
CGAGCGTGTTCCCTGGATTCAGAGCACCGCGCGGAGGGCTGAGCTCCTGGTCTTCGG
TGGACCTGTGCTGACTCAGACAGCGGAGTTCCTCCTCTTCTCTCAGCAGACGGCTCC
TCTTCCATCCACCACACCCAAAGACTCCGCCGACAGCCGAGGGGAAAGAACTTCCAC
AACTAGGATTGCCTTGGATGTC
>non-DHS211
TGAATACTGAATGCGAGAGAGGACGGTGGCATTGGCATTTGTTCAGGAATGACAAAG
GCAAGAAGCACATTGCCTTATTTGGTCAAAGGCTCAGGACTGGGAAGATATGGGGAA
GTCAAGAGGTTTCCATCTGGAATGAGATTGGAAGTTGGTGCCTTAAGACCTGAGACT
TTGAAAATTATTAGGTTGGTGAAACGTAATTGTGATTTTTGCCATTAAAAGTAAATT
GCAGTTTTTGCCATTAAGAGTCACAGCAAAGAGTAAATGCC
>non-DHS212
TGTTACCTAGGGGACATTGTACTGCATACCACTGCTTTAAAGAAATTTACTGAAAAC
CAATAAAACCACCATATGTGTATGTTGACAGTTCTCACACACATTTTTTAAAGTGCC
TTTTAGAATGACAATATCATTTAATAATTATCTTTTTCACAGAAGTCCTTTTAATAT
CCCACAGGACAATGAAGAGACTGCAGGACCCAGTATGGGAACTGCAGGCTGACACGG
CGGCCGGTAGGATCCTGCTTCGTGAATTTGCCCTCTGAATACTGA
>non-DHS213
AAAACTTGCAGACAATGGAGCCTACACATATGTGTCGAATAGCTTCAGAAATAATTT
TGCTCTCTTGTCAGTGCTGAGTGACAGCTCTAATAAATTCCTACATTTTAAGCAGAC
TTACATTTCACATGCACTTGCTCTCCTCTCTCGAGCAGAGTGGAACATCTATTGTCA
AATGCATAATATAGCATTAGAAATGCAAACTTGCTGTAGCCAATGGTAATGTGT
>non-DHS214
TAGAAATGCAAACTTGCTGTAGCCAATGGTAATGTGTTATTTAAAACTTATGAACCC
AAATGAGGGCTTATTTTCTCCAAATTGAAATTTATAGTTTCATTTTAGGGTGCTAAC
TTGAATATTGATGGGGCTTAGGCATGCTGTTATAAAATGGCAATGAGAAACAGAGCA
AGTAAGTGCCAGACACAGAGGAGCGATTATAGCAATGCATGGATCGTATGTAAAAAT
TGAGTTCCCTTACCCTACTGTGGCTAA

>non-DHS215
TTGAGTTCCCTTACCCTACTGTGGCTAAAAATGTAGGGAGGCTTTTGTGCAGGTAGG
GGTGGTATGCAGCTCTCAGTCTGTTAGTATTCTATTGGCTTTCACACCATGTGAGTT
TAGTGCAAAATTAAAAAAAAGAAAAACACAAAAAGAGTAAAACAGAAATGACTGGTT
CTTCATATCTTCCAGAAGAGGAATCTTTGAGATCCCATCACCCCTCTGTTAGATGAG

CCTGTGATT
>non-DHS216
CCATCACCCCTCTGTTAGATGAGCCTGTGATTAATAGCTTTGTCTATCACCTTGGCA
AAATTGTTACAATATTATACTTTACACAATTGAAATTCAAATCAGTTCACCTAACAA
ACATTAAATGTCAGGCCCTGCTAATGGGTCAGAGATTCAAATCAAACAGAATACCTC
TTTTCAAGGAGGCTGCAACGCAGGGGGAAACACTTATTTAAATACACTCTAGATGTC
ACGGTGTAGCACATATGA
>non-DHS217
GGCTGTGGGCTTCTACAGAGTAGTTGCAGCCCTGTGCACAGGCAGGGGGGGTGTCAC
CGACTTGCATGTGGGCAGGTCTTTGCTGCGCATCAGGTGCTTTAAGCTTCGGAACAA
CTGTGCAGGATTCTATTTTAGTATTCTGGAAGCATCATTGAGGAAGTAGTCCAGTGA
AGTTAGCTCTAAAAAAACTCTTTACTCTAACAATTAAAAGAAATATGCCAAAGGATC
CATAAGGG
>non-DHS218
TTCTAGAAAGTGGACACAGCAAGTGCAAAGGCATGACATCAAGAGAAAAAAGAAAAA
AATGAAAACACTATGAATTCGGGGAACTATGCAAATGTCTGAAATGGCAGAATGCAG
GACTCCGGAAGAATAGGTAGGTAAGTCCTTTGCATTTTATCCCAAAGAGTTTGGACT
CTGTGTTATATATAGTAAGGAACCAAGTCAGGGTTTTAGTAAGAAATTGGTGGGATC
AGATTTGAGTTTGGGAGAT
>non-DHS219
TGGTGGGATCAGATTTGAGTTTGGGAGATTAATCTCTTGGTATGAATGAAATTATGA
ATGGCTGAATGAAGGTACAAAACCCCGAGTATATTATCAAAGACTTTCTTGCAATTA
TTGACTGTGTGCATAAGGCATGGTTTAGGATAAAATCTTCAGGACTTGGTTTGGATG
GAAATAAAACCATTAATTTACCCTAGTATCATAGTCTGCTCAAAATGGATGGAA
>non-DHS220
AGGAGCCTCATTTAGAGAGCAGACATGGCAAAGGCCTATTTGAGAAAGTGATTATTA
ATTAAGACCTGAAGAATGATTAGGAGACAATTAGATAAAAAGGGAAGAAGAGAAATA
ATGGGCTAAGGCAGTGAGGCAGAGAAGAGCTTCTTTTATTCCAGAAATGAAAGAAGC
CAGACAAGTTTGGGGATGAGTAAATAAGGAAGGGGATGGCAAGGGAGACACAGGGGG
TTTTGGTGGCAGCGATCAGGGAATTGTAGCAAAG
>non-DHS221
AAACCTCAAGTCTAGCAGCTCTCCAAGCCCAGCTGTCTCAGACAAAGAAGCTGACAA
ACATATGTTGAGACTCTCTATGCAGCACAGTTTGGTGAGAGCTATTTGATAATGGTG
CATTGGGATTGTACAGGAAGAGAGGGCAGGAAGAACAACAGAAATAGTCTTGACCTT
GTTGTCCACCTTAAGTATTGAGAATGGCACTCATCAAAATTCAGAGAGAATGAAAAG
CAAACAGAAAGTGTTCCTTGGTT
>non-DHS222
AAGCAAACAGAAAGTGTTCCTTGGTTTTCAAGTTCTTATACAGTTTTTCTAAGAGTC
TTTCAAGGGCTTGAGTGAAATCAAGTAGTTTCATTTTACTACAAGTTCTTGCAAGAC
AATCTCCAAATTCCAGTGGCCTGGTTTTACCCACTCTCTCCTCTCCAAGTTTGAATG
CTCCTGTACAGACTTACTGCAATTTCACTTGAAGTCATTTCTGTCTCATCCAGTTTC
ATCTCCCAG
>non-DHS223
CTCCCTTTCTGAGCTGGGATTAGGAGTTGATAATGGTCTTGCAAGTGCAGTTCTGCA

GAGCGAAAGGGCCCGGTGCCTTGCAGCAGAGACACGTTCTGGGAGAGAGGGTTAGGA
TTTGAGTAAGTTCACACTAGGAGGCTATATATACATGTGTGGCATTGCCCAGGGACT
GGAGGGAAAGAGGAGAAGGAAATGCATCACAACAATGACTGGAATGCATCAGCAG
>non-DHS224
GCATGTGGAAATCCAATGACTCCCAGCTAGTATAGTTTGGGAAATATTTCACTCAGG
AGCAGCTCAAGCATGTATGCACTGGTGCAAATACACCTACAGCCTAGGTGTAGGGGA
GCACACCTTTATGTTAGCAAACTGAAACCCATCCTCTGTGGACAATTCTATGCAAAA
ACAAAAGTGCAGTAGCACCAAATGAGCATGCTGGCCTCTCGGTGCAGGCTGGCTGGC
AGCCAAGTCCAGCCTGCTGAGAGAGAAAATTGAAGGA
>non-DHS225
CCTGCTGAGAGAGAAAATTGAAGGAAGAAATAAAGCAACTGCACAAGATGAAGTCTG
AAGGTAGATGTGGAGACTCTTCTGGGAAATCTGTGGAAAAGGTCAAAATTTTGCTCG
TGGGCACTTTTAGAGCTGGACCCACTGCTGGCGGTCCTAGTTTCATGAAAGTTCAGG
TCCTTTTTGTGGGGAGAAATACACACACATGCAAGCTAACTCAGAGTGTGAGCACTG
AAGTGTGCTGTTAGGAACAGG
>non-DHS226
GTGAGCACTGAAGTGTGCTGTTAGGAACAGGTATTCGCCAGCAGCCACTGTTTTGTT
TTTAGTATCTGTCATTCGCAGTCGATCTGGATTTCAAAAATGATTCACTGACCACCA
GGGTCTGTGACTCCTTAAAATTAATGCAGTCTGTAGATGTGCTTCATTCTGAGAAGG
TACAGAAGAGAAGTGACATAATTGCAGAAAGATGTTCTGAATAAGAGTGCAAAAGAT
ATACTTTTCTGGCCACAGGCT
>non-DHS227
TCTCACTGCTTCATTGTTTGTCACAGGGCTCCTTGCTAAAGAACACAGAAGTCCATT
TTCCTCTCAAGGCTCTTCTCAACTCACAAACTCATGATGACTTGTCTCTTTGACAAT
TGCTACAGTTCGTTTAATGGATGATTACTGAGTTCTTAATGTGTGCTGTATAACTTT
TTAGTATCTTTTTTCTTAACATATGCTTTTATAGTTGTCATTTTCCTGATTATATCC
CATCTAGGGCTATGA
>non-DHS228
GAGCTGGAAGCCCTATAAACACTGAATCTGGGGTTAGTTTCTTACCTTTGTAATTTT
CTTTCTTTATGAAAGAATCCATCAGTAAATTGAATGTAAAGTTATCTGGAAAAATTC
CATATTGAACCTATAATGAAAATGAACATAACAGGAAAAAAAGAGAAAATTATCTAA
TTTAGTCTGTAATATATGCCTATGTATTATTCAGAATAAAACTTGCCACCATTAGC
>non-DHS229
TGGTGTACTCCACAACTACAGTGCAGGTGGAGAGGCAGATGGCCTCAATCAGGCTAC
CAAAGTTTCCTCAGAAACCCCTCAGCAGCCAGTAGCCTTGGTTAGTCAAGCCATCAT
CCAGGATAATGAGCTTACAGGAAAATCACTGGTTATGGGAGGCGAATCAGCCTCTGA
GACAAAAGAGGAAGTGACTTCCAGTAAAAACAAGTTAAGACATCAAATAGGAGAAGG
ACTTCCG
>non-DHS230
CTGAAGAAATAACGGAGCCATACCCCTAAGTCCCCATCAATTCCTGGAGTTGCTCTT
GGAGCAGCTGGCTCCATTGTTTGGGCTTAGATGAGAGTCATGGGTATAGTTTTCTGA
CATAGTACAGCAGCCCCACAGCAATGGTATGCTAAATGAAGTCAGTCTCTTAAGAAT
CTAGTTTGCACATCTCTTCTCAACTCCCCATTCAGTGACTGTTATGTTGCTAGCTTG
AAAATTGGCTAGGGTGGGAGATTTTACATTG

>non-DHS231
GCTAGGGTGGGAGATTTTACATTGCATGGAAATCAGGAAATGCTGCAAACTAGGACT
TCCCCCACTCCACCCAGAGCTGGCTGTCAAACATTTACCAACATATTACTGTTCCAG
AGTAAGCTAAGGTCAGAGGAAAAGGACAATACCCTGGAGGGTCAGGAAACTATTCTA
AGAAGAGACGTTACCTACAGAGTCTGCCTATCCCTACCAAATCCCATGAGGCAGATT
CTTGTAATCA
>non-DHS232
ACCCCTAAGGGTAGAAGTGTTTGGATTTTCACTCCAGCATATATTCCTTAGGAAGAT
TTTCCTTTGAACTTGGAAACTCTGGCTCTCAGAATGCTGCCTTCTCAAAGCAGGCTA
ATTATAGTGATTCAAATGGCTCTGCAAGACAGAATGTAGACAGCATACAATTAGAGG
CTTGCTAAATGCCTGCAAAATGCAGTTCTTCCTAATTTCATTACTGTCTCTTTTGAT
TACAGGCTGATTACTTTCAGCC
>non-DHS233
GCTTGTATAACACATTTCTGCCAAGAACAAATGATTATGGTGGAATTATAAAGCTCT
GAAAATGGAAGTGATGGCACAAACTTGAATATCATAGTGTGAATGTGGAACTATAAG
AGCAGTTTTATCTTTATTATATTAAAAATCTACAGCTGTTTAATGATAACAAAAGAA
TTTCTGTTAGATTCAGCCAAGTCTGTCTTTATCACAGTAATGAATAGTCGATATGAA
CGAAAGGGCTGACTA
>non-DHS234
ATAGTTGCATGCTTGGGATTTGGGTTTTGTTGAGTTCAAATGTGAAGTTTATTTTCC
ATTAAGTATTTATCAATTTACACATTTTACTTGCAGGAGAAATTTTACAATTCCATG
TGATAAGAACTTTCCCTGGTCGAGGAAATGTTACTGTTAACTGGAAAATTATTGGGC
AAAATCTAGAACTCAATTTTGCTAACTTTAGCGGACAACTTTTCTTTCCTGAGGTAA
>non-DHS235
GCCGTTGTTTGGTTCTTGAAAAAAAAAAAGAATTTCTGTTCTGTAACTCTATATTAA
ACTATAGCATGAGTATTAATAAAATAATTCTAAAAGATGAAAATAATTGAAAAAGTA
ACTTAGCAGTAGAAGTAATTTCTACTACTTTTAAAAAGAGTTTGTTTTATAATTCAC
AGCCAAGATAGTGAAATACAATTCTTAGGTTAAAACAATAATTGCTTTATAATGTAA
TCTAAAGTTATCCATTTACTCTTTCCCATCCCT
>non-DHS236
GGGTTATCAGGAATGGAATTATGCCAGGGAGCTGTCATTCAAAAACCTCTTCTGATC
CACATTTCCCAGGTGTCCATCTATGCATCCCCAGACAAACGGAATCAAACACATTAT
GCTTTGCAGGCATCACTGAAGCTACTTGATTTTTATGAAAAGTACTTTGATATCTAC
TATCCACTCTCCAAACTGGGTATGTTCAAATTCCACATTATTGTCTTCATTTTTGCT
CATAAAACTTGCTTTGATCTCTTCCCTCTTT
>non-DHS237
TTGCTTTGATCTCTTCCCTCTTTCTCTTTCTATGTGATTTAAATGAGCACTGAGGAA
TTCAGTTAGCTCAGGAAAAAATAATTTGTTCCTCAGAGATGATTCTTGAGTGTAGAA
AATAAAATATTTATGACATGCCCCAACAGTGTGGATCATTTCTCTATTCTTTTATCA
GGTTTAACGTTAACATATCTGCATCGAACTCTTTCCCAGGCTGATCAGAAAGGGCAC
ACACTGGAG
>non-DHS238
CAGAAAGGGCACACACTGGAGGCTGGGACGGAAGCCAGGTAGAGGGTCCAGGAAAGA
GATGGGGAGAAAAAGAAGGAACACAGTGACTGCTCTGTTCAAAATAGGGGTCCACAT

GTCCAAGATGCTGTGGCTCCCTGTGGCGGACATCAACGCTCTCATCCATTATGCTCC
TCTTCTGTGGGAGGGAAACACACCTCCCATCGTGCTGCTCTTCTATGCCCAGCAGCA
TTGATTAGAGAATGGA
>non-DHS239
CAGCAGCATTGATTAGAGAATGGATTTTCCATTAAAAAATACATACACACACACACA
CACACACACACACACACACGCATTGCATATTAGAATTAGAGGGATTTCTGGAGGAAT
CACCATACCTTATTTGTACAAGGTCAGCAATCTTTTATAAAAGTTGTCAAAAGTTTA
TGTAGAGAGAGAACTGAAAACTATGCTTCCATCCGTTATCTGTGTTGGGCACTGAGG
TTGGATGGTAAGACTGTG
>non-DHS240
TAGACTGCCACATGCTCCATTTACATAAAACCCATTAGTTTGCATTAAATACAAATT
AGCTGCTGATATGAATTAAATCACGTATAAATAGTTGAGAATAGCCGCAGAACCAGG
CATTTTAATTAATATAACTGTTAACCAGATTTATTTGTAAGGTAATCAAGACTCTTA
CTCATTTATTCTTTAGTACAATAGGCCAGGCAACACTGGCTGTCCCAGAACAGGCCA
TCCTTTCTAC
>non-DHS241
GACCAAATGATTGACAGAAACCAGTGAACATTTTCATAGTGATCAAAGCACCCCAAA
GCTGGTCCTTCTAGACTGATACCAGTTGATCTGTTGTTTCGTTTTGGAGATGGGCAG
CCTCACTGCTGGCCTCTCAGTGGTTGCTTTTAGGATACAGCTGTCTGCCCTCCGAAC
TGGTGGCTGGAGTATCCTCCAGCGAACAGAAGGCTGTGGAATTTGTATCATGCTCT
>non-DHS242
ATTACCAGGATGGAGGGCACTTCCCAGCACGAGCCGACACCCTAGAGGGCACCGGCG
AGCTCCCGAAAAAACTAGGGCGGTGGGGCGAGGAAGATCCGCGCCTCGGATTCCAGA
GAAGGAAAGAAAGCTAGAGGGAGCCGGAGCGGAGCTGCACTTCACGCCGAAGTTTCC
CCTTCGAATCGTCTGGAGTTGTAAGTGGTTTCTGATTGAACACAGCTGTCTAGCTGC
T
>non-DHS243
CCTGGCATGATTTATTTGGTTCTCCGTTTGTCTTCTTTAATATGCAGGGCCACCAGC
AGGTTAAACAGTTGTATATTTTCCATTTAAAATTTCCATAATCTTTTTTGTGTGTGT
GTGAAAACCTAAAAATTGTGGTAACTTACCTGGAGACCTTGTGGAAACCATTTTGCT
TTGATTTTTCCAATCACTCACAGTAAATCAGCTACTGAATATATTTCCCCGTTAGG
>non-DHS244
GGTCTCATTTCTCTCTCCCTTGAATGAAAAGTTGACCTGCACAATTCAATCTCCATT
GAGATGAATTAGAAATTAGAGGCGTTCTATGCAGGTTTAAGCAGCTCCGGGATTTGT
CATTACATTTTTACAAATTTCCTTAGTGGGAGCTAACAATAAAGATTAAAAGCAATA
AACCTGCAAAGTACATATTGGAGCTAGATTTGAAAGGACACAACAAGCTCTTGCTCA
CAAAGAAAAGTCCC
>non-DHS245
GTCCAGAGAGCTTGCTGGTCTTTGCAGCGTGCACACACAGCGCGCGAGGAAGGCGCC
ACATACTGAGCCCGGGACTCGGGATGAGCCCAAGGCCCTGAGAGCTGCTGAAATAGA
GCCCCGCCCCCCGCCCGGGAGAACCCATTGGGCAGACCCGACCCCTCCCACTGGGCA
CGACCCCGCCACCGCCCCGTCTGCCCCTGGTCTTCCGGAGGAGCCTGCGGCTGAATC
CTGCCTGGAACAATGGGAGTGCTACA
>non-DHS246

AAGGCAATACACGATCAATATAGGCAGTGAAACAAAAGTATCATTTGCAAGTTAAAA
CAGACTTCCCAATTTTAAATCTGGTTTCCCCCTGAATATGTGGCATCCTTGGCAGCA
CTTCTGAGAGTGGCTGCTTTCATTCCAAGAAGCCCATGGGTTTGGAGGTGGGATAGG
TGCCTTTCTGGCTTCTCATTGCTGCTTCTAGATCAGTCTCCAAATATCCCCCTTCCC
CACATTGGAATGA
>non-DHS247
GCTATTTGTAGGGAGGCTGTGTAGCATCATGGTCAAGAGTGCAGGCACACTGCCTGG
ATCCCAATTCTGCTTCTCACATTTGTTAATGGGAAGCCTCTTTATAAGCCTCAGTTT
CCTTATCTGTAAAGTGGGGAAACCAAAATTACCTCCCTCATCAGGTGGTCTTTAGAG
CACTCACTGATTTGCTGCATTTAGCAGAATGTCTGGGCTACAGTGAGTGTTTGACAA
ATG
>non-DHS248
AGAATGTCTGGGCTACAGTGAGTGTTTGACAAATGTCTGAGATCATTTTCATCATTC
TTATTATTCTGTACATTATACCATGAGCTCCCCAATGGAAGAAACAGCTTAAGTTCA
TTGTTCAAATCTTCCACACTGTTTTTCATAACCCCTCACACTCTATGGGTGTAATAA
ACATTTGTCCAATGGAATTCTTATACTGTTATACTCCACCTTAATATTTAAGTAGCC
TCAAGTCTTTTATGGAGCA
>non-DHS249
CCTCAAGTCTTTTATGGAGCAAATGTTTTGGAAACTCAAAATAATGAAAAAGTGTAT
CCAAAGGAATAAGATTAGGTTCACAGATATAACCTTAGTATTTTTATTCAAATACAT
AAAAGTTACAAGGCAATATATCCATTTATTTGAATAGCTCAACCTAAATAAAATTTG
GAAAAGTATGTATCCATCAGTGCTATTGAGGAATAGTGGTATGAGTAATAAATGAGG
AATAAATCAGTCTGGGGTATG
>non-DHS250
ATCAGTCTGGGGTATGAGAGAAGGACCATCTGGCAGAGGCAATGGGAAGCTTCATTA
AAGCTGTTAAGCCCCAGATACTAGGTCAAAAACAGAGGGATTTGTTTTGAGCTCTCT
AAAACTGCAAGTATGCATAATTGCACTGTGTAATCCAGCAAACCTTTGCCCCTTCCG
AGGTGCTCTGCTGCTAATCCTGCCACTGTTGCAGATGTTTCCCTTCATCTCAAAGCT
TCTCCTGTGAGTTAAAGTGAGCCACAGTGG
>non-DHS251
CAGAGGCTAAATAGCTTGTCAAAGGTTACATAGCTAACAAGGTAGGTCTTATTACTC
CCATTTTATAAGAAAACTGGAACCTATGAAAATTGCCTGTAAAAGCAAGTAACTGGG
GCAGGCTTTGAACCAGCATCTGTTGGACTCTCAAGTCCATATGCTTTTATTTCTGTT
ACTCTGCCACCTTATGTGGATTATCAATAGCAAATGGTTAAGGTTGGCTTCCCTCAT
TCTCACCTTCCTTT
>non-DHS252
GTTAAGGTTGGCTTCCCTCATTCTCACCTTCCTTTGCCATAAGCCAGAGTGTACTGA
GAATAGTCTCGTTTGAACATTTGCCTCTGAGGAGAATATAAACAGGAAGGCAAGTCT
GATGCAAAACTCCATATTCTGAAATGCATCCCTTAAAACAATGCACCAAGGATCCTC
TCTCCCGAGGTGTCTGTCGCACTCCATCTAGCATACATATCTGAGAGTTTCTAAGGC
TTGCCTTTCAGATACTTGGTGGTTCCT

>non-DHS253
TAAGGCTTGCCTTTCAGATACTTGGTGGTTCCTAATAAGGTGCCCTAATCAGTTTCT

TAATCAAGGCAACACTTCAAAAGATTTTCAAATGAATGCTACAGACAGGTTTCCTAT
TTCCCTCTGTCTCAGAAGGCCCTCAAAATCCCCTCCACTGAAAGAAACAAAGCCGCT
TCAAGCCTCCCAAAACATCTTGAAAATACTCACATTTTCAAAAGGACTGCACTCATA
GGAAG
>non-DHS254
GAAAACCCTAGGCCATGTTAATTGGTTATACATGTTTGGAATGTTAACCAACGTATT
TGTCAGTTGTGGTTTTTATTCGCTCTTAAACTTTGTGCATGCTTTAACAATTTATTA
CTTTTAAATCTAGAGTGAATTCTAAAGACTGCCGCTAAAGATCTGAGTTTTAAAAAT
GTTGTTGCTGGTGGATTTCTTGTTCCTGTTACATAACTAAAAGTGAGGCCATTTGTG
GTT
>non-DHS255
GCTCACAATTGCCAGACTAATGACAAGAGATGGGTTTCTTGTATATACTTGGCATGC
AAATAATTGAGAAAACAAAACTGAATTACAATATTAATACAGGACTCTAAAAGAGAA
GCAATGAATGTACACCATGCAACACTAAGCAACCACTTCAATGACAAATATGTTGGC
TATGTCAATGCACAAAGATTGTAAATAAAACACAGCAATTGGATTAGGCAGAGATCA
CACCAGAAACAGCTATGCAAGT
>non-DHS256
AGATCACACCAGAAACAGCTATGCAAGTCTCGCAACAAAAATCCAAAAGACTGTGGA
GGGCAGTAAGAGTTGTCCTGTTTGGCATCCCATAAGTTTTGTTCCTGTAAACTTGCT
TAGGTTCATAACAAAAATGAATGGTCCAGAAAATAAGTGCAATAGAAATGTGGAGGA
TGGAATGGTCATGGAGGAATGAGACCACAGGGAACATCTCAAACAGGGAGAATAGCA
TATGCAAGCATTCAGAGGTAAAGACGT

>non-DHS257
GCAAGCATTCAGAGGTAAAGACGTATAGACTATATCAAGACATAGTGAGTAAACCAG
CTTAGTTTGGAAATACAGCTTTAGATCCTGATGTGAAGGCATATAGGCCAATCTAAA
CTGTCTGGATTCAATTTTATAGGTTAAGGAGAGTTTCTGAGGGTTTTGGAGAGCAGT
TCTCATTAATCTTCCTGTGAGAATTAAATGAGTTAAAAGTAAAAGTAAAGTTCTTAG
AACAGAGATAAGTGTTTATTATTACTTTCACCTTTGCAGTATGTCGC
>non-DHS258
TTACTTTCACCTTTGCAGTATGTCGCCCAAATAAGCCATTAGTTTCTTGAGAGTAGG
GATTACATCTTCCAGAGAAGAAAGGAAGTACTGGGGATCTGGTAACCCAGACTCATA
GGGTCAGAGAAGACTTCCCAGTATAAACCAGAGAAGGCTTCCCAGTATAAATAATAA
ACCTTGTTTATTATTACTTTCACCTCTGTGTATTGTCTCCCAAATAAGCCATTAGTT
TCTTGAGAGTAGGGATTACACCTTCCAGA
>non-DHS259
ACATTTACATGACATTGGCACCTGGTGAGGTATTTCTTACCAGTTGGTCCCTAGATA
TTGACTTTTATCTCTCCAGGAATTTTTGCAGTGTAATAAAACTCCTATGGTCACTGT
ATCATAGAGGGCCTGGTTCTCCATTACCCAGCTCCCTGTTATCTGTTTCATGTGTTA
TCATACTCTTCTGAGTTATATGATTCCCCCCATCAAGCTATCAATTGTAATGTCTCT
TAACTCAAAGTCCCTTGTTC
>non-DHS260
TCTTAACTCAAAGTCCCTTGTTCTGAAATAAAAACCCTTCTTGTTCTTGAGCCCTCC
CAGCACTTGGCTATCTTGGGGACTATGGATTTCAGCTGCTTTTTCTGATCTTCATTG

CAACATGACAGACTCCTGTAAGACTGCTATAAAGTCCAAATACTTGGATATCTTCCT
CGATACTGCCCACTCTGTTTTACCCATGATATGCTGACTAATGCTTTAGATGCCTCC
AACAGCCAAGGAATTTGAACTAAACCAGG
>non-DHS261
GTGTTTCCCATTGATAGCTGGAGACAGCCCAGTAGCTGTGAGTCGGTCTGACAAAGC
CATATTGAAGTACGGAGTACGGTTTCAAAGCAGTCAGAAAAAGAACGGGAATGCTGT
TCAGGAAATTCTTCAGGCATGGGCAGGGACTTGGCTGCAGTTCTGCAGTTGGAAAAT
CTGACTGGGGCAGCTTCTGAGCGCAGGCTGGGCCTGCACACACTCAGCGGGCCGAGT
GGCCACCTCCTTCAGAGCTGCTCAG
>non-DHS262
AAAACGCTGAGGGTCAGGAACAGGTGTGGGAGGGGGTTCGTTCTGATCTTATCCCAT
TTCTCAGTTCCTCAGAATGTTAGTGCTGAAAGGGACTGGTGTGAGAAGGCATCTGGT
CCAACCCCCTCATGTTACATGAGACAACTGAGGCCCAGAGTGGGGGCTTTGTTGCCT
AAGGTCACACAGCAAATCAGCAGCAGTGCCTGGCCTTGAACCCCGGTCTTTTGACTC
CCAGTCCAGTG
>non-DHS263
GGTCTTTTGACTCCCAGTCCAGTGCTCCACCCATGGCATCTGTGGCCCTCTGGGAAA
GCAGGGAGTGACTCCAGGGTGACACGTAATGAGGTTCTCCTCTGATATACACGCCCT
TGGCCAAGCAGAGCAGGGAGCTACCTGGTGTGGCCAGGTCCACTGGCCCAGGGATGC
CACGGGGCCCGGGTTGGCCATGAAGGTTTTCACAGTTCCACATTTATTTCTCAAACC
TTAAACTGGTGCTTCCTTAGGA
>non-DHS264
GAGCATCTACTATGTGGGAAAGACCCTCTTTTTGTTCCAGTCATCGGCAGCTGGGGG
CTCTAGGTCCATCCCTGCCCTCCCCGAAGCAGGAGAGTAACCTTGTAGACTTCCTGT
TAGGGCCCTGGGGTCTCTGGGTGAATTTGATGCAGACTCAGCCATTAGCCTGGCTGG
GCCACAAAATCAGGAAGCTTAGACCAGTGTTGTGGAACCTTGATTCCTGTCCCAGTT
TTGTCATT
>non-DHS265
AAGACTTTTGATGCCCTGTTTCTGGAAAGAGACTAGAAGGTATAGTCTCCTACCTGG
GATGTCAGAGTGAGTCTCACCAAAGTCAGACCTCAAGTCCAGTTTGAGCCGGACTTA
TCAAGGCAAACAGGGATTTAATCAATGCAAGCTTATGACCTGCACTTACTAGGAATT
CCTCGGTCATGGGGAATAATTGCAATCCCCAATCCCCATCACGAATAGGGTTCAAGA
GTTTACCAGCG
>non-DHS266
TTCAATCATCTTACAGGAGTGGATGACATACCTTAAAAGATTAAGCTTCCCCACCTG
GGATTTGTCCAGAAATTCAGTGTATTACAGCTTCTCAGAGCCTTCTTCGTTCTCCTA
TATTCTTGTGTTATCAATAGTAGCTTTCTTTCTTATTTTATGTTTAAGTATGAGACA
CAGGCAGCTATGCTGATGGAGTCAGTTTGTACTGTCACTCATCTTTCACTACAACTG
CAAGGTTCACTTCCTTT
>non-DHS267
GTCTGTCTATTTTGTGGCTTCCAAAATTCTAACTGCAAAATAAATGACCATCTTATC
ATGTTTTCCCATGGCTATCAGGAGGATATAGCAATTTTATAGATTACTACATGGTCA
AGAATCTACGCATCGGATGTATTCTTTTCTTCTTCTTTACTTGATGCATTCAAAAGC
AACCACTGGATTTTTTTCAATGTAAAAAGAAAAATAGATACCCATGCCAAAATTCA

TGGGTGTAAACAGCCAAC
>non-DHS268
CCTCTCTCCCTCGACATCCTCCCCCCCATGTGGGAGGGGAAACTTTTCTCCTACATA
CTTTCGAGTCCTTCTCGGGCCGGGAAGCGCGGAGATGACGGCGCCCGCCCTCCCGCC
GCCGCCCTAAGGTATCCGGGGGTGCCTGCCCGGGGTGCTCGCGCGGGGGTGCCTGCT
CGGGGCTGCTCGGTGGGAGCATCCGCCGCGCCCTGCTCTGCGGACTTTGAACCT
>non-DHS269
TGGTGCCTAGAATCCTGAAATGTAACATTGCCACCATAACCAAAAAAAAAGCTGCTA
CCAAGAGTGAATAGCATTTAATTCAGTAAACTTTATTTCTACTCACTAAACACAATA
CAGATTACAAATCCATGCAAATCACTTGACACTTTTCTAACAATTTCTCCTCTGAAC
AAGTATGCTAATATTCCTCCCATCTTCAAGATACTAATTTTAACCAAAAGCTAAAAT
TTTGTTAACCTAATTCACAAAGTTCTAAGACCCTTAAACAGC
>non-DHS270
AGCTCTTCTACAGGAAGTGGCATTTTCCACTTGTGAAACGGTAGGTCATTCCCTGCC
TCATGCAGAACTCAGCCCTGTGGAGCCTCCACCACCTGGCCCAGGCCCTGCCCACAT
GCAACCTCCCGGGGTGGCCCTCAATGACCTGCACGTCCCTTCACTCTAAGGAACCCT
GAGTTACAGTGGCCTTAAGGACATGTGTATTTAGAAGCCTTTGTGTACAAACTAGCT
CTGTGCGCTCTCAGTTTAC
>non-DHS271
CTGGAATGGAATAGGGCTGTGTGGGCAGAAAAGAAATAGAACACGAGACAGGGAAAG
GCAGTGCAAGTGCAGAGGGGCATATGGGGTCCCCATGGCTCCGAATGCTAACCTCTG
CCCTCTTTGCAGGTGGATGGGCTCGCGCTTCTGGCCGCTGTGCTCCGACACTGGCTG
GCACGTGGACCCACATGCCCCCACATCTTTGTGGCCACCAACTTTCTGAGCCTTGTT
CAGCTACA
>non-DHS272
CAGGAAGGAGGTGATTGATGATACACTGTCTTTTATTCTCTTTTAAGACCATGGAGA
CCTGTGAGGATGGCAACGATCTTGTCTTCTTCTATCAGGTTTGCGAAGGTGTTGCGA
AGGCCAGCCATGCCTCCCACACAGCTGCCCAGGCTGGGCTTCCTGACAAGCTTGTGG
CTCGTGGCAAGGAGGTGATGAGATCCAAATGTGCAACCACCTCCACATCAGAGCTCC
CTTTCATTCCTAGTC
>non-DHS273
CAGAGCTCCCTTTCATTCCTAGTCCTACTGGGCCTGGGTCTAGGTCCACAGGATTTC
TGACCCTTATTTCCCCTTCTCTTCCCCACTCCCCTTACTCCTCCCACCTTCTTGCTT
GTTCCTAGGTCTCAGACTTGATCCGCAGTGGAAAACCCATCAAGCCTGTCAAGGATT
TGCTAAAGAAGAACCAAATGGAAAAGTGCGTATATGGCCCCAGTGTCTTTACCCTCT
CTGCATCTTCTCC
>non-DHS274
TGTGAAACTGGATCTTCATAGTGGCCCCCCACCTCCCTGCCCCGCACTGGGCGGCCA
TCACACCAGCCACACCTGTCCAGCCTGCTTCCCATCCTATTCTGGCCCTTGGACCCA
CATTCCCTCTAGCCAAGTATGCTTTCTCCCCACCCCAACACAAAAATCGCAGTTTAT
TACCAAACCCAACATTTATTGAGAACAAAAGGAACCAGTTGGCATAGAGGCCCGACT
TCAATTCAT
>non-DHS275
CTTCTTGTGTCCAAGCCCTCAGACCCTTCCCCACCTCCCCCTCCTTTCCCTTTCCCG

AAGGATTGTTCCCTTTCTCTGCCTGGTCTCCCAGGGCAACCCCCGCCGCCGGGTGTG
AGAGGAAAGAGTATGTGTCACTGTGTATGCGTGACACTCCGGGTCTTTTTGAAGGGA
GGGGTTCGTGCGTCACCCCTTTCCACTGGTTCTGCAGCACCAGTCCCCTCCCCCCAA
CTCCCTGGGTTCTTATGGTC
>non-DHS276
TGCTTCAGGCTTCTATGCTAACTCTTGGAGGCCCAGAGGCATTTGACTTTATGTATT
TGAGTTTTCAGTATCTACTAAAATCATTCTTTGGGGAATTTTTTTAAAAGCTGCAAC
TGTACAGAAAACCTACATCTTTACATCTGTACTTATGGTATTGGGGTGGGAAAAAAT
GGACAATAAGGGTGAAAAGAAACAGTGCTATGAAACACAATGAAACCAAGTAAAGCA
TCTGCCTT
>non-DHS277
TAAAGCATCTGCCTTTACTTGACCTTAATACAGAAGCAAAGCTTTTGGGAAACAGTA
ACATTGCTTGCTGCCCCACAGCCATGGCTAGGACCCAGTCACTACAAGACATTAATA
CAGTAGAAGGCAGCCTTCATCTGCCTGTCTCCCCTGGCTCCCATTAAGCTCCCTACC
TACTGCAGGGAGCCAGAGAAAAGGAGAGGCACTACAATTACCACTTGTCACTCACCC
T
>non-DHS278
GCCCATAACATTTCCTGTTTGTCAAGGGGAAAGCTACCATGAGCACACCTTGCCTTT
CTCTTGAGGCTGAAACAGTAGCAAGAAACCTCATGGTTTGAAAGTGGAGTTGGCTGA
ATATAGATTAAGGGAGGGAAGAGTCTGAGGTGCAATTTATACAAAAAGGTTCTCTTT
CCCCTATTCCATAAATATTTAATAAGGTGGGGGGTGCTAGTTGCTATCCTGTGCCCT
AAGAAA
>non-DHS279
CCCTCCTAATTGCTGGAATACATATTAGAGAAATTCTGTAAAGAAAATTTTTTTGCT
CATCACCACTCTCACATGACTGTTTGCATTTACCCGCTGTACCCAGCCTACCCTTGT
GCAAACAGATTTTTACATGGCTGCAACCACAGAGCAAACAGTTGATTACTCCTTAAT
TCCCTTACACATCCCTACATTGATTTTCCTCCCTTCCATTCCACCGCCTGTTCTCTA
AGGGCTGGATATCG
>non-DHS280
TCAAAGCTTGTTTGTGGAGGTATCTGAGGCTCATGTCACACTCTGGGGGTAACTCGA
GTTATGGTCTAAGTGGGGGAATCTTCTGGGTGTTCCTGGGGCTGTCAGCCTTTGGGT
TCTTTGAGGGTCTCATTGGGTTGTCTTTGGGGGTCCTGGACAGTTCTCCCCTGAACA
TTCTAGCAGCCTTAGGTGGTCTTGAGCACTCTCTCCATGGGTCTTTATGGGGCACAA
GTTCTTG
>non-DHS281
TGATGGAGCTGTTACTGCTTAGTTGTAGCATTTGATATCTGACTGCTATTCAGATTT
AACACAAGCAGGATTTGGGGACCAAATTATCTCAGCAGTCAGGCAGTCAGTCTTTCT
AGATGGGGGAGGCGACAGAACAGTAGTTTAAGAAATCTGGATGGAGGTCTATATGTG
CCTACAGTCAGCTTGTCAGCATCAGGGATATGTTTGGCAAAGTGCAGCGTGTACATG
TATG
>non-DHS282
GGCCAAAGATTCCTTCACAACTGCAGTTTGAAGGAATAAAAGAAATTACCTTCATTT
TATAGCATTAAAAATGAAATGAGACTGCTACTGTATTTTGAGAACAAAATAAATGTA
CTGTCAAATACTCTTTTATCTTATTTTAAAACATCAGAATTGTGCATATTTTTAGAA

AACACAAAATGTTGAGCATCTATGAACATCAGAAGAAAATGATGATATAGATTATGT
TGAATGTTAGTTTTAAACTATAGATGG

>non-DHS283
AACCCATCTTCCATTACTGATGCTACAAGCATGAAAAATAATTCGCTTTCTTAACTA
CTTATTCTCTTACTCTACATGTTAAACTGAAAAAGGTTGTTCCTCTTTTTTTGTAAT
ATTTAGGCATTCTACCTGTTCCTCTTTGACATTTGGATTTTTAAAAGAAATGTAAAA
TTTAGGTATTCTTCTAACATCAGATGAACTGCATTAGAATGTCTTACATAGTTTCAC
AATCCCAATAATCTATTTCATTTGTGTACTTGAGATGAAGGTGGGT
>non-DHS284
CTTGAGGCTCCTAGAGAACGAACAAGGCCATGTTTGAATGTCAGGGAAACAGACGCA
GAAGGTTTCAGAGGACCTTTGTCACTGAAATTCCCTTTCCTTCTGGAAAAGCAAACT
GCTAGTTTGTTGACCATCAGGTACAAAATATATTTTTTTTCTATAAACCAACATTGT
ATCTGCTGAGATAGTTTTAATTCATGTGATTTGTGACTAAACTATTTGATTTGTGGG
TCTGATGG
>non-DHS285
CTGGTATAGGAGAAGGCTGGTTTGGAGGCTGAGATCTGATCCAGGGAGAGCTGCAGC
CCTTTGTATTCTGTGTCTCTGCAGGGTGGGAGGACAAAGCCCTTAATCAGCCACCAT
AGGAAATGGATTCATGCATACTCTCATCAGGAATGATAGTAATCCGAATTTGAAAAG
AAGGCAAGCAAAAGTTAGCCCTATAACTAGCTTCCACAAGCACCTGGATTATTCTAG
CAA
>non-DHS286
ACATAACAAAAGACACGCTCCTGATCGGGTTTTAGAAAACCCACAGACCTGGGCACA
AACCCCCGTTCCACGACCTACTAAACATACGCTATCTGAGCACAGGACATAGTTAAG
AAAATTTTAAAAACAATTGAAAAAAGAATAAACATACAATGTCAGGAGAGTTTACTC
ATCTCCGTCTCTGCATCTACCTCCTAGGTTTCACTACCTACCTTCCAGTTTAGCCTT
AGGATACGTACGCAA
>non-DHS287
TGATCTCCTCCCACCTGAACTAATTTGCCTTAACCTTTAAACATAACTATGTCTTTA
AAAAGAAGATCTGGTATTGTATGTGCCTCAAACACTGAAGATGATTAACAAATGTCA
TGAAAATAACATTCTAATCTTTTAATTTAAACTGAAAAAATTCACAGTCTGAAAAGA
ATGACATGACTTGTCTTTAGACAAGTCAATCCAGAAGCACAATACAGGAAGAAGTAC
ATGTGAAATATGCTGACCAGGTGATT
>non-DHS288
GAAAGGACCCTCCTCCCTCTTAGGCGGCAGCTCGCCTTCATCGGGTGGATTTCCTCC
TCCTTGCCTGGCCTCGTTTCTCTTTCGATCGCCTCCGCCGCGTGGTCCCGGCGCCAG
CCTCGTCGTTCCTTGCAGACGCTCTCCCTCTCCACCACCAGCTTCTGGCCACCCACT
CGTAGCGGCACCGAGGGTCAGGGCCAGCCCCCAAGGAGGGCGCTGCTAGGAAGCTCG
AGCCC
>non-DHS289
AGGCAGAGTAACCCTTTGGATATGGGAAATGAAAATAAATCAAACTCAAAACAAGTT
TATTATTTGCTTGAAGCTCTGGTAAAAGATAAAGTGAAACCTGCAAACTGTAAAATG
AAGTTGCTGCTACACATTTCTCCAGTTTTGGATTTCATTCTTGGATGGCCTTTAACT
TGGATAGAAGGATACAAGACAGCCATATATATATATATATACAGCTGTTGCTCTTTA

AGATATCCACTTC
>non-DHS290
AGATATCCACTTCACCCTGTTGTGGGGTGGGAGGAGGGGGGAGGGATAGCATTAGGA
GATATACCTAATGTAAATGTCGAGTTAATGGGTGCAGCACACCAACATGGCACATGT
ATACATATGTAACAAACCTGCACCTTGTGCGCATGTACCCTAGAACTTAAAGTACAA
TAAAAAAAAAAATTAAAGAAAAGAGATCCACTTCGCTCAGCACATGTGATTTGGCTT
TGGTCTTTGGTGTTAATTTGGATGC
>non-DHS291
GGATGCCTAAAGAACTGACTTTCCTTGATGCTCACTCCAGCCTTTTTAGGACTAGAT
TGGATACATTTACATGTTTGCTGGCTGTTTTTTCTACAGTCACACATCCTACCCATA
ATAATATTGCAATATTCTAAGTTGTCTTGGAGGATATCATCAAGTAGGTGTCAAGTA
AAACTTAATGGTAGACTGTAAATTTTAAAACAAGTTACAGGTCATTTGTGTAGTCTC
TCTCTCT
>non-DHS292
ACAGGTCATTTGTGTAGTCTCTCTCTCTATATATATTAAAAGAAACAAAACTTTTCC
CTCTTGAATCTTTATTCCTATCTCAGCTTCATTTATTGCCTCGTTTGTCATCTAAAT
GGTCTTTGTTCTTTCAGCTTTCAAACATGATTAATTGACATTGTGCAGCATAGCACA
GGGAAAAGGCTGAATATTTTGTATGATGAGTAATCTAGTTAAACGCCCATCTTTCTT
TTCAACTTTACCAACAAATGTGTCTTATGCAGATG
>non-DHS293
CTGAGGGAGCTACTAGGAGGTTTGCACGGCCTGCTCTCCTGCCCTCTTCTTGCTCTG
TGGCTGAACTTCAATTCTCTTCGGGCTTAGACCCCACTGACTCGCTCCCGGGCAAAG
CAAACGATTTGATCAGATGGCCACGTGCATTCTTCCTTTTCCTGAAACCAGCACCAT
AGGGTAAAAGATTATTTCTACTTGGTTGCCTTCCAGATGTTTCACACTTGGACAGCA
AACTGATTTCAAACCAC
>non-DHS294
AAACTCTCCCTTGCTGTGTGCCAGGGACACGCTTCGTTGGTCTCCACGCACGTGCTT
GCCATCTCTGCTCTCACAAATGCTCACGGGCTCCTGGACCTGTGTCTCCTGCCAGGA
GCTGTGCTGGCCTGCCCACCCCTCTCAGCTGCCCACACCTCCGCGGCAGCTGCCTCC
CGTGAGTAGGATCTGAAACACCAAAGCCACGGGCAAGGGCAGGATGGAGGGGCAGCG
GTCCATGTCAACATACACCACAGATG
>non-DHS295
TCATGGCCTGCTCCTCTGTCGGGGCCTGTCACCGGGAAACCCCTTTTTTAGCAGCGA
TGACGACACTGGAACCACGCTAGTCTCCGTCCCTCCCGCTCACATGCGCCCACGCTC
CCCCTCGAGGCAGGTTGAGCCCCGGCCTGGACCGCCCCACGCTGCCTTCCGTCCACG
CAGAGCGGGCTCGGCCCTCAGGGCTTTCCTTCGAGTCGTGTTGGAAAGAAGCAGAAG
>non-DHS296
GATTCCCACTGAGGACCTGTGGCCACACAGCAGAGGCTCCCACAGCCCAGACTCCAG
GGTGGTTTCCATAAACCCCTTGGCCCACTTTTCTTCACTGCATGATTTTTAACAGGT
TTTTTTTTTTTTTTTTTTTTTTGGCTTTATCTCTGCTTCGTACAGTACAGTCATCTC
AAGTTCTTTCAATGAGGGAAACATCACCAACACACTCACAAGTATTGGGCCTGGTGA
ACAAC
>non-DHS297
CACCAACACACTCACAAGTATTGGGCCTGGTGAACAACCCGAGGCCCTGAAGATCAG

GCTCTCCCCAGCCCTCCTGGAGCCAAACCACCCACGGCAGCCAAGTGACAGCACCCG
CAGAGCTGCCCGAGCCTCTGGGAGGTCACGGGGAGGAAGGGAAGACGGAAACACCGT
GACGGCCACTTCTGCCTAAATCGTTAGGAACTATTTCAGAGTCCCTGCAACCTGAAA
CGGGGATTCTCCACACACCTCCACAC
>non-DHS298
AACGGGGATTCTCCACACACCTCCACACGGCCAGCAGCGCAGTAATCGCTTTTAATT
AAATAAATCGGAAACACAAATCAGGCCAATGTGTTGAATCAAGGTGGCGTGCACTGC
GTATTTATAGCTCTATCCACAGAGGACTCCTGACACATGCTACGGGCTCCCGCAGCC
GCTTTATTAAATGGAATAAATAACACCGTGCAAGACGGCAAAACTTCACCAGACGGG
TCTGGGGTTCTCCCAACTCAACACTGTTTTCCTGCA
>non-DHS299
GAATGAAAACATGGTCCAGATGAGAACAGACTGGTGTCTGGTTTAGCTAACGGGTGG
GTGGAAGGGAACCACTCTCAGAGCGATGCTGCATCCTCCCGCTGGCGACTACCCCGA
GCCCACCTGGCGGTCAGGTAGCAGATCCTCACCTGCCCCGAGAGGCGCCTTCCCCAG
GGGCATGCGCAGGGCCGGGCAAGCTTCCAGGGAAGGCTTGCGAGGCCCGAGGTCAGA
AGACATAGGAGTGTGA
>non-DHS300
CTGAGAATGTTGTCAGCTGATGGTTCACAGACGAAGGGAGGAGCTTTGCCCAAGGTG
GCCACCAGTTCCAACTTTACATTTTTAATCAATATGGGGAAGGAGAAATAATCTTAG
TGAGGGTTTCCATACACCATACACTTAGTGAGCATTTCCATACATGGATGGTCCAAG
CATTCTGGGGCGTAACTGACAAGATCAAGAACTTTCCTTGCTGAGCATACTCCT
>non-DHS301
CAGTGCACCAGAGAAAACAAAGACTCGGCCTCTAAGAAAATAAATATGTGGTTCCTC
CCACTGCAAGAGCCCTCAGCCTAGGGATAAGGAGACAGAGGGACTCTGGTATCAAAA
TAGAGGGGAGTGGGTGGTAGGAAATGTTGACTGCATTTGCTGTGATGTGATTTTGCT
CAGCAAAAATTGATTCCATGCATGGCTTGCTATGAGCACTGTGGAATGCAAAATATG
AGACAATCCCTGCCTTAGAGAAG
>non-DHS302
GCACTGTGGAATGCAAAATATGAGACAATCCCTGCCTTAGAGAAGTTTGTATTCCAC
TTTTGAAGCCAGGCCAAAAATACATACAGAGTTAAGTAACGAGAGAAATCAGAATAT
GGTTGAGTGGCAAGAGAGATGTTAGGATAAGTGCCACAAAAGTTCAAAAGAAGCCAA
GGAAAAGTATCTTTTAAAATAAGGTGCCTGGCCAGGCACAGTGACTCATGCCTGTAA
TGAAAACACTTTGGGAAGCTGAGAT
>non-DHS303
GTGTGCCTCAACTGATTCTGACTTCAGGAAAAGTAACACAGAGTGGCCTTGGCCTGT
TGTCTTCCCCTATTTTCTGTCCCAGCTCATCCGTGTCTCTGAAGAACAAATATGCTT
TTGGACCACGAATTCCCAGTTTGGTCTACACAGGCTCGCAGCTCTCACCTGTCCTGC
TGTAGCCTGAATGCTCCCATGTGGGAATAGCACCCCACCACCTGCCCAGTGTCTGCC
TCTGCTCCGTGTCTGGGGTGAGCACCCTCACCCTGAGACT
>non-DHS304
TGTTGGTACATGTGGGACTTTAGCTCATCTGAGGAGTCGAACAGCAGCTGTGAAAGT
CTTCTACTCACTCCCTGTCGAGGCTGGCCTGGTTTGCTACACAAACACGGTGGCCCT
AGGACCATGGGGCTATTCCTGCAGACGCCCTCGGGAACCAGCTGCTCTGAACGTGGT
GGGGGTGTTGGAGCAGCAGTTTCTCAAAAGTCCACACACAGTTAAAGCTCTGAAAAC

AGCACGA
>non-DHS305
AGTGGTGTCTGCCTGCAATCTCAGCTACTCAGGAGGCTGAGGCAGGAGAATTGCTTG
AATCTGGGAGGCAGAGGTTGCAGTGAGCTGAGATTGCACCACTGCACTCCAGCCTGG
GCAACAGAGCAAGACTCTGTCTCAAAATAAATAAATAAATAAATAAATAAATAAATA
AATAAATAAATAGTAAAGTCGATGATTGTAGTTGTTAATCACATCCACAAGACACCT
TCACAGAAACACCT
>non-DHS306
TTGTTAATCACATCCACAAGACACCTTCACAGAAACACCTAGATTAGTGTTTGATTC
CATAAGTGGGTACCACAGCCTAAGCAGGTTGACACATAAAACTCACCATCACCCTAC
CCTATTGCCAGGTTCCCTAAATTGGCATCCCAACTCTAGTCTATGCCTATTCTGAGC
GCCTTCCATGCCATTTCCAGGCTCAGCTTACTGAAGGAAATCTCTGGCCAGGTCTCT
CCTCTGCTTAACTTCCA
>non-DHS307
AGGTCTCTCCTCTGCTTAACTTCCACCACCCCCAGAAAAAGACCAAGACTGACATCT
GCTCCTTTTTAGACTGTGGTCCAAGCTCTCCCTACCCCTCTCCCCTGGCTTTCTGTC
AACATAAACATCTCATTCATGCTCACTCTTGCTCCCTGCTGCTCTCCATCCCATCGC
ACCCCCATGCCTAGGCTTTTCTGTTACTGTGCCCTGTATTAGGCCATACTCGCTTT
>non-DHS308
CTGTATTAGGCCATACTCGCTTTGTTATAAAGAAATACCTGAGACCAGGTAATTTAT
AAGAAGAGAGGTTTAATTGGCTTCTGGTTCTGTAGGCGGTATAGGAAGCATGGCGGG
ATTTGCTTTTGGGAAGGTCTGAGGGAGCTTTCAATAATGGCAGGAGGCAAAGGGGGA
CACAGGCACTTCACATGGCTGGAGAAGGAGCAAGAGCGGGAAGAGGGGGGCGGAAGT
GGTGCTACACACTTTCAAACCACCAGATTTC
>non-DHS309
TTAACCTGTGAACATCCAGCTGTCCCTGGGCTGTGATTCAGTGTCTGTCTCCCGGGT
GACCTCAGCATGGGCTTTGAGGAAGGGGAGAGAGTAGTTTCTTCTGAGACTGGATAG
TGACTCAGGGACCCGGGGCTGGGGCCTCAAAAGTGCCTTTGTTGGCCTGGGCTCAGG
AATCCAGAGAAACTGGTCAGGAGGAGGCCCCAGTGACAAAAACCCCTCCCTCTGCCC
CCGCCCCTCTGCCAGAGCCATATAACT

>non-DHS310
CCTGAATGATGACTGCTGAATCACGGGAAGCCACGGGTCTGTCCCCACAGGCTGCAC
AGGAGAAGGATGGTATCGTAATAGTGAAGGTGGAAGAGGAAGATGAGGAAGACCACA
TGTGGGGGCAGGATTCCACCCTACAGGACACGCCTCCTCCAGACCCAGAGATATTCC
GCCAACGCTTCAGGCGCTTCTGTTACCAGAACACTTTTGGGCCCCGAGAGGCTCTCA
GTCGGCTGAAGGAACTTTGTCATCAGTGGCT
>non-DHS311
CTGAAGGAACTTTGTCATCAGTGGCTGCGGCCAGAAATAAACACCAAGGAACAGATC
CTGGAGCTTCTGGTGCTAGAGCAGTTTCTTTCCATCCTGCCCAAGGAGCTCCAGGTC
TGGCTGCAGGAATACCGCCCCGATAGTGGAGAGGAGGCCGTGACCCTTCTAGAAGAC
TTGGAGCTTGATTTATCAGGACAACAGGTAAAAAGAGGTGAAACCTATTATGTGTGA
GCAGGGCACAGACGTTGAAACTGGAGCCAGGAGAAGTATT
>non-DHS312

AGGAGAAGTATTGGCAGGCTTTAGGTTATTAGGTGGTTACTCTGTCTTAAAAATGTT
CTGGCTTTCTTCCTGCATCCACTGGCATACTCATGGTCTGTTTTTAAATATTTTAAT
TCCCATTTACAAAGTGATTTACCCACAAGCCCAACCTGTCTGTCTTCAGGTCCCAGG
TCAAGTTCATGGACCTGAGATGCTCGCAAGGGGGATGGTGCCTCTGGATCCAGTTCA
GGAGTCCTCGAGCTTTGACCTTCAT
>non-DHS313
AGTCCCACTTCAAACATTCGTCTCGGAAACCCCGCCTCTTACAGTCACGAGGTAAGA
AGCAAGGTTTCATTTAGGGGAAGGGAAATGATTCAGGACGAGAGTCTTTGTGCTGCT
GAGTGCCTGTGATGAAGAAGCATGTTAGTCCTGGGCAACGTAGCGAGACCCCATCTC
TACAAAAAATAGAAAAATTAGCCAGGTATAGTGGCGCACACCTGTGATTCCAGCTAC
GCAGGAG
>non-DHS314
AGACTCCCTCTCGGGGTAGGTGGGGGTGGGGAAAAAAAAAAAAAGAGTCAATGGGCG
CCAGCAACACACCAGGCCCAGCTCCCTGCGTGACTCCATCCTCAGCAACTCGGGGTA
GTTAGTGTAATTAACCCCCTTTCCACATTGGGAAGCTGGGCCCAGGCATATGTCATG
TATCTCCCAAAGGCACTGCTGTTGTGACTAGTAAGAGGCATCGGGATCCGAGCTCAG
GTCTCCTGGGTTCTGATCACT
>non-DHS315
TGACTTTTCAGGAATCCCATGCAAAATTCTCCCTCAAAACATTCTCTTTGTTTGTGT
CTTGGTAGCAACTCCAGAGTTTTCAGGACTTGGACTCTGCTTTAGACTTGTGCTCAG
ACTGTCTCCACAAAAGTGGTCCGTTTCCTCATCCAATGAGCTCTGCCTGTTTGTCTG
TGTGACACTCCAGTGGAAGGAAGCCAAGAGCCTATAATAGCGATCCTGGGGAAAATT
>non-DHS316
TGCTTGAGAGCATGATACAATCTGAGGTTTCCCATGCAACCCTGAGCTGTATGATTA
TCAAAAAGGTGAAGTGAGGAATACAAGAGAAATAAATCGTCAATGCTGAAGTTAGAC
TCAGGAGATCTGTTTGTCCTGATGAACTGACATCTTTGCTAGTAAGTTCAAAGGTAG
ACAGTGTTTATTTCCTGTTTCACTTCCAGATATTGCCTGTGCTTTTCCAGTCACTCA
ACAAAGAAGG
>non-DHS317
CTTGCCTGCAGAAATATGTAGTGGGCCGCGAAGTTTGAAGGAAGCCCGCAGTGCCTG
GTATATTCACCAACTCCTCCAAGTAACTGATCATTGCCAAGGTCGAATTAATAAGCG
TTCTTTGATGTTAATGATTGTGAGTGTTCTCAGGAAAGGACTAAAAGCTGACTTTCT
AAACAGGATGTTGTTTGGAAATACCCCAAACAAACTCGATGTCCATTCCTAACGTGT
TATTAAGTGAACCGATCTGACTCGA
>non-DHS318
CTGTACTCATGCATTTTGGTTTAGCAGTATGCTTTTTTCCCCCATGAATTCAGCAAT
CTTAAAATATGCTATCGCACAAGATACTCATGGGGGATTTAACACCAAATTTTGTTT
TGGAACCTATAGTCACTCACTGTTCCAATCTGAAAAATACATAGATGTATCTCAAGT
GTATATATTGGTTACCAGCTGGTAGTTTCTCAAATATCTCCATTTCTTTCAGCTGCT
TCACACTCACTACCAAGCAAA
>non-DHS319
ACAGTGTCAGTTACGGGGTAGGATTATAACATTAGAAAGAACAGGTGTCCTTGATTG
TTAGGTTTGTCAGGGAAATAGAGAAACGGTGTGCAGAGATTTTGATACAACGACTCC
ATGCAATCTGCACACTCCTTTTGGGGGGCTCCCTCTGATGTTAGTTGACAAGCTTCA

CAAGGATGCAAAGTAAGACAAATTAGTCCATGATATACAATTAGGGGGGACATTACT
ATGAAGAAAGTCTTGCATTTTCCGAAT

>non-DHS320
GGAGATCTGTGAGCTTGGGAAGCGAGAGTGCGTGTGCGTGCACGCGCGAGGGTCACC
GGCGCTTCGCTGGGGACGAGGCGCCGGGGCTCGCGGTGGACGCCGCTTTCCGGAGCT
AGGGGAGCCCATGGACAGTCCCTGGGGCTCTGGAGTGGTTTCAAGGCTTCTCCATTC
CAGGGAAGCGCCAGGTCCTGCTGTTTCTGGTGCCATCCGGAGACAAACAGAGAT
>non-DHS321
TCTTTCAATTATGGAGAGGGTCGTGATGTGTATTTTAATGAAAGCCAACTATCTTCT
CTAATATTTGAAAAAACAATGTTTAAAAGGCTTCTATTTGTACACCTTTTCACATTT
TAGCTGGCAGAGTTCAGGACTTGATGGAAACATAACGAAGGGCTCAGAATGTACTCA
TCGCAGACTGAGCCCATATGGTCACAGTGGGGAGAGCACGCTGCACTCAAGATGCTA
GCAAGCCTG
>non-DHS322
TAGCTTTTCTTCTCCACGGATCTTGTAAAGAAAAAAGTGAGGGTGTATGATATATGG
CCAGAAAAACCTAAATCAGAAAGAAAGAACATTGATCTAGATTGCAGTGGATATTGG
TGGATTTTTTTAAAGCTCTAAACTTTAAAAAAAAATACTCAGGAGTCCCATATAACC
AAGAAAGAAATAGAATGACTGTGATAAAGGGTAGGATCCTCTGCCCTGTATCCTCCT
CTTTGCCCTTTA
>non-DHS323
ATATCTGTGGTTTGCAGACATTGCTAAGTGATTCCTATGTCAAAATTTTAATATAGT
CTCATAACAGCACTTTATCTGGCTGAAACTGACTGCCTTTTTCTTCAAATACATGTT
TGCTTAGCTATATATTTTGCTACATAAATTAGATATTAATTTGCAAAGCACTTGAGA
TCTAATTAATTATAGTAAATTTATTTCTGCTTTATCTCAACAACAGCTCCAAACAAT
ACCCT
>non-DHS324
TGTGTGCTCAGTGCTACTGTGAGTCAGGGTTAATTAGCATTAGTCATAGACTTGGGA
ATCCTCACAAGAAAAGGGTCATCTGGTGGGTCTGGGCTGTGCCCTGGCTGTGATGAG
ACACCACTCATGCCCCTGTGAGTGTGTCATCATGCTTCATTAGTGGCTTGCCTAACT
TTGCAGGTCGGCCCTGGGGTGGCCCAGCCCTGAGTGCGGGTGCTGAGATCAGCCAAA
GGATGACACCT
>non-DHS325
ATATCATCATGGCCATCTTCCTGTGGACTGACTACGTGGACCCAACTCCTTCTTCTC
AGTGGCTGCTTAACATTTCAGTACATGCAAATGCCATTATATTTTATTCTTAGTGGG
TTCTCTTCCCATTGTTTGTGGAGGGCCTGTCATGGACAAGGCTATGTTCTGGGGACC
AGAATTTACATTCTATTAAGGGAGACAGGAAATATACACTAATTAAGTTGATAGGCA
GGAGCATTGT
>non-DHS326
GCTTGGCATGCATAATACAATAGCAGAAAGAGGAATCTTCTACTGAAAGGCACTGGA
AGAGGGATTCTTGAGGACAAGATGGGCTGAACGTCAAGGATGCCATTGACTGTCAAT
AAAATGGTTGTTTCTCCTCCACTTTCTTCTTCTAAACCATCCTCCCTCCCCACTGTT
GAAGATGTAAAATCAAATGGAAACATCTGATGGCTACTTGTCACAAAACCTTTCA
>non-DHS327

GGACTCACACATTTCCTTCTCTTAACACATTTACCACTTACTTTCTGCATTTTTTTT
AAACCTATTTCTCTCTCTTCAAAGATGTGACTACAGGAATCTACAATGCCTAGACAT
AACAGAAGGCAAATAAACACTTGAGAAATCCATTAACATCACAACCTCATTCCTTTC
CCCACCGTCTGTACAGCATCCTGACTGTCCACAGGGGTGCCTAATTATACAACCCCA
AGTCCTCCTTCACCCTAGAATCTC
>non-DHS328
AATTTGCAAGTTTGGTGTGGTGAGGCCTCAGTCTGCAACAAAGGGTGAGGGAGGCCT
CAACGTAGGAGCAGAGGGGTGGGGCCGGCAGATGGCACTGCAGAGATGCAGAAGGGC
AGCTGGGCAGGGATCCGAAAACGACTTTATTGAAGATGGAGTTGGCAAGACCTAGCC
CCACTTCCCACCCCCAGAGCCGTTCCTGTCTCCCTCACTCACACGCTTTCCACAAGC
TCTGCACTTC
>non-DHS329
AGCAGGAGAAGGAGGAAGGTTAAGAATGGTGACAAGGCAAATTCGATTGGAGGAAAA
TCAGGACAGAAGTCCATGACAGAGTTGAGAGTTTGAAGGAACAAATAGGTAACATTG
TTAAAAGCTAAGTAAAGAATGCAAATAGAATAAAAATATCTCTGGAACTGATGATGG
CAGGTTATCAGTAACCTTTAAGTTTTCAAATTCTAAAGATTAGTGAGGGTAGAAATG
TTTACAGTCTCAGGA
>non-DHS330
AAGGCTGGACTAGATAGCCATGAGCCCGCAGTGCCAGCTGCCAGCCTTAGGCGTGGT
CCCGGTGCGCTGTGGGTACCAAAACATTTCTGAGTGGGGTTTCCTTTGGCACAGGGG
CTGAAGGGTCCACGGTGGAGCCAGTAACAGGCCTTCCAGGTAGCTCCAGACTCCCTG
TACCTCTGCCTCATGGCCTCCCTGTGGGAAGAGCAAGCCCAGGGACCCCTCCCCGCT
TCCCCCTCCCCTGCTTCCTGGAGAGGCTGAAACT
>non-DHS331
GCTATCCCCACTACCTGAGTGACTTCCGAGATGCACTTGACCCTCAGTGCCTGGCCG
CAGTCATCTTCATCTACTTTGCCGCCCTGTCTCCTGCCATCACCTTTGGGGGGCTGC
TGGGTGAGGAGAGCCTTCAGGTAGGGGGCGGCGGGGACTGCCCAGGGCCTGGCCACC
AGCTCCTGAGCTGGTTCCTACACCCCTAGGAGAGAAGACGCAGGACCTGATAGG
>non-DHS332
AGTTCTGTAGCAGCAACCACCTGGAGTACCTGGTGGGCCGTGTGTGGATCGGCTTCT
GGCTGGTGTTCCTGGCCCTGCTCATGGTGGCCCTGGAGGGGAGCTTCCTGGTCCGCT
TCGTCTCCCGCTTCACCCAGGAGATCTTCGCCTTCTTGATCTCACTCATCTTCATCT
ATGAGACCTTCTACAAGCTGGTGAAGGTGGGCAGGCCCCTGCCGAGAGCTGGGCCAG
GAGGGCCGAGGTCTCAGGGCTGGAGGGAGTCTCTTGGTCC
>non-DHS333
ACGTATACCAGGGCTGCAGATTACTCTTTTAATTAATTAATTAATTTGAGACATTCT
TACTCTGTTGCCCAGGCTGGAGTGCAGTGGCGTGACCTTGGCTCCCCGCAACTTCTG
CCTCCTGGGTTCAAGCGATTTTCCTGCCTCAGCCTCCCAAGTAGCTGGGATTATAGG
TGTGCACCGCTATACCTGGCTAATTTTTGTAATTTTAGTAGAGACGGGTTTCACCGT
ATTGGTGAGGCTTGTCTTGAAC
>non-DHS334
CTTTCTTTCCCTTCTCTGTTCCACATCCACACCTTACTCTGATTGGGAGTCATAGCT
CTGTTCTCTCTGCCCCAGCTGTCCCGGAATCCCTTTCCCTGCCATGGGCATGCTAGA
AACATGCTGCAGTGCCCTGGGCCACCTCCGGCTCCCGCCTCCTGGCCTCCTGCACTC

TAAACATACATACCTGAATGTGAGAGTTTGTCCGGGGTGACCCTCTACCTCCTTCCA
GAC
>non-DHS335
CTCCTTCTGGCCCAACAATAAGTGCCATTTGGAATCTTCTCGGCCTTCTCTGTGCCT
TCCGCGCGTCTGGGCTCCCCGGTACCGTAGACCCGGCCCCGAGCTCCGCCCCCTCGC
CCTGGGCTTCCCCGCCCCCTCGCCCCCAGTCTCCTGGGCTCTTCGGGGCGGCGGAAG
AGCCGAGAACCTTCGCGAAGGTGGGGCGGGCGCCCCCGAGGTTGGGCCCGCGGTATG
GGCAGTGGAAACT
>non-DHS336
ATTCTCAAAACAACACTGCTCACGCTGATCTGTTGCTGAAAAACGTTTAATTACTGA
CAGAATAAAGTCTAAACACCTTTGATATTCTTCTATGACCTGGCTTCTAGTCGCTTA
TGTACCAGTCCTCTGATAAACTTTCTCCATTTTATCCCATCTTTTCTGCTTATTTTT
CTCAAACTGTGGCACACATTTTGCCCTTTATACTTATTCACCCACCATTCCCAT
>non-DHS337
CCTGCCTCCTATTCTTCACTCCTCAGGCAAAAAGGTTGCTCAAGGAGACTCCAGGAT
TTGGGGTCTGCCTCGTCACTGTCACCAGGAGGCTTTTTTTCTGACTTGCCAGAGCTC
CCCATCAGTCCTCACAGAGCCTCTGGAAGCACAGGGAAGGCAGCTGTGGGATCCTGG
CTGAACAGAGTCCAGGTGAAGCAGGCTTGAGAATGGATTGATGGGGAGGTGGGGATG
TCACAGGTCT
>non-DHS338
GAAATTGAACCTGGGGTTTTGACTTGCCACTGCCATAACTTGTTTGTAAAAGAGCTG
TTCTTTTTGACTGATTGTTTTAAACAACGATTTCTCCATTAAACTTCTACTGAGCAA
ATGGTTAATAAATTGGTTTGCTGTTTTTAAGTGAAGTGACAGAAGCAGACTCTGCAC
ACACACAACCAGCGCTGCCGCTCCCGGGAGGCAGAGGTAAAAAGCGCATGGCACTGC
TGTGTAGAAAGGAGGATAGGCA
>non-DHS339
CCAGAGTCCCTGAATCAATGATAATTAACATTTTTAAAAAATTTATTATTTTTAAAA
ATAAATTTAATCGGGAAGCTCCGAGCCAGAAGAGTAGCGTCTGAGACTCCCCATAAT
TAACATTCTTCACTCCTTAATTTTAATTTACCTCACACCAAACTAGCTGCTAACCCT
CCTCTCCTCATCTGCCACATTCCCCACAAACCTTGAGCTGTCTTCTTTGCTCAGTCA
GTTCCTCAGACTCAA
>non-DHS340
GTAAAGGAGCACCTTCCCTCATACCACATATCCTACATTGTGTAAACGTGTAATACG
GTTAATTGAGGAAGGTTACATACAGAGAACTAGGAAAATGTAAAACTGTTTCTAAAA
GGCCTGGACTCCAAGTTAGGAAGATAACATCTCTTTTGTCTATATCAGAGAGGAAAA
TTCTATTTCATTATTTTTTTCTGTCAGGAGTCTTCTGCAGACTTGCTGGAGACTATT
GAA
>non-DHS341
GTAGTTGAACAAAAGGGCCAGAAGAGAGCATTAATGTTGTGACATTTTGCTGTTTTC
TCAAGCAGAGATCCAGAAAGTGAGACAGATAATGACTCCCAGGAAATATTTAAGTTG
GCAAAAGATGTGCTGATTCAAGGATTGATCGATGAGAACCCTGGACTTCAGTACGTG
AATAACTGGGCTGGGACAATTAAATGTTAAGGGTATTGCTTTCAAAGGTAGCAT
>non-DHS342
CACTGTCTTTAGCAGGAATTCAAGGCTTTTCAAGATTGAGCGCCAACCTGTGACTTT

CCCCTCCCTCATAAGTCTGAGCACCCTCTAACCAAATTCATTATCTCTGGAAACTCT
CCGATTTTTTTCATAGTGTCTAACAGTCAAAGTGTTTTCTCACCCATTTAAGATCAA
CAGTACTTTTTTTTCCATTAAGGACTTAGTGACTGCTTGTGTCAGATGTAACTTCAT
TCCTCTGGCTCCTTTAGT
>non-DHS343
TTTTGTGGACAAACAGTACTGACCTAGATGCAAAAGAGAAAAGACAGTGTAGAAAGT
GGGGAGACAAATGTATGTGAAGTGCAGAGATATGAAAATGCAGGACTCTTGCACAGA
ACAGTAAGGAGCTGAGCGGGACAGAAGCACTGGTTGCCTGCAGAACTCTGCAAAGAT
GGACTTGGGCAGGAACTGAATCACACATTTCCTTTGTGCTCAACAAGGTGATTTGAA
CT
>non-DHS344
GAATCACACATTTCCTTTGTGCTCAACAAGGTGATTTGAACTTTACCTTAAAGATAT
AAGGACACATGACAAGATTCAAAGAAATGAAATTTCTAAGTGAAATTTTTATTTTAC
AACATTGCCAGCAGCTATGCAAACAAAGGACTGGAAGTTAGCTAGAGAGAGACTCCA
GCTAGAGCAATGGTTTGCAGTGGTTTTCAGCTTAGGATGATTTTGTTTTGTCCCCCT
CCATCCTCCCAGGGAATATCTGGCAATGTCTGGAG
>non-DHS345
CAACTGTGCAGGGAGAAACAGAGGGATCAGGAATTCAACACAGCAACTCCACGCCCT
TCCTCCTGTTCCACACCCCGCAGGACCTCCCATCACAGAACCCGGCCTGAAGTCCGA
GGAGGGAGCAGGGTGGGAAGTGTGGGGATGAGCATGGAGGGACCAGGGACAATAGCC
ATCCAGGTCTCCCAAGACAGAAAACCCAAAGTGTGTGGGTTAAAATATCCTTTGAGG
>non-DHS346
AAGACAGAAAACCCAAAGTGTGTGGGTTAAAATATCCTTTGAGGTTCTAACTTAATT
AATCATTATGCTCATGAAAAATAACTCCTTGCATTAGAAGCTCTTATTAGGTATCCC
AATCCTTCTATTCTATGACGGACAGAGGGACACAGTGGATTAGAGCACTAGAGACTG
TTTCCCAAAAATCTGAGGGGAAATACACTTCTGTGACGTGTTTGACCTTGAGACAAA
CGATTCTGGTGATCTCCTGTGTAC
>non-DHS347
CCTGTGTACAAATATGCAAGGTCTGATTTAGACAGCAGAGACGCCCACAGCTAGGAA
ATTGTAGGTCATGGCCCCAAACATCAGAGGGTACTCAAGTACCATCCCTATTTTAAT
GTGAAATGAGGTACAGTCATCCTTCATTTGGAAACAGCAGCAACTGCAAACCAAACA
ATCCTTTTCTAACTCTTGAAAACTTGTTTTCAAAGTTTTGAGACAGACTACAACAAA
CCCTGTATGTTCCCAGACAGGAAGTGG

>non-DHS348
TGAAAGCCTTAGTGAACTTATCTGGGGAGAAATATATTTGCTTTTCACAGAAAGCAA
AATCTAGTTCAAGGACGGTTTCTTTAGATGAAAATTATGTTTAGGCTTACATAATCC
TAACGATGACCACTTTTCTTTTGACCTCTTTTCTAAGGTGTTATGTATTAAGCATGT
GAGTAAGCATTTTGGTGAGCTGCAGTTACTCTTGAAGGGGTCACCACCACCATCACT
ATTGGAAGAAAGGTGA
>non-DHS349
GACTCTTCCATTACCCTGGATTTGTGAATCGTATTTCAAATTTGAATGTGTTTTGCT
TTTCCCCTCACTGTTGTATCTTTTGGTGTGGGATGTTTTGCCATGGAAAGAAGGAGA
AAGAAGCAGGATGCAGTGGGCATACACTTAATTGGGGCTGAAGGACGTGAGGGCCCC

TGTCCAGCCCACCATTCTGTGCCCTGGACAGCCCCTTAATGTGTTAGTGCCTTGGGT
TGCATGTCTGAGTTGAATTCCATG
>non-DHS350
TAATGTGTTAGTGCCTTGGGTTGCATGTCTGAGTTGAATTCCATGAACTGAGTTTCG
TTTCCTGAGAAAAGAAAAGTTTGAGAGAGAATCAAATTAACTGTTTAAATTCACGAA
ATCTTTTGAACAAAAGGGTAGTGGCCACTTGCTTTCTAAATATATCATAAAGGGGTC
AAGAGGAAGTAGCTAGCAATAAGATAATGTATGTAATGACAAATGCCTTGGAAGTGG
A
>non-DHS351
GTAATGACAAATGCCTTGGAAGTGGAAGTTAGTACATATCAGGACAGACTATGTATG
GTTTATGTATCACATACAATTCATTAAAACTAGAACAGACTCTTTCTTTCCATCTCT
GTTACTTCTATCCTTTTTTCTTTGGCTATGGGAATTTTATCAAGTTTGGATGGTGCA
GGCTGTGTACATTCCCCAGGCATGGGTTTTGGAACACAGGGGATCTGACTGTGGAAT
CAAGTTCCATC
>non-DHS352
GCAAGGACTGATAAGACTGCTGATGGCATGTCAGGTGAGTGAGACAAGTAGCATCTT
CTTTACGCTCCTTCCTTTTCCCCCTGTATACCAAGCTCAGATTTTTAACACACATCC
TCTTATTCTACATTCAGAGTTCCCAGAATATATTGTCCGTTGCCACATGATAATATG
GCATGTAATAATTGGCAAGACATGACAATCATATTGTAGGGACATGGGACTGAGATC
CACTGCTTTGATCTACCATTCT
>non-DHS353
AGTGGTGAGTGTTGGATGGCATAGGGGTGGGATGGTGTCGGGGGCTGCTGACCTCAG
CAGCGTCCCTGCTGGGGTCATGCAGGCGCAGCAGCAGCGGCACGAGACTCTGCAGCA
CCAGCTTCCGCAGGGGGCCGCGGAGCCCCAGCCGGAGCCCGCCCCGGCCCCGGCGCA
CCAGAGTCCCAAGGAGCCCGACGGCCGAGGCGCGGATTGAGTCCCGTGTCTGCGT
>non-DHS354
GGGCTAAATGGTTCTACCCCTTACTAGGTTGCCCCAATTAGTGGCACTAGTTGGCAG
AGCTGTTCATGAGCTGCCAGTTATCATTTTGGAGTCAGTTTGAAACCAGCCTCTTAA
CACACTGCTGTTAACTCATAAAAGAGAAGAGTGTTCCATTTCAGTCTCAATAAACAC
TTCCCTCATTCTTTCCCCCTGCCTTTTTCTTCTTGTTATCTGTTGTTTTATCTTACT
TGGATGGGTGTGG
>non-DHS355
CCAGAAATTGGCCTGCTTTACTCCCCTTCCACAAATAAACTTTAAAGTACTCCCTTG
AAGAGTGAGAAGCTGCCAGTGGTACAGGCAGTGAGTTTTATAATTATTTTTTGATAT
TTTCCATGTGGCTTTTGGTCTGAGGGTAGCATGTTTGGGTTGGGTTGTTGGAGGGGT
TTCCCATGGACATGGTTTTGTACCTGCCAGCCATGCAGGATGCAGCATGGTGGGTGA
GATCCTTGCTCCACTGG
>non-DHS356
TGATCATTCATCCTCACAATAGCCATTTCAGGGGAGGCTTTATCTGCATTTTTGGAA
GGAGGGTGACTGAGATCTTGATCAGCAATCAGTACAGAGTTGAACTAAATTGGAATG
AAGACCTTTCTGCCTTCACTGCACACGTTCTCTCTGCGGCTCCACACTGCCTCTCAA
TATAGCCAGTGCTAGACTTAGATCCCTGAAGGGCAACTAGATTTGGATAGTTGATCA
GAAGCCCAGAT
>non-DHS357

TTGATCAGAAGCCCAGATCTAATTCTATAACATTGTTTCTATGGGAAAAAAAATGGA
TTCCTACTTTTAAACACACTTATAAACTTTGGTAATATTAACCCTTTTTAGATGGAG
GGGAGACTTGCTATGCTTGTTGATTTAAATTCCTAAAATCTTGTTGATTTAAATTCC
TTAAATACTAAGACAGTGAGGTTAGTTGAGAATTGGCCTACTGTGCACTGCTGGCCA
TATATTATGTTTCC
>non-DHS358
TTACTTAGAGCTGAGTATTTGGTGTGGTATTGACCTGGGTAATGAGCGTATAACAAA
ATGTTATAAATGAAAAGCTATTTCTGACTTAAACTCTTGATTCTTCTACATTAGTGC
TAGTAAAATTTATGTATTCTTAGTTCCAAGTAGTGGAATTTTATACATTTTATAATG
CCATAGCAAAGCACGATTGGAAATGAGAAGTGCTGTCTCTTAGAAGTTCTCTCA
>non-DHS359
GAAAAGTTGGAAACGAGATCACAGAAAGCCTAGAAATGCTGACATGCTCCTGGTGCT
GGCTGAGGCACAGAAGAGTTTGTCTAGAGATAATTAGCTCGGCGTTCCATTTCCCCA
GACCTGGAACATAACCACGCCTTGGGTCATCAGACAACAATTTGGTGAGATCAACTA
CATACAAAGATCACTGTGGCCCCAGACAGACCAAATAAAAACACAAAAGCAGCTCAA
GGATC
>non-DHS360
ATGTCTACACGGCCAGGATGTGTGCGTGTGTGCGCGCGTGTGTGAACTCCCACATGC
TGCTGCTGTCTGCTTCTGGCCAGTGGCACCGATGCCTCCCTCCTCCCTGCTCGCCCC
CAGATTCCCCTCCCCTCCCTGGTGCTTTTGTCTGGAGGGTGTTATGGGTTTGTGTGT
GTATGAGCGTGTGTGTGTTTTTGGATTTCAGACTAATTTTCTGGAGTTTCTGCCC
>non-DHS361
AATGGTCACTGTGGTAAGATCAGGGGTCTCTGGTCGCCACCGTACAGAGGCACACCC
CAACCCAGGTCAGGACAGAACGCCCTCATCACAAGGTTCACTCAACAATCAACACAT
TAAAAAAAGAGAGAGAGAATACATACTACACGAGGGTACGTCACACAGTTCCATGCG
TACGTTTTTATTCTGCGTAAAGCACCAGGGGCCCTCCATCTGGCCTCCGGGGTTCCG
GCAGTAGGCGTGCCCCCCTCCAAGCTC

>non-DHS362
CTCTGAATTCTCAGTTCCCTTGCTGATGCTGTCAGGTGTGTTTGAGTGAGGCGAGGG
CAGGAATGGCGGTCTCCAGGGACCTGGCTGTCATCCACCTCCACCTGCTCCTTAGAC
AGCAGATGTTTCTGGGCTAGGCCCTGTGTCAGCCCACTGGAGACAGAAAAAGATCCA
GTGATGCAATCTCTCCTTCAAGTTGGCCAGCTTAGTGCACTGGCCAACAGTGCATTA
TAAACAGAGCACCAGAAGAAGGG
>non-DHS363
CATGGGAATTTCAGGAGCTTGGTGGGTTGGATAGCTTTTTCCTGATTGGCTTCCAAA
GTTAGGCAAGGGGAATAGTTCCCCCCTCACTGCATCTCACACTCACTTCAGAAAAAC
TAAAGTAGGAAGAGTCAGAAGTCTAACATCAGTTTAACTGGCCAAAATCAAAGTATG
TGCAGGGCTGCAATTTCTCTAGAGGCTCTAGGGGAGAAACTATTCCTTGCCTTTTCC
AG
>non-DHS364
AGGCTCTAGGGGAGAAACTATTCCTTGCCTTTTCCAGCTTCTGCTAGCTTTCCTTGG
CTTGTGGCTGCATCTGTTCAATCTCTACCTTTCTGAATGTGCCATCACCTCTTCTGT
CTGAAGTCAAATCTCCCTCTGCTTCCCTTTTATCAGAATTTTATAGTTACTTTTGTT

CAAACTACATTCTGCTCTAAATTCACTTGTACATAGCTTACTCATGCTGAAGAATCA
ATTTGAAAGCTCATCCAAGAAGCTTTCTCGGATTCCTACAGTGCAT
>non-DHS365
CAAGAAGCTTTCTCGGATTCCTACAGTGCATGAACTTGCTTTTCCCTGGAATAGAGT
CACAGAAAACATTTGAACTGCTGTGATGTCATTGACAATAGTAACAATAGATAACGT
TTGTGTGGCACTCCCCAAGACTCAGGCATTGTGATAACTAACACTTTCCATGAATAA
TGTCATAGAATTCTCACAAACATCCTGTGAGATAGACACTATTATTTTCAACCCCAT
GTACATATGAGAATACTG
>non-DHS366
GAGTTGGAGAGACTGAGTAACATGCCCTAGATGACTCAGCTAGCAAGGAATGAAAAG
CACATTCTAACTCCACCCACATATATAGTTCCAGGATCCCATCTCTCTACCTCACAT
TAATTAGTTCTATACCAGCTTTATGTTTCCTACTAGAATGTGCATGTTTTAAAGGCT
GAGCTTTCTGCATTTTTATGCCCCTCCTGCACCTGGCACAATCCCTGATTCCATGAA
T
>non-DHS367
GAAAATCACCGTCATCCTAGAGAGTTACAAGGGCTATTTTAATAGAACCTGATTGTT
TTCCTAAATTCACCAAGCCCAGGCAGAGGTCAGATGACTAATTGGGATAAAAGCCAA
CTAGCTTCCTCTTGCTGTTTCTTTAGCCACTGGTCTGCAGGCGTTTTCTTCTTCTAA
CTTCCTCTCCTGTGACAAAAGAGATAACTATTAGAGAAACAAAAGTCCAGAATGCTA
AGGTTGC
>non-DHS368
GCAGCTGGGTGACTCAGGACCCCGGTGGCCGGGCCCCCAGCTCCATGGGACCTTTGT
TTGCTCCGGTCTTTGGCTCTGGCCCTGTGGCCTAGGGCGGTCAGAGGATTTCCTGAG
CCTCGCCCTTGCCCTCTGACAGCTGGGCCAGCAGGCCCCGCTGAGGTCTGGGGTGGG
GGGGTCCCTGAGTCTGGGGTTGCGGCCCCTTCCACTGGGCTCAGGAGAAGATGGAAC
ACAGAGAGGTAAGATGGGGTTC
>non-DHS369
GCCAAGGACCTCATTTAGTTTGTGTATTAAAACCAAAAAGCTTTTGTCTTTAAGAAA
TAAAACCATTTTTTTAAGCCCCAAAAGGTTGCAGGAAGGTGGGTAAGGGCCGCAAAC
AATGTTGGTGGCTGAGATGAGAGCTGGGACCCCACAGGGCTGGATGGCCTTCCTGGG
CCCTTTTCTGGCCTGCAGCAGGCATGGAGACCGCCTCGGCCTGGCTCAGACCAGCAG
CCATGGGAGGCCCACTGCCCTCATATCTGTACTGTTCC
>non-DHS370
TTCCTCAAAGATGACCTTGAGTACCAAGCCAAAGATTTTGGAGATATTTGGTGGACA
TTGGGCATTATACATACCCAGTAAGATATTAGCACCATGAAAGCTCCACAAAAGTGT
CCTGAGCGCCTAGAACAGTACTTGGCACATGGTAGATCTTCAATAAATAGTTGTTGA
AGGAATTTGTTAAAATATCAGAGCTATGATTTAAGAAAATTATTCTGTAATCTGAAT
TTAGGTTGGGAGTGGAAAGG
>non-DHS371
ATTAAAGGGAACGTTTGTCACGTCTGACATGCTCCATCCTCCTGTCCCCCCCACACT
GAACCGTCTCCAAGAACCTTCCGGAGAGCCCACAGTGTGAACAGTAATGAATGCTCC
ATAGCTGGCCGCCTTGCACTCTAGTGGGAACTATGATGTTGGGCACAAGTGCCAGCA
GGAAGGTGGGAAGCTTCCCGGGGATGTTTTTCCTGGAGGTCAAGTCCCCCCTTGGGC
TGTGAAGTAGCCGGTACTGGG

>non-DHS372
GCTTCCACGAGGTGACTTTCTGCAGCGGCCCAGCCCATTCCCTCCTCCCCATAGGAA
GCACTTTGCTGACTGCGATGTGGACCAGGGACCGTTGCATCCTGGTGGATAAATCCA
CCCGGGAGTGGCCTCTGGGTGTCTAGAAAGGAGACAGCCTGACAGAGCAGGCCCTGG
AGTGGCCCTTCACCCCTTTTAGGGGTTCATGCACCCCTTCAAGAATCTGGTAGAAAG
TGGGGATGCTCATCAC
>non-DHS373
CTGGGTCTAGGCGGTCTCTATCCCTCCCCCCAGCTTTTCCACATTCCCTCTCCTGCC
TCCCCGCTCCTCGGCCTGAGAGGGGAAACCCAGCCCAGGGGGGTCCAGCCACCACAC
TTTAGAGACCCAGCGGACCCGAACCCCGGGTCTCCTGATTCCTGGCTCTGGGCTCAG
TTTCTTTATCTGCAAAACGGGAAAACAGTCCAGGCTCTGCTGGCTTCGCCTTGCAGG
GGTGAGGACCAAGCAGGACTTG
>non-DHS374
GGAAAAGATTGCTTCTGATGGAGGCCCAAATGGATATTAATGCTCACTGGCCAGGCA
AGCACTTCTCAGATGAGAATGGCCTCTCTCCCAGGGCCCCCGGAACCGGGTGTCTGA
GCGCCAGGGCTGGCGGGGATTTCCTCATCGACTCCCCTCTAATCGCCCCTGAAGCTG
AGTCCGTCGGAATGACCGGCCTCCCAGCCCAAATCCTGATTGACTTCCCGGGAGATT
GGCTTCATGTCCACCAGAGTGGAAG
>non-DHS375
CTCAGGGCCACAGATCCACTGGGTCCTTGCAAGAGCTGTCCCTGGCCTGGGATGGGG
TTTGTAACCTGAGGACTTTCCCCATGGGGTACAGGTGTGGGGGAGACCCCTTCCCCT
GCTGCACCCCAGGGGCCTGGCTTCCCCAGCACCCCCTACCTCACTCAGCCACGTCTC
AAACCTGGGATTGAGGTTTCCTGCAGAAAAGAATCCTCAAAGCTGCCTTTGGTCCTT
ATTAAG
>non-DHS376
CTGAGGTGCTGTTAATGACATGGGCCACTGGAAGGACAGGGTTCATGAGGTCACAGG
TGAGAAAGTTCTCTGAGAACCTGAAGATGAGGTCAAGTCTGGTACTATTAGAGATGG
CCCTGATGTCCCAGGGCCCCCATGTGGGGGTCTTCAGGCTGTCTTCATGCTGAGCTC
AAAACCCCACTGAAAGGGCAGGGCAGGCTTCCAGGTCTGACCATGCCCCCAAGCAGA
TCCGGGTCCCTGAGCTTTCTAGCTTCTCCTG
>non-DHS377
GGATCTCTGGATCTAAGGCCAATAATGGATGACCGGTGCCACCCCCCAACCTAATGG
GAGATGGTGTTCAGAGAAGAGGTGTGCTCCTCCACAGAAAACTGTAAAATCAAGGCT
ACGGTGGGGGATTGACATGATTAAACTGAGCTAGGAGTGACTTGGTCCCTAGATGTC
TGGGCAGAGGTGGAGAGGCCGAAGATGGGGGAGCTGCTGCTGCACAGTGAGTTTGGT
GATTGGCGTCTGGTGTAAGTGAT
>non-DHS378
ACGGCTGTTGAGTTACCCTAGCTACTTCCTGGGGCGGTCACAATAGTGGCAATAACT
GCTGTTTATTGAGTGTTGACTGTGTGCCAGGCACTGTGGTAGTCATCGCGGCAGCCC
TACGAAGTAGTCGTTAACGTAGAGGAGTAAGTTGAGGCTCGGAAAGTAATTTCCCTA
AAGTCACATTTGACGCGGGGCTGAAAGCCAGCATCATTTAAGACCTTTGCTCTCCCA
GTACTTCTGT
>non-DHS379
ATACTGTCTTTGCTCAGTGTGCTGGAGCCCCAAAGGAAAGCAGCTGGCAGTGGGAAA

ACAGAATGGAACTGTGGTCCAGTATCTTCCTGTAAGTTCTTATCTTGAACTTCAGAA
TTTTTCTTAAGTTGCCCACTTTTTTGGGGGTGGTTTGGGAGTATCTTTCTTGTTTTT
CCTATTAAAAAGTAATTTATACTCATTGTAACAAATCCATCAGTGAATGAAGAGTGG
AACTCTTCCCTTTCCCATAGTCCT
>non-DHS380
GAATTGGGGAGGAGGTAAATTTGTTTCCTGAGGGTGTTTTTCTGAAAGTAGCTGACA
GACTTGGATGGGTCAGAGTGCCAACCCCTGTATGACTAGTTAGTTCATTCTAGCGCT
GAAGCATGGCCCATCCCAGCAGTCTGAGCAGTTAGTAAGCACGATAGCAGTGACATT
ACTGTTTGTCCTTCAGACTGAGTAGTTCCTCACTTGTGGCTTCTACAGATTGCACAC
TTTCAGAAGGAGTTG
>non-DHS381
GTCCCGGTAGATATTGGTGAGCTTGGCCACGTTGCTCTCGCTGACGGGCACGGCCCA
TGTGACGCTGGGGTAAAAGTTGTCATTCATGCTGATGATGAACTTGGAGTCCCTCTT
GGTGGGGCCCACGATGGTGCAGGTCTCTGTGGTGTTGCCGTACCAGGGGTAGTTCAC
CCCATCCGAGTCGCTGATGGCTTGGATCTTGCCCTCCTGGAGGTCGGGGAGCTCCCA
GCTGGACCTGAGGACAAGGAAGGCCAGTTCACTCACTC
>non-DHS382
AGGAAGGCATCAAGAGTGACAACGCTGGGCAGAGCACATGGCTGCGTGGCCTGTCAG
CTGGGGCTGCTTGTGGCTTGGACATCCCTGGTCTCAGGGTGGAAGTGGGGCTGCTGG
GGCTGAAGGGGAAGCAGGGACAGGGACATCCTCAACTACTCTTTCTGGAAGCCAGTG
AGGAAGGAAGACAGCCTGGGGCTTAACAGCAGGGCCAGGAGAATGGATGAATGAATG
GGAC
>non-DHS383
GGCCAGAGGGTGTCTGAAAGGCCTCTTGGCTGCAGGGTGCATCTGCTTTTGGGACAG
CTCTTCAGAGCCATCTCAGAAGGGACAGCATCCGCCTGTCTACTTCAACTCCCACTG
ATGACGTCCATGTGTCATTAGTGCCAATTAGAGGAGGGCAGCAGGCTGAGTGCTTGG
CCTGGGGCGCAAGCTTGTGGGAGGGAAAATTGGATTCCCCGCTAGACAAATGTGATT
ACCCGTGCT
>non-DHS384
GGAGGTGGGTATGTTTGCACTCCGGGCCGTCGCTCCAGGAGAGAGAAGCTTGCTCCT
GAGCCTCCTCCCTGTGCCAGGCGTGGTACCAAGTGCTTCCCCCTGAGCGTCCTGAAG
CTGCCTCAGCTGGCCTGGGTGGGTCTCCCCTTCCTGCCCTCTGAGCTGCCTCTCAGG
GACAAGTTCAGCTCTTTGATGGTTTCTGGAGACAGTAACAGATGCACTTGTGTTCAG
C
>non-DHS385
GGGCTGTTTTGTAATGAGATGCTGCTGGCAGGCCACTCAGAGGCTCCCAGCTGGGTT
GGTGGGACAGCCAGGCCAGATGACCTGATTCCAGCAAAAATAAAACTCAGATTTGGG
CAAAATGAGACTTGGAGTTTGGGGCTCTGTCTCGAGTGGGTTTGCTTTCATCCATGT
GGTGTGGTTTTTCTTCAGGCACACACTGGGACCCCAGTGATGAGAGATGGGACG
>non-DHS386
GGAGATGTGGTTCTCGGACAGGCGCCTCGCTCCCGCCGCCGGGGGCGCGCTGTGGCC
ACCGCAAAGGAAGTGCGTCACGGGGCGCGCCCGGAGCATGCCGGGAGCTGTAGTTCT
CTGGTAGCTTCGTTCCTCCCCGAGCTGCAGGCGGTTCCCACCCGGGGCACTCACCAC
GTGGGACCTCATGGGTTCTAAAGGTAAAATACACGCTGGATCCCAAAGAAGTAACGG

>non-DHS387
CTAGGGTATTGGGATGCATGTTCTGCACTGCCAGCAGAGAGGGTGTGTCTGGGGGCC
ACCACCTATGGGACACGGGGTCGAAGGGGCCTGTACACTCTGTCATTTCCTTTCTAG
CCCCTGCATCTCCAACAAGTCCAAGGTGACAGCTGGTGCTAGGGGCGTGGGGTTAAT
AAATGGCTTATCCTTCTCTCCACCCAAGTTTCCACCTGACCAGGTGAAAAACAAATC
AGAAGGGTAAGATGATGACAGG
>non-DHS388
GATTGTAAAACCTTCCCTGCTCCTGCGTGGCTCTGCAAGCCAATCGAAAATTCCGTC
GTCTATGCCGCCAGCCAATCAGGATGTCCAGAGCGCGGTGCTTCCAGCCATTTGGGA
CTCCAGCTGGCTCTGCGCCTCCAGCCAATCGGGGTGCCCGTCCTTAATACAACCAGC
CAGTCAGTGCCCGGCGCGCGACACCAGCAGCCAGTCAGGAAGCCCTGCGTTCATGCT
TCCAGCCATTGT
>non-DHS389
GTCACCATGAGACAGAATGTTTCCCTCCATATTTTTATCAGGCACTTTTGTTTCCTG
AGCTGTTTTTCTAAGTGTGTGTGTTTCTGGTTTTGCTTCTGGTTGGCAAATAGGTCC
GATATTCAGGGAGAAGATTGGATACTATGGTGTGTCAATTGATCTTTAAAAGAAATA
AGCAAAAGTTGATGACATGCTGAAGCTCAGGGCCAATGCAGATCTGCCTCCCAAGAG
GCCAGTTTCAATTAAGGAGA
>non-DHS390
TGCAGGATACGTCTCAAAATATGACAAACGTTATTTCACCTATACTGTGACTTTACA
TGTAAACTTCAAAAATCACATGCTGTACATCAAATTTTTAGATTCAAAAGATATCCC
TGTTTTCCTAATAATGTAAACAGTAAAACTGAGGGTTTCAGTTAAAGGGATAATTCG
TAACAAAATCCCATTGTTGAAACAATGGTCAAACATAAACATCTTATAATTCAGATC
TGCATTTGGTAACAGGAGAATTG
>non-DHS391
TTGGAGTTGAGTTCAAGCATGTTCACCCCGAAAAGCACTGGTGGTTGTATATTTGAT
GCAAAGTGAGACCCTGCCACACCCAATCTCTGCCTGAAACAGAATCATAATGACATC
AGATGATAATACTGGAGTGACTAATGATTGTGACCTCATTAAAAGAAAAAGGAGACT
ACTTCCTGCTGATTAGAGGGATGGTAGCCATGGATTAGTCATGGCAAAGACAGGCAT
GGTTGGATCTGTCACTCATTTATC
>non-DHS392
GCAGACAAGAGGAACAGACACTTCCCCAGAGAACCTCCAATGCACCAGGCACTTTGC
CTCTCCTTTGTTTAATATAAGGTGATATAGGAATGATTATTTTCCAGGCAAAGAGAT
GGAGGCTCCCAGAGTTTAAGTCTCTAGTCCAAGGTCACATTGCAGTAAATGAAAGGT
GCTGGCCATGGCCCCAAGCTCACAGCCACTTACAAAACAGCAATACCGTTTCCATTA
GGCTTTGGAGGTCTCCACCCAGTATCAGTGAACA
>non-DHS393
AGACTGCTCTCCAGTCCCTCACCCCAGCCCTCGCCTTTCCCTCACCCACCTTTCTTT
GCAGCAAGTTCAAAGGCAGGTCATACACCAGCTAAGCGGGCACAGCTGGGCACAGTT
CCCGGGGCCTGCCGGGCAGCGCTGGGAAGGGCGGCAGAGAGCGCGGACTGGACGCTC
GTCTTGGAATGACTCAGCCAGGAGCAGCCGCCAGCAGCATGGGCCACGCAGCAAACG
GGCGCGCCGTGGGAGCTGGAGAGGCCAGGTCTTCTG
>non-DHS394
TCGAGTATTTTGCCATTGTCAGAGTTTGGCTGTCAGCTTTACAAAGAGGATCCAACT

GCTGATTTTAGTCAAGATCAAATAACTTGTTCGAGAAGGGTTTTGTTCAAGGATGTG
TGTGTGTGTGTGTGTGTGTGTGTGTATGTGTGTGTGTGTGTTCATTTGCTCTCCTGC
TATTGCCTCTTGAACATCACATGCTTTTTATATTTTTTGCCTTTTGAAAATTTACCT
TCGTGTCTGGCTCG
>non-DHS395
TCTCTCCTGTGAAAGCAGGAATTCCCAGAAGTAGGGGTCCTCAGAAGTGTGGAGTAA
GGTTTTGAAATGTGAGGTTCAACAAAGGGAGGGAAGGGAACTTTATCGAGCAATGTA
TGTTGTTACAGATCCCAAGTTTATCACAGTGCTTGCTGTGTGAGCATTTAGGTTCAG
TTGTGCTAAGGGCACCCAGGGTTTCAAAGGAGCCATTTGCTCTTGCTGTTGGGTTAT
GGTA
>non-DHS396
TAGAAGAGTGTGTGGACACTCCAGGGGTAGGGAATGCCTGAGCTAGGACCATCAAAA
ATGCCAGGCTGACAAGCTTTTGGCCCTTGCGTAGACAGTGGGAAGCCACGAGGTGTT
TTCAAGGGAGGGCATGCTACAATGAAGTCTGTTTTATAAAGATCACTATGACTTTAC
TGTAAAAGAGAGGCTGCTGGGGAGCAGGAGACGGGGGCAGAGCAGAGGCTGCATGGG
CAGGTCAGATGGAAGATGGCGT
>non-DHS397
ATGGGCAGGTCAGATGGAAGATGGCGTGGGAGGGACTGGGACTGTGAAGGCGGATGA
GGAGAGGAGGGATGTGAGAGCTTCAGAGGAGCCAAAGGACTTTGGTGAGAGATTACT
TACGGGGGTTGGAGAGGGATTGGAGTGAGGCTGCAGTAAGAGGGAAGAAGGCTGGAC
CCAGATTGGAATACGATTTGGCAGAAAGAAGTGGCCTTTCTGAAAGGGGAATGCCAA
GGAGGACCCTCTCATAGACCAAGCCTTCTGCAT
>non-DHS398
CTCTCATAGACCAAGCCTTCTGCATGGCTCACATCCTCAGACCCCTGGCTCCTGCCT
CCAACACTGGACCCAGGAAGCTGTGCTTTAGGAGGATTAACCCAGTGGTATATTGGA
GCTGCCTTACACCGGTTCACAAGGTCCAGTTATGCACATCTCTTCCCCACAACATGC
TCAGGAACATCACTTTAGTAGCTTGAAATTATCCACAGTGAGAATATTTACACCATG
GGAACTGGCAAACACTACAAACCAG
>non-DHS399
TACAGTTCTGACATCCTGCCCTTCTCCAGGTCTGATTTTTGTCTTTCTCCCTGATCC
TTAAAATGAAGCTCTCCAACCAGGCTTTGGTGACTTTACAGAGCCCAGGGTGAGGGG
GCATGGTCATTTTGTAGGCTGGGTGGGTCCCCAGCTGTCCCCCAGCCTTGGTTTCTA
CTCACCTACCCTGTCTCTGTTGCCCTCCTTGCTCCAATAGTGGCCGTTGTCTCAGAG
GACGACTTTCAGCACAGTTCAAAC
>non-DHS400
CTAAGATGTGGTCACCTTTGATGGGTCAGTTTGTGGTCAATCATCCATCAATAGTAA
TGATGATAACATCAAAATGGGCCCCCCTTTTTTAATCTTAAAACATTTTTCTTAGAC
CTTGAAATGACTCTAATGTAAATAGAAAACAGTTTTTGTTACTCTGCAGTACTGATC
GATTATGATCCCATGCCAGCTGGTTGTTTAACTGGCTTTACTGGGAAGTCATCAGTT
ATGGGTTTGTATCAACAGGA
>non-DHS401
CTATTAATCCTTTTGATGGCAGCAGGCTTCTAATTATAGCTCTGCTCAGTTGAAGAG
ATGTGATCCAATAGCCACAAATATATGTATTCCATGTTGAAATGAATTGGAGCATAC
CGGAAAGTAGTATAAAAACCCTAACAACTCTTTAGCATCCATATACACTCTGTATTC

TTGAGCTTTGAGTAATATGGAGACTCCTGAAACAATGATAGCAGTTGACTTCAGAGC
AGTTGTAAGGCTCAG
>non-DHS402
CAATTGGAGTCAACTTCCATGACTGTTCGGATTTCCTTTATTTTGGGGGCAGTTCAT
CCAAACTTCTATTAAACGGCAACTAGTTCACTTTTGAGAAGTGGTTTACAAGAAACA
ACAACAACAACAACAAAGCAGTTGCGGAGGAAAGAAAAGAGACAAAGTAAAAAAAAC
GGAAAAGAAATCTCCCAGGAGAAAGGGATGTGGAAGCTGAAAACACGGACAATTTCC
ACAGTAAGA
>non-DHS403
CAGATGTGCCTGGTGGAGAGATGGCCCAGAGGTCGGACAGAAGAGACGATCAAAGAG
CAATGGGAGGAGCTGAGACAGGAGGGGAGCCGGGGGCCTGTGCTGCGGGTCTGAGCT
CATTTCCACTAAAAGCTCCTAAAAGGGCCGACATGGATTGGGGTGGGGACCAAGGCT
GGGAGGAAAGCTGAAGAGCGCTGTCCCCCTCCCAGGCTCTGCCCACCTCCCAGGTTC
AGTCAATGGGTCTGAG
>non-DHS404
TGAATTTTCTAAGCGTCTTGTCTCCTTGCCCCAGCATCCAGAAGGGCAGTACACTTT
TCAGTATACAGACTGAGGGAGTGCAGAGATAGAATACCTTTGCAGATAATCTGACAT
GAGAAAACAAGATTGAGAGAAAGCAGGGCTGGATGCTGTGGATGAGTGTGGGGTCCC
TGGCTTACGCCTACTGTTCCATGTTGAGTTAAATTGAAGCCAAAGTCATGTCTCATT
TG
>non-DHS405
TTAGAACTGTGTGATCAGGCCATATGCCCCTCAGTTCCTGAATGTTCACTACCCTGT
GGTGTCCCTTTGCCATGGAAGAGACTCCAACCACACACATCAGTTAAGCTGCCAACA
CTGTTTCCTCCCCATTCTGCTCTGCGAACAACGCACAGTCCAGCCAGGAGCTCAACA
GGGAGGGTTTTCTTGTTGTGTCATGGCTGAGATCAAAGTCATTGTACACCAAGGACA
TAGTGGACAGAA
>non-DHS406
GTCTCAGTTGGGAATCTTTTGGAGCCTCAGGTTAGAATTATTTTGCTTTTCATAAAC
AGGCTTCTAAGACTTTATGTTCAAAGCCTTTATGTTCTCTAAGACTTTATGTTCAAA
ACATGTTTTGATTGATGTCATTATCTTTACAGGGGAAAATGCCTGCTCTAGGAAGCA
GGTAGCCTACTGCGCAGGGCACTTCATCCATCCATCCATCCATTCATTCACTAAGAA
TTGTGCCAGCCCTTTT
>non-DHS407
ATTGTGCCAGCCCTTTTGTTAGGCCTTGGGAATTTTAAAATGGTTAAGAGAGCATCT
ATTTCCTCCTGAACCTCATGGCCTACCTGGGGAAAGAGAGGAGGGAATATACTATCT
GGTCCATGTATCATGACCAGCACAGGATTGCAGGTCTGCACTGGCATGGTGAAGGCT
AAAGATGAGGCTTTTCTGCCCATATCCTGGAAAAACCCTTCCCTTCTGAGCTAAGAG
TTGGTGGGCTTCTCCCTGACAATCTGGTTTCT
>non-DHS408
CTTCTCCCTGACAATCTGGTTTCTGGTGGGGTTGGTGCCACCCTGGTCCCCACAGTA
GTGACTCTTTTCTCTGCACCTTCCCCACAGGAAAAGGGTCCCGCCAGCTCCTGGATG
CCAAAGTCAAGCTGATTGCCAACACTTTGTGCAACTCCCGCCAACTCTATGACCACA
TGATTGATGACAGTATGATCTGTGCAGGAAATCTTCAGAAACCTGGGCAAGACACCT
GCCAGGTCAGAGACTCCAAGTGGTGCTT

>non-DHS409
CTCCAGCTCCCTCCTAAGCTCGCCCGCCAGGCAGCCGCAGCTGCACACGCCCGCTTG
CGGTGGGACCCGCGGCCGGACCCGTACTCGCGCACAGGACACGCAGTGTTCTGCAGC
CGGCCGCGGGTGCTGCCAGACACACACACTCTCTCTCTCTCTCTCTCTCTCTGTCTC
TCTCTCTCTCTCTCTCTCACACACACACACAGACACACACACACACATACACGGG
CACAAGGGCTCTTTCTTCTTAGGG
>non-DHS410
AATCTACTGCAGATGCTGAGTTCCAGGCACCACGCTCCACCACCTGGGCAGGTGGAT
GATTGACATGGGTCTGCACACAGTGGGGCGACCGGCTCAAGGGAACTCACAAAGAAT
TCCGCCTGCAGCAGGAAAGGGTCCAGGGCCTTTTCATGAAGCTCTTCTGCTTGGAGG
ACACGGGAGGCGCTGAGCAGGTACTCGCGGGCGGACTCCTCACGTGGGGACATGAGA
TAGCCAAAGCC
>non-DHS411
CTCACGTGGGGACATGAGATAGCCAAAGCCCTCCTCGTTGCAGCCCGGAGTGCACAT
GTCCAGGGTCAGGGAGACATTGGAACCCCTCCTGGAAGGACCAGGAAAGGGAGTGTG
AGCCTCAGGGGCAGAGGGACGGGCATGAGCTGGGGTCTCCAGGGCTGGGAAAATGAG
GGAGGGAGAGATCAGCTGGGAACAGGGTGGGGTTGAGGAAGAGGAGTGGGAGTGTGA
CAAGACCCCCTGACAAAGAAGCGTGAATCTCA
>non-DHS412
CCTGACAAAGAAGCGTGAATCTCAGCCCTGAGGCATTGGCCTGACTCCCTATCTCCT
GCCTCCTGCAGTGGAGATGTTAACTTAGTGGGGATTTGGGGCTCCAGTTCTAAAAGT
TCCATCCAAAGCTACCCCAACATGGCCTTACCCTGGACTCTCCTCCATCACCGTTGG
AGGAAGTTCTACCCTGTGGTCCCCAGGAAGCTCAGAGAGGGGACACCTGCCTGAAGC
ACAG
>non-DHS413
GAGAGTGCTCTCTTCTGGTCACACACAGCCTCCTTCAGCCTCAGAGACCTGCAATCT
GACTGCCTTCAGTCCTGAGTCTTTTATCTCCTTCCTGGCCAGAGGGCAGGGACCCCA
AAAGACGGGGTCCTTAGGTTTTCCTCACAGCCAGCATCGTGGCACTGGGAACTTGGA
ACCTGGCAACAGTTTCGTGCAATAGCTTCCAATGGATTCCTGTCACAGAGCAGCCAG
GAGAGAGCCAGATTCCAAATACA
>non-DHS414
AGGAGAGAGCCAGATTCCAAATACATGAGGCAGGAAACTAGAAGGCAAAGACAAGAG
GACTTAGAACCAGGGGGAACATGCACAGGAGTGGGTAGAAGACACCCTGGGGGAGGA
TTCCTTCAACCACGAGCACGTGGCACAGGCTGCCCCCTCTGTGTTTTGTGTGGCTCG
CTGTCCTGGGGCCCCGAGGATACAGCCCAAAAGGGTCTGCCTGATCTCCTGTGCCAT
CTTGCTTTGTACCTGGCAAAATCTTCAAG
>non-DHS415
TGCTTTGTACCTGGCAAAATCTTCAAGAAGGGAATTCCCTTGCTGCTTGTCAGAAGT
TCAGAATTTTTCATCAGTTCTCTTGGTGAACTGCTATAGGACATCCACAGACATAGA
AAGCCTTTTCTTGGCTGGGAAACTCTGCTGACTACAGAATAAGCCTTCCAACAACCT
GAGATAAAAATAGGTTGTCTGTCTCCTCCGCTGAATAGCATGAGAGGCCAGAAGAGA
GAAGAGGGGCTGTAAATCTCCTTT
>non-DHS416
ATGAGAGGCCAGAAGAGAGAAGAGGGGCTGTAAATCTCCTTTGTCTATTTTGGGAAA

GAAGTGCCTCACTTGGTTTATTGATAGTCATGGAGGGTAAAGCTGGAAGGACCTCCA
GGACAAGGATTTGCTAACTGTCTGCAGAACCTCAGGGTTCAGGGAGGTCCTCAGGGG
GTCATTAGACAGATAAGTGAAGGGCCAAATTCACCAGGGCACCTCCTCCCCAACTTC
AACCAGAGAAGCCCT
>non-DHS417
TAAACCAGAGGGCAGATAATTGCTGATATGACCTCAACAAAGAGATAAGGTGGAGAA
AGGAATGTTTGCTAATTATCTACAAAGGAAAGAGAAAGGTGAGAAATAATAAGTTAC
CTGATTCTCTGTGTTCTGTGCTTACTTGTTCCTCCAAAGCATTCTCCCCAGCATGTC
TCTTCCAGCTTAATAACTGAAAACACTTAGTCAACAACAATACACTCTAGTGGAAAA
GAGATCCTGTTAGTTTTCTAGACGATG

>non-DHS418
AAAGTTGTGCCGAACTTGTCGCCTCCGGGAAATGCCCTAAAAGGCGACGAGCCCTCC
CGACTCTGCGACACCGGGCTGTAGTAGGCGTCCATGGCAGCGGCCGGCGACTCGCAG
TAAGAGACGCAAGTCTCAGCATTCATGCCTGGCTTGCGCAGGCGGCGGGCGGGGACG
CGAGCGAGGGCGCGAGGACGCCACCGCGCGCCTTGGCTGGGAGTTTGGGGAGGCGAG
GAGGCTGTGGCTGTGCACTCCTTG
>non-DHS419
TCTTTCTTGTCCTCAGGGACCTACCATCCACACCTGCTTATAGGGTGGGGGCTGCAG
AAGTTCTTCTAATTAAGGTAGCATGAGCATGCCAGGCAGTCCCCTGGGGTCTTTTTC
AAGAAGTGAAACCTGGTAAGGCAGAAACTTTTTTTGCACCTCCTTCAGCTATGGTAA
GTGTTAAACCAAAGTAATTGGAGCGAAGCCCAAGGTAGCAGAAGCTACTGATTTCCT
GTCACCTGATGTCTATCAGCGATT
>non-DHS420
GTCTATGGAAGTCGCTTCGTCTCTCAAGCCTCAGTTTCCCCACATTCATGGAGAACT
TGAGTCTTCAGAAGTGAACTAGCAGAGAAGTGTTTCGGAAGCAGCCAGTCGCTCCAC
AGCTGTGAGCAGCAGGGGCGGCGACTTTTCTTTCGCAGAATGATGATGCATGCCTTG
TTCCGAATACACCATTGCATCTCAAAGGCCTGCCGGTGGAGCCCCCGCCATCCTACC
AGAGTGTGATTCAA
>non-DHS421
GGATGAGAAATTGATGCGAGGATCAATACAAGCTTAATTTGAATTAATAAAAGGAAA
TATTTTCTCCCTTTGAACTTATCTCCGTAAAGCCATTGTGCCTCCTCTTGGGGGTCA
CGTGTTCACAATCAATGGCCTTTGAGGAGCTCTTGAGTCAAGTTGGAGGCCTTGGGA
GATTTCAGATGCTTCATCTGGTTTTTATTCTTCCCTCTCTCATGTTATTAATCCC
>non-DHS422
ATCATCACAGACTGGGTGAGAATGGTGAGGGCGCAGATGGGCTGAAAGGGAGAATGT
AGGGGGTCCTGCTTAATTAAGGGGGAGCTAGGGAGGGCCTTTCAAGGGAAGTTTTTT
TTTTTTTTTTTTTTTGAGACAGTTTCACTCTGTCCCCTAGGCTGGAGTGCAGTGGCA
TGATCTCGGCTCACCACAACCTCTGCCTCCCAGGTTCAAGCGATTTTCCTGCCTCAG
ACTCCCCAGTAGATGGGATTACA
>non-DHS423
TCTTTAGGCTGACCAGAGAAACCCACTCCAGAGGGAAGGGAGAAGGGTGATGAGGCA
GCGGCTGGCTCCTCAAGCTGAGGGCTCCTCAAGCTGAGGGCTCCTCACCTGGGACAG
GGGATCCTCATTCAACAGGGAAATGACTGAGCTGAAAGTCTCGTTTCTGTTGTCTGG

ATGGTTTTGGGACAGAGAAGAAATCATGGCTGAGCCCTGAGTGAAGGCTGACTTGGG
CTCCCCAGCACCTGGAACCTTGGTGT
>non-DHS424
ACCTGGTTGTCATTGTCTTGATTGCATTCAGTGTCAAAGGAGCCAATTTGACATCCA
GGAAGACATGATAGCTAAAGGGAAGGCAGTCAGAGAACCCAGGACTGGTGAGGAAAG
GGGTTTGAATGTCGGAATTAGAAGGATTGCCCTGTGGCCCAGAAGGATTTAGCTCAT
CGTCGTCCTGCTAGCCTGCCATCTTTCAGACTTTCCAGGGCAGGTGGCCTGGTCCTC
AGCCCTCAACTTGAGTTAGTCACACCA

>non-DHS425
CATCTGTACAGTGGGACTGTTTGTGCAGCCAGCCCGGCCAGCTTCATTTGCCATGAT
GAGAATTTATCTGAGGGGCGGGAGAGGAAAGCCCTCCCTATAAAGGTACAGGCGCTA
AAATGTCGTGACCTCAGTGGTCCACCTAAAAGTCGTTCTGGCCTGGGTCATCGCCTG
TCGTGCTATGCCTTTGTCCAGCCCCTTCTGGTTGGGAGTTAAGTGGCACCTGTGC
>non-DHS426
GAGTTTACTTCACCCGACCTGATTTTTTTTTACCAGCTTGGAAATTCCTCTTGAACC
TACGTCTCCATATGTCACATCATGACAGGACTAGCCTGAACAAAAGCCATGTCTATC
TAAGCGGAGGCTGTTGACTTCATTCAGTTTGCATATTGTATATAGCAACACAAACAC
TTGACAGGTATATACTCCAGTCGCCACATTTGTCCTGCATAACAGCTTCACTCACAG
GCCTCACCGTCACTTTATTTTGTGTCC

>non-DHS427
CTGCTTAAGACACCCAGTTCCTCCTGGAATCCCTGTTTGGGTTTGGTTTGGAGTTGG
AGTTTGTTTCCATTCAGGGCAACTTGATCCCGGCCCAGCCTCCAACCCCTCACTGCC
CCCGGTATCCCTGCCCCTCAGGAGGGGTCTTGACTTTGCCCACATGCTGACAGGGCA
GGCTCCTCTAGACCTCCCACACGGGCACTGAGAGCTCTGGCTGTCTTTGATGTCCCG
AAAGGAGAT
>non-DHS428
AAACAACAGGAGGCACCTGAAGCTAGCCTGGGGGGCCCACGCCCAGGTGCGGTGCAT
TCAGCAGCACAGCCAGAGACAGACCCCAATGACCCCGCCTCCCTGTCGGCAGCCAGT
GCTCTGCACAGAGCCCTGAGCAGCCTCTGGACATTAGTCCCAGCCCCAGCACGGCCC
GTCCCCCACGCTGATGTCACCGCACCCAGACCTTGGAGGCCCCCTCCGGCTCCGCCT
CCTGGGAGAAGGCTCTGGAGTGAGG
>non-DHS429
CCCGACAGTGTCTTTGCTCAGGGAGGGGAAGTGGAAGGGAAGGGCATGTGGGGTCAG
GACAGGGCCTTGCAGGGGAGCAGCCTATATAACGACTTCTTTGGTCTTAACCTGCTG
GGGCCAAGCTGGTCTGGGTTTGGGGGTTGGGGAGCTTGAGCTCAAGATGTGCCTTAA
GTGGCTTAAAGGGAAGAGCTGAGATTGGGGTGGGTTGGGAGGAAGTGCTGGACATCA
GGCTGGGAGGTGAGGACCAAGTGTGCA

>non-DHS430
ACACAGCCAGAAAGTGGCATAGCCTAGATCTGAATCAGATCTCACAGTCTCCAAAGC
CAATTCTTTTCACCTGTGCAGAGGAAGAACTCCCACAAGAGCCCTATGAGGTGAATG
GTACTAGGGAAGGAAAGTTAAAATATGAGGATGGGGGTCCAGGCCTAGCTCTACCGC

CTACTGGCTGAGTGAATCTGGGCATACCATTTCATCTTCCCAAGCCTCACTGTCTCT
AT
>non-DHS431
GAAAAGACAAACTCCAGCTTTCAGATCTGCCACTGCAAGAAAACCAGGGTTTTTGCT
GTGGTGGCTCTGGGTGGAGTGGTGATGGTGGTGGGGGCAGATGGGCAGTAAACAAAT
AGCCAAACCCCACATAGCCATGTGGATTGGTGACATCCAGGTGACTCTGTGACCCTA
GGATGCACGGGGAAGCACAGAAAACCCTTCTCAAAGACAAAAAACTGCTTTAGTTCT
GTGTAGCAGTGGCTTGAAG
>non-DHS432
TGCTTTAGTTCTGTGTAGCAGTGGCTTGAAGGTTGATCACTGGTATGATGTGCCTAA
GTTAGGATGCAGCTGGCATGGAACAGCACGGTTTTCCTCTGTCCAAAGAAGGTTTGC
AAACAAGAGTAAAATTCAAAACATTTAATAATTGCTGTGGTACAGGCACACTGCCAA
TGAGAATGGAGATCAGCTACAACCAAACAGCATGGTCATGGGGGGGTGTGCCAGCCA
AACATCCACCCTAGCAACAAG
>non-DHS433
CAAACATCCACCCTAGCAACAAGGAAGCCCACCCTGCAAACAGGGAGAAGAGGATCT
GCAGGGTGGGGGCCTAGAAGTCAGCAGAAAATCTGCCTATCTCCTATGGAAATCCCA
GAGAGGATTTCATTAACCTCTCAGGGCTCTGCAGAGCATGATTTAAAAGCCATTGCT
GGGCCGAGGGGGTGGAAGTGGGGAAGTAAGTGCCTTCAGTGTCCAGCTTCCAGGCGC
AGTATTATTACT
>non-DHS434
AGGCGCAGTATTATTACTCCAGTGTACAGGGCCAGCCTCCAAAAGGGCAAAGCCCTG
GTTCCACCCAAAGACAAGTCCATGAAGGCTCTGACAGGTCATCATGCTGTTAATCAG
CATACTTCTCCCTCTGCCAATGAGTATTCATATGTAGTCTCCCCAAGCACTTGTTCC
TGTTCTAGCTTTTATAGATGAGGGCACTAGCACCCCTCACTGCAGTCCCAGGACTTA
GAACACAGCCTTGAT
>non-DHS435
CCTAAAGTGTGCCCATTAATATGAGGATAGATTTAGGCTCATAAGCCTTTTGGTAAC
ACTGAAAGTAGTATCATATAGGCAAGCTCTCCTTATAAGTAAGGCTTTCAATTTTTA
AAACAGACATCCTGCTTTAACAATTTGTAAGATGACTGTGCAGTAATAAAAGTCCTT
TGTATTTCTCCACCGTGTTTTCATTAAAGAAAAATGGAGCTTGTGGGCCACGATAG
>non-DHS436
CACAATGTCAGAGGGCAATAAGAAAATAAAGCATGCCAAGAGGACAGAGAGAAAGAG
GTAGAGGGAGTGCTGTTTTAGACTGCGTAATAAGAGCAGGCCTCCCTGAAAAGTTAA
TATTTGAATGGAGATGTGTGTAAACTGAAGAAGAGAGCCATGATGATATTGAGGTGA
GCAATATTCTAGGCAGAGGGACACAAATGCCAAGTTCCCAACGTGGGAAAGTACAGA
GAAAACTC
>non-DHS437
CTAAGTCACCACTATGGGGCTAGGCACCCACAGAGCTCCAACAGGACACCTCCTCCT
GCCCCATCAGCCCAGATTAGGGAGGGAGTGGGGGCAGGGGAGGATGGAGAGGGCCTG
ATCACTGAAGCCCACAAATCCCACTCAATCCCTAAAGTGGGAAATGGGGAGCAGAAG
CAGGTCTGGGGATAAAGAGTGAGACAGCCTCTCACCATCAATCATGTGGGGCTGACC
CCAAGGAGGATCCGGGGAATGTAGGATGATTGCCCTC
>non-DHS438

ATGTAGGATGATTGCCCTCCACTGTCCCCACCCTGTCATTCCCCTATGGCCCAGTCT
GGCCTACTGGGATGCTGCTGGTCTCCAGGCTCTGCTCTGAAGATCCCAGGTCCTGGG
CCCTCCTCTGCATACTGGGCCTCCATAGGGGGTTGGTCATCCAGCATCAGGCTCTGG
GGAAATAAGGGCCAGACACCCAGGATCCCCACATCAAGGACTGCTGAGAGAAGTCAC
AGCCCAGTCCAAGTCCCCATTCTTTAAC
>non-DHS439
CAGTCCAAGTCCCCATTCTTTAACCCCAGCCATGATTCCATTGCCACTTGTAAACCT
CTACTCATCTTGGAGACTCCCTGTATATTCCCACAAATACCTTGCCAGGCTCCCCTG
TGCCCTCCTGGGGCCCCTAACCCCAATGACCTTACTCTCACATGGCCAGTAGCAAAC
ACTTGAATGCAGATTTGGCCCACTCCAAAGCCTGCACTCCTAGCCACTCCTTCATCT
TCCATAAACATTGAAGGCATCTACTAAAGTCCC
>non-DHS440
GTGCCGAAGGAAATCTGTAATCTAAGGTGCTGCAAAAGTCTCAGTATCTTTGCACTT
CTCCTAATTTGGAACTTCTCTCTTCCTCTCATCTGTCTACCTAAATGTTCTCCAGAC
TTTCTGAAAGTGAGGCAGTGTGCTGGGCACAGGTGACCTTGGAGTTAGACTTCAAAG
GAGAAATGACTTTGGTCTAGACAGACAGAGAAGGGTGCCTCGAGTAGAAGGCACAGT
CTGTCCAGA
>non-DHS441
CTCTGCATGGCTTTCTGGAGTCCCTGTGACAGCCCAAGACCCCCAAGGGCCCACTCG
CTGACCACAAACTCCCTGTCAGAGGGGCAGCCTCTACTACCCGAGGGAAAAGGCTGC
CAGGCTAGACCAGTTCTTGGGCTGTCCTTGGTAGAGTGGTTTGGCCTATGGGCTACT
CTGTCTCTGTGTGGGGGAAGGGATGGCAGCCAGGAGGAATTCACAAAGGCACGAAAT
GGTCCCTTAATCCAACTCAG
>non-DHS442
ACCAGCCAGTATCGTCTAGCTACCTGCAGGACATCTCACTTTGATAACTCAAAGTAC
TTTAATTGTTCACTGTTTCTTCAGTGTTCCTGGTTTCCATTAATGACTTCATCTATT
CAGTTGCCTGAGCAAAAAAAAAAAAAAAAAAAAAAAAATCAAGAAATAATCCTAATTC
TTTCTGCTCTCACTTCCCATGTATAATCGTGTGTCTCTCCATCCCCGTTACCAGTAT
CCTAAT
>non-DHS443
CAGAAGGAGGCAAGAACCAAAAAAAAAAAAAGAAAAAAAAATTCCGAAGCGCCCGCA
GCACTGAGCCCGAACCGGCTCAGTCCCGCCGTCTCTAGAAAGTTCTGCACCCCGCCC
CCTCTGTCTGCGCTCCCGCTCCTCCCAGCCCCTCCCTCGCGGCGGGGCGGGACCAAT
CGCTCCCCGAGCCGCTCCTGACCCACCTACCCCAGCTCTCGCGCCGCGTGCAGAGGT
GCTCAAGCCTCCT
>non-DHS444
CCTGTCAGTTTCCCCTCTCGGCCAGGTTGTGTCCCAAAATCCCCTCAGCCTCTTCTC
TGCACGTTGCTGAAGGTCCAGGCTTGCCTCAAGTTCCATGCTTGAGCAATAAAGTGG
AAACAATAAAACCTGGGTGTCAGACAACCCTTTCTGTTCAGCCTTGGAGTGGTGGGT
ATGGGTGGGTACATAATGGGTAGTAGCACAATCAAGGGGTCACCCACTTAGTTCCAG
TTGAG
>non-DHS445
ATGGGTGGGTACATAATGGGTAGTAGCACAATCAAGGGGTCACCCACTTAGTTCCAG
TTGAGCTTAAATAAGTGAGCATCTCATGTAGATGCCATAGTTGGGCAGGAAGCAGCC

CCACCATGCATGGCTTCCTGAGCACTGCACAGGCTGCCGCTGGGATTTGTGTCTATG
GCTTCGCCCAAGGTTCCCAGCACTCCCTCCCTCCAGCAACCTGGCAGTTTTAGTG
>non-DHS446
GGTAGTGGTCTCAAGTTTCTTTTGGCCAGGGAAGGAAAAGAGAAACGTGAAAGAATT
TAGTCTCAGTTCGTCTTTATTCCTCTTCCACCTTCCACCACTGGGTTGAATTAGTTG
ACCCTGACACGTTTTGTGACCTTGGTGGGAAAGAAGAATCAAAGGCACGTCCTTAAC
GTGCCTCTTTTCAAATGCTATCAAGTATGATGACATATTGCGGGAGAGTCAGTCACC
CCTTGTAGGT
>non-DHS447
TATGACACAGTGCTGATAGCTTGGACACAGTTCCTTTAGAACCTTTATTCAAGTCAT
AGTCGTACCTTTTAGAAATAAGGCACAAACAACATCCAACCCACCCCATCCCTCTGG
GCTAGAGTCCCAAAGAGAAATAAGGGATACACCTGACCTGCAGTAAGGAAAGCAGAA
CCCAGTCTCTGAGGTGGTGAGGCCCACCCAGGGCTCAAAGGTGCCATTGTTTTGC
>non-DHS448
GGGATGGGTTGTAGATAAGAGCAGTTGAGAACCACGCTTCATCTCCCTCCTTAGAAA
CTCTGAAACGAGGCTTTATTCCTACCAGAAGTTCAGATTGCATTATGGTCATCTCAA
TTCCAAAATGTTAGATGGCAAGAATATCTGCCCATCCTTCACTTTCCTTGGAAAAAG
TTGCTCTTCGGGTTTTATATGCGATTGCAGTTTTCCAGTGTGTGAAACTAGGAAAAC
AAAACACTCAACGGTGTACATCCCTA
>non-DHS449
GACATGGGCAAGTCATCAATTTACACTTGTAAACAACTTTTCAGCTAGACCTTGGGA
ACAAGGAACATGATATTCTGTCTACAAACTCATGTGGCTTTCTTATTTGAAAGTTTT
TTGAAAGATTTTTGGTTTTGTTTTGAGGAAATTTTCTGAATCTATTTTGAATGTTAG
AATTTGGGCATTTGCTAAAAGGAAGTTGCTTGAGCTTGGATGTTTGTGCTAAGAT
>non-DHS450
ACTAGAGGAGGAAAGTCACGAAGCCCTCACACTCTGATGCCCAATTCATTTGGGCTC
CCCCGCCGCGGGGTATGATGGGAAGAAATAACCAGTGTGGAGTGGCACTAAGTTCCT
CCATAACCTTACTCTCTATCAATCAAGTGTGCCTTTTACCCCTTTGCTGAAAGAAGG
AGCACTATGGAGAGAGGGACCAGAAACAGGACTCCCCTCACCATGAAACTACTGTCC
CAC
>non-DHS451
AGCCTTCCAGCAAGAGATTGTTTTTCAAGGACAAGGACTTTGATTTATTTCTGCCCC
CACCCTGGTACTGCCTGGTGCCTGCCACAAAATAAGCACTAACAAAAGTGTATTGTT
TAAAAAATAAAAAGAAGGTGGGCAGAAATGTGCCCAGTGGAAGGCTGACCCCATTTA
AGTGCCAACTACTCCAAACCGACATGCTCACGGTCTCTGGCCTGTTCAGTCCCTGCA
AAACAGCTAG
>non-DHS452
AAAGCGTTAGGAGAGGTCAAGAAAGGAGCAGTAAGGAGGCTGAAGGTTACAGGGCAT
TTGAATCCAAATCACTGCTCTGGGCTAGGGAATAGAGCCAGCAGACCAAGGTGGGAA
GGATTCTGGAAGGGGGACATTTTAGTCTCCTAACCCCAAAGCTCAGGGTGGAAGAGG
GGAGAACAAGGAAGCAGAGTGTATAATTATTTTTTCCTTTTATTTTTGGAATCTAAC
AGTACCTGGCA
>non-DHS453
CCTACCTATCGGGTCCTGTGACCACAGGCGACCTCAGTCCCCGAACCATCTTTGCAG

CTTCTCAAACGCACACCCCAGTGCCCTTTCTCGCCACAGGTGTGAAACTACATCTCC
CGACAGGCGGCGTTGTCCCCTTCCTTCCTTCCTTCCTCTCCCAGGCCCGAGCCATGG
CAACAGCGTGACGTGGGGGTGCCGAGATCTGCGGGAGCAGCGGTCCCGGCTTCAGTT
CTAGCCTACCCGG
>non-DHS454
AGGTGATTCTTAGTAACTCCAGTAGCCTTCATTAGTTAAAAACATTATTATTTTTTG
CATGCTGCTTCGACTCTAAATATCTGGTTTTCCCTGTCTTTTTGGTTTACTACTTCC
CCAGATTCAGAACAGAGGAGTAACTAGGGGATCTGATTTTAGAGGCCTTAATTTTCT
GTTCATGGACTGTTAAAAGTAAAACCAAACTTTCAAAAGGGATAAACCTAAATATTT
ACTTGTTATCATTAGAGAGGGAACATCAAATGCTG
>non-DHS455
GGATTTCAGCGTAAACTGAGAGAGAAAAGTGATTTAGAATTTTTTTCCCCATTAAGA
GGTTGTGCTTGCATGTAGCAATATAAAATATGCATTTGGGCAAAGAGAAGGTAGAGT
ATATTATTTTGAGCCCAGAAGGCATTCCATCAGGTTGCCCCACAGTCATGTGTGCAC
TTCACGAACACTCTGAGGACTTATGGTGATAGAAATCATTGCTTAGCTTAGAATTTG
ACTGAAGAGACGC
>non-DHS456
AATTTGACTGAAGAGACGCATAGCCAGTGTGATCCAGGTTGGCCTCATATCTGTGGC
AACACTGACAACGTCCTCCCGGATATATAGCAATGGCTTAGTACACAGAGCTTGTTA
ATTAGCAGTCAGCTAAAATTAGCTGGAGGCAGTGAGGGCTACAGGAGTTCCTCCACA
TCTCTGCATCTCTAGTGCAGAAGTGGTAAATGCCTGGCACTTATAGATTCATTCCTG
ATGACCATGACAGAC
>non-DHS457
GCATGTGAGGAAATAAGCTTGAAACTCAGAGCCTCCAAGATGATAAGCATGGCATGA
AGGAGAATTATTTGGAACTCACCTCCTACCCTGGTCCTCTAGGGACCCTGCTGGTCA
CAGAGCTACTACCACCACCACACTGGCCTCCAGGGTTGGTGCTCCCCTAATTTAACA
CTGAGTATGGCTGAAATAAGACTGGGCCCTTAAACAAAATGCCACGGGAGGCTTAGA
TGTGACAACAGAAACAAGCCCAG
>non-DHS458
GGGAGGCTTAGATGTGACAACAGAAACAAGCCCAGAGGGGACCTTGGCCCCAGCAGT
TAGGGTCTAAGCTGTGGCTACAAAATCGTAGGCTGGGGTTTGCTCAGGGGCCTTCCT
TTCTCACTCCTGGTCACCTCCCTGAGGCCACCACCATTTCAGCAGCAGCACCCAGAC
CATCCTCCTACATAAGAAGTGCCAGGGCTTTTCAGGAGGCCACTGGCCTTAGAAATG
GCCAGTCCTTTAGGGGTGAAAGGTCTCAGCT
>non-DHS459
AGTCCTTTAGGGGTGAAAGGTCTCAGCTTCTGGGGCATGCAGAAGGGAGGGCCCCCT
GAGATGCTTAGAGGCTGAGAGCCTGTTGGCAGAGAGCTGGCTGTGCTGCAGGCCCTG
TCCCAAAGCAAAACTCAGCTCAATCAATTCTCCAGGGAAATCAGAGCTTTCCGGAAC
CATCCAAGACACCAGAGTTGATAAGAGGCACTGCTGAGATTTTCACCTGGGTTACTC
CTGAACTGGTGATAAGCCCTTGTGAT
>non-DHS460
CATGCAAAAGTGGCAAAAGCGTCTGCACTGGGGGCTGTGGGATGGGACTCAGACCCT
GCTGCTTCTAAACAAGGGCGTACTCATGCAGTATTTCTATGAAACTCAACATTGCAC
AAGGTTGAAATTTGAAAACAACAGGAAGGTAAGCCAGCAGCCCGGCCCTGGGAGAGA

CGCTCTTGGCAGCCACCAGAAAGGAGCTATTGACCAAGAGAAGAGGAGCCCGGAACT
>non-DHS461
AGAAGGCAAACTGCAGAAAGGTCAGCCAGATTGCAGTTGGGCAGAAAATCTCATTTA
AACTGTATTATCACAACCAAAAAGCCCTATTTGGAAATCAGACGTAAATAATGCCAA
GCAATCAGGGAGGTAGGAAGGACGGGTGGATCTCAGTGCCTGCCCCAGCCCCTGACT
GCCTCTGACGAGAGGTGGACATCTGTTCTGCTCCGGGGGCAGAGGCAGGAGTATGGA
GCTGTACTGCTGCTT
>non-DHS462
CGCCAAAGTGTCTAGGTGATGTATTACTCTTTATGGTAGAACACCTATTCATTATAA
ACTTCCCCCAATACAACCCCTGTTGTTGCAGAAATCTTAGGCTGTGACAACCATAGC
TGCCTACACATTCCTTGTATCTTGGGGTAAAAGCACACGTGCTCTGGAAGGAATGTG
TAGGTGGCTATGGGTGCACAATTTCAGGGGGTTCGTGAACTCCAGTTAAGACTTGCC
CTAATTATACC
>non-DHS463
CAATGAAGAGCTGCTTTGGAAGCTCCAAACTGGGGACCCGACCAGTCCGATTAAACT
CTCGCCCACATCTCCCGTTTACCGCGGCTCCTCCTCGGGGCCCTCCTCTCCGGCCAG
AGTCAGCACAACACCCAGATGACGCCACTACACGGCCTGCGGGAGCTCCGGCTTCTC
GTCCTCCGGTCTCCACCCTGAGGGAGCACCGACCCGGTGCCGCCGGAGCTGGCCCTG
TGCGCATGCTCAGTAGCTGCGAATGCATCCTAGG
>non-DHS464
AGGCTTTGCTCTCTCTATTTTAGTGACCGCTGACTGCGGAGCAACGCTCTTGTGTTC
TGCGGGCCCAGCAGCTCCGGGAACGGTCAGCGTCGGGGGCCAGCCTGGCGGGACATT
TGGGGTCCCCCATACGTGGACCTATGCCTGTTGTAAGATGTTTGGAAGAGCGTCGAA
AGATGAGATCATGTTGTGTCCTTGGAAGCTGAATGAAGAACCGGCTTGCAAAGGAG
>non-DHS465
CCAGTGCTGGCTCTGTTGTTTATATACAATTTTCCAACAAAATGTACACAAAATAAT
CCAAAAGCATAAGTGAACAACTTTTATGATATATTTTTTACTGTTGACTATAATGTT
TTAAGAACTCAATTCCCCTATTATCTTTCCTTATCCCACAGTAATGTATAAATTAGA
AACCTAACCATACTTAGTAAAATGAGACTTTCAGTGTATGGAATATAATTCCTCAGA
TTTTTAGCAGGACAGTTTTCTTCATTTCC
>non-DHS466
TATATTGGAAAGGGGTCCCAATGTTTTTAAGCCTTTAAATCCATTAAATAAATGCTA
AGTCAGGATTTTTCTTATATGGACACGTAAGTTTTAGAAAGTAATTATATCTCTACA
TAAACTGTGAACCCTGCTCAAAAAATTAGCAAATGCTTACATGCTGAGAACCTAAAT
ATCAAAATATAAAGAAAATGGAGCCTCTAAAAAATCTTTTATTACTCTGGGTTTTTA
TAAGCATTCCTTTTTATAAGCAAACTGAATTTGTGTGCACCCAG
>non-DHS467
CAGAGCACAATAGCCTGTCAGCCTCCCCTGCCCCCTTATAAATTGTGCCCCTGCTGT
CATCCCTTTGTTTCAGGCCCAATCAGCAAACTAAACTTTTGTGAAGAGAGAAGGCAG
TTCAAGTGTTTCTGCCGCAGCTGAAGGGAGGAGCTAATGTGGAATTTATTGGGAGAC
CAATCAGTGCTGGAGGGAAGAATGACCCCAAATTGGAAGAAGGACTTTGTTGACC
>non-DHS468
TCTTAGGAGAGAGAAAGCCTGTGGGGTAGGAGAGTGGGAACCTGAACTTTCAGTTTT
TAATGGCACTGCTTGTCACTAATATTAACACTATGGGGGCTCACAGCATGGGCTCAA

GGTGGTGTCCTCGAGGTCTTCTTTTCAGAAGGCAGGCTGCACTTTCACCAAGAGTGG
GCTGCCACACAGACACAGGCGGTTCAAGACTCTGGCATTTGGCTTTGCTAGGACTCA
G
>non-DHS469
CATTTGGCTTTGCTAGGACTCAGCCGTGCTGCGAGGGAGGAGCAGCGTCTGGATCTG
GATCTGAGAGGCCCCAAAGCATAGGCCGTAGCTGCCTGAGAGCCCCTCATGCAAGGC
AGTGCGCTCAGTCCCAGAGTGGCCGACAGATGCTCACAGGCACGCGCAGGCCGGCCC
AGGGCAGCCCACGGATCTTGGAGGCTCCGTTTTCTCACCTCAAAAATATGCATTCTA
CCCAGGAACTGTGGCCCTTATGTCCAT

>non-DHS470
CCCACTTTAGAGATGGGAATTGAGGCATAACACGTAGACGGCTGCCCACTGTGGAGG
AGCTGAACCTTGGCGGTGGGGCCGCAGGGTCTCTCTGCTCCCTGTGGGACCGCCCTT
TTCTCACTGTTGTGAGCAGGGCTGTTCCTTTCCAGCAAGATGAACACCGGTGTAAGC
GCGTCTTCGTGTCCTTCCATGGATGTTTGTAGAGGTGGAGCTACTGTGTCGAAAG
>non-DHS471
CTATCTCACCTCCATTGACCACACACGGGCATGGGTGCCACCTTCCACCTTGCACCT
GCTGCCTTTACCTTCTGTTCTCTGTCTCCCTAAATATCTCCCAGGGTCCTCTGAGCC
TTCACCGTTCATTTCTCTGCCTGGCCTCAAACAGAAAACGTCAGGAGGGAAGCACAC
AGCTGCACAGGTCGTGCCCCGAGGCTCCTGCTGCCGGCACCTCCCCCAGCCTGGACT
TTGGCTGTGTTTGTAG
>non-DHS472
CCAGAAATTCTTTCCTTCTCCAAGTCCAGTGTTCCCTGTGCTCAGGGAATAGCCACC
CCAGCCTGCACACTGGTGAGTGTGTTCTGGTGCAGAAGCGACAACAATCTCCGGTTA
CAGCAGAAAGTGACCTCCTCTGTTGGTTTACCAATTGCCACTGTGACTGAGGACCAT
GGCTCAGTGCAGAAGCAGCCACTAGAAGCAGGTTGGCAATGGAGGAGAATGACCCTG
TGGGTATC
>non-DHS473
CTGTGCTTCCTCCTCCTAGACTTTAAGGCCCTGAGAGAGGAAGGAAGGGGTGGGGAC
AGGGGATTGAGAATTCACAGTCCCATTCTCCTGCTACCATGAAAGGTTTCATCACCC
AAGGCTGCTCAGGAAGTTTGCCCACAAACTGCCGGAGATGTGGGTGAGACCGTGTAA
TCCAAGCCAACATAGTGAGCTCTTGAGCAGCTGCTAAGTCAGTGCCGAGAGCAGAAA
TAGGAACCCAGC
>non-DHS474
GGAAAATGAGATTCTTTTGGCAGACAACTTTTTAAGATCGCTCCTAGTAAGCACAGA
TCTCCTTAGCCTTGGTTGCCAAGTGCAATGGGAATGTTGCTTGCAACTTGGTCATCT
GGAAGAGGAAAACATGATCCGACTGTTCAGTCTAGTTGTCCAGGGTCACTTCAGAGT
GCTGTACAGCAGCAGTTTTGCATGCTTGTGATGTCCTGTCTACATAACTGTGTGA
>non-DHS475
ACATTTTGGAATGATCAGGGAGAGATCTTTTTTAGGAGCCCTTAGAAAGCAGACAGG
TACGAATACTTAAACTTGATTCAGCTTTGACTGTGAAACAGTTTCCCTCTGTGGTTT
CTCCCTCCGGTTTCACAACCCTTAAGAGGCCTGCGGTATTCTTTACTTATTGATTGT
AAGTACTTTACTTATTGATTACTTACTTGTAATTAGCACTAGACAGTGTGAGAAATA
CAATCAAATTCCAGATATCCTCTGTCTTCAC

>non-DHS476
TTCCAGATATCCTCTGTCTTCACGCAAAGGCTGGAAAAGAACAGTTCTGTGTATTAG
TTCAACACAGCATTTACTTTCCTTTTTTGATATAATCTTTCAAATGTTTCACAATGA
GCTAAACCTGACTTTGTTTTCAAAGGGGAACATGCACTGGAGTCTAATGAGTTGAGT
GGCACACACCATGGATGCCCTCTCCTGCTACAGGAAGCATCTGCATTGTTGATCATT
CAGTCATTATGTAGAGTACCATCATTGGGTGCTTTAC
>non-DHS477
TGTGTTTCCTGGAACTAGAGGACTATTTGTCTCATAGCTTTTATTAAGCAAACTTGA
TATAACCACCACACAGTGGCAAGAGATAGACTAGCTGTTTCCAGCTGCAGAAAATAT
TGAATTCTACCCTAAAGTACAGATCATTTGCAATATAACCCATAATAGTTAAAGCAA
ATCATAGTGCAGTTTGTATTGAATTTCCTTATTTTTCCTTTCAATGGAGTATATTCA
TTTTCCTGTCATTTGAGATCAGTTTCT

>non-DHS478
TTACCAATAGTCCCACCACTCAAAAACAGCTCCTGTTTAAAATTTGGTCTACTTCCT
CCAAGGCTTTTCTTCAGCCTCGGGTTAGTTAATATTTTTAATTTTAAAAATCTCATG
GGTTTTATTTATATCAGAAAACATAATCATAGGTCATTAAAAATTTTCATCATCTGT
ATCTAACTACCTAGAGAAAAACAGTGAACATTTCAGTGTACATGCATCTCTTTCCTG
GTACACATATG
>non-DHS479
GGCTCAGTCTCAGAGATTTCTTCAACATGTAATTTTCTAATGTTTCCTTTCCTGCAA
GGAATGAGACAATATATTCTTGGAATATTAAAGTACTTTTGAAGTAACTCATACGTT
TTCTCCCCTCTTCCATTTGTTTAGCTATTGAGGGTTTGCTTTAAAACAAATTTATGT
AGAAATATCAGTACTATAATAGTATTGTGATACTGTGTATTTCTTAAGCATATGCCA
GGAAGGAGAGCATTA
>non-DHS480
CGGATTCTGTTGGAAACTAAGAGGGGAAATCCAGGAAGGCTTCCTGGTGGAGCTGAT
CTTTAAGCTGAACCTTGAAGGACTGGTAAGATGAACGGATGTTCTGGAACCTGAGAA
ACACCCAAGTACCAGAGAGACAGCATCCATGTGTGAACTACTCTAGACAGAGGCAAC
AGGCGTGGTGGGGCCTGGGGCTCCTCAGAATGGCACCTCAAGTCCCCATGGTGGTCA
GGGACTGGAAT
>non-DHS481
CTGTCACCACCATTTTATGCATTCAAAAACTGTTATTTTATTTTATTTTTATTTTTA
TTTGTTAAATGGTCCTTTTCCTAGACTGCCAGGGTCAGCAAACAGAAGTGTCTGTTC
AAAGAGGGAGGGCTGGACGGGAAGAGGGCTGCTAGGCCCGGTCAGAGAGGACTTCAG
GCAGGGAGCATGGAAGCTGTGGACAGGACAGCTTTTAGCTCTGGGAGCAGGTGCTGG
AGCCAAGTCACACACAGGTAGCAGTCA

>non-DHS482
CACACACAGGTAGCAGTCAGTGAGGTTCACATCCAGGAACCTTTTTACAGAAACCCC
TCTCTCTCTCTGTACAGCTGTATAAGCACCAGCACAGGCTCCGACAACATGCAGGTG
AGGAAGGGGACAGAAGGGCCTAAGATCCCACCATGGAGAGAGAGGGAGGAGAGGGGA
GGGGGGCTTGTAGCATCAGCTTATCAGAAGGACCCCCTGGTGCACCAGAAGATTGTA
GTCCTGGGTGAACAAGA

>non-DHS483
CTGGGAAGTTATTTAAGGGGTCAGAATGACTGACGTTAGACCCCTGAAGATTGAAAT
CAGTGACTTGTCTCTATCCTGAGGTGTCTTCTGTCAGTGTGGAAAAGTCTGGGCACA
AAGAAGGAACAGAGCATCAGCATTCTCCCCCAGCCACCCAGCCGGGTTGTGAACAGA
AGCACCAGTTTTGCCTCCTAAGTCTCTGTTTTTATTTGCTTTGAAGAAGCAGACAGG
TGCCTTT
>non-DHS484
CTTTCCCAGGTGAGAAATGAACACACACAGAGAGAAACATACGTACCTTCCTACCTT
GCTCTCCTTCATTTTCATTTTCCTGTAGCCAATCCTGTAGGTGACTCCATTACCCAT
TCTAATCAATATTTTATGTACATATATAATCACCCAGAGTGGGAGGCAGCGTGCTCG
CCTGTGTGTGCCTGTGTACAAATGGGTCCATGACTGGACCAGCTGGAGACCCACGGC
AAACAGTCCAGAGAGCAAGGG
>non-DHS485
GGCAAACAGTCCAGAGAGCAAGGGGGGCTATCTTCCCCACCGTCCGATGACCACTTT
GCTGTTGTTAAAGAAACAAAGAACAAATTGTCCTGAAGGTAAAATATGTGGGTTCAC
ACGTAGGAAAAATGCCACATGGTAATTCAGAGTCAGTGACCCGGAGAAAAACAAAGG
GGGAAATTAATTTTTAATGGTACATTTAAAAATAAATCAGAGTGAGGACATCAGTCT
CGGTTTTCCAAG
>non-DHS486
GAACACACAAAATCAGTAGCATGGCCCCATTGTTGGGGTCTCACAGTTTAGCTCTAC
AGATGTTCCCTGGATTATGAAATTCCCATGAGCAAACTTCCCCTTAACAAATTGTTC
TCAGAGGCACTGGATACAGTTTAAGAGATGGGTAACAGTATACATTACTCAAGTAGA
CATAAAGCACTCTTGAGCATAGATGAAAACCAAAGAGAGGAGGCAGGAGCAGGAAGG
AGGAGAATTTTGCAT
>non-DHS487
ATGCTCATCTTTCTGTTGTCGAAGTGATTTGTAAGGCTTTTGGGCTTCTGTGAAATG
AAAGCAAAGAAATATATCCTTAAGCCCTTTCATCAATATATGGGAAATCTGACTTGT
ATACGAAAAGAGTTATTGCTTCAAGTGGGGATGTGTCTGTACACAAAGGCATATAAC
CAAGTGTACACACATAAACATGTAATCAAGTGACAAAAGTACATGAGGAATACCAAC
TGGGAGAGG
>non-DHS488
CATGAGGAATACCAACTGGGAGAGGCCCATCTGCGGCAGTATGCTTCCTGAGTAGAG
AGCCTGTAAGGCTGTGTGGCTACAGGGAGGAACTGAGTCTTGTATAAGGATGGTTGC
GATTTACAACGGGGAAGGGAAAATGGGGGCTACTACTGGAGAAGGTCTGCCAAGGAT
TTCTGTAGACAGAGGAGCTAGTTTCAGGGATATGAATGGGCCCATTTTGATGCAAGA
TCAATGTAGGCAAGCACCATAAAATCAGGGTAGA
>non-DHS489
AAGACCCAGCTCTAGAGTCACCTCCAACCCCTTACCCACCAGCCCCCTCTCCAAGTC
TGTGTCCCACAACCCCCCTGCTCCCTCCAGGGCACCCTCCACCCTCTGGGCCACAGT
TGTCAGGAGTCAGGCAGGGCAGGGGCCGGGTGGTGTCTTCTTTGTGTTCTTGCACTC
AGGGCAGAGCTCAGCACAGAGCAGACGCTCAAAAAACATTTAAAGGATAGAAGCATT
GATTTGTGGGT
>non-DHS490
CTCCTAGAAGCAAACGGACTTTTCCTGGGAAATCCCAGAGGTGATGATCAGTATCTC

TCCCGTGACTCGTAGTTCAGCTCTTCCTCCATGAGCCTGACTATCAGTGGACCTTCC
AGAAAGAGCCCCTTTTCCTTCTCTCACCCACAGCACAGGGCACTGGGAAAATGCCCA
ATGAGTCCTGCCCTCTGGGTTGTGCTTTGGACTTTTCAGTGTGTCTTCGCATCCAC
>non-DHS491
GTGCTTTGGACTTTTCAGTGTGTCTTCGCATCCACTCTTCAGCTTGAATGTTGCAAC
AGCCATGAAAAAAGAAATGCAAAGCGATTCAGGATGAGAGCAATACCCTACTCCAAA
GAAGGCAAAATAGAAGCTCAGAGAGGTCAAGCATTTTGCCCAAGACCACACAGCTAG
GAGTGGAACTCATGGCTGTCCAAGCCCCACGCCTCTGCTGAAGGTAGAGATGAATTA
CAGCAACAAGTCTAGAAAGGTGCC
>non-DHS492
GGTAGAGATGAATTACAGCAACAAGTCTAGAAAGGTGCCTGCCCTATGGTCTGTGAG
TCTTGCCTAAGAATGAAAGAGGAGCCAGTGGGTTAAAGATGAGGTCACCAACAAACG
GTGGTGTTGGAGTTTACCACTGGTTATTTTAATGGGTTTGCAAGAATTGTTAATTAC
TAATGTTTATTGAGCCTAGTGCAGTGCTTGGGGCATTTTGCACATTGTCTCTGATCC
CTATCACAACCCTGAGAGGTAG
>non-DHS493
AAACACCCTAAACAACTTCCCATCCCTTATTATTTCATCAGTTGCTGTATATAATCA
ATAAAGTTCCCTGACAGACATCAAGGTGTTATGGCCACTCTTAGAGCCTATAATCAG
TACAAGAGTTTCATGATGAGTTTGATATTCAACTTACCCAGGTGACTCTCGTTGGGC
TACTGGGTGAGCTTGAGGAAGTGAAGAGGTTGGTTGGATATAGTAAAAGCCACACTT
CTAAGCTGCCTAAGAATGGTGAGG
>non-DHS494
AAGTGGGAAAGCACAGTTTTAAGGAGCTTTTGAAGTAAGGGGCTGGAAAAAGATCAT
GTGTTAACCTTTGGTATCAGTTACTGATGATATAATGAATTAATAAGAGACATAATT
TCTCCTGTTTCCCTTTTCAAATGTTACAGAACCAGGCTTTAGCAAAGATGGATATAT
TGGTGACTGAGACAGAAGAACTGGCAGAGAATATACTCAAGTGGCGTAAACAACAAA
AC
>non-DHS495
CTCAAGTGGCGTAAACAACAAAACGAAGTTTCGTCTTGTATCCCCAAAATATTAGCT
GAAGAAAGTTATCTTTATAAACATGATATTATAATGCCTCCTTTACCTTTTACTTCT
AAAGTTCATGTCCAAACTATTAATGCCAAGTAGTCATCAACTTTATTTTTGCTTAAT
TATGTGTAGTCATATGAAGTCTATTTCTAGTTGACTGTAACATGGGTATTAATAGTC
TTTGCTGCTGGTAATACTGAAAGA
>non-DHS496
TCTTTGCTGCTGGTAATACTGAAAGAACCTGCTTTATATTGGAGTATCAAGATCTCA
GGTTCATTAAGACCAAACTGACTTTTCCTTTGTTTTTCATATATTTTTATTCTACCT
TTCAGTAAAACTAGAGAAGCTAAAAAATAGCCATGACAATTTATTAATATAAGTGAA
TTAACCATTTCTCAGGTGGGAAATTCTCTTTTTTTAAAAATGTGTGTTTATCGTCTG
GTGTGGTG
>non-DHS497
ATAATTGGGGAGCAACATTAGGAGGCCACCATTACAGTTGAGATGAGAGGGTATAAG
CTAGAGATGGCAGAGCTAAATCGTTTTCTTTGGTCCAAAGCTGCCTATGCCCTTTTC
TAAGTGTTTTCCAACAGAATTCATGAGATCATTCCACAGGATCTCAGCAAGTGCAGT
TTCTGATAACTTGGCCGAATTTGTTCAGTAGCTGCTGCAACGGAGGGCTTAAATCTT

TCATCTACACTTAACAAGGGGAGC
>non-DHS498
ACCTCCTTGAAGGGACTTAACCTCACCTCTTTTTAAGTCCAGTTTCTCTTCTCTGTC
CTGGTCCTCATACCTGGGCTTTCTGTCTGGGCCACTCTTCTGGAGTCACTGTTCCGC
ATGTGTCCCCAGCATCCCTTCTTCATATCAGGGTCTGGTCTTGCTTTTGTATCTTTG
CTGGCTGCTCTGATCTGGGGTGACCAACCATTCTGGTTTGCCTGGGACCAATGGGGC
TTCCTTAGATAGAGG
>non-DHS499
ACTCAGTGCTAAAACCGAAAATCCAGGCAAACTGGACCGTGTTGGTCATCCTATCCT
GAACCTTTTGCTCTGTGCCTGAAGTCCCGCAGCTCCCTGGTAGCCCACCCTGGTGTC
TCTTCTGGCTCTGTTGCAGGTCTCAGCCCACCTATCAGAATTTGCAGCTGTTTAATC
TTAAACTCCCTGTTCCTGATTGATGGTTGGGACTGTGACTGGGAGTTGCTTAAATAG
CTCCCTCCCGTTTCT
>non-DHS500
TTAAATAGCTCCCTCCCGTTTCTCAGCTAAACTCAATTTCACCATCAGCCCCCGCAG
CTGGGGTAGATCTTGAAGTTCCCTTCCTGCCCCTCTGAGCAGACCCTGGTCCTGTCC
CATCTGCTCTGATGGGAGTAATCATAACACCAGGGTATCTTCCCTGCCTGACAGGGA
CAATGGGAGCTGAGTGGCTATCAGAAGAGGTCACGGTGAAGTGATGCTGACTGAGAG
GGT
>non-DHS501
GGTGAAGTGATGCTGACTGAGAGGGTGCTGATGGGGTGTTTTTGTGGGAAGGCAGAG
ACAGAGCATGTATCCAGGAGACAGAAGCACTCAGCATCCCAGTCCCTCCTTCCACTC
CTGCTCTGAGGTTGCAGTGAAGCAGGGTTTGGGGATTGGCACCCAGCATGATGACTG
TGTGTCTCTGGATGTACATGTGTCTGCTCTGATTAATGAGTAGCCTCTGACATCAAG
CACTCACTCCCATTA
>non-DHS502
GGCATCCAGTTGGAGATATAGGACATATTTTTAAAAGACAACTAATAAACACCACAT
CCTCCTTACCAACCAGGATGTTAAAAATTAGTCATCAGGGATGCAGGTCTTTGGAGG
TGGATTTCTTGCTAGGGCCAGGTGAGTCTGGAAGACTTTGTGAAAGTGGAAGGGTTT
GTTCTTGACCTTGGCAAATGTCTAGGACTTGGCTACATGGCGAAGAGCAAGTAGGGA
AGCATTCC
>non-DHS503
CTACATGGCGAAGAGCAAGTAGGGAAGCATTCCAGGAAGTGAGAGAAAAATGTATGG
TGAGTTTAAGAAAGAGAAAAAAGTCAGCTTTACAGAAGCAAAGCATTCACTCATTCT
TTTTGTATTAATTCAGTGCTCACAATGTGCAAAGCCCTGGTAAGATGCCAGGAGGTC
TTCATATACATATAAGGTGTGGCCCCTGTTCCAGAAGAGTTAAAATCTAGACATGCT
CAAAAGGAACCAGCAATAAAGG
>non-DHS504
CACTGAACAAACCTAAGTCACAATGGGCTTTTTGTTTAAGTATTCATTGTCAGGAAA
TCCAAATATTTTATAATTCACTGGACAGTAATGAACAATGACAGACAAAACACTAGT
GAGTAAAAACACTAATTATGTTACTGTGGTCTTTGTGTGGCTTTGGCACAAAAGGTT
GTTTATTTACAATGCTGCAACATCACCTTGGTAATCAAAAGAGTGGCATATGCG
>non-DHS505
GTGGCATAGAAGTGCAGCACACGGGGGTCACAGCGAACCGCCATGGGGTCAAAGTCC

AGCACACGTAGGCCCTGCAGGCTGCGGGCCCGAGAAAGGGCCACATAGGCCTGGCCA
CTGGCAAACACACGGCCCAGAGAAATCTCCACACAATCCAGGGTCATGCCCTGGGGG
ATGCAGGGACAGAGATGGAGTCAGCTCAGCCTTAAGTCCCTTGCCCCGAAAGGCCTG
ACCCTTTACCAGCCCTGCTACTG
>non-DHS506
CAGCTATCACAACTGATTGCTTGAGCAGATATTTGGTTGGAATGACAGCCCCCAAAC
CTGTAAGCAGATCTTATTTTTCCTTTTTTTGAGAGAAAGACAAAGAACAAATTAAGC
AGGTTTCAGAAACTTTTTTTTTTCTAAAAAGCAGCTTTCATCATTTCTAAGTAAGGC
ATAGACTGATAAGTCAACACATATTTGATTATCTGCTAGGTGTCACAGCGCTGGATG
TACACTGATTAAAGCTGTCATG
>non-DHS507
ACATAGTTGTGTGATGGCTGTTCATTCAACACGTCCTCCCAGCACCTGGCACAGTGC
CCTGCATTTACCTGGCACTCATTGTTTGCGGGATGAATTAAAAGAAAGCTCTAAAAA
ATACAAGATAATATGATGTTAAACGGACAATGCCTTTTTTTATGTGACAACCATAAT
TATCTACATATTAAGTATGACTTCTAGACATTTAAACAAAAAGCATAGTTTGGGGAC
AACATAAATTAGAAAAAATACTGTCGA

>non-DHS508
GACAGTACATCCCTAGGTCCCTGTCCCAGCGTCGGCTACCAGCCACTCTAAGAAAGA
GCAAGGTGCACGTGCCTGGCTGAAGGATTTCACTGGGAAGTGAAAGAGCCCGTGACT
GTATCTGACACTCAGTTCCTGCCCGGGTGAGTCAGCCACAAGGGGCCTGCAGGACCT
CCCTCCACAGCTTCCAGCCAGAAACAAAGAAGGATAGGGAAGCCCTGGTCAAGTTAA
TGGCAGCTAAAACGCTC
>non-DHS509
AGATGCTTCAACGGATCACAAAGGAAGCCCGTGGGCCGAGGCTGCAATCCACACAAG
CCAGCGTTCAACTCCGGACAGGGCCCAGGGTCCTTGCTGGAGGAGAAGGGGCATGGA
ATCTGGGCCCTATGGGCACTCAGCCGGGCGTGCGGCCTCTGGGGGCCCCCTGGACGC
CACAGCAGTATGGCAGAGCTGTGTGTGTTTTCACAGAGCCCTGTGACCAGTCTCCAT
CTCTCAG
>non-DHS510
CTTCTGGAGCTAATGGGTTCTGAGAGAACCCTGAGTGGTTATTCTCACAGATGTGAC
ACGGGGAAAAACTGAGGTGAAGCTGCTTCCCTCCACACGCACAAGTAGGCAGCGGAG
GGGCCCAAGAAAGAGCCAACAGGCGGCCCTCTCTCCCTGACAGCCACCATAGCCCAG
CCTGGCCCCCACCTCCGATAACTGCAGGGTACGCTGCTACAAATGACGTCTGCGTTT
GAC
>non-DHS511
GTTTGACAAGGATGAGCAGAACCGGAGCGTGTGCGTCAATTCAAATCTGTGACACTG
GCGCAAAATGTGAGAATGGGCTGGGGTGCCAGGCGGCCAGTCACGGCTAGGGCACAG
TTCCAAGAAGGGCCACAGGGGCCCGCAGGGACACTTACCCCAAGCCCTCCAGAAACA
GCTGCTGCAGGTCAGCACCAGCACCAGGGCCAGGCACACAGTCTCATCGGCACGTCT
GCCACTGGCTGTCTGCAAAGTGCTTCCCTTTC
>non-DHS512
CTCTCGGTTCCAGAATCCTTTGGCCTGACAGGCAACTACAGATACTTTCGTGGACTC
TGCTGAAACCTGTCTGAGACTATGGAGCTTTACGTGGCTAAGGAAGAGAAAAAATAT

GTTTGGAGTCCTTGCCCACTGACAGGTGCATAAAAATACAAGATGGACTGGGCACAG
TGGCTCACGCCTATAATTCCAGCACTTTGGGAGGCCAAGGTGGGCGGACCACTTAAG
GTCAAGATTTCGAGA
>non-DHS513
AAGATGGACAACGTTAAGGCTAGGAATTATAACCTTGCCGCTTAAAAATGAGCTGTG
CACAGGATAGGCGCTGCCCAGCTCTGTGCGGTTCACATGCAAGCTGCTGTGAGTGCA
TCTGCCCTCCCCCAGGGCTCCTGCTGAACCAAGCTGGCGGGTGTGCGTGTGGTTTGC
AAAGCTATCACCTTTAGTCTGAGACCCACCATTGCCCCAGCGCTTTAGAGAAGCTGC
ACCGAGAG
>non-DHS514
GGACCCAGGCCTGACAGAGTCCCCGGCAGCTACAGAAGTGCATGCTCCCTCTCTGCA
CGGGCCACTTCTGGTACCTCTTGGTGTTGAAGTAATTTAAGTCAGAACCCCTTTTAT
TGCCCCAGGGGTGACCGGGAGGCCGTGGTCTCGGGGCTTTGTGTTTGCAGGAGTGTG
GGGCTGGGGCTGCCTTTTCCGCGTCCCCTGGAGGAGACCTCAGGCAGCCTCTGTAG
>non-DHS515
TATAAACAGGGATGCAATGCACAGCCTTTTAGTACATTTTGAGATACCTGTTGGATT
ATTTCCATCATGTTCCTGGAATATAAATTCCTGGATTGAAGTGTGTGCAATCTGAAA
TTGTTGTGAATATTGCCAGGTTACCCTCCGTCTGCATGCCCACCATCAAGGTATGAG
GATGGTAGAAGCTCTCGTCGAACCAGATGGATGAAGACCACTAACGGCTTTTGT
>non-DHS516
AATAGCCCCTGGAGGTTTGAAGAGGCCCCGTCAGAAACCCAAACAGAGCTGCCAGAC
GGGAATGCCGCCCTCCCCCGTCCACACTCATGGCCGCTGCTCAGCCCCCAGCCGCAT
CCCTCCCCACGCGCGTCACGGCCCTGAGGCCATGGTGGGTGGATGCGGCTGCGGAAA
GGCTGGGGAGTCCGAGCCCTAGGACTGGGAGCAGCGAGGTAGAGAATGAAAGCTGCA
CCTGTC
>non-DHS517
GCGTGATTGTATCTGAAAGGGTAAAGGAGGAGGAAAGCTGAGACGCCTGCTTGGTAG
CAGAGTTGGGTGTGGGAGTGTCCACAGACACCCCTGTCCTGCAGGGTGGGGAGTGGG
CACCTGTGGCCCCAGGCAGGTTCCTTCCCACAGCTGCTGGGCTTCTGGGCCTGCCCT
GGTGCCTGGAATCACACATGACAGGGTGGGGAGGACAGGGGCAGTAATGCCATTTGC
CTGCCTGCATTCTCT
>non-DHS518
GAAACTGACTTTCAATCCCCAAAGAAATCCCAGTCCTAAGTTAACAAGAAGAAACAC
AGAAAACCCTACTCCCCTTTTAGTCTAATGAGACCAACCAAATGTCCTCCCTATGGA
ACTATGAAACAGGAAAGTTCCCACGATCTTGCAATCCAGGCTGTAGTGGATTATAAC
AGCTACTGTTTGGGACTGCCCAAAGCGTCTCCTTTTGGTGCACTTGATGCAGACATA
ACCAGT
>non-DHS519
GCACTTGATGCAGACATAACCAGTCATAGCATCTCATTACCCGGGGCAGAGGGATTT
ATATGCTTCAACCAAGACCAATCAGAGTCCTTCCCTTGGATAAATACGCGGCTGTTG
GAAAAAGCAACTGCAGTAGAAAAAGGGCTTTGAAGATGAAAAGTTGGGCGGCACCAG
CGAGGGCCCAGGCGGGAGATGCTGATGAGGCTGGGAGGACGCTGCTGAGCCCCTGTC
AGCCTGACAGCCAGCACATGAT
>non-DHS520

AGCACATGATGGCAATAGAGGAGCTCAAAGTGTGCCTTCTCGGGGACACTGGCGTAG
GGAAATCAAGCATTGTGTATCGATTTGCCCAGGATCACTTTGACCGCAACATAAGCC
CTACCATTGAGGTATCTTTTGTGACCAAAACTGCGCCTTCTGGAAATGACCTTCACA
AGTTCCTCATCTGGGACACTGCTGGCCAGGAACGGTTTCATTCGTTGGCTCATGTGC
TAT
>non-DHS521
ATTCGTTGGCTCATGTGCTATGGAGGATCGGCTGCAGCTGATACAGTGCATGATGTT
ACCCAAACAGGATTCATTTCATACAGATGTTACCCAAACAGGATTCATTTCATGCCT
TGAAGAAGTGGGATAAAGAGCTGAAAGAACATGGTCCAGAAAACATTGTAATGACTA
TTACTGAAACAAGTGCCATCTCTCAGATATTAGGGAGTTTCCCCTGAAGGATGCTGA
GGAATATGCTGAATCC
>non-DHS522
TGCTGAGTTTGAGGGACCTTGGGCTGAGGCTGCACCAACTTTTTTCTTTTAAGAATT
TACAGTGCTGCTGTAACTGCGTCAACCACAGTTTCTTTGACATTCTCTCTAAATGGG
TTTGGGGAATGCTGAAACTCCCTGTTCCTCTTCTGCTATAATATCATGGCTGGGGAA
CAGGACTTTTATTCCAGCCTTCCACCTGTTTACCACCAACAGCTGTTGTCCCCAGTA
TGCTGCTGCTGCTCCTGT
>non-DHS523
CTGGCTCCTGTGTTCCCTCTATCCAGCCAATGCCTGTTTACACAGACACCTGGCCTC
ACTGCCAGCCCACCTCCCCTACAGCCCTGTTTGTTCCTGCTTCTCATGATTTTCCTG
TAAATAAACACACTCTTAATTTGCCATTTGTGCCTTGCGCTTCACAAGCTCCAGCAG
CAGCCTTTCTCTTCTTCTGTTATCTATAGCCTGGGACCACCCCCTTCCTCCCCTTGG
CCTGTCGTTTGCTTCCTGTCCTTCT
>non-DHS524
CATCTAGGGATGCTATGCCCTGTGGGCCCTATGCCCGGGGCACGGAGCTAGGGGAGG
CGGTCACCCTACTTCTCTCACGTCCCTGTCCACCTTGCGCCTCAGCGGAGCCTTAGG
TAAACAAGCCAGGCTCCCAGTCCCCAAGGACGGCCTTCACCCCAGACCCCAGGAGGA
GGCGTCTTGGGCCATCTCGGGAAGGCTAGCTTGCTCGGGAGGGGCAGATCACGAAAA
CGGTTCTTTAGG
>non-DHS525
GCCTCAACTGCTCTCCCTGTGCAGGCAGTCGGCAGGCGGCTCGGGGTCTAGGGGTCC
AGGGCCGGCCGGCCCAGTCGCTTGTGTGAGCAGAGTACCAGCTGCTTCTGGAAGTTG
GGGGCCTCCACATCATGAATCCCAGTCAAGGCTCACAGGCCAGCCCCTTGGAGGGGT
GGGCCGGGCAGAGAGCCCAGCAGGATGCTCGCCCTCAGCAGCCCCTTCCTGGCTCTG
GACTCTG
>non-DHS526
TACAGCCTCTCATATCCATGCTTCGAGTCTTTCAAATGGAGCAGACCGAGGTTTCCC
TATCTGGAACTCTATGGATCATTTTCTTCTACTATCTCCCTGCCCCCTTTTCATCTT
TAATGAAAGATCAAAGAAACCCCAAATTCTACCAATCCCCAAAATTATTGTTTCAAG
TTTTTCTTAATGTTATATATCTGATACATTTCTAAGGAGTCTGCATCCTCTACTCAA
CATTAAAGGACACGAGAAGGA
>non-DHS527
GAGGAGCGTAGGAACCTGGTCCGCAGCCTCACCCAGCCCCCGGCAGGCCGGACCTGA
GCTCCCCCGCGCTCGCGACGAGGAGGATCCCGAGCCGCCGGTGGCTGCCCCTGACGC

CCTCTAGGCCAGCAGGACCCGCCCGTTCCCGACCTTCCCTCGAGACCCGCCAAGAAA
TAAAGGCGATGATTTCCGACCATGCTCGCGTTCTCCGCGGAGTCTGTGCTACACCGT
GGAG
>non-DHS528
GTTCTTGGTGGTCTGCTCCTTCCAGCTGAATTCACCGGAGTGCGATAGGCCCTCAGC
ACCATGCTTGGCACATGGGAAATGCTCACTAAATGCCAGCTGTGGCATCATAGAGAG
GTCTCAGCATAGTCGCCTTTATCTCTATGCTCAGTAGGTAGAGAAGAAGCGAGGAGA
GAAGAGTCTGAGGGGTGGGTGACTCCACCTGCCGTCTCCCTCCGTGGGTGGGTGTGA
TGGAGTCATGGTTCC
>non-DHS529
TGCAAAAGGGGAATGTAATCCTGGGGTGGGGGGAGACCCCTCATCTGTAGCCCCTCC
CTTGCTCCTCCCAAAGGGTGGAATTAGAACAGGGACTGTTATTGGGAGACAGAAAGT
GGGGGATAGTAGTTGACCTTTGGTAAGGGGGCAGGTGCCCAGGGCCAGAGGCTTCTG
CTTCAGGCTGTAGTGGGCACTTGGCTGCCAGCCCAGTGTGAAGGGGGGAGGATGGAG
AGAAAGAGAGGCGGGGCT
>non-DHS530
CAAGACAGCTGTGTGCTTCTTTGTGGGAGCTAGGGGATGCTGGTTTCTGGGCTGAGC
TGGGGAAAACAGGAGGTGGCGAGATGGGGGCGGGGAGGGGAGGCAAATAGATATGTA
ACCTGCTAGGACAAAGAGGAGGTGAGGGACTCACAGAGGAAGCTGGGTGGTTCTAGA
ACAACAGAGGTGTTCACATTTCCAAAGGAGGTAGGGGAAGCAGATGCTATATGGTAA
AATGAAGCTGCAGAGTTG
>non-DHS531
CTTGGCTAGAGGGATCATCATAGGAGTTGCATTGTTGGGAGACCTGGGTGTAGATGA
TGGGGATGTTAGGACCATCCGAACTCAAAGTTGAACGCCTAGGCAGAGGAGTGGAGC
TTTGGGGAACCTTGAGCCGGCCTAAAGCGTACTTCTTTGCACATCCACCCGGTGCTG
GGCGTAGGGAATCCCTGAAATAAAAGATGCACAAAGCATTGAGGTCTGAGACTTTTG
GATCTCGAAACATTGAGAACTC
>non-DHS532
GTCCATGTGAATGTTGCAAACGCTTTCTTATAGGGACCAGTATTAAATCTTTGTGGT
TTTACCCTGAGTCAGAACACTAAGAGCCTGGTTTATTTACTGACCACTTGCTTTGAC
GCTCGTGAGAAGGCTGTTCTGTTGAAGTCGTAATGCTTTGCACCCAGTAGGAGCTCA
ACAAATGCCTAATGAGCTGAACTCAATTGAGAAGGTCAAGGGGCTAAGCAAACATG
>non-DHS533
AGAACAGACTCCAACAACGTCCATCCCAGCAACAGGTGGGGGTCTTGGGAGGGTTAT
GTCTAAAGGGCTGTGTTCACCCCACAGATGACTCTCAGGGGCCTGCCATGGCCCCTC
CCTCCTTGGTTTTTTCTCCCTTTTGTCTGCTGCCCCCATCCCACAGGCCCTGGAGGG
CAGCAGGAAGGAGAAGGGCAGGGGTTGTGGCATGGCTGAGTGCACCCTCATTTCT
>non-DHS534
CTACACGCTGTGAATGTTGAAATGTTCATCAGCTTGTTTTCTCTTCTGCAGCTTTGT
GGGGGATGCAGTAGCCTCTCAGAGATGTGATATCCTCTGTTCTGACAATTTGTCCAA
GGCCCTGAAAGTTCCCGATGTGTAGTCATTTGATTGAGGAGGGGGAGGAGCTGGAAT
CTCAGCATGCAAGGCCACAGGCAGCAGAGAAGCCAGTGGTCACCATCTGCCATTGGT
CTTGGTGTGGCTTTG
>non-DHS535

GACTCTCCTTCCTTCTCCAGCTCCTCCAGCACCACCCTCAGCAGCCTCCAAGAACAG
AAGGAGTTAACGCTGGGATTAACTTCTTGAAGGAACCTGAAGTTTTTTAATCCTTGT
TCTTTCCTCCTTCCTTCTCCCTCTCCCTTCCTGATCTTTACTCCGCCTGTGGGTCCC
TCCTCCTTACTCTCACCTCCCCAACTCCAAAAACACTCTGATTGCCAGACTGGGTCA
ATGGTTAACACTCAACTGGCTT
>non-DHS536
TGAAAGGCACGTGTGTACATAATCTCTTGCGTGTCTGTAAGGAAGGGGTGTATGCTC
AGTTTCCTATGTGCTGGAATAAAAGGTGTGTGCATGTGTGTGTGCGCATATGTGTGC
GCCTGCATGGATGTGAGGGGTGTGTGACGTGAGGCTATCTGAGGGGGGCTGTGTGCA
TGCACATGATCCTAGGTATGTATGTTGGACAGTGCACACGTGTGTGTTCACAGACAA
TACAACATGCCCTCTCTG
>non-DHS537
ACCCACTCAGCCATTCTCTACCCATCCTTAGTACATGCTCTGTCCAGCTTTCCCCAG
GGTGACATACAGAAGGGGCAACATCTACCCGGGCACCAAACTGAGCCCAATTCCGAA
GCAGTGTTTGGAAAAGCCCTTGCCCTCGGAAACTTGAACATGTCCTTTTTCCCCAGT
CCCGGGTGCAGGAAGGGCCCCCTCCAGGTCACCACAATCTCACTCCTCCTCCAGAAA
GAA
>non-DHS538
CCTTAGTCGGGCGAATATTAAAAGTAGAAGAAGGGGCCGGACACAGTGGCTCATGCC
TGTAATCCCAGCACTTTGGGAGGCTGAGGTGGGTGGATCATGAGGTCAGGAGATCGA
GACCATCCTGGCTAACAAGGTGAAACCCCGTCTCTACTAAAAATACAAAAAATTAGC
CGGGCGTGGTGGCGGCGCCTGTAGTCCCAGCTACTCGGGAGGCTGAGGCAGGAGAAT
GGCGTGAACCTGGGAGGCGGAGCTTGGAGTAAGCCGAGATTG
>non-DHS539
AGCTTGGAGTAAGCCGAGATTGCCACTGCACTCCAGCCTGGGCGACAGAGTGAGACT
CCGTCTCAAAAAAAAAAAAAAAAAAAAAACCAAACGAGTGCACGAGGCTGATATGCTC
ATTTTACCACAGCAGGGCTGAGGCCCTCCACTCCCATTGTTTGGTGCAAAACAACTC
TATTTGGCTTTATCTACACTAAGGTTATTTTGTTTGGTGGTTACAAAATGAATGACA
GCAGG
>non-DHS540
GGTGGTTACAAAATGAATGACAGCAGGTCCTCTTCAAGAAGTCAGACTTGATCATCC
CATCTTACTTCCCTGGGTTTTTCAAATCATTGAATTCTGTCAATAGTTGGTTTTCTG
CCAACTATTGAATAAGATCATGATGGCTTTATGACACTCTCTCACATGGCTTTCAAG
GCACAGCGTGTTCTGGTCTTCCTACTCTTTTACCATTCCTTTTGTCTCCTCTACCCT
CTTCTTCCATTCGCAGGACTTCTCTAT

>non-DHS541
CCTCTACCCTCTTCTTCCATTCGCAGGACTTCTCTATATATTTTTTAGATCTATGGC
AAAATGAAACTTTATTTTGTAAATTGATACTCAGGTAATTCCATATGCTGATAACTC
CCAAATCTATACCTCTAGCCCATTTTAATTCCAAACCTTATATTCAGCTGCCTCTTA
GATATTTGCCCTTGGATAAGAGACAGCTGGGTTCATGGCCACCCATCACCCAAGCCA
GAAGCCTGAGGGCCCTCCATGATGTCACCTCCTTCC
>non-DHS542
ATGCTGCATGTGGTGACAAGTTGGGACCTGGGCCCAGGCCGCGGGAACTCTTGCCTC

AGGCCTGGGTTAGCTGTAGGCTGGGCTGTCTTGGTTCCTGAGCCAGGTTCCAGCATT
TCCTGGTGGGAGAGTTTGGGACTGACACAGTGTGTCTCCCAGAAGAGCGTGCCTTTT
CTCTCCCTCTGTCCCAGGCAGAAGGGCAGATGCGGGCAACATCACCGAGGCTGTCCA
GCTGCCTCACCAGGCTTCCGTGGTTTC

>non-DHS543
ATGTCACCCCAGTATGATCTGGTAGGTGGGGGAGGGGAAGGGAGGTGGAGAAGTGAA
CACGGGCCCTGAAACTTCAAACTTGGTCAGCCCAGGTCAGCTACCTCATCGGAGCTG
CTAACAGTAAGAGCCTCTTTCTCTTGGTTACCATGACCACCAGCATCCCCTCACATT
AGAGGGGAAGATTTTGTTTAACAAGATTCAGTGGTTCATTTTAAATCAACTTCAACT
ACAGAGGGCACTGTCTGAT
>non-DHS544
TTATGAAGGAGTGGGCTAGACTGGCGATGCCCGGGTGCCCCTGGCAACACCCCCTGG
CATAGCCTGAGGGGGTGGGGTTGGGCCGCAGCCCAGCTATGGGGAGAGCTCCCCTCA
CCCCACTGAGGTCACTGTGCCCAGAGGCAGAGCTTCCTGGGCACGCCAACTCCTGCA
TGAGGCAGGTGGTACCTGGAGCCAGACCATGGGTCTGGGGCCCTGACCGCTGAAGCA
AGAGG
>non-DHS545
AGATGTTGTTTTGCTGTGTTGGCCAAGCTGATCTCGAACTTTTGGCCTCAAGTGATC
TGCCTACCTCAGCCTCCCAAAGTGCTGGGATTACAGGCATGAGCCACCATGCCTGGC
AGTGTGGCACTTCTTACGTGTGTTCAGCGGACACTGTTTATCTTCTGTCCCTCCAAG
ACGGTGCTGAGCTCAGGTCGTTCATTACTGGCAGACAACTGCTGATTTCCAACAGAA
>non-DHS546
CAACAGAATTGCCATCCTCTTCTCCCCTGCGACTTTCAGAGTGTGACCTCAGACTCA
AAAATTAGAAGTGAAAACATCTTAAAAACTATCACCTTTTCTTCCTAATCCTCCTCT
CCCCTCCCTGTCTTCCTTGTTGTCCCCATCTAATGAACTATCATGGCAAAAAGAGCC
CATTTCTGGTCATTTTCTGTGGCCTTTCAAACTCCCACCTACCCCACTGCTCCTGGG
TGCATTACCCGAAAGCTGAGACTTC
>non-DHS547
AAGCTGAGACTTCAGTGCAGAAAGTGCCAGGCCCTCTGTCCCCCCAGATCGCCTTCC
TTGTCTTCCCTGTGCTTGCCTGTCACATTGTGTGGGTTCCAGCGCTGGAAGGAATGA
GGAACAGATTCTCTGGTTCTCCTTTTGAAGTTTACCTTCGCTCCACCACTTCTGAGA
CCTTCCCGGAAGTTGCCCCTTGTTTCTCTCCTCTCCAGGGCTGCCCCAGAGCTGCCT
CTCACCTCTTCCTGCTGTCAC
>non-DHS548
AAGTTGGGACAAAGCCTCTCCTACTGGCTCCTGCTTTTCTCCCTTAGGTCCAGCCTC
CTCTTCTCCATCTTCAGGAGTCTCCTTCTCCACTCACACGTCATGACTTCAGCACCT
CGCATCAGTCCAGAATATGACTGCTTGTTCAAGTGCCACCTTTCTCATGCATTTTTT
TCTAGTGACAATCACAGCCACCCTGTGGGGCAGGAGTGTCATCATCCCCATGTTTC
>non-DHS549
GATATTGTTTGTGGTGGTTGTGTGTGGGTGTGTGTTTTTTCTTTTTCTTTCTTTTTA
TTTTTTTTGAATGGGGGAGGCTATTTATTGTACTGAGAGTGGTGTCTGGATATATTC
CTTTTGTCTTCATCACTTTCTGAAAATAAACATAAAACTGTTGAATGTGCCTGCCTC
AGTGCCAGCATGGGGGGACATGGATGGGGACTCAGTTGGGGTTGTACCCAAGCTGGT

GTACCCAAGGTGTTCTGTCAGCTTTCATT
>non-DHS550
GTCTCCTGCTCGGGTCAGTGCGCCGCGCTGCGCTGGGCGCCATGGCGCTCCCCGGAG
CCCGGGCTCGCGGCTGGGCGGCAGCAGCCAGAGCGGCCCAGAGGCGCCGCCGCGTGG
AGAACGCAGGAGGGTCCCCGAGTCCTGAGCCTGCGGGCCGGCGCGCGGCGCTTTACG
TACACGTGAGTAGGGGCGGGGGCGGAGCCCACGGTGTGTGGCCGCAGCCCTGGCCGT
GACCGTGGCCGTCCAAAACCCAGGTGGCGGTTTGTCACTCTAT
>non-DHS551
TAATGGTGACAATCCCTTTCTGTCTAACTATATGTACTTAAAATACTTTCAATCTCT
CTTACTAGATACTTTATTTATTTATTTATTTATTTATTTATTTATTTATTTTTATGA
GACGGGATCTGGCTCTGTCACCCAGGCTGGAGTGCAGTGGTGCAACCCAGGCTCACT
GCAACCTCTGCCTCCCGGGTTCAAGCCATCCTCACACCTCAGCCTCCCAAGCAGCTG
AACTACTGGCATACTCCACCATG
>non-DHS552
CAAGCAGCTGAACTACTGGCATACTCCACCATGCCTGGCTAATTTTGATATTTTTCG
CCATGTTGCCCAGGCTGGTCTCGAACTCCTGAGCTCAAGCGATCCACTTGCCTTGGC
CTCCCAAAGTGTTGGGATTACAGGCGTGAGCCACTGCAACCGGCCTAGATAGACATT
TAGATAGAACTTGTGTTTTCCTTCAAAAACACTTTACAATGTCAAGGACATAACTGG
CAATCAA
>non-DHS553
CTTCATCATAATTTGGAGGGAAGCTCTTGGAGCTGTGAGTTCTCCCTGTACAGTGTT
ACCATCCCCGACCATCTGATTAAAATGCTTCCTCCCAGCATAGGATTCATTGAGTTG
GTTACTTCATATTGTTGCATTGCTTTTTTTTCCTTCTTTTCATGTACACTGAATAAC
CTGACCTAATAGAGAGTGTAAATACTATAAAAATGATTTATTTTGAGGTCTCACATA
CACACAGGCAT
>non-DHS554
CAGTCAGACCTGAGTGAGAAATCCATTGTTCCCTGATTTCCACAGGCCTTTGCTGAG
AATAGTGGACCAAGCCCCATGTTAGGCCTCTCGGAATGGGGGTCCCTGCAGAAAAGG
ATCCAGAATTTCCCCCAAAGCCTGACTATTCCCTCCTTCAGGTAGACACATCCTGGC
CAGTGTCTTTAGGTCCCTCTGGGAGAGCTGGTAGAAGATTCTCAGTGTGAACTGTGG
TAGCATAACCAGG
>non-DHS555
ATCCAAAACCCTTACCACAGCAGTGCCCTGCTCAGCTGACCTGCCCCCACCCCACCC
TTGCTTCTACCCTTTGGCCCCTGACAGATTCCCCTCCACAGGGGTCGTGGAGGGCCC
CTCCCTATCTCACCTGCTGGGTGGGGAAGCCCTGGGGGACCTTTGACTCTTCATTAA
CCTGAAGACTGGTGGAAGGAATGGGTGGCCCCAAGGGAACTCTCATTGCTGAGGTAC
ACATGGTACGAAGGGAG
>non-DHS556
GCTGGAATAGCCCTACCTGAGTGCCCTGTTTGACTCCGCCACTATCTGCCATGTGAG
TTGGGCAAATTGTTGACCACCTCTGAGCCTTGAAAAAGTAGGAGGTTACTTTGTTAG
AGCAAAATAATAAAATTTAATTTTAAAAAAGAAAACGTAGGAGGTTGGTTTGGATGA
GCTAGTCTCTTCCGGTTGAAAAGTCTGACTCGGTGAGCATCCTGACGACTCCATTTC
CTTCT
>non-DHS557

CCACTCAGTTCTTCCTCCACCTCCACCCAGTGATCCCAATAAACGAATTCTGTCTCC
CCGTGCCATGTGGTGTCCCATTCATTCACATTTGAGAGTGCAGGACCCATGTGTCTG
GGGGATGAGTGCAAGGACAGCGTTGCTCAAGTCCTTGCCTGGGCGGACGTGCTCAAT
TTGCCTGCCTGGGAAACAGGCATCCTCGGAGGGCTGTGCCTCCTTGAGTGCGGGGCA
GGGATGCAGTGGCAGGTCATGGAAAG
>non-DHS558
CTAAATCAGAGCTGGCAGCCTTCAATTAGAAGGGGGTGCAGCATTCTCATCCGAGAG
CTCTGACTCCCAAGCCCAGGCTGGGGGGCCCGATGGCTCACCCAAGGCAGTGTTTTC
TGTCCAGAAGTTTCCACCCACAAATATGCCAAACCCTTCCTCTCGCCTGCCTTGCTT
CACTGGGACAATTTGCTATCGAAAGGCACAATTATCAGGGGACATATGATGTTTAGG
A
>non-DHS559
CAAAGCCACAAGAGAGAAAGGACTTAACCTCTGGCGACTTTGCAGATCAGAACCCCT
CCCGGCCCCCGAGGTACATGGGGAAGAAACACAGTCTCTGTAACAGTGGCTGCACCT
GGAGCCTCGGGTACAGACACAACAAGGATGTCGCGTGCAGATGAGATATGGGGGTTC
GCTTTCATTATTCTGCTGGTAAGGTTAACAAGTACCAACGACCTTTGTTTCGCTTCT
CTGTACA
>non-DHS560
GGCGCTCCTTTATCTGATGTGGTTTAGTAAAAGCCTCCCACACACCTCCCCGTGGCC
TGGGACGTGGCTGGTAGTGTTTGTGTGGGAAGCAGCTCAAGACGCGGACCGCGTGAC
ATTAAGGAGAGCTCAAGGGCGGACCGGGCCCTGTGTAGAGACCCTTCCGCGCCGGCG
TTCCCGCTCCGCTAAAGATTTCAAATCCAGGGGATTCCATGCTCAATGGATGAA
>non-DHS561
AGTTGTGCTCTTCCTGACTAGCTGTTTTTCTGCAATCATAATCAACATCACCACCTT
GATAAAGCTAGCAATAAATTGGTAGCAGTTCTGAAAGCACTAAACAAAAGATCTGTT
AAAGGCAAGCAGTGAAACAGTGTTGTTAACTACAGAGCCTCTATTTGGCCATTGGAA
ATTATTGAAATTGGTATACCACTCTAAAAATATGATCCTTAGAAAAGAGTTTTGTTG
GTCCTCATCAACC
>non-DHS562
GAGTTTTGTTGGTCCTCATCAACCTGCTGTCATCTAAATATGACATTTGTACCTTGA
TAGGGTTGAGTCTGCATGTTGAAACTTAACACCTCACTGAACAGGGAGCCAAATCAA
AATGTTAAGTCTTATTACATAAAGTAGACAGCTTTTTAGCATTCCTTTGATGGTTGA
CTGAAAGAATAGTGGACAGTCTATGCTTGCTTCTTTCAAAGTTCAGTTTGTTCTTCT
ATAATGAGACACATGCATTCTCCAGAATCACTC
>non-DHS563
TGCTTCTGAAGTTTTAAGGTTCTCCAGCTAACAGTAAATGCACAATTGTTACCACAA
TTGAATGGGAACAAAGGTAAGCTTTTAAAGAATGAATCTTTTGAGTACAAATTCTAG
AAATAGTCTCCACTGGTTATCTGGCACCTTTGTTACCATTTCTCCACAAACAACACT
CAACCAAAAGGAATCTTCTGAGAGGGAGTATAGTATAATCAGAAGAACACGGACTGG
G
>non-DHS564
AACATGGGCCTTAATCCTTGTTAGGGCAAATAATCATTTTGGAGAATCATGGGGAAG
GTGGATGGAGTGGTAGGAGGGAAGCCTGGCTGCCTGCGCTGTTAGGATTATCATCTG
TGGCTATTCCTTGGCTTTTATCTCTTTCTCATCATGTCTAGAGCCAGGCCCCAGATA

CTATAAGCCCAGGGTGTAAGAGTTCATAGGGATAGGAGAGGGCACACACCCACTCTT
CGAGTGTCCTCTCTCATCC
>non-DHS565
CTCTTCGAGTGTCCTCTCTCATCCCGCCCTTGACTCTTAGTCACTGACTAATCTTTT
ACTTAAAACAACTAAGCTGTGATGTTGTTTTAACCCTGCCTAAAATCCAAGGAGTCC
ACAGGCTGCCAGGCAACCTTAATTATTCTTTAGTTTACTCATCAGTAGTCCCTGAAC
ATAAAGTACACTTACACGCATAGTCAGCAAGGACTTTCTCAATGGGTTATTTCACTA
CTTGAAGCTCTCTGGTGACATTTGGAAGTA
>non-DHS566
GAATTGAGTGTCTCACCTTATTCTGCCCCAATTTTTTTCAGGATGGTTGAGTTCTGA
GTAAAAATGGTCCTGAAATACTACGGCCGCTACAGCTTAGCATATAGCTTCTTTAGC
TGGAACTGCCCACACCCCAAGTCTAACTTCAGCTGGCACCTGACATACTTTATGTAA
CATTGTGACAACAGAAATAACTTGGCACCCCTCTTAGGATGAAAAGGAATCATGTAG
GTCAGAGGCTGTC
>non-DHS567
TACTCCCATTTGTCTTTTCCACCTCACTATGTAGGAAGCCTTTTGGATCTCTACTTT
TAGTATTTAATTAATAGATATGAGGATAATCTCTCTAGGGGTTCCAGTAGTTCCATC
TCAAATATTTTCTTCCCTAGAGAGATTATCCTCATACTTATTGTATGGTGACTAGTT
GGAAAGGCATTTGGCCATATTACCACCAATAGCAGAGTTCTCTAGCATAAATTCTGT
AAACCAACCTGATCGGA
>non-DHS568
AAGCTGAAAGGCTACCACTACAGGTACATGTGATATGCAAGTGTATGCTATAGCTAT
TCTCAGGCAGTTTTCATGTTCATGTGCATATTTTTCTCTTTCATAGTTTACTTTTTA
TATGCCTGTTGTAGTGAATTTAGGCTTCTTAGACCTGAAATGAACAGAAGAAAGGAA
GTATCACTGAGCTAAGGGAAGGGAGAAGAGGTAACCAGAAATATATATATGGCAATT
ATTTAGGTTACTGCCTCAAAATTGGAAC
>non-DHS569
GAGGCTCTCCACCCCTCAACATGTTAAGGATACATCATTATGGAAATGGGAAGGAAT
TTCATGGTGAGAGTCAAAGTGCAGGGGGCTTGCCAGGAGAATGTATTTTTGGACATG
CTGGGGGACAAGAACGTTAGAAAAATGGCATCTAATGAACTGGAGTGCAGTGGTGCG
ATCACAGCTCACTGCAGCCTCAACCTCCTGGTCTGGTGCAAGCCTCCCGCCTCGGCC
TGCTGGTGGTCTTGATGGTGGGGAAGGAAAAC
>non-DHS570
GGGAAGGAAAACTATATGCGATTGAGGGGAGGATGAAAAGAACACCCATCACAGGAC
TATTCACAAATGGGAGACAGATGCAACCCCGACACCTGGGTCTTCAAGTCGTTCTGC
CCCAGCATCATTCCAGAGTCTAGCCCCGCCCTCCTGGTGCTGTCTTGTCCCAAGGCC
CTGCTGCCTGGCCTTTGGGAACACTGTAAGGTGCCACTGCTCGCCCTTGCCGCGGTC
CTCTGCCTGACCTGTTCC
>non-DHS571
GGTCCTCTGCCTGACCTGTTCCCCAGCATGAACCTCCCCTACCATATGCCCAGTTTG
GAATTCCATGAGGGGGAATGTGGGAGGGAACTTAGAAGAAACTGTTTTAAATAACAA
GCTAACACTCTCGAGGACAGTCTAAAAAGGCTTTCTTTAATGATTAAGCCTGGGAGA
GTTAAGACTGAAGGAAGGACTGGAAGAAGGAAAATGAGGATTTGGATGAGACTACAG
GTGA

>non-DHS572
GGAAAATGAGGATTTGGATGAGACTACAGGTGAATTTCCCGCAGATGGAAGCGCTGG
AGAGGGACGCCGAGCAGCTCTGCAGCGCCCATCGGCAGGATCACCAGGCAGGCTGGG
CCTCCATCCATGGGTGCCTCTGGCTCCCTATCTGTGCAACCGGACGGCCATCACCCC
TCACAGAGTTGTTATGAGGCTCCAAGGAATCCCTGTGTATGAATCTCAGCACAGTGC
CCGACTTGTAGTAAGTG
>non-DHS573
GTCAGCACACTGGCATAGAGGCAGCGCCGTGCTGTGGGGATGATCTGGCCCCCTCCG
CGGTGGATGGCGTCGGCGTGCAGGGTGACGTCGTGGACGTCGAAGCGCACACCCCGC
ATGTTCTCCTCACACAGTGCGCCCTGGGGGAGGGGGAGAGCCACCGTCAAGGGCCGG
ACACACCTCGGCTGCTTGCCCTCCACCTGCCAAGTCCTGCAGGTCTCCACCAGGGGG
ACCTGGGGCCTTGCCCGCCTTGGCCCCATTAGGGTCTCT
>non-DHS574
AGAGTGACGAGAGGCTGCTACAAGCCGTGGAAAACAACGATGCACCTCGGGTGGCCG
CCCTCATCGCCCGCAAGGGGCTGGTGCCCACGAAGCTAGACCCCGAGGGCAAGTCCG
CGTGAGTGCCCGCGACCCGGGAGTGAGATGGCTGAGGGGTGGCAACCTTGCGGCTGA
ACCCTTGTCTCTCACCTCCAGGTTCCACCTGGCGGCCATGCGGGGTGCGGCCAGCTG
TCTGGAGGTGATGATAGCTCAT
>non-DHS575
GGGCCCTTGCTATAGAATTAAGGTGTCTCCCCCACAAAAAATCATATGTTGAAACCT
AGTCCCCACTATGATGGTAACAGGAGATGGAGTGTTTGGGAAGTGACTAGGTCATGA
GAGCAGAGACCTCATGAACGGGATTAGTGTCATAAAGGATCTCCCAGAAAGCTCCCT
TGCCCTTTCTGCCACGTGAGGACACAGGGAGAAGATGCCTTCCACGAACCAGAAGAT
AGCCCTCACCAGACAC
>non-DHS576
CTAGTAGGGTCTGGGGTGGGTTAGCTGTAATCTCAGCTCTGTAAGCGGGCCCTGCCC
TCTGGCTTTGTCGTAAACAGCCACAGCAGCATCTCATTGCAAAGGGAGGGGCCGGGA
ACTTGTCCCTCTCTGCAAGGGAGGTTCTGACAGTGCACACATTTATCCTGACTGCTT
TGCTAGGCAGGAGGCCAGGCCCTAGAAAGCAGCACGGGGCCAGGCCCTAGAAAGCAC
ATCCCCATG
>non-DHS577
CTTCTTAAGTGCTCCTGACCTTGGGGGTTGAAGGGATGGCCTTCTAGTTCTAAGAAC
TTGCCCTTCTGCACCTCTAGAATAAATCCCTGGGACCTGCATCCTAACCAACCAGGA
GAACCCCTGACCCAGAGGGAGCATGCTGGAGTCAAAGTGGGCAAAGGGTTGTTACTT
ACAAAGAGGCGACTGGGGGTGAGGGCATTGCCCCAGTTGGCAGGGCTGCTTCTCCCA
GCCCTCTGCCCTCTGTTTTAGAGAC
>non-DHS578
CTCTGAGGAGACCTCACCCACTCCTCGCTCAGTTTGACCACTGTAAGTGCCTGCACT
CTGTATTCTATTAATAAACTAAAATAAAGGGAAGACGCTGCTGGTGGCTGCTGTGTG
GGCCTCTTGTGTTAGTGTTCAGGCTGGGGACATGGGTGCCACCTCCATCAGGCCTCC
CATAAGGATGCCCAAGTCCCTGCACTCATAAGTGCCTCCCATCTCCCTGAGGGCTCT
GCT
>non-DHS579
GCCGGGTTTTATAATCACATCCATGGATAAGGCAACTTGGTCATGGATGGGGTAAGA

GATGGAGGCAAGGAACAGCAGCTCAGGGGAGCCCAGCCACTCTAACCAGGAGAACAT
AACACACCAGTGGCACTGGTTCAGCCTTGGCAACTATTCTTTTATTGAACATCAGTA
ACAGAAAGATGCTTGATTTAGGAGGAAGCTTTCCCTCCCTCCCTCCCCCATTGCACC
CCGGAACTCAGCAAAGGTGAGCCAG
>non-DHS580
CCTGAGGAGGAAGTGGATCATTAGTGAGGATGATGCAGTTTCTCCGAGGGCTGTCCC
GGGAGCCCTCCCCTTGGGTCATGTGAGCCCTGGACTTCCCTGGAGCAGGACACGGGA
TTGGGGTGGGGGTGGGAGGATCTGGGCACACGTGTGCAGCAGCCTCGGCCCACACAG
CCTCCGGGTGGACCTGCAGGGGCCTGTTTGTGCTGTAGGCTTGACACGTCCAGGTAT
CTCTGTGTGTCTGTG
>non-DHS581
CTGGTTTCTGGGACGACTGAAACCACGCAGAGCCACGGGTAACCAAGGTACCCGCAG
CCCACGGCGCTTCCTCGGGTCCCAAAGGGCGCCAATGTGCAGGCACCGCCGCCAGGG
AGCGCTCCCTGCCGGAGCCCCGGAGACCAAGGTTTGGCTCCCTCGCCTGGTTTCCGA
AGGAGTGAGGGTCCGACGGCGGCCACGAACAAAGCTGCACGCTCTGCAGACCCCGGA
GATCCCCTAGAAATTCCGCAG
>non-DHS582
TTTCCAGAGAGAGAGAAAGATGTTGTAATAGATGTAAATTCTTTCTTGGGAGACAAA
GCTTTAACATGCTCCAAGACTAATTATCTGACCAATCTCTCCCCAGATAAAGAAAGA
TTGTCACTAGGCAAATGTCCAAAAAGCTTCTCAGCCGAAAGCTGGATTATTCAACAG
TCCCTGAAACAAATATACACAGAGGACAAGTGATTTTTTTAAAAAGGGAAACAAGCC
TGGC
>non-DHS583
AACCCCAGAGCTCAAGCAACCCTCCTGCCTGGGCCTCCCGAAGTGCTGGGATTACAG
GTGTGAGCCACCGTGCCCGGCCTAAGCCTCTCATAAGGCCGTGCTCCCTGCCAGAGG
CCACCCTTGAAGAAGATTCCCTGAGACTTAATGACCTGGGGCACCCCGGACTTTCTT
AATCGTAGCTCCACCCACTCGAGCCGGGTGCCTTCGCGTTTCCCGTTTTTAAATGAG
ATAGTGGCACACCCG
>non-DHS584
ATCCTAGCCTAGAAGCCCTCTCTCCCTCTGGGCTGGAGCTCAGTGAGGGACAACTCT
CTAGGGACACCTGTACCAGCCCCACCTGGCGCTGAGATCCCTCAGACAGCATGGCCC
AGCCCTGGCCAGAAGCATCGCTCCCCTTCAACCAACCGCGTTGATGGACACCCACTG
TGTGCCAGGCCCCAGCGGGGCCCATGGGGGAGGTGACCTGGGTGAGGAAGGTCATTT
GGGTTTT
>non-DHS585
CTGCTAAAACATGGCCTTGGAGGGCCCTCCCCCAGGCCCAAACCAAATTTTTGTTGT
CTTTATAGCACTCATCGCTCATGTCTGCAGTGTTCTTGTTTATTTGTTTACTAGTTC
ATCGTCTACTCAAGTAGAATGAGCACCATGAGGGCAGGGATTTTCTCAGCTGTGTCC
ACCATGGTATCATGAACTCAGTACCAGGCTTGGCAAAAAGCGTGGGACTCCTTAGAA
CCAGGTATGATTCTCCCAAACAACCCTT
>non-DHS586
ATGGCTCAGACCCTAGACAGAGTTCTATTTTGTAAACCAGCCCTTCATGAAAATTTT
TTTATTCTTTGATCACATTATAATATAAAGAATATTATTAAAGGTTTTCTTGAATAA
TGGTGAATGTCAATAATATACAGCATTTGAGCTTGATTTTGAAACCTTAAAAAACTC

ATTAAATGGCACTGCATCACTGTATCTTTTAGCCTTTTTGGAGGAATTAGATACTTA
AATGTCTAAATGAATCTGCCCTTTATATTATGATGTACCCC
>non-DHS587
GGCCTGAAGGACTGAATGATAAAATTAGTAGTAACTCAGTATAAAGCAGAATATTTC
TTTCCAACTCTGAGGACTGTGGTGCACATTTGATGGAAAGATGGGAAGAAAATCAAA
TGCACTAAACTTGTTCCAGGGGCAAGCACAGCAAAGTATCTGCCAAGAAGGGGGGAT
CCCAAAGAGAAAATTTATCAGCCTCAGTTTTGCGTGGGGTTAGCCTTGTTCACAAGG
AATCCCAGGTATCCTGCCAATAGCATGGGATACATTGTTATGGG
>non-DHS588
CCCTCTTCCATTAGGGCATTAGGGACCCCAGATTGCAGGCTGTCAGACTCAGCCTTC
CCATGGACCCAGGCTCCTGAGCCTCCAACGCCTGCCCCCGCAAGGCTCCCGTCCCTG
GGCTCCCGCCCCCTGGCGGGTCTCTGAAGGGCTCTTTGTGGCCACACAGAGGCCCCA
TTGAGCTGCGGAGGCTGGGATGGGGGAGGCTGTTTACTCACCTTCACACCTGGGCCA
GGAATCTGTGAGTAACCACCCT
>non-DHS589
AGACCATCGCCATCATTGAGACATCCCAGCCACTGGCGCTTGAGGACACCCTGCAGC
TGTGCCAGGCTGCACTGGGGGCCAGTGAAGCAGGCGGGCTCTTGCAGTTGGACACGG
CCTTCGTGTGACGCAGCTGAAAAGCAACAACAAAAGGGTTTGGTTGCAACAGCCAGT
GTGGGTACCTCTGGGGAGAGAGGACCTCCTCTGACAAACTGGTCTGGTACCCACCAT
>non-DHS590
AGTCTCACCTGTGTCCCTGTCTGGATCCTTCCCCAGTGTGGCCAGAAAAAAACCCAC
CCCACTGCCTCCCAGGAATCAATGAGTAGAAGAGGTGACACCTGATGGGGAAGGAAG
AGTAGGGAGGTCGGGAAGGGTATCAAGGAATAACACCCTATTGTGGGCTTGCGGAGA
ATGGGGGACTTCAAGGCGTGTCAGTTTCAGGAGGGTGAGGGCAGGAGCGTGGGTGGA
GTCAGCAGGTCCCCATGAT
>non-DHS591
AGAAGAGGAAGAAGCAGAGTCAGGAAAGCCGGATGCCAGAGACAGTGCCCCAAGAGG
AGATGCCAGGGCCGCCACTGAATTCAGAGTCTGGGGAGGAGGCTCCCACAGGCCGGG
ACAAGAAGCGGAAGCAGCAGCAGCAGCAGCCTGTGTAGTCTGCCCCCGGGAAACTGA
GGAACTAAAGAAAGCTGAAGGTGCCCACCTGGGCCACCAGAAGGTGACACCCCCAGA
ATCCCTCCCCAGAGA
>non-DHS592
GCTTGATTGAAATTCACTGCTCACTTGATACGTTATTCAGAAACCCAAGGAATGGCT
GTCCCCATCCTCATGTGGCTGTGTGGAGCTCAGCTGTGTTGTGTGGCAGTTTATTAA
ACTGTCCCCCAGATCGACACGCAGCTAGCCTCCTGCATTGTATGGTTATAAATAGCA
CCCTAGTGAGTGCTGTCGTCCAGTCTCTCTTGGGTGTGGTTTGAATGATCTCGGGAT
GCATGACTAAAATGGAATTACGATGGCAG
>non-DHS593
CTCAACACCGACTTGCAAAGAGAGCTTTGGGAAAGAGCCAGACACGGCGCAGAATCC
CACTTCCACCTCTCCCTAGCTGTGTGACTTTGTGCAACAGAGTCCCCACTCCTTGTC
TGAGACTCAGTTTCCCTCGCCTGTAAAACAGGGCGTATTCAAGAGTCCAGGAGGATT
GTGTAAGTAAAAAGCCCACACTTAGTGCACAGCCCTGACAGGCTCAATCAGCGGTCA
ACTTTGTC
>non-DHS594

GCTCCCTACCCTTGTTCTCAGTAGGAGAGCCAGCCAGAAGAATCCTTCTAAAAATTA
TAGACGTCCCACGCCAGTCCAATCAGGTCCTGTGTGACCTCTTGAGATCACCTCCAC
TGTCTTCCCCTTGCCCATTCACCTCCACTCACCCTGGTCCTGCTGTTTCTCAACAGC
TCCAGGCACGTCCTGGCCATAGGGTGGTGGTCCTTGCCCTTCTGTCTGGAACTCATC
TGATAGCCACACAG
>non-DHS595
ATTCCGTGACCTCTTACCTCCTCCTCTCCTCCAAGCTTTTGCTCAGACATCCCTGCT
TCAGTGAGCCCTTTCGTCAGCCTTTTACAAATCGCAGCAGGCTATTTCTCACTTCCT
GTTCTCTCTCGCCTCTCCTTTTTCCCATGGTGTGTATAGTCTTCCATCGCATCAGTG
TGCTCCCATGGGCATGCACACATAAATAAACTTCTGTTTGCTGTGATTTTGTGAATC
TGTCTTTCATGACCCCAATGTAC
>non-DHS596
TCTTTCATGACCCCAATGTACAAGCCTCCAGCCAATGAATCTAAGATCAATAAAGGG
ACAGAGAAAAGCTTTTCCTCCCCTACACTTTATTTTCATTCACTGAAATCTTAGCCA
CATGAGGACAACAATTTTTTGTGTATCTTTTGCTCACCAACGTATCCCCAGTGCCAA
AAATAGAGAGTTTGGCGTAATATTTGACACTGGGATATTTCAGTGAGTAAATCCAGA
AGGACTGTGTTATGAACTGA
>non-DHS597
AGATGTGGCCTGCCTATCCTACTCCCCACAGGACTGAGGGATCCAAGAGAACCAAGT
GCTGGTTATATATGCAGCCCACCTTAGCCCCTACAGAATAGAGGTCCTAGATGGCAA
AGTGGACCATCCTGTTCCTGCCCAGGACAGCCTGTGGGCCGCATGGATGCCACCCAA
GAACAGGGACGCTGAACCCTGACACTCACATCTTGTCTATGAGGGCAAGGCACGCAC
TGATCCAGGTGCTCACAGCT
>non-DHS598
AGTATCTTGGGAGAGGTGATGGAGAAGGGCCCACAGCTCACCTGAAGCTCCCAGCTC
CTGAAAATCCCAGGTGGACTTTGCTTGTTCACCCCTGCCCCAAACCCCCACCCTTAG
CTTAGTACCAAGTGCCTCTTGCCTTAGTGATGCCCATCCTCCCAGGGACCCCAGTCT
GGTCCCACATCAGGCCAAAAGTCACATGGGCCTGGAGACTAGTGTCAAATCTCTTTT
ATTACAGAGGGGTTGTGGTAAAAGATC

>non-DHS599
GAGAAACCAGCATTGTGTCCTGTGGGGCCCTGGGCAAGACCCCAGAAGCCCGCCAAT
GGCGGGATTGGGGTAGGAGACAGGGTCAGCAAAGGCAGGAAGAGTTGCTGGAGAGGT
GCCCTCATGCTCTTGTCCCTTCTTCTCCCAATGTCTTCCATCTCTTGGGTGACACTC
CTGCCAGGACTTTGGATTCAACCCCTCCTTCCCCTCACCTGGCCTCCCAAGCACTTC
TAGGCTTGCCCTTCCTAAACT
>non-DHS600
GGGGAATCCACATTAAGAGAGATGCCTTTAATAATTTCATCTCTAGCAAAACACTCA
TCCACATAAGCACACACTTGTGCTTGAAATGGAATGAAGATGAGCCGATGTCATCAA
CCAGCATGTTTCTGTGTGAAATCCAGATGCTATCTGAGTCTCTAACGAGACTTATGA
ACCAGGATGAAGAGCATGTCTCTTTCCTAAATGAGGACGGTATGGTGGCCATTTAAC
AGAGC
>non-DHS601
CTATCTGCCAAAGTGAGAGCATTCTTGGAATTAGGCTGCCCTAATTCCTTTCCTGCT

ACTGCCATGCCTTTCCAGACAAGTTGGCTCCTGATCCTGTGACCTATTGATCTGAAC
AGCTCCTCAGCAACTAGAAGGCTTGGATACTGTGCACCGGATTGTCTCGTTGCCCAC
ACTAGTCTCCACGTTGGAGCTGAGCTCATTCCCACACTCTTATCCTATTTAGTCCTT
CATTACTGTACCATGGCA
>non-DHS602
GGGTACTACTGATGAGTGCATGTGATGGAAGAAACCACTCAAGGTTAGAAAGTAAAC
ACCAAAGGGTAACAGAGAAAACAGTCTTCAGGATTCACACAGGGCTGCAAAGCTCAT
GCTTACACCAGCCAAAGTGGAAAAAATTCATATTTACCGAGGTATCAGTTAGAATAC
TCTATATATACTCTAAATACTCCAATTAGAACATAGGAGTTGTCAGGTATGGTTT
>non-DHS603
TAGGTCATCTGGTTACGGTCAAACTGGGGATAGGTCATATGAGTATGGTCAGCAAGG
AAACAAGTCATTTGGAGGGTATGGTCAGTCTGGGGTGGAGTCAATAAACAGCCTGAG
CCTGGGTCATCTGGTGGCTTGGGTCAATCTGGAGCAGAGCCAATGAATGGTAAGTAA
GGGGCTGGATCATTTAGGGGTTTGGGTCAATCTGGGGTAGGTTCAATGAATTCTCAG
ACCG
>non-DHS604
AGAGGGTAGAGCACAGGGGTTTCCCCATACTACCTCCCCTCCCCAGGACACTCCCAG
GCTTGGGTTTTTTCTATAGGTTTGGCGGGGGGCCACAGGGAGGGGACCCTGACAATA
AAGAGATTGGATCCCAACCTGTTCTGAGATGGGATGGTTTGTGTTTTCTCATGAAGA
TATCCCGGCCCCTCTGCCAACCAAAAAGCTGGTCTAGGGTGCCTAATACTGATCCAT
CTGGACC
>non-DHS605
AGGGATCCTGAAGAGAAAAGTCAACACAACAGACAAACAGACAAAGAACAGACAGAC
AGACAAAAAAAAAAAAAAAAAAAGAAAAAAAAAAAAAAAACAGGAACAGAATGTATA
CTTATTTGGGAAAATGGATCATGGTTAACAACAGCAAATGTGAAATGCCCTTTCCTC
CCTGCCCACCCGTGAAAGTTCTACTCCTCTCCAAGTCACTAATTCGGGAAGCTT
>non-DHS606
AGCAAAATCCAAAAGCAAGCACGGCGGAATCTTCTGGAAGGGGGCTAAGATGGAACT
CAGGAGGCGGGGGTCGGTATGGAAAGAGCAGATGGATTATTTTTTTCCTCTCCTGGC
GAATGAGGAGCGCCCCCAGCCACCCCTCCTCATAAACACCCCCCAAGGCGCGCATGC
GCACTTAGGTGGCGGGCGGGTACTTAAGGCGCGGCCACCGCGGCTGCGGCAGTGCGC
CCAACAGCGGACTCCGAGACCAGCGGA

>non-DHS607
GCACCAAAACTTTGTCTCTTGTGATTTCTTTAGCGGCATCAACATACACTTCTAAGA
CTCAGCTGATGTGCCCACTGTGGAACAGGCACACTGCTTGGGGGAGGGAGGAAAAGG
AGGGCCATCAAAATTGCAATAAGCTGGGCCCCTCACACACCCAACCCCATCTCAATG
CTGTCCTGTGATAAAGGACCATCTCCCCCTCTCCCCAAAGAGAAAGGCTGACATCTT
CA
>non-DHS608
GGGAGACAGCAGGGAATAGAGAAGCCAAGAAAGAAATAGTTCCAGCAGAAATGAATG
TGTTATGGAGAAGCAGATGGCAGAGACGTAATCCAGGTTAGGGATGAGAAAGGTTGT
ATTTGAGGAAGTGACTTTAAGTCTGAGACAGGACATTCCAGGCAGAGGGAAGAAGGA
GGTACGTGAGACGAGGCTAGAAGCATGGGCAGGTCAGTCCAATGCCTTCGTAAGGCA

TTTGGATTTTATCCTC
>non-DHS609
AAACATACCAGGGCATTCTTCAACATCAGGGCAGCTGAGCCTGGCACCACTCCAGCT
TTCATCTCTAAGAAGCTCGAATTTGGGTCCTTTATTAGCTCTCCCATCTTTATTAGT
GTCCTGGAGCAGAGTGTCTGCAGCTAAAGTTATTGAATTTTCACTAAAACCTCAATA
CATGCACGACCCTTTTGTACTTTCATGTTCAAAAAGGTACAGACTTCCAACCGC
>non-DHS610
CTTAATATTGAGGCTGCAGCAGGTGCTGGGCTGGACTGGCCATTTTTCTGGGGGTAG
GATGAAGAGAAGGCACACAGAGATTCTGGATCTCCTGCTGCCTTTTCTGGAGTTTGT
AAAATTGTTCCTGAATACAAGCCTATGCGTGATCTTGTGTGTCAGGCCTGTGTTTTC
TGAGAAAGCCCTCCCATTATCCGGGGCAGAATTCAGGGGCAGAGTTCTCTGTGAACC
TCACAAGGTGAAATCACATG
>non-DHS611
ACAGACAAGGCCTGTGCTAAAACATGACACATACAGAGCACTACAAAGAAAAGATCA
TTTTTTTATTATTGCAATTGTTATTGTTACCCCATGAGAATGGGGACCCAGTCTGTC
TTAGGGACTGCTGTATGACCCGTGCCTAGCATGCAGTAGATGATCAATAAATACTTG
TTAAAGGAATGACTGATAACCACAGGCTCTGGACTCTGCCCTCAGGGATGCAGAGCA
GTTCCTTTGGAGCATC
>non-DHS612
TAGGGAAGGTGCAGTAAACGTACAGTATTGTAATCTTATGAGACCACAGTTGTATAT
GCAGTACATCGTTGACTGGAACATTCTTATATTATGCATGACTGTATATGAATTGTA
TACATATTGAATAAATAGAACTTTATAAAACTTTACTCATTGGAAGGCATGAAAGTT
TCTGACAAAGTTATAATGTATTATTATTCAATTCCAGAAAAGAACAATTAGCGACTG
GTGGTAGCT
>non-DHS613
GTCATTCCCTATTCCGTCACTGTTTCGTGGTCAGCACGGCACTTTTCATACCCAAAA
TCACTGTTTCAATACTAGAAGGGAAACTGCATGAGGACAGGCTCTTGTCAGTTCGCT
GCTGTGTACCCAGCACACATTATACGCCTAGTAAATAAATACCTGTTGAACCAACAG
ATGAACATGTTGAACCAACAAATGAACCTCCTCAGGACTTCTGATGGGATTGGGCCC
ATTTTCCTTTCACTGCGTCCCTCAT
>non-DHS614
GCGAGGGAGGAGATTCTGAGCGAGGGAGGGGAGCGCGCCCGGGCAGCAGTGCGTGCG
CTCCTCCCGGCGCCACAAGCTCGCCCCGCGCAGCCCGAGCCGGGCTGGGCGCTGTCC
TCGGGGGCCTGGGGAACCGCGCGGTTTGGAGATCGGAGGCACCTGGAACCCGTGGCA
AGCGCCGAGCCGGGAGACAGCCCGAGGAACCACGGGTTCTGGAGCTAGGAGCCGGAA
G
>non-DHS615
CTGAGTGTGGAGCGCTACCTGGCCATCTGCCACCCCTTCAAGGCCAAGACCCTCATG
TCCCGAAGCCGCACCAAGAAGTTCATCAGCGCCATCTGGCTCGCCTCGGCCCTGCTG
GCGGTGCCTATGCTGTTCACCATGGGCGAGCAGAACCGCAGCGCCGACGGCCAGCAC
GCCGGCGGCCTGGTGTGCACCCCCACCATCCACACTGCCACCGTCAAGGTCGTCATA
CAGGTG
>non-DHS616
AGGTGAGCTGGTACCGAGACAGGCAGCAGGTGCACTCCCAAGTCTTTGAGACGCTGC

TGGCAGGAAGTGTTCCCTTCCGGATCTGTCCTCTCACGCACATGAGTGCGCACACAC
ACACACAGGCACTTGCCATGGCACAGAGACATGTTCCACCAGAAGCCTGTGGACTAC
TCGCTTGGGCTTTAGGGGAGGAAAAGGGAAAAGGGAGGAATTGCTAGGCAACCAGCC
AGGGGTTGCTATGGCATTTTCAAGAGAG
>non-DHS617
GCATGAGTTCCGTGACGATGTACACAGGCTCCCCGCCCGAGCACACTGCGTGCAGCC
GGATGAGCCGCTCGTGCCGCAGGCCCTTCAGTGTCTGGATCTCCTTGGCGAGGTCAG
TGAGCTTCATGTTGGCTGCGAGAGAGGGTGTGGCTCCAGGACCGGCCCACGCCACGA
ACATCACTGCCCTGGGGCTTGAGGGTTGGACAGCAGGTGCGGGGCCCGGCCGTGGCG
CAGGATCTCGGGGCCTCACCTGACTTGATGACCTTGA
>non-DHS618
AAGACCAAACCACTGCTTTCTAGGAATAGGCTCAGATTTTTTTAAATCAATGCTTAA
AAAAGTATCAAGACCTTAAACCAGTACCGCGCTGGGCTGGTTCCTATTGGGTTGCAA
AAGTGATTGTGCAAATCTTTTCCCAACTTTATATTCAGGGACACTATGCCTGATATT
GGTTGTAGTAGAAGTATTACCACTGTAGAGTTTGGCAACCGTTACAAATCATGGCTT
TTAAAAAGATAAGTGTCCCACAACCCTAATCCAAGCTTCT
>non-DHS619
ATAAGTGTCCCACAACCCTAATCCAAGCTTCTTAGACTAGTTTTCAAAATTGAACCA
TTCATGGTAAATACAGAGAATGTATAAGTGATTGATTATATAAAGCAAGTACAATCA
CCTTTATAAGAAAGAAATGACGTATCACAAATAACTAGCTCTTTTAGGATTGTGTCA
CACACCTATCCATGTGGGAATCTGAAGAGTTGATTTTATATATAGACTTGTCAACTT
TGGAATAATTAGATGCCCTATATGGC
>non-DHS620
GCCCTATATGGCATGGTGTAGTTCATTGATTTTTCCAGAACAATGAGGTAGGTAATA
AATGTGTTTAAAATTTTAAAAACTAAAGAAAGGAACATTAGCAAAACAAAAACAAAA
ACAAAACCACACCCTCCTTCCCTCCTTTCTAATGTGCAGGGTATTTATGGAGGAAAG
TGTGGTAATTTAATTAGAATTTACTTATTTTATAAAAATGTTCAATTCTAGAAGAAA
CTTTGCTTTCAACTGTGTGTAGTATGCATG
>non-DHS621
AAATCAGCTTCAGTAGTTGGAGCAGTGCTGAGCTAGAAGTACTTGTCATGTTCTCTG
TTCTCTCAATGAATTCTGTCAAAACGCTCAGTGCAGAAAATTCAGCGTTTCAGAGAT
CTTCAGCTAATCTTAAAACAACAATCATAAGAAGGCCCAGTCGATGACACTCAGGGT
TCTACAGCTCTCCCACATCTGTGAACTCGGGTTTGGGGATGTTGGTTAAGTTTG
>non-DHS622
GAGATGATCGTGCAAATAATGATGCATACTATATTTGGGAAAAATAAAAGTTATGAA
ACAGTAGCAGGTCCAAGTTATATACGCAACTGTGTAAAATGTGTTCTTTAAAAATCA
GGGTTTCTGTGCTTTCAGCAACCTACACATGTTCTGACTTGTGATCTCAAGGTCTGG
GAGACATGTGTGGTGAAATCACACACACCTGCCCCAGCAGAGGGGTCACTGGACGTC
TT
>non-DHS623
ACAAACAAACAGCTTCCCTTTCCTCCTGTATCACCTCAGCTGCCTCCACTGAACCTG
GAAGAATGGGGCTTAGGGAAATTCAGGCACGACCTTTGAGGGTTGGCTCAGCAGGAG
TGGCCAGCAGTGAAAGATGGGCTAACTGTGCAAGAGGGGAGGAGGCCAACGAGATGG
CATCAGAATCCAAGGGCTTAGGACGAAGTCAGTAGACTTCAGCATGTGGAGGCAT

>non-DHS624
GGACTTGTCTGAAAATCCGAAGGCGTTGGATCTTCTCTTCCCCAGAAAAATGCACCC
GCTTTCAGAGGTTCGTGTCCCACGCTTTTCTTTCTATCCCAACCTTGTAAGAAGCCG
CCGCCGTGAGCGGAGCAGCACCTTCCTCCGCGGGTCGCGGGAGTCACCTACGGGAAG
GGACTCTCCGTCTGGCACAGGCCCTGTCTGGCCTGGGGCGCGCATGCTCCGCCGCCG
AATCTTCCAGAAAGCCGTGGAC
>non-DHS625
AGTGACTGGCAGATGAGAAGCTCCATTGTCGCCCCAGGCGAGTATGGGGCACAGGCG
CCTCCTTGGGTTGTCTGCCCTCCCGGGAGCCCCAGGGTGCCCAGGCGGGCCTCAGCT
GAGTCCAGGCCTCGGGGACAGTCCGTGCAGCCCCTCCTGGGGCTGGGGGCGGGCACT
TGTCCCAGCCACGTTTCTGCTGACTGAGCAGCCTTCTTCATGAGCTCACGCCTTTCC
AGAGAAATCCCTTAATGC
>non-DHS626
CCTATTTCCTCTGGGTCCCTTTATACCTGGGCTTCTTAACAGCAGCTAGTGGGAGCT
ACAGCTGCTGGCCTCCGTCAGCCTCCCAGGGACTGTCTCATCTTTCAGAAGATCCTC
GCCAGGGCGGCTCACCGCCACACGGACACACGGAACGAGTCTTCTGAGAGCCACATG
GGAGCTGGGCTGCATAATGGGAGGCCTGGGGCGCCCTTTAGACCCCACCAGACTGGA
>non-DHS627
CTCTTTCCAACCAATCCTCCTCCCCCACAGAGTTCGGCGTTAGCCTGTGCCTGTTAC
AACTCGCTTCTTGTATCCATTTCTGGTTAGTCTTAAGTATCTTCTTGAGGACATTGT
CTAAAAAGCAGCTGCATGCCATTGACATTCAGACAGAACAGGGCCCCTTGGCCTTAG
AAGGTGTAGGGGCCTTAAATATTCTGAAGGTGACTTCATGGAGTGGCCGCCCTCTGT
GGTCACCCTACCTGTGC
>non-DHS628
TTCCATCTGGAAACTCTTGCACGCGTTTGCACTGGGAGATGAAGTGGTCCCTGTTGG
CTCAAGCTGCAGTGAGAGGGTGGTGGGGGTAGACTCAGTCCCTTGCCGGGGTTGGCC
AAAGCAGGCTTCCTCTTCCTCCCTCAGGTGTGTGTTATCGGAGGTTTCATTCCTGGA
AAATCCAGTGTCTATGAAACCAACATACATGGCGTTTGCATCACAGTTGGAGTCAGA
TGTGA
>non-DHS629
CTCTTCGCAGATAAAGCTCCTGGCACCCGTTCCATGCTCCTCGGGTTTGTTCTTTAT
TTTAAATCCTTTAAAGTAAAAATCTCTGTCTCTCTCCCTCCGTCTTTCTCTCTCTCA
TTATCAACTTTTCTGCTCTCAGAAGCCCTTTTGATCTGGAGCCTTGAGATGCTCTGT
CACTTAGAGACAAGCCCCCTGCTTCTCTTTTTGACTTCTGTCCTTCCCTGTCCGTCC
TCTCC
>non-DHS630
AGAAGCTCAGGAGGACTCAGGACCTGGCAATGCCAAGAAAATGACCCCCTCCTCCAG
GAAGGCCACTGGGCTTCCACTCAGGTGGGGAGGCTGGGTGACCAGTCCCTGGGACGT
GAGCTGAGGCCAGAAGACATGCTGGCAGGGATCGGGGCCTTGAGCATTCGTGGGGGC
AGAGTGGGGCGCCTGATAAAGGGGGTCTCTGAGCTGGTGGGAGGGGGCCCCTGGAGA
GCTGGGTGGGGTGGGGGACTCAGAGTC

>non-DHS631
CTGGCGAGAAAATGCAGATGCCCAGGCTCCTGGGAGGCGTTCTGGTGGGAAAATGCA

GCTAGTCCCCTCACTACGACTGCAGCTTCACTCACCCACTGTGTCGGAGCCGGGTCT
CTGGGTGCTGACGGGCAGCTCCGCGGCCAGGCTCTCCCCGATGTACTTGTTCTGGGA
GTTGAAGCTGCACACAGGGGTATGACGAGGGACGGGGGGCAATGGCCCTCCGTTCAC
AATTTCCCCGACTGAC
>non-DHS632
GTTCACAATTTCCCCGACTGACACCGTCAGCTTCCTAGTAATAAACACCGACTTCCT
GGTCTTGGATAATCCCTCTGGTTCCAGGAATGCAGACCGGTTCAGCTCCACACAGCT
ACCCAGACCACAAAAGACCCCCGAGGGGTGTCACCAGGGGAGAAGACAGCTTCGCCC
TCAGTGCCCTGCCGAGTACTGCAAGCCCCTTGGGTGTCACCACAGCCATGTGTAAAT
AACAGAAAGCACAGGCGC
>non-DHS633
TCACCACAGCCATGTGTAAATAACAGAAAGCACAGGCGCCAGGTCCCACTGCCGCCC
ACCCTGGAGCCTCTGTAGGTCCCTAGAACCACTAAGCCCAGCGTGTTAACAGACGCA
CGCTGACTCACTCCTGCCTCCTTGCTGGCTCACCCAAAACCGTCTAATTTTCTGAGG
ACAGAACTGGAAGGAGGAGACAGAACTAGAAGGAGACAGAACTGGAAGGAGGAGAC
>non-DHS634
AACAGAATGCCTGTGTCTGGTGGCTGCCTGCTAGCTCAGGCTGCCTGAACCAACAGG
TGCCGATACCAAACCTCCCCCAGGGACGGCCTGACCACTGGGCTTCACTGTGGCAGG
AGGACAGAAGGAAGAAGTCTCCAAGGGACCAAGCCACCCAACGAGAGAACAAGGTCC
TTTTCCTTGGAGCACAGAAGTTCATGGCAAAAGTGTGGTCCTGCCTAAAATACATGC
TGTGT
>non-DHS635
GAAAGACTGAGCGAGACAGCACTGCCTCAGCACAGCCCAGGATGCATCGATGACCCT
GGAAGAGTGGGCCCCCAGTGACCCGTGAAGTTCCATGTGCCACCCTCACCTGCAGAC
GCTTGGCCACATACACACACAGAGACACAAACATACAAAGGAAAGATCCAGACATTC
AACGTAGAAAAGATATGGATGTGCTAAAAATCGCACAGAACCCGCTTAATCTCCAAA
CCTCAGAAATGTTAAAGGC
>non-DHS636
CTTCGGCTGCTTGGAGCTAGTGAGCCACCGGCCACGGCAGGGATTTGAGCAGAACTT
GGGGGCAGAGGGATGAGATGGTGCATCCCTTGAGGTCACTTCAGGAGATGACACTCC
TGTGGCTAAGTGTAGCTTCCCAGGTCATGGCTCCCAGGTCAGGCCTGGTCAATGGAG
AATTAAGTGGGCGTTGGGTGGTGGATGGATGTGATTTGGCAGAGGGGACAAATGTGA
CAACAGTGTAATAAGGGCCATG
>non-DHS637
TGTGCTCCTGTCTGGAACCTGCCCAGCCTGCAGCTCCATCCCACCTGCTTGCCTACC
AAGGCCACAGCTCCGGTGACTCCTCATTTCTCTTCTGCCTTATTTTCACTCTCCACA
GCTGGGTTCAGCAAGCATCCCGCTCATCCTCCTATCCTAAACACCCTCTTGACCCGA
TGTCCCTGCTAGCTACCTCCCATTTTTTCTACTTCCTTTTGCAGCAAAAACTCCTCA
CAACAACCATTCCCATTC
>non-DHS638
ATTCCTCTCCCTGTTCTCTCCAGCCACCATCAGGCTGAGTCTCTCCCAAGCTCCAGG
TCACAAGCAGCCCCCTGGCCCTGCCATCCTGCTGTCTGCTCAGCAGCAGGCCCTGTC
CCTGCTGACCGGCCCCTCCTCTCCAGTGCATCCAGGAGCCAGCACACTCTCTGGGTT
TTCTTCCCACTGTACTGGCTGCTCCTGCTCCCCTCCTCAGCCCACCCCTGTTGGGCA

GTCTGGTCTTTTCACCTACAC
>non-DHS639
GCAGTCTGGTCTTTTCACCTACACTCATCCCCAGGGGACTGCAGGCAAACATAGATG
GATGTGGATCACCTATATGTCAGCAACTCCAGGTTCACTGCTCTAGCTAGGCCCCTC
CTCTGTGGAGCTCCAGATCTGTCTCCAGCTGCCTACATGATGCCCCCACCTGAACAT
CCAGCGGGCAGCTCAAGACTTAGCACCATGTGTCCAAAAAGTAATTCTTGTCCTTCC
CTCCCGTGCTGCACCTTCAGTCTTCCCCTCATCCACT
>non-DHS640
CCCTCATCCACTGGTGATAACTCCGTTATTTCTACTTATTCAAGCCAAAACCAAATG
GAGTTATCCTGGGCTTTTCTCTCTCACTCCACATCAGTGCATTAGCATATCAAGGTG
GCTGCTCATTCAAAGCACACACCCAATCCTGATGGCCTGATCACCTCTCACCCTGTC
ACCACCACCCTAGTCAGAGTCACTGTCCCTTCTCGCCTAGACCACTGCAACAGCCTC
CAGCTGCTCTTCTGGCTCCTGTCCCTACT
>non-DHS641
GGTGATGCTGGTCTCCAACTGCCAGTCCAGCGTCCTCCACACCTTTAGGCTGCGGTC
ATCAGCTTGAGAAGCTATGTATTTACCAACAGGGTCCCATGTCAACCCTTTGACCAA
GCCAGAATGACCTCTCAGAGTAGCTAGAATTTCTGTGAAGAAAAAAGGAATGTGGAG
AGGTGTTGTCTGAGGGAAACATGACCTGCAATCCATGGGCCATGGGATGGATATGCA
AGAGAGGGGACTCAG
>non-DHS642
CCATTTCAGTCTTCACACCAGATGTGATGGTGTCCCAGGAAGAAGAGGCCATCTCTT
CCCATGGCTCTCTTCGTCAATAGAGAGTACATTCTAGAAGCCCCGTGGTAGGCTTCC
CTATGGCTTGCACAGGACTGAATCACAGAACTGTGTCCTCAGCAGTCACTGGCAGGG
GACAGGGGTCACCCGAACTGGGTGGAGGCCCGAACCTGACACCCCTGAGCTGTGCTA
AG
>non-DHS643
GGCTCCTGAACAGAATTGAGATTGAGGCCACAGGGCCAGGCTGACTCCCTCCTGCCC
CACGTGCTCTCCGGTGAGACCCTGCTTGGTCTGCAAAGCCGAGCTCGATGCCCTCCC
TACCTCTTCTCTCCTAGGCCTCATCCACGGCCTTGTCTTGGGCCAGACAGCCATGTG
CCATGTCTGTCCTCCCATGCAGCATCTGCACCGTGGGGGTGGGGGGGGAGATTCTCT
GCCACGTTCAGC
>non-DHS644
AGATTCTCTGCCACGTTCAGCACCTCAAAGGCAGGGGGTGCATTTCACTCAGCTCTG
CATCCTGTGCACCCTCAGCCATGCCGGCAGAGCATGTGTGGGGAAGGGCCCGAGGGA
CCCCAGGGAAGGAAGAGCACTGTGTCTGCATCCAGAAGAAAGTTCTGAAAAAAATGG
GAACCTCTGTGACTACAATGAACTTGTAACAAAAGCAATGACTTTGCTCTTCCAGGA
GGC
>non-DHS645
CTTGCTGGTTGGGAAGTCCTAGAGGGAGGACTGGGTGGCACGCTGGCACTGTCCTGC
CAAGAGGGCCACACGCAGCTGCTCACCAGGCGAGAGGGGGCAGGACTCACTCAAGAC
GGGCCTGCAGCTGAATGGCTGTGAAGATCCCTGGCTGCAGAACGAGCCCTTCGGCAA
GAGCCCCGGGGAGGGGACAGAGTAACTCCTTTGCCCTGCAAGGCCCCGCTTGGGTCT
CCCCAGGTTCAGCTCC
>non-DHS646

GGCCTCCAGCTTCTAATGTTCTAATGTGCAGGGGCTTGGCCTGACCAGTCCTACCCT
GGAACCTTTCCAGGCCCCATGTCCTGCTCCGCTGGGCTTGCCTGTTGGGATTCTACT
GGGGTCATGGAGGTGGGTGGCCCACAGAGCGGAGCTCGGGAGACCCCCGCAGTCCCT
GGAGTAGCCACATGGGAGGGAGGCCCGGCTGGCAGGAGCTCCCAGTCCCTCCAATCC
TGTGGGTCAGAG
>non-DHS647
GAGTGCTGAGACCCCATTTTCTGTCGAGGCGGGCCGAGTCTTCCCTTATCCCCAGAC
GCCTAGCGGGCAGGGTTGGGCTGAATCAAATGGGAGCCCTCCAGACATAAGGAGGCC
AGAGGCTGCAAGGAGCGGGGTCGTGACCGCTTACACCCCTTCTCCACAGCCCGGCCC
GACCTGGAGGGCCCCCGGGGCACTGGGCGGTGAGCCACCTCCTGGCAACTCTCGGT
>non-DHS648
GTGCAGATGTACACAGGACACAAGCTTGGGCATACCCTCAGGCTGGGGCACATGCAG
GAGCCCCACCCCCATGCCCCCACCGACTGGCCCAGGCCTCTCTGGGTGCAGACTGCC
AACACAGGCACGTCTCCTGCAACACTACCAAATCTGCGGCCACCAGCATCCCTACCA
CCCGCCTCCACGGGACTGTCCTTCCTGCAGGAGCAGCTCCTGGGGACAATGGCTGGT
CCTGCTCAGAGAGGAAGGAAAGGG
>non-DHS649
GACATAAGTCATTGTCACTGTCATGGCCCAGAATGACTACTCAGGGCAAGAAAAATG
CACTAAGGACAGCTTCCCTTTTCTGACATGGCCCACAAAGCTCCAGCTGCAGGCGGC
AGGGGTTGTGGGGGGCAGGACATGAGGCACCCCATACAGGCAGCAACACACACCACA
TCCCAGACAGGCAGCACTATTCGTATACTCACCCACTGCGGCCCTGGACTCACATGC
GTTCCTGAACCTCAG
>non-DHS650
CCGGAGGATGCACTATTAGACCACGAGGCGAGGGGGCTCTGGCACAGGCATCCCTGG
GGCAGAGGCCAGGAGCCCTTGCCAGTTTCTTCCTCCCCAGAAGAGGTCACGATGAAC
AACATGCCCCCCGGTCAGCCTGGCTCTGCAGAGCTGGAACAGAACCAGATGTGCAGG
GCAGGCTGGGCAGGGCCGGCCATCACAGGGCAGCGTCTCCACCTTCTGGGAGAT
>non-DHS651
GCAGCTCAGCTCTCTGTGCTTTTTGGTGTGCAAACATTTTCCAAAGAGGCATAATAA
CTACTGCAGGATGAGCCTAATCACAGGGCAGGCTTTCTTGTTCAAAATTGGAATGGG
GGGAGGAAGCAGAAACAAAAGCATTAATTCAGGCCCACGAAGCCTAGGCTTAAAGAG
CAATCCATCTGCCTCTTGAGCTTGAAGGCCTGCTCTCCAGAGGGAATACTCACACAA
AG
>non-DHS652
AGGATGTGTTACCGGGCTCACGGAGTGACTCAGGGAACTAGTGCCGCCCCAGGGCCC
CAAGGTGGGCGGTTCGGTGATTCAGAGAGGGCAGCTCTGTGTTAGGACACACTGGGG
CCAGCCAGGAAGGGTGGAAAAGATAGGGACCAGCGTGAGCATAGAGGCTAAGGGACC
ATGGGAGCTCCAAGCGCGCTCACAGTGGGGACCAGGTCCTGGGGGCTGGGGACACCA
GGGAGGTGAAATAC
>non-DHS653
CTGAGTAGCTGGTAGGAGGCTTGCAGTGTCGGGCGACAGGCAGGACAGGTGCTGCCT
TCTTTCCCCTCTTGACCAGTTTCGTAAAGGAGTGGGCCCCTGGCAGCCTCCAAAGGT
GACCCATGCTCCTTTCTGCCCTTCCCTCCTTCTTTCCTGTTTAACTCGTGCAGGTGC
AGTGGTCTGGTGTCTTTCAGTCCGCTGACGTCTTCTGTATCCCTGATGACCAGA

>non-DHS654
AAATTTAGCAGGATTGAGGGTCAGAACTTGTTCTCCCAGGAGGAACCTTGGTCAGAA
GGTCCTTGTGTTTATGCTGAAAAGCCTGACAGGGCAGCTCAGCCAAGAGCTGGGCAG
GGGCTGGCTTGGCTGTGCTCAACTACCCTCTCAGCTGGGACTTGAGTCCCTTAGCGA
ATGTTCTGTGCCCAGTCCTCTGCTGGGCGCTGGTGGACCCCAGAATGGGGGTAGGGT
GGGGACAGGGGACAGCTGGGAGCAGACATTG
>non-DHS655
ATTAAGAGCACCTGACATGAGTGGGACCATGACCCTCCGCCAGCTCTGCGTAACCTT
GTGGTGTAGGGGGGAACCAGCAGGGATGCTGCCCAGTGGGCTTTTCTAGGGGGAGGT
CCTGAGATGACTTCTTAGCCAGTGACCCTGGCTCACGCATGCTGTCATCCTTTCCTG
CCCTGGATGCACCTGGAGATGACCAGTTCCTCGGCTCATTGCATTCTCCAAGGCCGC
CTGGGGAACCAAACTGCCCACAACCATGCTTAT
>non-DHS656
CATCTGTGTAGCCAGCAGTGTCTTGTGCCCATCATGGGCCCTTGGTAGACAGGGAAA
GTGGCAGTGGGTAGAGGCTGGCTTCTGGGTAAGGTGTCGAAGAGGCTGTGGGCTGGG
TCGGGGCGGGGTGTGAAGGGGGCATGCAGGGGGCTTGGCATCCGCTGTGGCAGGCTG
CAGTAGACGAGACCCCCCCCAGCTCCCAGTTCAGATAGTGACGTGTAGGGGTATCC
TG
>non-DHS657
AAGTCAGCATGGGGTTCAGGCCCCGCCTGCCTGATGGGCGAACACACCCCCAAGGCC
CCCGTTAGGGCCAGGAGGCAGGCTGGCAGCCAGCGCAGGTGCAGAGGGGCCCCGAGG
CTTCCATCCTGAGGTCAGCACTGGGAGGACAGAGGCTGCCTCCTTGCAGCCCGAGGG
TTGACCAATGGCACTCGCCGCATCCCTTCCCAGAGCAGGCCAGCCCCACGGGCATCA
TGAGGGCCTGCGTTTGGTTTAATGCTCCGCT
>non-DHS658
CTCCCATCTTCCCAGCTTATAGACCTGAATATCCAGGTTGAGAATAAACCATTCTCC
CAGGGCACCATAGCCTCCAGGAAGCACCTCCTCCTTCCACCATCCGCCTTTCAGGAG
GGGCCTCCCTGGGGGGCTGTCTCTGGGACTGCAGAGGCTGCTTGGTGTATCTGGGGC
TCTGACATGAGTGAGATTGTTCCGGGGCCTGTGCTGCCCAGCCTCTTCCCCAACAGC
TTCCT
>non-DHS659
TTCCTACTTCTGGCTGTGTGATTTGCGACACGTTACCCCACGCACCTGTACCACAAC
TTCCTCATCTCCCAAATGGGATGACAGTAGTTAGCAAACCTCCCTGGACTGCTGTGA
GCATTCTGCCAGAAAAATGTACTCCAAGCTCTTGAAGTAGGCCTGGCGTTTGGTTGG
TGCTCAGTACATATTCAATGTTTCTGTCGCTGGCGTCATCACTGTGTTCATGGCAGA
G
>non-DHS660
GAACTGTGGACAGGACCCTCTGGGGGGACTAGGCCAAGGCTCTCCCACCTCTGGGGA
CCCGCGCCCATGGGCCTCAGATATGTGGCTCGAGAAGGGGGAGTGGGGGCAGGGCCC
CTGGAAAACCCCTCGAGGCCAGACCCACCACTCCCAATATCCGTAAGACCAGGGGCT
TGAGTCTTTCCTGCAGACAGATGTGGGCAGGAACCCACCCGACTCCCAGCTACGTGG
GGACGTGGACGAACTAACAGGACAAATTC
>non-DHS661
ACATGAACATGAACTCAGTCAGCATCTAGCTCATGGCTGCTGGAAATAGTTGGCAAT

CTCTCTCTCTCCAACACAGCTCCATCTTTCAGCATGAAGGGTAGGGAGATTTCTCAC
ATGCACCAAGGTAAGGTGGAGGGGTCCCTGGAAATCTAATGGTAGCTGAGTCTTGTG
GGCCTGAACCACTGTCCCTTGCTCTGCCCAGAAATGGATTTGTGAGATCACAGCCTA
AGTGACTGATGGA
>non-DHS662
GGCCAACAAGGGCTAAATTCTATCCCCCCCCCTCAAAAAAAACAATAACGATTTTG
GGAGTATTAGAGCAGAAAACGTTTGAAAACATGAACAGCTCTCACATCAACACAAGA
CATGAAACTCTGTTGGCAAGAGAGGCCTCTTTGGGGAGAGTGCTCACTCCTCTCCCA
CCCACTCTCTACCACCTTCAACCTCCTGTCTGGGGCACAGTAACAAGGGGTGTAAGG
GGTGTAC
>non-DHS663
GTAGAACCCAGAAAGTGGAGAGGAACAGAAGTAAGAGAACCATTCCATGAGAGAGAA
GGTCTGGTGTCCTTTCTGGTTGTTTCCCATAGTCAAGGTCATGCTATCCTGGGGTTT
TTTGTGCAGCATCTTCCACATCAATAGGGGATGAAATGTTCTCCTTTAATTAGAAAG
CCCTGGTCGGCACCAAGGAACTCTTGTCATACCCCTCTGTGAATGTCTCTTGCCATC
CTCAGTTA
>non-DHS664
GACATGGTAAGTAAGTGCCAGTGCAGGGAACAAATTCAGGTCCGACTCTAAAATCTG
TCTCTTTCCAGATCTCAAGGCACACCACTGAGTTGCCTTTCACCTTTCTCCCAAGAT
GAGGATTGCTCTGGCATGACTTCACCTTTCTCTTCTGAAGCAATTCCGAGTTCACTT
CTCTGCCCACTGACAGTTCACTCTCCTTTCCCTGCCACTGTCCCTTCTGCAAACGCA
CAATCTTTTACC
>non-DHS665
GGTGTAGGGTGTGGAGAGTGACTACGCAGAAGGGAGGGACAGGGGAGTGGACACAGG
CTTCCGTCCTGTCAGGTGCAGTGCCCGCCACAGGCTTCCTGAGGCAAAGCGGCATCG
GTTTGGATTCCCGAGGCTTCATCCCTGTCAACAAGGTGGGGTGGATGGCACTGGGTG
GGGAGGACCCGGTGCTGGGCTGGGAGTGGCAGGAAGTTCAGGTCAGGGCCCCTGTCA
CACCCATGGAGCTCT
>non-DHS666
AGAGCCTCCAAAGAGAGCTGAGGGGATGTGGGGCCCCAAACTCATTAATTCACTGAA
GACACAGGTCCCACAGGGAGCGGATGATGAAGCAGACCCCCTCCTGTCATCACCCTC
TCCTGGTGTAGTGTGGATGCGAGGCCACGGCTCAGTGATGGGCTCACCACCCAGAAG
TGGGGAGAGACTTTGGGCCTCCCACCCAGTGGGGCTTGGCCTGGCTTCTGTGGCCTG
GGCGTGTTGTGGACTCAGGCACTG
>non-DHS667
CTGATGTCATGGCTGGACAAAGTCACGGAGTAAAGCCAGCAAAGCCACCCTCTTCCT
GTGTAGTCCTTACAGGCATGACTGGAAAGTTGGGGGGCATCTATGGTAGACATGGCA
CAGCCATGAAGAGACCAGTGGGGTGGTGCAGGGTGGACTTGGGGACCCTACCCCTGA
AGACTGAGGCCCTGCAGCTACCAGGTGGGCTAGAAGGTAACTGGAACAGGCCTG
>non-DHS668
GCTCCATGTCCAACTGCCATAGCAGCCTCATGGCCGGCTGTCTGGGCTCCCACAGCC
GGGACAGTGACCTAGGTGCCCAAGGCTCATTGCCACCTGTGAGGGACAAACTCCTGC
TGGAGAAGAACCTCAAGCGGCTGCTACAGCTGGAGAGGGAAGGGGTGAGAGCCAGGG
CCATGGCTGGGTGGGGTGGACTCCCATGGAGAGCCCAGGGCTAGGGTCAGCCCTGGC

TTGGCTGCTCCTGAACACTTCACAGAGTCACCCT
>non-DHS669
ACTGTCACCTGAGCCCCTAGTCCTCCCCCTCTTAGCTGCTGAACCCCTGCCAGCCCC
ATGAGGCTGGGGTGAGCCTGTGCGTGGCCTAAGCAGACCCTCGGCCTCCAGCTCCCC
TGCTCTCCCCAGAGCTGGAGCCTAGTCCCCGCCTGTGTGTCTGAGGTCGGTGCTGCC
TGGCATGGTGGGGCTGCCCCAGGGTGAGGGCAGGGGCTGAGCTGGGACCTTGGCTTG
TAGCTGCCATCGACGA
>non-DHS670
ACCTGGGGACAGCTGGAGTTCCGGGGTCCCCTGGGCAGCCTTGGGGGCTAATGACCT
GCCCTGGTGGGCAGCGCAGGGAACCACGTGCACACATGCGTCCGTGGAAGCTGCAGG
TTCCCAGAGCTGGCTGTAGAGGGTGGGCAGCTCCTGGTGTGCTCTTAGGCAGGCCAG
GTGCAGAAGGTGCCATGCTCTCCCTAACCATCCAGCCCTCCTCCCCAGCTTCATCAC
GTTTCGCTCCTGTGCCTACCTGGACAAGAAGCATACCATC
>non-DHS671
TGAAACGCCAGCAGTACGATATTAAAATCCACTTAGAAGTAAAAACTTCAAGACGGA
AATATGCCACCAACACTGGCGTCAGTAATCCATTTTAGAAAGCCTGCGCGGAGACCA
CCATTAGCACCGCTGAGGATATTTAATGAAAGCCGTTGGCTGCAAACAGAGGCGAAG
AGAGAAAAACGATTATTTCGGTGTTCTGCACACACGCGGAGGCAAAACGCTTTCAGG
ACCTCAGCAGCC
>non-DHS672
CTCAGGGAGGAAAATCAGGTTGCTGGCTAGACCAGGAGGGTGTAGGCCACCAGGGCT
CTCTCCAGAATTTGTCTGCTCGCTTCGCAAGATTTGCAGATGGTCCCCAATATTAAA
GCAGCTTCCAAACACATCTGGACATCAAAGGGGACAAGATGCTCAGAAGTCAGGAAG
AAGGCAGGACTCGGGCGACAGAGTTCAGAACTTGTGGTTTATTGACACTTCTGCATG
GT
>non-DHS673
AGATGGTCCCCAATATTAAAGCAGCTTCCAAACACATCTGGACATCAAAGGGGACAA
GATGCTCAGAAGTCAGGAAGAAGGCAGGACTCGGGCGACAGAGTTCAGAACTTGTGG
TTTATTGACACTTCTGCATGGTGGGTCCTTGGGAGACTGCCTTTCTGGCATCTTGGG
ACTTGTCCCTAAGAATAGGGAAGACAGTCATCCTGTCCTGGAGCAAAGCTCCCCCTT
GCACAGGAACACAACTCCCATGC
>non-DHS674
CTCAGCCCTGAGTCACTGCACCATGCAGATCCACCCTGATTCTGGTCCCTGCAGAGC
AGGCTCCTACACAAGGCAAGAAGATGCTCCAGGGACAAGCCAAGGTGTATGCAAGGC
CCAGGTGCCTGAGAAACACGTCTCACCGACCTTGGGGCAGGGGCTGTTACAAGAGGC
TTATAGTCTAGTGGCAGAGACAAAAACCACACTCAAATCATTAGTGTACGGTATGCA
CTGCTCA
>non-DHS675
TGCTGTGATTCCTCACACATAACATGTATCTGTTTTAGTTTCTTGGTTCAGTTCTCA
GGCTGTGGTGACTCAGGAGCCCTCACTGACTGTGTCCTAAAGAGAGAGACTTATTCT
CATGTGTGGCTTCAGCACCACAGCACTGAATTCAGCAGAAGCCTGGTCAAGTCCTCA
GGACATTGATGTGTAACACAGATAACAAACACCCCTGGATGCCCACTTGATTCTCTG
GCTCCCTCCT
>non-DHS676

CCTCTGTTTCCTCCTCAGTAGTGGGAAGACCCCCTGTCAGGTGGGCCAGTCCATGAC
ATCTACAGAGGGAGCAGGAACCTCTCCTATTTCCTGGAGGAGAGCTGGGGTGGAGGC
TGCAACCCAGGATCATCAGAGGAGCTGGGGTCTTCAAGGTTCCTAGGGACCCCTTAA
GCGGGGGTCAGAGTGGCTTCAGCGGTCTTATTGCTCGGTCCAGACAGAAGATGTTTC
CAGTTG
>non-DHS677
CCAGACAGAAGATGTTTCCAGTTGTGAAAAACGACTTCAGGGACAACAAAAACAGAG
ATTCGCCTCTCCAGACACCAGTGGTTGGTGTGCCTGGAGTACTCCTCGTACCAGGCA
GGGGAGAGAGTCCTAGACAGAGGAGGTTCTAAGTGTCACCTAGATTTCAGGCCTCGG
GGCCTGTATTGGGTAGGTGATGTCACAGTGAGTTGATGCTCTGTAGCCCCTTCCCTA
GGAGGTGGCAGAGGGAAGAGCTGGTGGTCCTCTGAGGTGTGAGTG
>non-DHS678
CGCCTCTTTTCATGACTCCTCGGCCCTAAGGGCTTTTCATTCATTCATTCATCCATT
CATTTGTTCAGACTCACTCATTCATTCATTCAACAAACTCTCAGCTGTGGGCCAGGG
GCTGGTGTATGCAGCCCAGGACCACCTGCCCTAGAGGAGCTCACAAGCTTGGAGAAC
GACATGGAGACACCACTGCCCCCACCCTGTCAAGTCTCCAAGGTTGGCACTCTG
>non-DHS679
AGTCCACAAGGACAGCTCCTGAGAGGCCATGTGTCACAGGACCCACCACGGGCCTCC
CACTTAGCCAGAGCCATGGCAGAGGCTCTGCCCCTCGTCTTTAGTACATTCACACAA
GGGTCAGTCAGCAAAGACAGTCACTCTGCTGCCCTAGGAGGGAAAGGCAAAGTGGAG
AAGCTGGCACCTACCAGCCCACACTGAGGGACAGACACAGGTGGCTGTGACCAGCAG
AGCCTGGGTCTT
>non-DHS680
CTGCAACTACAGTGCAGGATGGGCTGTGATACTCAGCACTGTGGATGTCTGTCAAGG
CAGGGTGTAATGGACTGGAGGTCAGAACGTGTGAGGCCCTGGATGAGCCTCTCACCT
CCCCACCCCCAGCCCAGGCGCTTGTCCCACACAGCTGCCTTGGATGTCATTAGCTGG
AAAGCCGTGAGTGCCTGATGGGGAAGGAGTGGGGTGTGGGGAGGTGTTTAGGCTGTG
ACAAAGGGATGCTAACTCCAAAA
>non-DHS681
GTTTAGGCTGTGACAAAGGGATGCTAACTCCAAAAACAGGCCCCCAGTCCAGAGGCC
TCTTAAAATGCAAATATGCCGCACCTGGGAGGGGGACAGGCCCCTGGCTGAGATGAG
GAAGCCTGGAGGATGCCTCTGTTCCCTCTCCCACCGCAGGCCCATGAGCTCTCAGAC
CAGCAGGAGGCTGATGCTGGAATATGCGCGCACCTGCTGGGGGATCTGTGCGCCTCA
GCTCCAAAGTACAGGAGCTA
>non-DHS682
CTAGACTCCAGTGTCCATGTCAGGTCCCATCCAGCTGGGATGCAGGAAACCAAAGGC
AGCCCTCCCATGACCCAGCACTGAGGGCCAGACCTGTTACCAGGAACGTTGGTAACT
GGAAGAGCCTGGTACTTGGGGCTGTTCTGTGCCCCACAAACAGAGGGACCGGCTGTA
AGGAGACTGGCCTGATGGGGAATGAGAGGGGCTGAGCCAGAGGTGCCGACACGGTAT
GTTTTAAGTAAGGTC
>non-DHS683
CCGACACGGTATGTTTTAAGTAAGGTCCAGGTGGCTGCAGGCGGGATGGAAACACAT
CCACCATTCATCACATCGGCCATTGCATCTCCAGGCTAAGGGGCCACAGGAGCCTCC
CAAAGAGCCAGGCTGACCTGGGCCCTGTGGCCAGCAAAGAAGTGTTGCTGGCTGAAT

CCCCATCTGCAGCATCGAGACCTCTCCCACTCCGTCCGGCTGAGCTGGCCCTGCATT
TGGGTAGCCTTCC
>non-DHS684
CAGTTCACCTGCACTGGTTCTCCTCCTGAGCCTGCCTTGTTCCGCTCTTGCCCCCAA
TGATCCCTTTGCCTAGCATGTCCTTCCCCATCCTCACAGCTCAAAGTCACCTGGGCA
GCTTCTTGGAAGTCTCCTCCTTCCCTGCATTCCATCTGTCAGGGCTCCAGGGTCGAG
TGGGAAGGGGTGTTGGAAACTCCATTGAGCAGCTCAAGGCCAGCGCCTCTGACCCAC
CTCATTCCTCCAAACAATGC
>non-DHS685
AAGTGACCCCACAGCTTAATCTTCCCTTCTCCTAAATCCCATTGGCACACTCCCTTC
CCCTAATTCTCTATTTACTTCTCTGCTTCCCGCAGTGGACTCCAATTTCCCTGAGGG
TGGACACCATGTCCGTTCTCTTTCCTTGTCTCCCCAGTGCTAGGCATATACCAGGAA
GCTTTCAGTACATGTAGGACAAATGAATAGTTAATGGACTTGAATCAAGCTTTACAG
TATAAAAGCACTTTCACCATCTCAGGTGATCC
>non-DHS686
ATAAAGAGGCCCATCTGGGTAAGACCTGTCCCGCCTGCTGTGGGGTATTAGGGCAGA
TGGGGTCTGAGGGGTCTGAGGGCTCTGAGAGCAGCTGGCAGCTCAAGGACATCCGGA
GTTGGAGGATGGAGCAATGCAGGCCCTTGTGGTAAAGACAGTCCTGCAGCCGCGCAG
GCAGGGATGCTGCAAGTGGAGTGCCAGGCGGGTGCGGAGCCCTGTGGGACTGTGGAG
GGGTCAGAGGGAAGCCAGGATTTT
>non-DHS687
GATTTTGGGGTCTCTGAGAGTTTGGAGAAGGGGAAGAAGATTAAAGCTTGTTTCAAA
AGTTTCTAATCAGGTGGGCAGGGCCAAGGGTGGCTGTGGGGTGAGACCCATGACTCA
GGGTGGCCCACTGTTACTCTATTGATTTTTGGGCGTTTTTTTCCAAATTGATTATTC
TTGCTGAATGAGACCTGAGTCCTTGACTGTCCCCTTAAAGCCACCTGACTTGTTTTC
AGTTCCACTGG
>non-DHS688
TAAAGCCACCTGACTTGTTTTCAGTTCCACTGGCCTGTCGGGCTGTTTTCTACTCAA
CTCCACTCTTGCTTGTCTGCCCTCCCTGCCTGGGGCCCAGCCAGCAGTCAGCTCAAG
GGCCAGATGAATTGGGTGGCTGTGCTCTGCCCACTGGGCATCGTGTGGATGGTGGGT
GACCAGCCCCCTCAGGTGCTCAGCCAGGCCTCAAGCCTTGCTGTGTACCTCAGAGCA
GCTCCGTACCCTGATGTCACAGCAAAGAAACTT
>non-DHS689
TACCTCAGAGCAGCTCCGTACCCTGATGTCACAGCAAAGAAACTTAGACATGACACA
AACTGTGGCTTCCCAAGGCAGCAAAGAATGGCCAGGGGTCATGAGGGCCGTGCCCCA
CTTTTGGACAGACCTACTCTAAAGTCACGCTACCTGCGTGCAAATCATAAAATCAAC
ACTTTTGAGGAGATCACAGCTATGCCTTCGTAACACAGCCCAGTCCGACCAGATAGA
CGGTGCCTCGT
>non-DHS690
GCCAGTGATTAAAGTTCATCTCACACACTCAGGAAAAATAATTTGACAGTTATTTTA
GGGAATGAAAGCTAGGTCGTTTCCCCGCATAAACAACATAAGCTTCTGACTCTTACC
TCCCTCTTGATTTATTTAACCTCTGAATAGATGGTCTATATTTAAATAAGCAAGTAA
AACACCGTACAAGTTAATACACAATGAAGACAGTACAGAGCCTATAGATTTGGG
>non-DHS691

CCCTCCTCTGGTCATTCAGGGGCAGGTAAGGTGACGCCAGCACAGATGGCGGGCGGC
TCTGGGAAGAGGCAGGCGGGAAATCCGACCCTCCTGACAGGCAAGCGGGGACCTGAA
AGGTGGTATCCGCCTGAGCTGGGAGACAAATGAGGAGAGTGATGGGGACCCAGGCCA
GGGATACAAGCAAGCAGAAGGCCACCAGCATCTATGACAAAAGTGTTTAATGCTCCA
TAATGTGCAGC
>non-DHS692
ACAAGTACCAAAGCACCGACACTTGAGTCTGGGCTCCGAGTGGTGCACGTTTAGCCA
AGCCACCTCCCAGCTCTGAGCTTGGGTTTCCTTAAACAGCAAATGGGAGGAAGGACC
CTGTCCTCCCCAGGTCACAGAATTAATAAAAGCTTCTACCGGCATGCAGAGCCGCGA
TGAAAGGTTGCAGAACAAAGTCATTAAAGTGATGATATTTACACTGAGCCATGCAAA
GC
>non-DHS693
TGCTGTTTGGTCTCTAGAAACAGGGTCACTTTTTTAATGTAGTAAAGAAGTAATAAA
TGGTGGCATTACACATTGTGATATTAATAGCGCTGTTGCTCATGTTTCCTGTAACTC
GCAGTGGCCCTGGGAAGTGGACGCCTCCCCTCTTCACTGCCCTGGAGGTTCAAGAAC
TCGGCTCGCCAGGCTGCTAAGTGGCAGGGCTGGGATTTGAACCCAGGCCTTCTGGCT
GCATTCCATG
>non-DHS694
GCAAAAGCCTCTACAAGACAGTCACCCACAGATATGCCCAAGAATCAGTACACAGTT
TCCAACCAGAGATCTCCAAAATGAAACACTCAGGGCTACACATAGGAAAAGCACGCA
CACACACACACACACACATACACAGACACTTACTTTTGTGTCCTTCTGGCTATGCTG
ACGAGTTTTCCTGGTGAAGCCCGGGGCTCACAGAGTAATCTCTGCAGACAACTGTGG
TTC
>non-DHS695
CCTGCACACCTCCAAGTCAGGGCACCACAACACGCCACCGGGAGAGATGCTGAGAAA
AACCCAAGACCGCTCGGGAAATGCAGGAAGGCCGGGTTGTGAGGGGTTGTTGCAATG
AAATAAATACATTCCATGGGGCTCACACGTTGCTGGGGAGGCCTGGAGTCAGGTTTG
GGGTTTTCAGATATCAGAAATCCCCTTGGGGGAGCAGGATGAGACCTTTGGATAGAA
CAGAAGCTGAGCAAGAGAACA
>non-DHS696
GGTCACCTAGTCCCCAGCACTCGGTGTTCTCATGGGATTAAATGAGGCTGTGCCGGG
GGTAGAGGGCGCAGAGCACAGCGCCAGGCACCAGCGTCTCTGGGCTCGATCCGCTTT
CATTAAGCTGTCCCCGCCCCTTTTCGCCCCCGCGGTCCGGGACTCACCGACCGACTT
GCCGCAGACGATGCTGACTGCGTTGAGCGCCCCAGACGAGGAACCGTAGATGCG
>non-DHS697
CCAGGGGACTGTGAGAAGAGGCGGGTCCCTCACAGCAAGTGCTCACTGGCCCTGCAT
CCCCAGAGCCCCCTGTGGTCAGCCCCTCCTGCAGAGCAGCCCCTCTGGGGTTTGCCA
AGCTCCGGGGACCTAGTGAGGTTGTGGCTGCATCTCCATCCTGCGCACCACAGGGAG
AAGCAATTAAATGTAATAGATGCAGAGGCTCCCGGAATTACATTATTTAGGAGCTGC
AGCTTACACACATATGCACGCGTG
>non-DHS698
AGACAAGGCCAGGTGGTTCCTGCAGTCCGACCCGAGAGGGAGTGAGGCAGACAGGGA
TGTGGGGTCCCCTCGAGGCTGTTCTCAGGGACACCCCCCTAGGCAGGGTCCCACCTG
ATCATGGCCTTGCTCAGGGCCCAACCTCCACAGCCCCTGAGGCCCCATGCTCACTTC

CCTTGGGGGCACTGTCCAGCTCCGGGGATGCAGACCCAGGCACCCTGCCGTCCACAG
TGGTGTCCTCATGCACTCACTGG
>non-DHS699
ACAGTGGTGTCCTCATGCACTCACTGGGCACAGACCCGCCTGCTTGGTCCTGGGAGG
AAAAGCAGATGGCACTCATGAGAGTTAACCCTCCCAAAGGAGAAGAGGGCTGCAGCC
GGGCCTGGCGTGGTCACCAGGGGCACCCACCGTTGGGAGGCCCGCAGGGAGCGGGGA
GTGCGCCCAGGTCTGGGGTTCAGGGCCGGCAGCAGTAACGGCATCAGAACTGTGCTA
GCGTCTGAATTGT
>non-DHS700
GACACACAGAAAGACCGACTCCTTGTGGGGCCCGGCAACGCGGCGGCACGCCCACCC
GCACGGCCAGGACAGGGAACGAGGCCGCCAGCCCTGACAGTCCCTTCGGCCCCTCCG
CAGTCCGCTACGGCGACGGCCCCCAGGCCTCCTCCCCGTGGCTCCAGTTCACCTTTT
CCAGGACACGGCAACGGGACCCGGCCATCTGCGGACTTTTAGACCTGGCTTCCCTC
>non-DHS701
TGAGATCACCCACTATCTATCACTGCTTAGATAAAAAGGGAAAGAATTTAAATACTG
TATAGTAAAAATTAACAGATAAGCTAACGATTCAATAGCCTAGCAATAGGTCTTAAA
ACCACACCTTACTTAAAACAGGTTTATAAACAATAGGGTCATTTCAGCCACAGTTTG
ATAAAGTAACATTGAACATGCATATTCTGTGATCCAGCAAGTCATCTACAAGGT
>non-DHS702
AATGAGAACAGCAGTTGTCTGTGGTGGGGAGCGGGATCGATTCGATAAACACATGAG
GGGACTTTCTGGGGGAAGAGAAATGCTCTACATCTCAATATGGGGGTAGGTTATAGG
GGTACGTGTATTTGTTAAGACTCATCCAACTGTACACTTAATTTCTGTGTATTTTAC
TGTGTGTAATCACACTTCAATAGAAAAGGAAAAAGGAAACAAGTGACAATGATGGAA
ATCAGG
>non-DHS703
CAAGTGACAATGATGGAAATCAGGGTAAGAGTCAAGACATTTGCGATCCAGTCCCAG
CAAGGAGATTAGCTGGGTGTTGTCACCTTCCACCCCCCTTTGAGTGTTCAAATTCTT
CTGGAAAACAAAATTTGGGCCCAGGGACTGTTTAAAAAATAGGAAGACAAAAATAAT
ACTAAAAATATTGAGTCTTTACTCCCTTACACTAGCTGGGGTCAGACCTTGTACATA
AACATCCTTTCCTAAAGGGG
>non-DHS704
AAGCCAAGGAAATGTGGATACAACTCATATACCATCAGTGACCACAAAGTCAATTGT
GGGTGCCTAGAATCTGTCTCACCTGTGCAGTTCACTGAAGACTCCTACTGCCTGAAA
GAGAGAGTCTCTACAGAGAGAATTGGGGTGAGGCAGTGAGGAAGTCATTCTTGCTCT
GTGGCTAACACTCAGCCTTCCCTCTAGCCCAAAGAAGAACAAAACTGGAATAACTAT
GGCTCTAAAAGCTCTTTCACATCTGCATCAGGAGTACAAACAC
>non-DHS705
CTCTCCTTTCCTGTCACTGTGAGTGACACACCAGCTGATAGTGTTATGAATAGGAAG
GTTTGGGCAGAGTGACTGTCAGAAATATTTTTACCGTGTGAAAGGACATTTGTGGAA
TGTTATGGCATCGTTGCCTCCTGCCCCTCAGAGAATGTGGCATTGAGTATTGGGCTC
TTGCCACTGTATCCAGCATACCAAAGCGAGCTCCTGGCCTCATTGTACATTACAG
>non-DHS706
CTCAATTGGCTTTTACTCTGGGTTATTCAACTAAAAGTTTCATTTTCAGTGACTCAT
AATATTGTTTACACATAATTTGTAAGCTCTATTTAATTACTTATGTAAATGACTAAA

CAAAAGATGGCAAATGCAGTGAATCTAAGACTGTACTTAATTCTGGTGTAAACTATT
CTAACAATGTAATTAAGAGCTGTCCAATTTACACTTTGAAGTAAACAAAAGATCTTA
CAAGAGATTGCAAGTGCCATC
>non-DHS707
CCCAAATGTTTTCCTTTGGTCTTATTTTGGGGAAATGGTGGGAGGTAGAGAAAATTG
GAGTTAAAAGAAGAAACATTATGAAAAAATTCCAAGCCAGCTATTTTATCTTATAAT
CATTCATCATGAGTTTCTTTGATGTTCTGGCTGTTCCGGTGTGCCTATGTTTTCTGA
TAAGGGCATAAATACACTTAGTTTAATATTAATTTAGCCTTTGTAAGCAGCACTTGA
AGGGACTTAAGT
>non-DHS708
CTTCCTCGTGTGAGGGGATCTGCCGGACCCCTGCAAATTCAATTTCTTTCCCATTCC
GGGCCCTTCCCTATCGTCGCCCCCTTCACCTTGGATCATGTTCAAGAAGTAAGGACA
TGCTGTGGCCTCCATCGGCTGCTCACAAAGGCGGTGGGGTGGGGGTGGGGAAGAGGG
CGAGAGCTAAGATCCTCTTTCTCTCTCCCCCGCCCCTGCCATCCTGACTCCCTAAGG
TTTTCT
>non-DHS709
AGAGTCCAATTGTGACCATAAGCAAATACATCCACCCTTACACATAAGCACCAAAAA
TTTGCCAACCCATAAGTTAAGTTCTGTTAGGTTTCTGTTTGTTTATTTGAAAACATT
AGTCTTCCCCTTTTCCACTAGGTATATAGGTAGAAAAATAAATGAAGTCAGCACTCT
TTACGTCTCACTAAAATAAACTGTGACTCAAATACTGTGAGTGGTTATTTGCAAGG
>non-DHS710
TCAAAGTACACTACCTGCCTCTGGTCCATGAACAAGGAAAACTTTTTAAAAGAAAAC
GGGTCATCAAACTAGGCTATTTTGGCTTGTCGGTTTTAATGCCTGTGGTTTCACATC
TCTTACTGAAGCCATGTTTTTCACCTGCTCTCTGACACAAAGTGCCTTCAAAGTCCT
GGTCTCTGGTGGTGTCAGGCTGATGATGCATTACCCTTCTCAGTGCTCCTCAAAT
